# Novel promoters and coding first exons in *DLG2* linked to developmental disorders and intellectual disability

**DOI:** 10.1186/s13073-017-0452-y

**Published:** 2017-07-19

**Authors:** Claudio Reggiani, Sandra Coppens, Tayeb Sekhara, Ivan Dimov, Bruno Pichon, Nicolas Lufin, Marie-Claude Addor, Elga Fabia Belligni, Maria Cristina Digilio, Flavio Faletra, Giovanni Battista Ferrero, Marion Gerard, Bertrand Isidor, Shelagh Joss, Florence Niel-Bütschi, Maria Dolores Perrone, Florence Petit, Alessandra Renieri, Serge Romana, Alexandra Topa, Joris Robert Vermeesch, Tom Lenaerts, Georges Casimir, Marc Abramowicz, Gianluca Bontempi, Catheline Vilain, Nicolas Deconinck, Guillaume Smits

**Affiliations:** 1 Interuniversity Institute of Bioinformatics in Brussels ULB-VUB, Brussels, 1050 Belgium; 2 Machine Learning Group, Université Libre de Bruxelles, Brussels, 1050 Belgium; 3 Department of Neurology, Hôpital Erasme, Université Libre de Bruxelles, Brussels, 1070 Belgium; 4 Neuropediatrics, Hôpital Universitaire des Enfants Reine Fabiola, Université Libre de Bruxelles, Brussels, 1020 Belgium; 5 Faculté de Médecine, Université Libre de Bruxelles, Brussels, 1070 Belgium; 6 ULB Center of Medical Genetics, Hôpital Erasme, Université Libre de Bruxelles, Brussels, 1070 Belgium; 7 Service de Médecine Génétique, Centre Hospitalier Universitaire Vaudois CHUV, Lausanne, 1011 Switzerland; 8 Department of Public Health and Pediatrics, University of Torino, Turin, 10126 Italy; 9 Medical Genetics, Bambino Gesù Pediatric Hospital, Rome, 00165 Italy; 10 S.C. Medical Genetics, Institute for Maternal and Child Health - IRCCS “Burlo Garofolo”, Trieste, 34137 Italy; 11 Laboratory of Medical Genetics, CHU de Caen - Hôpital Clémenceau, Caen, 14033 Caen Cedex, France; 12 Service de Génétique Médicale, CHU de Nantes, Nantes, 44093 Nantes Cedex 1, France; 13 West of Scotland Clinical Genetics Service, South Glasgow University Hospitals, Glasgow, G51 4TF UK; 14 Service de Génétique, CHRU de Lille - Hôpital Jeanne de Flandre, Lille, 59000 France; 15 Medical Genetics, University of Siena, Siena, 53100 Italy; 16 Genetica Medica, Azienda Ospedaliera Universitaria Senese, Siena, 53100 Italy; 17 Service d’Histologie Embryologie Cytogénétique, Hôpital Necker Enfants Malades, Paris, 75015 France; 18 Université Paris Descartes - Institut IMAGINE, Paris, 75015 France; 19 Department of Clinical Pathology and Genetics, Sahlgrenska University Hospital, Gothenburg, 413 45 Sweden; 20 Department of Human Genetics, University of Leuven, Leuven, 3000 Belgium; 21 AI lab, Vrije Universiteit Brussel, Brussels, 1050 Belgium; 22 Pediatrics, Hôpital Universitaire des Enfants Reine Fabiola, Université Libre de Bruxelles, Brussels, 1020 Belgium; 23 Genetics, Hôpital Universitaire des Enfants Reine Fabiola, Université Libre de Bruxelles, Brussels, 1020 Belgium; 24 Present address: Neuropediatrics, Clinique Saint-Anne Saint-Rémy - CHIREC, Brussels, 1070 Belgium; 25 Present address: Assisted Fertilization Department, Casa di Cura Città di Udine, Udine, 33100 Italy

**Keywords:** Functional genomics, Promoters, Neurodevelopmental disorders, Intellectual disability, *DLG2*

## Abstract

**Background:**

Tissue-specific integrative omics has the potential to reveal new genic elements important for developmental disorders.

**Methods:**

Two pediatric patients with global developmental delay and intellectual disability phenotype underwent array-CGH genetic testing, both showing a partial deletion of the *DLG2* gene. From independent human and murine omics datasets, we combined copy number variations, histone modifications, developmental tissue-specific regulation, and protein data to explore the molecular mechanism at play.

**Results:**

Integrating genomics, transcriptomics, and epigenomics data, we describe two novel *DLG2* promoters and coding first exons expressed in human fetal brain. Their murine conservation and protein-level evidence allowed us to produce new *DLG2* gene models for human and mouse. These new genic elements are deleted in 90% of 29 patients (public and in-house) showing partial deletion of the *DLG2* gene. The patients’ clinical characteristics expand the neurodevelopmental phenotypic spectrum linked to *DLG2* gene disruption to cognitive and behavioral categories.

**Conclusions:**

While protein-coding genes are regarded as well known, our work shows that integration of multiple omics datasets can unveil novel coding elements. From a clinical perspective, our work demonstrates that two new *DLG2* promoters and exons are crucial for the neurodevelopmental phenotypes associated with this gene. In addition, our work brings evidence for the lack of cross-annotation in human versus mouse reference genomes and nucleotide versus protein databases.

**Electronic supplementary material:**

The online version of this article (doi:10.1186/s13073-017-0452-y) contains supplementary material, which is available to authorized users.

## Background

Neurodevelopmental disorders (NDDs) are impairments of the growth, development, and function of the brain. They show vast genetic heterogeneity, pleiotropy, monogenic to polygenic origin, and age-related phenotypic variability [1–6]. Considering the main phenotype, age at presentation, and pathophysiology, one can group NDDs into discrete clinical categories [1, 2]—for example, cognitive (e.g., global developmental delay (GDD)/intellectual disability (ID), language disorders), behavioral (e.g., autism spectrum disorders (ASDs), attention deficit hyperactivity disorders (ADHDs)), psychiatric (e.g., schizophrenia, bipolar disorders), and epileptic (early infantile epileptic encephalopathies, generalized seizures). Patients can present with phenotypes of more than one category and phenotypic presentation can vary inside families, highlighting the importance of genetic background, modifier genes, and environment [5–7].

ID is a frequent and often severe pediatric condition. The prevalence of ID is estimated to be between 1 and 3% and lifetime costs of treatment and support average more than $1 million per person [8]. The formal diagnosis of ID requires cognitive testing, which is inaccurate below 5 years of age. Therefore, in this age category, the term global developmental delay (GDD) is considered more appropriate [8]. The development of higher resolution genetic screening methods has underlined the prevalence of genetic anomalies, such as copy number variations (CNVs), in children with ID [9]. Many of these CNVs occur de novo, but some can be inherited from an asymptomatic parent and nevertheless be clinically significant, increasing the difficulty for genetic counseling [10, 11]. Furthermore, the precise pathophysiological role of the majority of structural aberrations remains unknown [12–15]. Among the causes, one could be the presence of yet uncharacterized functional genomic regions.

The identification of all protein-coding transcripts encoded in the human genome is still an open problem [16]. As several studies have pointed out, the expression of novel transcripts and splicing sites is highly tissue-specific [16–18]. De novo transcriptome assembly in various fetal and adult human tissues identified thousands of novel transcripts, coding regions, genes, and splicing sites [19, 20]. Alongside this, the search for first exons and their upstream promoters gave birth to several promoter predictor programs [21]. Some of them integrate DNA sequence information with H3K4me3 histone modification and cap analysis gene expression (CAGE) data [22–26].

Genetic research in mouse has also improved the understanding of human gene functions, annotation of the human genome, and genotype–phenotype mapping of human diseases [27, 28]. Mice and humans share about 99% of their genes and many of the Mendelian/polygenic disorders [27, 29]. Furthermore, the synteny property of genes in these organisms enables their cross-identification [17, 27]. For these reasons, mice are often used as a model organism to study candidate functional regions in humans [30].

The *DLG1*, *DLG2*, *DLG3*, and *DLG4* gene products (also called SAP97, PSD-93, SAP102, PSD-95 in mouse) are proteins belonging to the membrane-associated guanylate kinase (MAGUK) superfamily [31]. They are located in the postsynaptic density (PSD) of glutamatergic excitatory brain synapses with specific distribution according to brain subregions, type of synapses, synapse maturation, and age [31–33]. They contain different domains (e.g., PDZ, GK, SH3), allowing them to bind to multiple proteins present at the synapse [31]. As scaffolding proteins binding to both cytoskeleton proteins and signaling complexes, they play an important role in the development, plasticity, and stability of synapses [31–43]. Mice and humans share conserved functional roles of *DLG2* in complex cognitive and learning tasks [44].

A multi-omics integration approach can discover the link between genotypes and phenotypes, especially in the presence of complex pathologies [45]. In this study, our in silico multi-omics integration analysis of several independent functional datasets contributed to the identification of two novel promoters and coding first exons in the *DLG2* gene. These novel isoforms are expressed in the fetal brain and have protein coding murine equivalents. Deletions of these new elements were found statistically associated with NDDs by comparing multiple independent case and control cohorts. So far, human CNV deletions in *DLG2* have been linked to psychiatric disorders [44, 46–49]. Our study now pinpoints *DLG2*’s association with neurodevelopmental disorders in general, and GDD/ID in particular.

## Methods

### Case reports

Here, we present two unrelated cases of young male children from Hôpital Universitaire Des Enfants Reine Fabiola (HUDERF; Université Libre de Bruxelles (ULB)) showing developmental delay and bearing a partial deletion of the *DLG2* gene as a single CNV: they both have introns 6 and 7 and exon 7 deleted; patient 1 has also lost exon 8. Both variants have been inherited from asymptomatic mothers.

#### Patient 1 (DECIPHER: 317136)

Patient 1 is the third child born to healthy unrelated adults and has two older unaffected sisters. He was born after an uneventful pregnancy and normal delivery. Growth parameters were within normal limits for height, weight, and head circumference. Motor delay was evident early on. He was able to sit unsupported at 17 months and started walking at around 24 months. Around 18 months of age, the parents reported an episode of mental absence without complete loss of consciousness that lasted around 15 minutes; there has been no recurrence. Language delay was also clearly evident; the child began forming comprehensible words not long before his third birthday. The child was referred to a pediatric neurologist by the resident school psychologist upon entry into the first year of preschool (at 3 years of age) as he had noticed major difficulties in climbing and descending stairs, as well as a hesitant and unsure gait, and a general slowness in executive function. Socially, the child was excessively shy and did not interact with others or participate in class activities; sometimes he spent almost the entire school day crying. The teachers noted that, on occasion, he repeated the same simple gesture over and over, at times for as long as 15 min.

At this time the physical examination was unremarkable. There were no facial dysmorphism nor skin pigmentation anomalies. The neurological examination was difficult as the child was very timid and refused to leave his mother, but there seemed to be no apparent deficit. He smiled often and eye contact was good. He was capable of pointing to and identifying various parts of the face. The parents reported that at home he likes to pretend to be cooking and that he often plays with the dolls of his sisters. Eating and sleeping habits were normal.

**Fig. 1:**
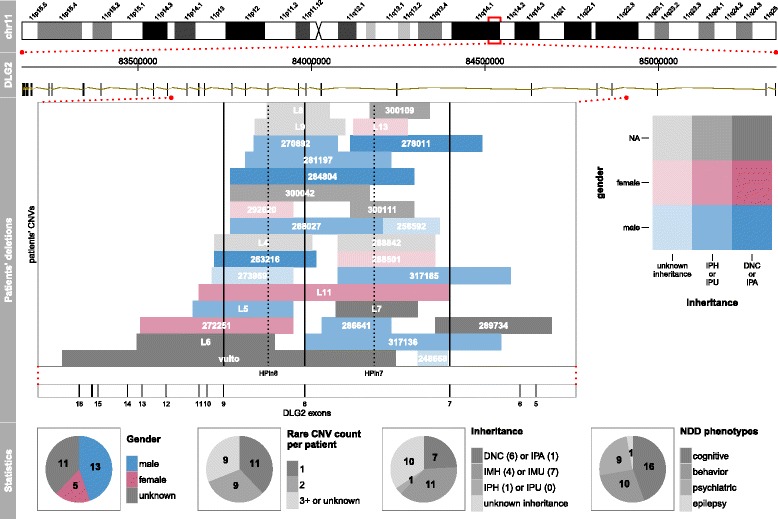
Patient overview for the *DLG2*. Four tracks are shown representing the data at different granularity levels. The first track, “*chr11*”, shows chromosome 11 with its cytobands; the *rectangular red box* indicates the *DLG2* location. The second track, “*DLG2*”, shows genomic coordinates on the *top* and all unified exons (Additional file 1: Table S1 and Figure S1) at the *bottom*. The third track, “*Patients’ deletions*”, shows CNV locations for the 29 patients from DECIPHER, ULB, and the literature; each *box* represents a deletion carrying three kinds of patient information: i) id, ii) gender, and iii) inheritance type (detailed in Additional file 1: Tables S7–S11). *Vertical solid black lines* represent exons, while *dotted lines* highlight histone peaks (HPs) discovered using Roadmap Epigenomics data integration and described in the present work. The fourth track, “*Statistics*”, summarizes some basic statistics about the patients’ CNVs and clinical characteristics. In the “*Inheritance*” pie chart, we used the following abbreviations: *DNC* de novo constitutive, *IMH* inherited from a healthy mother, *IMU* inherited from a mother with unknown phenotype, *IPH* inherited from a healthy parent, *IPU* inherited from a parent with unknown phenotype, *IPA* inherited from a parent affected by the same phenotype. *NDD* neurodevelopmental disorder. All genomics coordinates are in hg19

A complete workup for developmental delay including a head MRI, overnight EEG monitoring, and CNS evoked potentials showed normal results. Genetic testing was performed through CGH-array 180 K ISCA (see Additional file 1: Supplementary note 8 for a description of the array-CGH method used). It revealed a heterozygous 523-kbp deletion inside cytogenetic band 11q14.1 producing a deletion of the coding exons 7 and 8 of *DLG2* (Fig. 1, patient 317136). Further testing of the parents revealed the same deletion in the healthy mother, who did not report any related problems during childhood nor later. Siblings were not tested according to recommendations concerning genetic tests in asymptomatic children.

The child was hence diagnosed with psychomotor developmental delay of undetermined origin. Physical and speech therapy were continued and relational psychomotor therapy was added to the treatment. At 4 years of age, the parents reported a clear improvement in verbal and non-verbal communication. At school, the child was more willing to participate in activities and play with other children. He was calmer and less prone to crying. His vocabulary reached over 100 words and he was able to make simple sentences such as “Is where mom?” He was less afraid to speak with people other than his parents, but he was still very apprehensive in regards to unknown people or situations. At 4.5 years of age, the parents reported further progress. Verbally, the child could make full sentences but used “me” instead of “I” as a subject. He also had difficulty conjugating verbs. He could count to three but was not yet able to recognize colors. In view of the persisting difficulties in preschool, an eventual placement in a special education program was discussed with the parents. On cognitive evaluation at the age of 6 years by verbal and performance Wechsler Preschool and Primary Scale of Intelligence (WPPSI-R), his full scale IQ was 65 (patient details reported in Additional file 1: Table S11).

#### Patient 2 (DECIPHER: 317185)

Patient 2 is the first and only child born to healthy unrelated adults. Pregnancy and delivery were uneventful. Microcephaly was noted at birth (head circumference of 32 cm, 2.3 standard deviations below the mean) and the birth weight was 2990 g. The Apgar score was 7-8-10. Height and weight growth rates were normal, with progressive microcephaly. The medical history included recurrent otitis media (requiring bilateral tympanic tube placement), slight bilateral hyperopia (diagnosed at age 2), and surgical excision of right pre-auricular tag. Motor milestones were marginally delayed (seating position, 8 months; walking, 18 months), but there was a clear delay in language and social skills. The child exhibited poor visual contact, lack of facial expression, and minimal social exploratory behavior.

The child was first referred to a pediatric neurologist with concerns regarding global developmental delay at the age of 17 months. At this time, physical examination showed microcephaly (head circumference of 43 cm, 3.6 standard deviations below the mean) and a low-set right ear with an underfolded helix. There were no other facial dysmorphism and skin pigmentation anomalies. Neurological evaluation revealed a generalized mild hypotonia with no other abnormal findings.

A complete workup for developmental delay including head MRI, overnight EEG monitoring, and CNS evoked potentials did not show any specific findings. CGH-array 180 K ISCA (see Additional file 1: Supplementary note 8 for a description of the array-CGH method used) revealed a heterozygous 463-kbp deletion inside cytogenetic band 11q14.1, producing a deletion of coding exon 7 of *DLG2* (Fig. 1, patient 317185). Further testing of the parents revealed the same deletion in the healthy mother, who did not report any related problems during childhood nor later.

From the age of 3 onwards, he was sent to a school for special educational needs. At the age of 5, he presented a clear global developmental delay, without autistic features. Verbal comprehension was poor and expression was limited to a few words with many phonological difficulties. At school, he had learned to use augmentative and alternative communication devices. Reasoning and visuospatial skills were limited, with a poor attention span. Gross motor function was in the normal range but he still had difficulties with fine motor skills. He could count to three and began to recognize colors. On cognitive evaluation at the age of 6 by WPPSI-R, his full scale IQ was 62 (patient details reported in Additional file 1: Table S11).

Standard karyotypes as well as *FMR1* repeat amplification analysis were normal in both patients.

### Datasets

#### Patient and control CNV datasets

We first considered two public CNV datasets: i) the Database of Chromosomal Imbalance and Phenotype in Humans Using Ensembl Resources (DECIPHER) [15], a collection of thousands of patients with rare copy number variations and phenotypes; and ii) the Database of Genomic Variants (DGV) [50], a collection of structural variations identified in healthy control samples. We used release version dated January 1st, 2015 for DECIPHER and October 16th, 2014 for DGV. As validation, we used two more datasets: the Coe et al. [11] and Cooper et al. [51] “Developmental Delay” case and control cohorts recently available through the UCSC genome server and public repositories, and the database of structural variants discovered in the 1000 Genomes Project [52]. These are hereafter named GDD/ID (cases or controls, as specified) and 1KG datasets, respectively.

*DLG2* has been known to be linked to psychiatric disorders [44, 46–49]; therefore, we collected from the literature those patients whose clinical description and CNV were publicly available (Table 1). Genomic locations in hg18 were converted to hg19 using the UCSC liftOver tool.

**Table 1:** Description of the 29 patients having deletions in the DLG2 7-9 region

Patient ID	Type of CNV	*DLG2* CNV hg19 coordinates	CNV size (Mb)	*DLG2* deleted features	Gender	Other rare CNVs	Inheritance of *DLG2* variant	Neurodevelopmental phenotype
Patient 1 317136	Del	Chr11:84245639-84772741	0.52	Exons 7–8; HPin7	Male	0	Inherited from an unaffected mother	C, B
Patient 2 317185	Del	Chr11:84334015-84797219	0.46	Exon 7; HPin7	Male	0	Inherited from an unaffected mother	C, B
DECIPHER 248668	Del	Chr11:84548697-84628963	0.08	Intron 7	Male	32	Unknown	C
DECIPHER 256592	Del	Chr11:84456097-84607440	0.15	Intron 7	Male	1^a^	Unknown	C, B
DECIPHER 263216	Del	Chr11:84003279-84276072	0.27	Exons 8–9; HPin8	Male	0	Inherited from a parent with same phenotype	Unknown
DECIPHER 270892	Del	Chr11:84108622-84334253	0.23	Exon 8; HPin8	Male	0	Inherited from normal parent	C, B
DECIPHER 272251	Del	Chr11:83805117-84215024	0.41	Exons 9–13; HPin8	Female	3^b^	Inherited from an unaffected mother	C, B, P
DECIPHER 273969	Del	Chr11:83996254-84214903	0.22	Exon 9; HPin8	Male	0	Unknown	Unknown
DECIPHER 278011	Del	Chr11:84367238-84721340	0.35	Exon 7; HPin7	Male	1^c^	De novo constitutive	C
DECIPHER 281197	Del	Chr11:84085773-84477088	0.39	Exon 8; HPin7-8	Male	0	Inherited from an unaffected mother	C, B
DECIPHER 284804	Del	Chr11:84046644-84539636	0.49	Exon 8; HPin7-8	Male	0	De novo constitutive	C, B
DECIPHER 286641	Del	Chr11:84291759-84477088	0.19	Intron 7; HPin7	Male	1^d^	Inherited from an unaffected mother	Unknown
DECIPHER 288027	Del	Chr11:84046530-84454687	0.41	Exon 8; HPin7-8	Male	1^e^	Inherited from an unaffected mother	C, B
DECIPHER 288501	Del	Chr11:84334017-84595634	0.26	Intron 7; HPin7	Female	1^f^	Unknown	Other
DECIPHER 288842	Del	Chr11:84334017-84595634	0.26	Intron 7; HPin7	Unknown	1^g^	Unknown	Other
DECIPHER 289734	Del	Chr11:84595575-84907579	0.31	Exons 5–7	Unknown	2^h^	De novo constitutive	C, B
DECIPHER 292620	Del	Chr11:84046614-84214762	0.17	Intron 8; HPin8	Female	0	Unknown	C
DECIPHER 300042	Del	Chr11:84046614-84419502	0.37	Exon 8; HPin8	Unknown	0	Unknown	C
DECIPHER 300109	Del	Chr11:84419443-84581292	0.16	Intron 7; HPin7	Unknown	0	Inherited from a mother of unknown phenotype	C
DECIPHER 300111	Del	Chr11:84367238-84539665	0.17	Intron 7; HPin7	Unknown	0	Inherited from a mother of unknown phenotype	C
1339 [10]	Del	Chr11:83595987-84489649	0.89	Exons 8–18; HPin7-8	Unknown	1^i^	De novo constitutive	C, B, E
L4 [48]	Del	Chr11:84003321-84266329	0.26	Exons 8–9; HPin8	Unknown	Unknown	Unknown	P
L5 [46]	Del	Chr11:83945764-84214964	0.27	Exons 9–11; HPin8	Male	Unknown	Inherited from an unaffected mother	P
L6 [47]	Del	Chr11:83795102-84165325	0.37	Exons 9–13; HPin8	Unknown	Unknown	De novo constitutive	P
L7 [47]	Del	Chr11:84328458-84548416	0.22	Intron 7; HPin7	Unknown	Unknown	De novo constitutive	P
L8 [49]	Del	Chr11:84143697-84312722	0.17	Exon 8; HPin8	Unknown	1^j^	Unknown	P
L9 [49]	Del	Chr11:84111384-84354568	0.24	Exon 8; HPin8	Unknown	1^k^	Unknown	P
L11 [44]	Del	Chr11:83961633-84633847	0.67	Exons 8–11; HPin7-8	Female	Unknown	Inherited from an unaffected mother	P
L13 [44]	Del	Chr11:84375859-84521180	0.145	Intron 7; HPin7	Male	Unknown	Unknown	P

*Neurodevelopmental phenotype abbreviations*: *C* cognitive, *B* behavioral, *P* psychiatric, *E* epilepsy; we used patients’ DECIPHER id whenever available; this is the case for the two ULB patients and the 18 public DECIPHER patients. The 21st entry is identified by the reported id number in Vulto-van Silfhout et al. [10]. We provide inside square brackets references for the other eight patients found in the literature (L4-9, L11, L13). See Additional file 1: Tables S7–S11 for a more detailed description. HPin7 and HPin8 are names given to the new *DLG2* functional regions described in this work (see text)

^a^30-kbp dup on chrY. Genes: *AKAP17A*, *ASMT*

^b^225-kbp dup and 278-kbp dup on chr9, 148-kbp dup on chr11. Genes: *TRPM3*, *TMEM2*, *EHF*

^c^300-kbp del on chr17 variant inherited from normal parent. Genes: *SLC39A11*

^d^290-kbp dup on chr17 inherited from mother. 22 genes

^e^130-kbp dup on chr7 inherited from mother. Gene: *AKAP9*

^f^180-kbp del on chr1. Genes: *INPP5B*, *MTF1*, *SF3A3*

^g^541-kbp del on chr16. 28 genes

^h^191-kbp del on chr1 and 101-kbp del on chr12; both inherited from mother. Genes: *SUMF1*, *PIK3C2G*, *RERGL*

^i^5-Mbp dup on chr15; de novo. Several genes

^j^41-kbp del on chr1. Gene: *DNAJC6*

^k^23-kbp dup on chr20. No gene

Since those datasets come from distinct studies, different protocols might have been used to report CNVs. Hence, we decided to apply a common set of filtering rules on all datasets as a preprocessing step. We considered CNVs having a length between 50 bp and 3 Mbp (as imposed by DGV) with an absolute log2ratio value ≥0.32 (as routinely done at Université Libre de Bruxelles Center for Medical Genetics). Furthermore, DGV and literature structural variations are described categorically as either duplication or deletion; therefore, for each dataset, we converted log2ratios into categorical values using the following rationale: duplication for positive value, deletion for negative value. For the whole genome enrichment analysis, we used the release version dated April 22nd, 2016 for DECIPHER and July 2015 for DGV (see Additional file 1: Supplementary note 2 for details).

#### Roadmap Epigenomics Project

From the Roadmap Epigenomics Project, we used genome-wide profiling of six histone modification markers (H3K4me3, H3K4me1, H3K27ac, H3K9ac, H3K27me3, H3K9me3) in 13 cell lines and tissues: H1 cell line, H1 derived neuronal progenitor cultured cells (H1 NPC), H1 derived mesenchymal stem cells (H1 mesenchymal), H1 BMP4 derived mesendoderm cultured cells (H1 mesendoderm), fetal brain, and eight adult brain tissues (Additional file 1: Figures S19 and S20). We compared ChIP-Seq signal to a corresponding whole-cell extract sequenced control to identify narrow regions of enrichment (peaks) using MACS v2.1.0 peak caller with default parameters. Since the Roadmap Epigenomics dataset provides at least one ChIP-Seq control, we identified peaks for every possible profile–control combination in the tissue. In this work, we used Roadmap Epigenomics data version 9.

Following the general consensus regarding the histone code [53, 54], we used H3K4me3 as a marker of promoter potential and we grouped H3K9ac, H3K27ac, and H3K4me1 markers as genic activators and H3K27me3 and H3K9me3 markers as genic repressors.

#### Chromatin modification datasets (ENCODE Project)

We used histone modification and transcription factor binding datasets from the human and mouse ENCODE projects via the UCSC genome browser.

#### RNA-Seq data and pipeline (ENCODE Project)

The ENCODE Project provides a collection of genomics data available for the analysis of functional elements [55]. Starting from RNA-Seq data, we ran a pipeline to understand the transcriptional role of the two new functional elements described in this work (see “Results”). The pipeline consists of two main steps: de novo transcriptome assembly and gene/isoform detection. We investigated fetal brain RNA-Seq paired-end data (hg19) and collected BAM alignment files for six experiments (ENCODE ENCSR000AEW, ENCSR000AFD, ENCSR000AFE, ENCSR000AEX, ENCSR000AEY, ENCSR000AFJ; see Additional file 1: Table S19). Running *cufflinks* v2.2.1 [56] in de novo configuration (without -g or -G options) with *--no-update-check* and *--library-type fr-firststrand* parameters resulted in a GTF file per each BAM given as input. The assemblies were then merged into a master transcriptome via the *cuffmerge* tool. We then computed the detection and visualization of exons and splice junctions using QoRTs v1.0.1 [57] (*java -Xmx4G -jar < QORTS_JARFILE > QC --stranded* command) and JunctionSeq v0.6.9 [58] software (*runJunctionSeqAnalyses* function with *analysis.type = exonsOnly* and *method.GLM = advanced* parameters). We ran the same pipeline on fetal and adult non-brain tissues such as liver (ENCODE ENCSR000AFB), muscle (ENCODE ENCSR000AFF and ENCSR000CUI), skin (ENCODE ENCSR000AFG), thyroid (ENCODE ENCSR000AFK), and fibroblast of dermis (ENCODE ENCSR000CUH). Because adult brain paired-end RNA-Seq data were not available through ENCODE, we used paired-end RNA-Seq data from frontal, temporal, occipital, and cerebellum adult brain tissues from Yao et al. [59].

We also retrieved mouse newborn brain RNA-Seq data from ENCODE: hindbrain (ENCODE ENCSR749BAG), midbrain (ENCODE ENCSR255SDF), and forebrain (ENCODE ENCSR723SZV). From these three tissues, we collect six BAM files (see Additional file 1: Table S20 for details).

In our genome-wide analysis, we estimated the presence of a splicing site by means of differential coverage between adjacent nucleotides. We used *bedtools* [60] v2.25.0 (subcommand *genomecov* with parameters *–bg –split*) to measure coverage per each nucleotide, resulting in a bedgraph file for each fetal brain BAM file.

#### CAGE peaks and FANTOM5 project

Cap analysis gene expression (CAGE) is a method to determine transcription start sites on a genome-wide scale. We investigated the CAGE signal using FANTOM5 project [61]. We retrieved, from the project repository and UCSC (access date: 19 October 2016), robust CAGE peaks identified by decomposition-based peak identification (DPI) [62]. Robust CAGE peak data include position (start, end), strand, tissue-specific expression level (in tags per million), and tissue type. For each robust CAGE peak in H3K4me3 peak regions (HP, see “Results”), we compared the expression level in brain versus other tissues. The list of brain tissues for the former group is reported in Additional file 1: Table S18.

#### Conservation analyses

The conservation analysis of human genomic sequences or regions was performed in mouse via the NCBI BLAST online tool and across vertebrates by means of the CEGA database [63].

### Genomic coordinates, exons, and isoforms

#### DLG2/Dlg2 genome references

From UCSC we gathered and preprocessed data related to *DLG2* with the following objectives: i) gather all exons belonging to all isoforms and number them uniquely; ii) understand which exons are shared across isoforms and which serve as alternative promoters. The result of this preprocessing step is reported in Fig. 1 (details in Additional file 1: Table S1 and Figure S1). In this paper we use UCSC numerical reference to identify an exon; we also provide the Ensembl *DLG2* coordinates and isoforms (Additional file 1: Table S2 and Figure S2) and comparison of both annotations (Additional file 1: Table S3). For the whole genome analysis, we refer to the coordinates available in the UCSC browser *knownGene* table (hg19) regarding genes and exons.

For mouse, we list the coordinates of the UCSC known *Dlg2* exons in Additional file 1: Figure S3 and Table S4; we also report the Ensembl *Dlg2* exonic coordinates for two mouse strains (BALB/cJ and A/J) in Additional file 1: Figures S4 and S5 and Tables S5 and S6.

#### DLG2/Dlg2 exons and amino acid mapping

We used NCBI BLAST (access date: November 2016) and UniProtKB/Swiss-Prot (access date November 2016) databases to align DNA to amino acid sequences and to map orthologous exons between human and mouse *DLG2-Dlg2* genes. The mapping between *DLG2/Dlg2* exons is reported in Additional file 1: Supplementary notes 3–6. We describe here the information regarding the unmapped *DLG2/Dlg2* exons. In Parker [32], the expression at the RNA level of the six mouse DLG2 (also known as PSD-93) protein isoforms described by the authors were detected by reverse transcription RT-PCR using isoform-specific primers. We used the reported forward primers of PSD-93 zeta and PSD-93 gamma in NCBI BLAST to locate the start of their coding regions in mm10 genomic coordinates. For the former, the primer aligns to chr7:90504814-90504835, around 600-kbp upstream of the first UCSC mouse exon, possibly corresponding to the coding region of human exon 3; for the latter, the primer aligns between mouse exons 3 and 4, chr7:91711767-91711790, orthologously mapping to human exon 10 (Additional file 1: Supplementary note 6). Likewise, RT-PCR primers for PSD-93 beta and epsilon map to the two new exons described in this work (see “Results”; Additional file 1: Supplementary Note 6). We then compared the murine protein sequences reported in UniProtKB/Swiss-Prot to the human genome (see Additional file 1: Supplementary notes 3 and 4 for details). The beginning of the mouse Q91XM9-7 (also known as PSD-93 zeta) isoform, from position 38 to position 156, aligns with the start of the human Q15700-2 isoform (encoded by human exons 3 to 6) with E-value of 1.1 × 10^−58^ (NCBI BLAST score). The first seven amino acids (MQHAFIP) of the mouse Q91XM9-3 (also known as PSD-93 gamma) isoform match with the end of human exon 10. No known mm9 or mm10 UCSC or Ensembl *Dlg2* exons code for these murine zeta and gamma protein isoforms.

### Statistical analyses

All statistical analyses were performed using R software, version 3.2. Regarding the whole genome analysis, while it was possible to merge DECIPHER NDD patients with GDD/ID cases, we had to deal with missing patient information in 90.9% of the total GDD/ID CNV controls [11, 51]. Merging DGV and GDD/ID control cohorts would filter out most of the data in the latter (Additional file 1, Supplementary notes 1 and 2). For this reason, we classify as statistically enriched for NDD cases those regions having a *p* value <0.05 after Bonferroni correction in both the following settings: DECIPHER + GDD/ID cases versus DGV and DECIPHER + GDD/ID cases versus GDD/ID controls.

## Results

### Identification of novel *DLG2* genomic elements (HPs)

In the ULB cohort, DECIPHER, and the literature we found 29 patients with a monogenic deletion involving the *DLG2* gene (Table 1; see “Methods”). To our knowledge, we are the first to analyze these patients as a cohort. Except for five patients (three with unknown phenotypes), they all present neurodevelopmental symptoms (see the “Clinical description of the 29 *DLG2* patients cohort” section below). All deletions alter the region between exons 7 and 9 (7-9 region; Fig. 1). In our effort to explain why intragenic *DLG2* deletions occur exclusively in the 7-9 region, we noticed that 10/29 (34%) patients have only intronic aberrations, three of which have that intronic deletion as a single variation reported in DECIPHER (DECIPHER 292620, 300109, 300111; Fig. 1), possibly suggesting pathogenic involvement of yet unknown functional elements in intron 7 or 8 of *DLG2*.

**Fig. 2:**
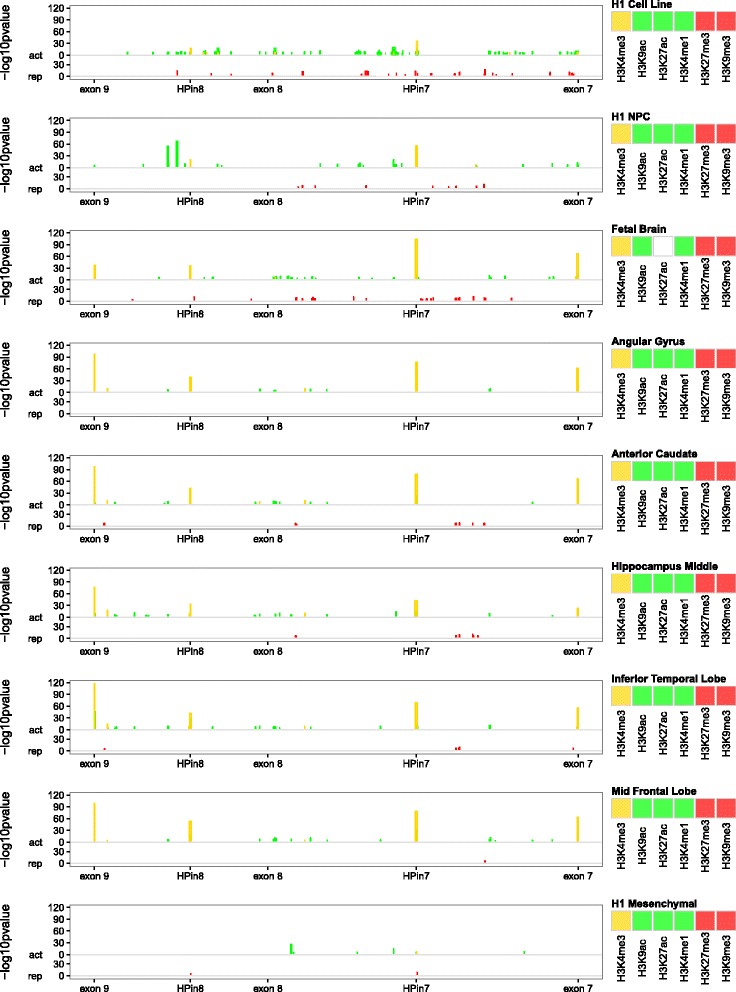
Discretized ChIP-Seq profile overview of different markers across different tissues or cell types. The data come from profile–control comparisons of Roadmap Epigenomics Project data using MACS v2.1.0. The *y-axis* reports the *-log10p value* as measurements of marker against control enrichments; the greater the height, the higher the statistical confidence. Each of the nine stacked plots reports a discretized ChIP-Seq profile for different markers in one specific tissue. Starting from the *top* we have stem cells (H1 cell line), neuronal progenitor cells (H1 derived neuronal progenitor cultured cells), fetal brain, adult brain tissues, and, at the *bottom*, one non-brain-related tissue, H1 derived mesenchymal stem cells. In each plot, histone modifications are grouped according to their related function: promoter marker (in *gold*), activation markers (“*act*”, in *green*) or repression markers (“*rep*”, in *red*). The same y scale is applied to the three groups. Exons 7, 8, and 9 along with HPin7 and HPin8 are reported on the *x-axis*. All markers are listed in the legend, mixed and overlapped in the plot. A *white box* in the marker legend means data are not available. All genomic coordinates are in hg19

We investigated the presence of unknown regulatory elements in the *DLG2* 7-9 introns by integrating the Roadmap Epigenomics Project data in a neuronal developmental scheme (Fig. 2). We compared histone modifications in available tissues and cell types related to brain and two other tissues as negative controls. We ordered these modifications along a developmental timeline: stem cells, neuronal progenitors, fetal brain, and adult brain. As depicted in Fig. 2 (see also Additional file 1: Figures S19 and S20 for all adult brain tissues), once a cell specializes as a brain cell, four regions enriched in H3K4me3 arise in the 7-9 region, suggesting the presence of four promoters. Two of these overlap exons 7 and 9, as we can expect from known *DLG2* isoforms (Additional file 1: Figure S1). The other two are in the middle of introns 7 and 8, positing them as novel functional elements. We called them H3K4me3 peaks of the *DLG2* 7-9 region (HPs): for convenience, HPin7 for intron 7 and HPin8 for intron 8 (see Table 2 for coordinates).

**Table 2:** Novel promoters and coding first exon coordinates

Human	hg18^a^	hg19	hg38^a^
HPin7	Chr11:84107722-84110266	Chr11:84430074-84432618	Chr11:84719031-84721575
HPin8	Chr11:83825494-83826799	Chr11:84147846-84149151	Chr11:84436803-84438108
CFEin7 coding region	Chr11:84108987-84109049	Chr11:84431339-84431401	Chr11:84720296-84720358
CFEin8 coding region	Chr11:83826079-83826156	Chr11:84148431-84148508	Chr11:84437388-84437465
Mouse	mm9^a^	mm10	
mHPin1	Chr7:98410804-98411661	Chr7:91262294-91263151	
mHPin2	Chr7:98691323-98692132	Chr7:91542813-91543622	
mCFEin1 coding region	Chr7:98411443-98411505	Chr7:91262933-91262995	
mCFEin2 coding region	Chr7:98691624-98691701	Chr7:91543114-91543191	

Genomic coordinates of the novel functional regions found the human *DLG2* and mouse *Dlg2* genes. We investigated their location in hg19 and mm10, respectively. Their corresponding location in other genome references were retrieved by means of the UCSC liftOver tool. In *DLG2*, we name HPin7 and HPin8 the H3K4me3 peak found in introns 7 and 8, respectively. Inside HPin7 and HPin8 we discovered a protein-coding exon. We identify the coding part of the human exons as CFEin7 (inside HPin7) and CFEin8 (inside HPin8). In mouse, we name the orthologous H3K4me3 peak regions mHPin1 and mHPin2, as they are located in the first and second introns of the *Dlg2* gene. We identify the coding part of the mouse exons as mCFEin1 (inside HPin1) and mCFEin2 (inside HPin2)

^a^Genomic locations retrieved using the UCSC liftOver tool

### Describing new promoters and coding first exons inside HPin7 and HPin8 in fetal brain

**Fig. 3:**
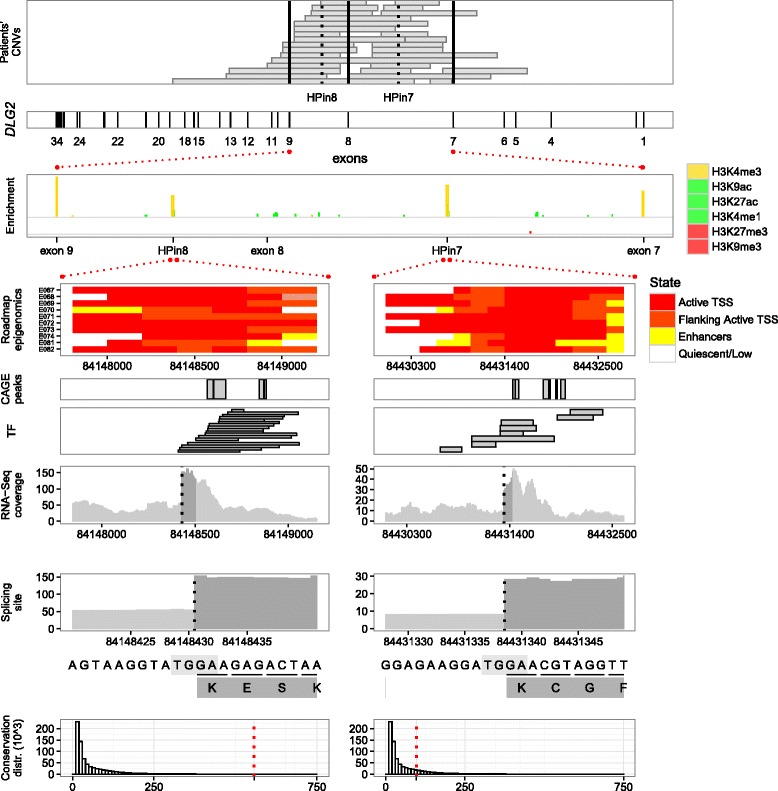
HPin7 and HPin8 data integration. The integration of genotypic, epigenomic, RNA-Seq, and complementary functional datasets for *DLG2* with a focus on HPin7 and HPin8, along with the 5′ splice site locations. Conservation is represented by the CEGA score [63]

We investigated the functional role of HPin7 and HPin8 by gathering further independent bioinformatics datasets (see “Methods”). Evidence of multiple robust CAGE peaks, human expressed sequence tags (ESTs), transcription factors (TFs), and high expression levels located in the HPs characterized them as having promoter activity (Fig. 3; Additional file 1: Figures S30–S34, S39, and S40). Likewise, ENCODE and Roadmap Epigenomics Project categorize HPin7 and HPin8 as active promoters in brain tissues and as weak/repressed/poised elements in other tissues (see the “Roadmap Epigenomics” panel in Fig. 3 and Additional file 1: Figures S16, S17, S21, and S22) [24, 64].

We studied in detail the HPin7 and HPin8 levels of expression in RNA-Seq data available from ENCODE (see “Methods”). The coverage in BAM files, along with CAGE and GTEx expression data [65], confirmed high *DLG2* transcriptional activity in brain tissues, specifically at the fetal stage (Additional file 1: Figures S23–S29, S32, S35–S40, and S45–S50). This suggests that the HPs could be the start of two new brain-specific *DLG2* isoforms. De novo transcriptome assembly with JunctionSeq, absence of reads splicing from upstream exons into the HPs, and absence of antisense reads strongly support such a hypothesis (Table 3; Additional file 1: Figures S39–S42 and S54 and Table S14).

**Table 3:** Summary of information collected and analyzed from different sources regarding HPin7 and HPin8 of *DLG2*

	HPin7	HPin8	Reference	Row
Human (hg19, *DLG2*)				
Location (UCSC)	Intron 7	Intron 8	Fig. 1	1
Number of del (*DLG2* cohort^a^)	15 (29)^b^	16 (29)^b^	Fig. 1	2
Number of del GDD/ID cases	7 (14)	4 (14)	Methods	3
Number of del GDD/ID control	4 (19)	1 (19)	Methods	4
Number of del DGV	0 (24)	0 (24)	Fig. S10^c^	5
Number of del called from 1KG	0 (15)	2 (15)	Fig. S11^c^	6
Roadmap Epigenomics prediction	Active promoter	Active promoter	Fig. S16, S17^c^	7
Roadmap Epigenomics highest peak	H3K4me3 in brain tissues	H3K4me3 in brain tissues	Fig. 2	8
ncRNA	lnc-TMEM126B-2:1	No	Results	9
FANTOM5 CAGE reads	Yes	Yes	Fig. S30, S31^c^	10
FANTOM5 CAGE expression	Brain	Brain	Fig. S32^c^	11
Number of human ESTs	2	2	Fig. S30, S31^c^	12
Ensembl predicted *DLG2* exon	No	Yes	Table S2^c^	13
ENCODE fetal brain RNA-Seq peaks	Yes	Yes	Fig. S39, S40^c^	14
JunctionSeq promoter and first exon de novo prediction	Yes	Yes	Fig. S54^c^	15
5′ splice site	AG.GT	AG.GT	Fig. S43, S44^c^	16
Coding exon	Yes	Yes	Results	17
Splicing into	Exon 8	Exon 11	Results	18
Recursive exon motif	No	No	Results	19
Mouse (mm9, *Dlg2*)				
Location	Intron 1	Intron 2	Fig S12, S13^c^	20
Epigenomics (from ENCODE)	H3K4me3 in cerebellum	H3K4me3 in cerebellum	Fig S12, S13^c^	21
Ensembl exon prediction	Yes	Yes	Fig S12, S13^c^	22
Coding exon	Yes	Yes	Results	23
Splicing into	Exon 2	Exon 4	Results	24

For each source, a reference is reported. For rows 2–6, values in parentheses stand for the number of deletions overlapping the *DLG2* 7-9 region. Row 3: the number of total GDD/ID cases corresponds to the number of intragenic deletions, i.e., those that are not affecting other genes; hence, nssv_3460188 and nssv_3461505 are not considered (Additional file 1: Figure S6). The “*5′ splice site*” entry reports the two nucleotides before and after the exon–intron border (marked with the *dot character*)

^a^The *DLG2* cohort is a collection of deletions from DECIPHER, ULB, and the literature overlapping the *DLG2* 7-9 region

^b^Five deletions overlap both HPin7 and HPin8

^c^In Additional file 1

We then looked for the presence of donor splicing sites to define the 3′ border of HPin7 and HPin8 exons. An abrupt difference of 46 and 59 in RNA-Seq read coverage locates them at positions chr11:84431338-9 and chr11:84148430-1, respectively (Additional file 1: Figures S43, S44, S55, S62, and S63). For both HPs, the four nucleotides at the splice site match the consensus AG.GT donor sequence [66, 67] (the “*Splicing site*” panel in Fig. 3).

**Fig. 4:**
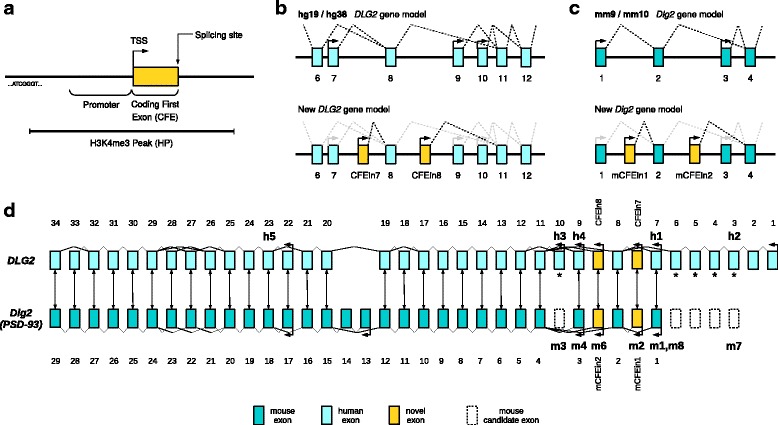
*DLG2* gene model and exon mapping in mouse and human. **a** Qualitative graphical representation of H3K4me3 peak (HP), upstream promoter, and coding first exon (CFE). *TSS* transcription start site. **b** Two *DLG2* gene models using UCSC data: the hg19/hg38 model at the *top* and the new model integrating our research regarding two novel promoters and coding first exons (CFEin7 and CFEin8) at the *bottom*. **c** mm9/mm10 and new *Dlg2* models comparison. **d** *DLG2*/*Dlg2* exon mapping by DNA sequence alignment. *Bidirectional arrows* between exons represent the best match according to NCBI BLAST. *Arrows* with tips pointing leftwards show transcription start sites according to UCSC, updated with our results. *Bold annotations*, such as “*h3*” and “*m8*”, mark the beginning of protein isoforms according to UniProtKB/Swiss-Prot, in human (Q15700) and mouse (Q91XM9). Regarding the mouse isoform terminology, “m*X*” stands for Q91XM9-*X* and m1, 2, 3, 4, 6, and 7 correspond, respectively, to PSD93-alpha, beta, gamma, delta, epsilon, and zeta. Human exons 3–6 and 10 are annotated with an *asterisk* because there is evidence (see “Methods” and Additional file 1: Supplementary note 10) that they map to unannotated *Dlg2* mouse exons in mm10 reference genomes. Those “missing” murine exons code for “m3” and “m7” protein isoforms, and are depicted with *dashed borders*. “m5” represents DLG2 isoform “Q91XM9-5” and maps from mouse exon 3 to exon 11 (Additional file 1: Supplementary note 4). It is not included in the figure because no experimental confirmation of its existence is available. In UniProt, “h5” is also reported as not experimentally proven. However, in GTEx (adult brain), it is the most expressed isoform (see ENST00000426717 in Additional file 1: Figure S26). Hence, it is included in the figure

The above analyses define two new *DLG2* exons. We named these novel human exons “coding first exons” (CFEs): CFEin7 inside HPin7, CFEin8 inside HPin8. We provide a schematic representation of HP, promoter, and CFE nomenclature in Fig. 4a. To find the exons which CFEin7 and CFEin8 splice into, we used the RNA-Seq reads that split over the donor site into the acceptor site. Those reads end in *DLG2* exon 8 and exon 11, respectively (Fig. 4b).

Collectively, our analyses strongly suggest that HPin7 and HPin8 promote two new bona fide *DLG2* isoforms (Table 3 and Fig. 4b). Several observations emerge from an evaluation of RNA-Seq profiles in human tissues. HP isoforms seem differentially expressed according to brain regions. CFEin7 and CFEin8 have peaks of expression at the fetal stage. The *DLG2* 7-9 region is quasi-devoid of transcription in non-brain or adult tissues. Nascent transcription is present in introns 6, 7, and 8 as classically found for long introns [68, 69]. We checked for the absence of the recursive exon motif YYYAGGURAG in CFEin7 and CFEin8 to rule out the presence of recursive splicing [69].

We summarize our bioinformatic analyses regarding HPin7 and HPin8 in Fig. 3 and Table 3.

### HPin7 and HPin8 nucleotide conservation

To characterize further these new isoforms, we checked for their existence in other species. Across vertebrates, elements corresponding to HPin7 and HPin8 are listed in the Conserved Elements from Genomics Alignments (CEGA) database [63], reporting moderate and high conservation scores, respectively (the “*Conservation distribution*” panel in Fig. 3; Table 3; Additional file 1: Figures S14 and S15). We then studied the murine conservation of HPin7 and HPin8 nucleotides by comparing their sequences with the mouse genome using NCBI BLAST. Both are highly conserved in the *Dlg2* gene, in the first and second introns. They overlap ChIP-Seq peaks of H3K4me3 in mouse brain tissues (ENCODE data visualized in Additional file 1: Figures S12 and S13), endorsing the same pattern seen in the human data. We called them mouse HPs (mHPs): mHPin1 in intron 1 and mHPin2 in intron 2 (see Table 2 for coordinates).

### Orthologous HP isoforms found in mouse

We inspected the transcriptional profile of mHPs using newborn mouse RNA-Seq data from ENCODE (see “Methods”). In the mHP regions, we observed transcriptional activity and the presence of splicing donor sites. RNA-Seq reads starting in mHPin1 and mHPin2 do splice into *Dlg2* exon 2 and exon 4, respectively. These features, added to the presence of H3K4me3 peaks and the absence of upstream reads splicing into the HP regions, strongly suggest, as in human, the existence of new *Dlg2* promoter and first exons inside mHPs (Fig. 4c; Additional file 1: Figures S51–S53). We named the new mouse exons mCFEs: mCFEin1 inside mHPin1, mCFEin2 inside mHPin2.

### Human and mouse HPs promote protein coding isoforms

We used NCBI and UniProt to investigate the coding potential of the exons featured in HPs. UniProt includes the manually annotated and reviewed protein isoform dataset UniProtKB/Swiss-Prot. Using BLASTX [70, 71], we found that human HP DNA sequences match with the beginning of some human *predicted* proteins found in NCBI BLAST databases (Additional file 1: Supplementary note 5). These predictions aligned to two known mouse *Dlg2* protein isoforms found in UniProtKB/Swiss-Prot: Q91XM9-2 (known as PSD-93 beta) and Q91XM9-6 (known as PSD-93 epsilon). The beginning of these mouse proteins correspond to the exons found in mHPin1 and mHPin2 (“Methods”; Additional file 1: Supplementary notes 3 and 4). Hence, proteic experimental evidence in mouse added to evolutionarily conserved gene structures, epigenetic regulation, brain expression, and amino acid sequences indicate coding potential in both genomes.

The genomic coordinates of the coding regions inside human CFEs and mouse mCFEs are presented in Table 2. The complete nucleotide and amino acid human sequences are available in Additional file 1: Figure S88 and S89. The nucleotides corresponding to the coding segments of CFEin7 and CFEin8 are pictured in Fig. 3 (see the dark grey in *RNA-Seq coverage* and *Splicing site* panels). The human coding sequences have been registered in GenBank under references KY368394 (CFEin8) and KY368395 (CFEin7). Likewise, mouse coding sequences have been registered in GenBank under references KY368396 (mCFEin2) and KY368397 (mCFEin1).

### New *DLG2* and *Dlg2* gene models

Remarkably, despite experimental evidence of the murine PSD-93 beta and epsilon proteins and their referencing in UniProtKB/Swiss-Prot, *both* human and mouse genome references lack their corresponding exon annotations. CFEs do correspond to the first exons of these beta and epsilon isoforms. Hence, the identification and detailed assessment of the CFEs presented in this work offer new *DLG2* and *Dlg2* gene models (Fig. 4d). The new *DLG2* gene model has seven promoters and coding first exons, with respect to the five of the UCSC standard model described in hg19 or hg38 (Fig. 4b, d). Likewise, the new *Dlg2* gene model has six promoters and coding first exons rather than the four previously described in mm9 or mm10 (Fig. 4c, d).

Human–mouse *DLG2–Dlg2* gene comparison provided additional unexpected results. Using human and mouse reference genomes, we were able to map orthologous exons from one organism to the other in most of the exons but not all (asterisks in Fig. 4d). Human *DLG2* exons 1 to 6 and 10 along with mouse *Dlg2* exons 13 and 14 resulted in being “species specific”. At first, we believed the unmapped exons were the results of gain of functionalities during vertebrate evolution, but our experience with the HP isoforms made us suspicious. A manual examination of the murine protein sequences described in Parker [32] shows that murine exons equivalent to human exons 3 to 6 and exon 10 (asterisks in Fig. 4d) *must* exist but are entirely missing from mm9 or mm10 reference genome annotations (see “Methods”; Additional file 1: Supplementary note 10).

Concerning the *DLG2* 7-9 region, our results show that it involves *five* (instead of three) coding exons, of which *four* (instead of two) start different DLG2 protein isoforms (Fig. 4b). In the next section, we describe the deletion of these five exons in relation to NDDs.

### HPin7 and HPin8 deletions are statistically enriched in NDD patients

A HP exon deletion-based analysis would explain the occurrence of NDD symptoms in 26 out of the 29 (90%) *DLG2* patients (Fig. 1). The remaining three have other rare CNVs elsewhere in the genome (Additional file 1: Tables S7–S10). The number of control patients with affected HPs in the DGV and 1KG cohorts is zero and two, respectively, further suggesting the importance of the HP exons (Additional file 1: Figures S10 and S11, Tables S12 and S13). We therefore assessed the enrichment of HP deletions in cases over controls via statistical analyses.

**Fig. 5:**
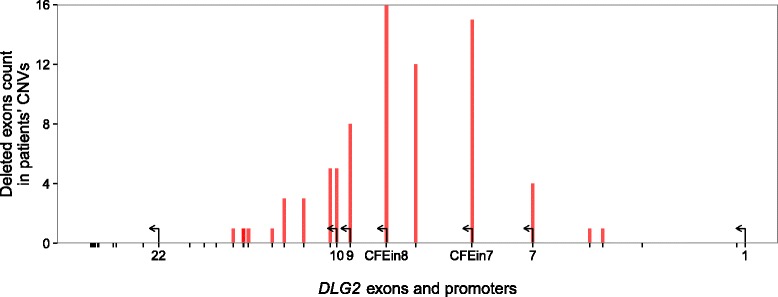
Number of times each exon is deleted in *DLG2* patients’ CNVs. Distribution of CNV deletions from patients in the *DLG2* cohort. *Red bars* represent the number of deletions overlapping the exon in consideration. On the *x-axis*, the exons that correspond to a transcription start site are reported with a number (shown by *arrows*)

One-tailed Fisher’s exact test comparing DECIPHER (case) and DGV (control) populations on the presence of deletions affecting any HPs resulted in a *p* value of 7.984 × 10^−07^. The same analysis using exons 7, 8, and 9 resulted in a *p* value of 6.107 × 10^−04^, pinpointing a stronger role of the two HPs, rather than the known *DLG2* exons, as links to NDDs (Figs. 1 and 5).

We validated the clinical importance of HP deletions by performing the equivalent statistical analysis with the independent GDD/ID cohorts [11, 51] (see “Methods”). In the case cohort, 11 deletions affect either HPin7 (7) or HPin8 (4) out of a total of 14 located in the *DLG2* 7-9 region. In the control cohort, three deletions affect either HPin7 (2) or HPin8 (1) out of a total of 19 located in the *DLG2* 7-9 region. The statistical analysis results in a *p* value of 4.501 × 10^−04^ for deletions affecting any HPs, and a *p* value of 0.3809 for deletions affecting exons 7, 8, and 9 (see “Methods”; Additional file 1: Figures S6–S9). This confirms the statistical enrichment of HP deletions in NDD cases, and the stronger role of HP exons with respect to known ones.

**Fig. 6:**
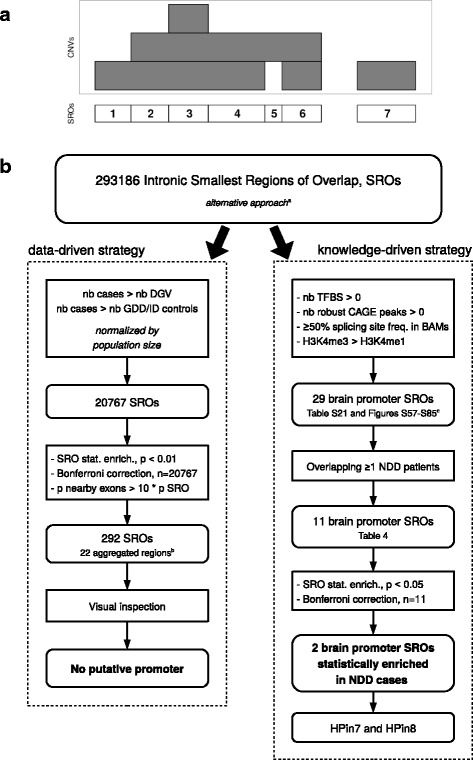
Genome analysis workflows used to discover novel promoters and first exons statistically associated with NDDs. **a** The smallest regions of overlap (SRO) definition (see also Additional file 1: Figure S56). **b** Summary of the whole genome analysis steps used to discover novel promoters and first exons statistically associated with NDDs. DECIPHER and GDD/ID cases are aggregated. The control cohorts are kept separate under the alternative approach (Additional file 1: Supplementary note 2). The four cohorts were used to define the SROs. ^a^Additional file 1: Supplementary note 2. ^b^One aggregated region corresponds to the set of one or multiple adjacent SROs. ^c^Additional file 1

To further validate this result, we performed a third statistical assessment using an unbiased genome-wide approach (Fig. 6; Additional file 1: Figures S86 and S87, and Supplementary notes 1 and 2 for methods and details). We used two methods: a data-driven strategy and a knowledge-driven strategy. The former, based on a straightforward patient versus control CNV enrichment analysis, turned out negative. The latter reduced the genome search space according to the presence of four functional characteristics known to be associated with promoters (Fig. 6). This strategy resulted in the prediction of 11 novel promoters and first exons found deleted in intronic regions in NDD patients (Table 4; Additional file 2). Two are statistically enriched in cases versus controls (*p* < 0.05, after Bonferroni correction). They correspond to HPin7 and HPin8. The knowledge-driven strategy validates, in a third way, the association between HP deletions and NDDs.

**Table 4:** Intronic regions harboring putative novel promoters found deleted in NDD patients

Entry	Chr	Start	End	Width	Gene name	Strand	Number of cases	Number of control	CEGA score	Splicing site	Type
a	Chr2	236577649	236583540	5892	*AGAP1*	+	2	1	31	236579701-2	P
b	Chr3	114167766	114174803	7038	*ZBTB20*	-	2	0	832	114173425-6	P
c	Chr5	14440397	14444098	3702	*TRIO*	+	1	1	192	14441469-70	P
d	Chr5	58722748	58727155	4408	*PDE4D*	-	1	4	300	58726119-20	N
e	Chr7	75266093	75269827	3735	*HIP1*	-	3	2	144	75268368-9	N
f	Chr11	84147024	84149361	2338	DLG2*	-	11	5	557	84148430-1	P^a^
g	Chr11	84429842	84432885	3044	DLG2*	-	13	2	97	84431338-9	N^b^
h	Chr11	84843131	84844944	1814	*DLG2*	-	3	0	862	84843811-2	P
i	Chr17	61227923	61231987	4065	*TANC2*	+	2	6	194	61228741-2	E
j	Chr22	28832791	28840308	7518	*TTC28*	-	2	1	553	28838873-4	P
k	Chr22	36355185	36358538	3354	*RBFOX2*	-	1	0	NA	36357610-1	P

Each row details an intronic H3K4me3 peak region overlapping any smallest region of overlap (SRO) meeting the following criteria: deleted in at least one case individual and demonstrating the presence of both transcription factor binding sites and CAGE peaks, of a H3K4me3/H3K4me1 peak ratio greater than 1, and of at least one abrupt RNA-Seq delta coverage of 20. We provide the name of the gene the intron belongs to, the number of case and control patients (sum of DGV and GDD/ID) found in the cohorts, and the exact location of the splicing site. The *asterisk* next to the gene name represents a significant statistical enrichment after Bonferroni correction of NDD patients in such region with *p* < 0.05 (see “Results”; Additional file 2). “CEGA score” documents the conservation score across vertebrates as reported in the Conserved Elements from Genomics Alignments database [63]. The “*Type*” column reports whether such a region is predicted as promoter (*P*) or exon (*E*) by Ensembl (archive 75, Feb 2014) or as novel (*N*). Coordinates are in hg19

^a^This region corresponds to HPin8

^b^This region corresponds to HPin7

**Fig. 7:**
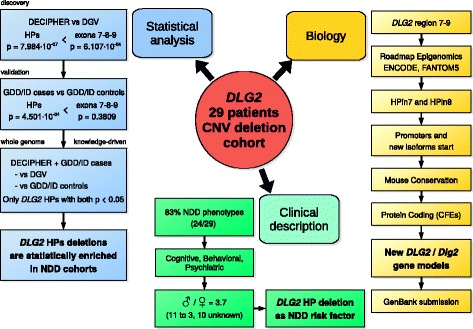
The main steps and results of the research. Summary of the high-level steps and main outcomes of the research described in this paper. *HPin7* H3K4me3 peak in *DLG2* intron 7, *HPin8* H3K4me3 peak in *DLG2* intron 8, *HP* either HPin7 or HPin8, *HPs* both HPin7 and HPin8, *CFE* coding first exon, *NDD* neurodevelopmental disorder

Collectively, our three statistical analyses show a consistent enrichment of HPin7 and HPin8 deletions in NDD patients (Fig. 7).

### Clinical description of the 29-patient *DLG2* cohort

Of the 29 patients we found with *DLG2* 7-9 deletions, 24 have a NDD phenotype (see Fig. 1 and Table 1 for an overview, and Additional file 1: Tables S7–S11 for a detailed description). The most common phenotypes are cognitive disabilities (mainly GDD/ID, 16 patients), followed by behavioral anomalies (ASD or ADHD, 10 patients) and psychiatric disorders (mainly schizophrenia, nine patients). The least-represented NDD phenotype is epilepsy with only one case. NDDs have been assessed for gender and heredity bias, classically describing a sex bias skewed towards boys with ID and maternally inherited CNV events [72–74]. Of the 24 NDD patients having aberrations in the *DLG2* gene, 11 are males and three females (ten unknown). Among them, the two ULB patients are males with aberrations inherited from asymptomatic mothers.

## Discussion

Although *DLG2* has been linked to psychiatric disorders [44, 46–49], our work now describes a cohort of patients with *DLG2* deletions who present three phenotypic categories of NDDs: cognitive, behavioral, and psychiatric. Such broad NDD involvement of *DLG2* is somewhat expected for a gene known to play an important role in the development, plasticity, and stability of synapses [3, 31–33, 43]. Concerning learning and memory, it is interesting to note that *Dlg2* (PSD-93) mouse knock-outs have defects in long-term potentiation of hippocampal neurons [75] and have been shown to be impaired in complex learning, cognitive flexibility, and attention [44]. Defects in cognitive flexibility can evoke ASD endophenotypes [44]. In the past, a hypothetical link was proposed between autism genes and *DLG2* through a PI3K synaptic pathway [76]. Also, while patients in the *DLG2* cohort have cognitive, behavioral, or psychiatric disorders, remarkably only one out of 29 has an epilepsy phenotype, suggesting this neurodevelopmental phenotype is less often associated with *DLG2* deletion. Epilepsy has scarcely been studied with reference to DLG proteins, and mostly with reference to DLG4 (PSD-95 in mouse) [77, 78]. *DLG1*, *DLG3*, or *DLG4* are not known as human epilepsy genes. Identically, *DLG2* has never been linked to epilepsy except for anecdotal reports [79–81].

The higher amount of male than female patients in the *DLG2* cohort (13 male to five female and 11 unknown out of 29; Table 1), with a penetrant NDD phenotype (11 to three and ten unknown out of 24), with a single rare CNV (five to one and three unknown out of nine), and harboring a maternally inherited aberration in *DLG2* (three to 0 and one unknown out of four) supports the NDD female protective hypothesis studied in other cohorts [72–74]. In addition to being more penetrant in males, the transmission of *DLG2* 7-9 deletions from clinically asymptomatic parents suggests incomplete penetrance. Such incompleteness is expected for inherited CNVs in NDDs [82, 83]. Two patients (GC33254, GC43330) having *DLG2* deletions and another relevant CNV cited in Sahoo et al. [84] also corroborate the additive burden “two-hit” hypothesis for risk factor CNVs in NDDs [82, 85]. Collectively, these data suggest that *DLG2* 7-9 deletions overlapping HPin7 and HPin8 are NDD risk factor CNVs. Larger case and control populations will help to determine their exact level of phenotypic penetrance. Note that very mild penetrance of any NDD risk factor CNV could be the norm [7, 13].

In the recent version of DECIPHER (December 2016) and the Signature Genomic Laboratories dataset [84], we found ten additional patients (Additional file 1: Supplementary note 9). Nine have a deletion in the *DLG2* 7-9 region and one is lacking exon 6 and entry “h” in Table 4. Eight have lost at least one CFE, and five have lost at least one of exon 7, 8, or 9. Six have a described NDD phenotype, four with a CFE deletion. Two patients with CFE deletions have no clinical information. This further suggests that the new fetal brain CFE coding isoforms described in the present work are crucial elements for the NDD phenotypes associated with *DLG2* deletions. It also leaves open the possibility that other *DLG2* exons may contribute to NDD phenotype penetrance.

Analyzing the *DLG2* cohort, we found that the mechanistic system explaining the NDD phenotypes was the loss of two new human promoters and coding first exons, CFEin7 and CFEin8. They code for the orthologous isoforms of murine PSD-93 beta and epsilon protein isoforms. Understanding in vivo their developmental role should provide useful knowledge to link tissue-specific expression of DLG protein isoforms and NDD development.

The comparison between human and mouse *DLG2/Dlg2* genes revealed a significant lack of cross-annotation in both reference genomes. For example, *DLG2* human exons 1–6 and 10 are unmapped to any mouse exons while these murine exons should exist. Because of the human–mouse genetic overlap and evolutionarily conserved properties, we expect that, as we already manually did for *DLG2*, an automated cross-species annotation analysis of DNA and protein information from UCSC, NCBI BLAST, and UniProtKB/Swiss-Prot databases will disclose novel exons in other genes.

Classic data-driven strategies study the enrichment in patients compared to controls one region at a time. Such univariate analysis is limited in detecting the possible complexity of NDDs. Here, a multivariate assessment was performed for the *DLG2* HPin7 and HPin8 statistical analysis, helping to reveal the importance of both regions. With accumulation of data and patients, multivariate data-driven analysis might become more relevant in deciphering NDDs.

In this work, we investigated NDD phenotypes, but the same methodology can be applied for other diseases of interest. Moreover, while the present work is focused on CNV data, using additional functional annotations, types of variant, and more sensitive whole genome sequencing analysis would be a logical development.

## Conclusions

Our work demonstrates the key importance of two new *DLG2* promoters and coding first exons for their association with neurodevelopmental phenotypes. It expands the *DLG2* NDD phenotypic spectrum to intellectual disability (GDD/ID, language delay) and behavioral disorders (ASD, ADHD). Through our manual investigation of *DLG2*/*Dlg2* exon mapping, it unveiled the lack of cross-annotation between the human and mouse reference genomes and between nucleotide and protein databases. Our study also emphasizes the importance of tissue-specific integrative studies along the developmental timeline to further explain developmental disorders.

## Additional files

Additional file 1: Supplementary figures and tables. (PDF 10077 kb)

Additional file 2: Knowledge-driven analysis results. (XLSX 13 kb)

## Abbreviations

ADHD: Attention deficit hyperactivity disorder
ASD: Autism spectrum disorder
CAGE: Cap analysis gene expression
CEGA: Conserved Elements from Genomics Alignments
CFE: Coding first exon
CGH: Complete genomic hybridization
CNV: Copy number variation
DECIPHER: Database of Chromosomal Imbalance and Phenotype in Humans Using Ensembl Resources
DGV: Database of Genomic Variants
EST: Expressed sequence tag
GDD: Global developmental delay
HP: H3K4me3 peak
HUDERF: Hôpital Universitaire Des Enfants Reine Fabiola
ID: Intellectual disability
IGV: Integrative Genome Viewer
NDD: Neurodevelopmental disorder
NPC: Neuronal progenitor cultured cell
PSD: Postsynaptic density
ULB: Université Libre de Bruxelles
WPPSI-R: Wechsler Preschool and Primary Scale of Intelligence
