# Supplementary material for: Novel promoters and coding first exons in DLG2 linked to developmental disorders and intellectual disability
Source: Genome Med. 2017 Jul 19;9:67. doi: 10.1186/s13073-017-0452-y (PMC5518101; doi:10.1186/s13073-017-0452-y)
Supplement: Supplementary file 1 — Supplementary figures and tables. (PDF 10077 kb) [file 13073_2017_452_MOESM1_ESM.pdf]

Additional file 1

Novel promoters and coding first exons in *DLG2* linked to developmental disorders and intellectual disability

Claudio Reggiani[1,2], Sandra Coppens[3,4,$], Tayeb Sekhara[4,24,$], Ivan Dimov[5,$], Bruno Pichon[6], Nicolas Lufin[1,6], Marie-Claude Addor[7], Elga Fabia Belligni[8], Maria Cristina Digilio[9], Flavio Faletra[10], Giovanni Battista Ferrero[8], Marion Gerard[11], Bertrand Isidor[12], Shelagh Joss[13], Florence Niel-Bütschi[7], Maria Dolores Perrone[10,25], Florence Petit[14], Alessandra Renieri[15,16], Serge Romana[17,18], Alexandra Topa[19], Joris Robert Vermeesch[20], Tom Lenaerts[1,2,21], Georges Casimir[22], Marc Abramowicz[1,6], Gianluca Bontempi[1,2,†], Catheline Vilain[1,6,23,†], Nicolas Deconinck[4,†] and Guillaume Smits[1,6,23,†,*]

[1]Interuniversity Institute of Bioinformatics in Brussels ULB-VUB, Brussels, 1050, Belgium.

[2]Machine Learning Group, Université Libre de Bruxelles, Brussels, 1050, Belgium.

[3]Department of Neurology, Hôpital Erasme, Université Libre de Bruxelles, Brussels, 1070, Belgium.

[4]Neuropediatrics, Hôpital Universitaire des Enfants Reine Fabiola, Université Libre de Bruxelles, Brussels, 1020, Belgium.

[5]Faculté de Médecine, Université Libre de Bruxelles, Brussels, 1070, Belgium.

[6]ULB Center of Medical Genetics, Hôpital Erasme, Université Libre de Bruxelles, Brussels, 1070, Belgium.

[7]Service de Médecine Génétique, Centre Hospitalier Universitaire Vaudois CHUV, Lausanne, 1011, Switzerland.

[8]Department of Public Health and Pediatrics, University of Torino, Turin, 10126, Italy.

[9]Medical Genetics, Bambino Gesù Pediatric Hospital, Rome, 00165, Italy.

[10]S.C. Medical Genetics, Institute for Maternal and Child Health - IRCCS "Burlo Garofolo", Trieste, 34137, Italy.

[11]Laboratory of Medical Genetics, CHU de Caen - Hôpital Clémenceau, Caen, 14033 Caen Cedex, France.

[12]Service de Génétique Médicale, CHU de Nantes, Nantes, 44093 Nantes Cedex 1, France.

[13]West of Scotland Clinical Genetics Service, South Glasgow University Hospitals, Glasgow, G51 4TF, United Kingdom.

[14]Service de Génétique, CHRU de Lille - Hôpital Jeanne de Flandre, Lille, 59000, France.

[15]Medical Genetics, University of Siena, Siena, 53100, Italy.

[16]Genetica Medica, Azienda Ospedaliera Universitaria Senese, Siena, 53100, Italy.

[17]Service d'Histologie Embryologie Cytogénétique, Hôpital Necker Enfants Malades, Paris, 75015, France.

[18]Université Paris Descartes - Institut IMAGINE, Paris, 75015, France.

[19]Department of Clinical Pathology and Genetics, Sahlgrenska University Hospital, Gothenburg, 413 45, Sweden.

[20]Department of Human Genetics, University of Leuven, Leuven, 3000, Belgium.

[21]AI lab, Vrije Universiteit Brussel, Brussels, 1050, Belgium.

[22]Pediatrics, Hôpital Universitaire des Enfants Reine Fabiola, Université Libre de Bruxelles, Brussels, 1020, Belgium.

[23]Genetics, Hôpital Universitaire des Enfants Reine Fabiola, Université Libre de Bruxelles, Brussels, 1020, Belgium.

[24]Present address: Neuropediatrics, Clinique Saint-Anne Saint-Rémy - CHIREC, Brussels, 1070, Belgium.

[25]Present address: Assisted Fertilization Department, Casa di Cura Città di Udine, Udine, 33100, Italy.

[$,†]These authors contributed equally to this work

[*]Correspondence: guillaume.smits@huderf.be

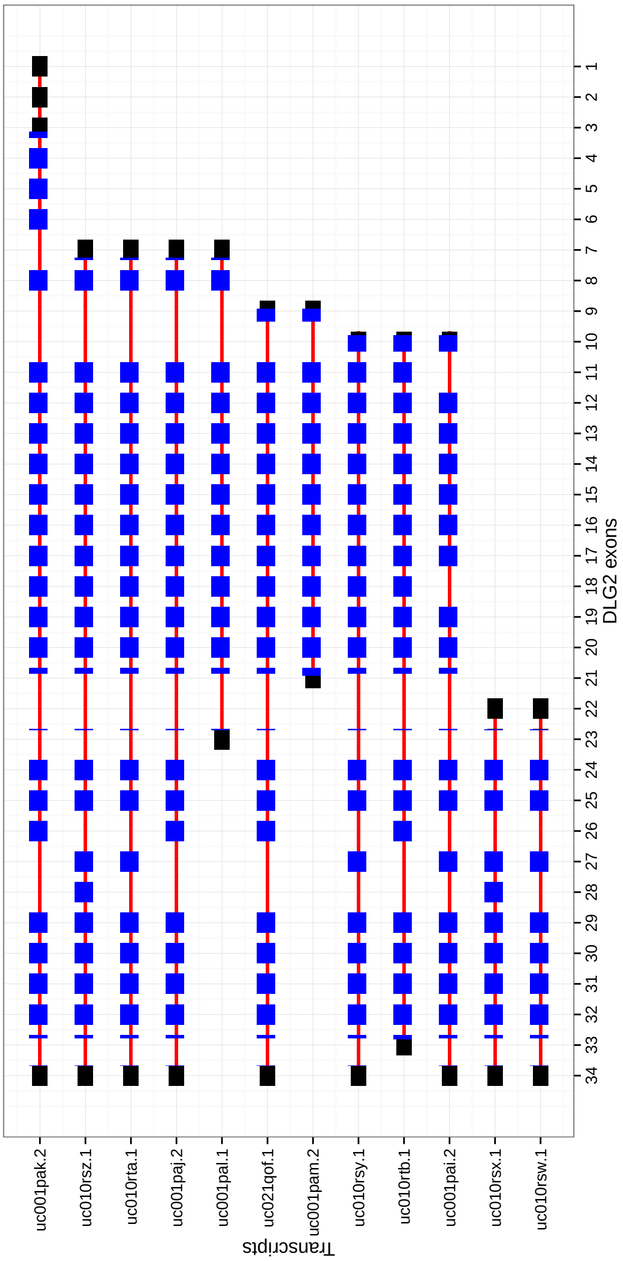

Figure S1: *DLG2* isoforms schematic representation using UCSC data. This representation visualizes exons across all isoforms and identifies which ones are shared. Each line represents a UCSC *DLG2* isoform, which is a combination of several components: blue boxes are cds regions, black boxes are non-coding regions such as 3'-UTR and 5'-UTR. Red lines are introns. In the y-axis are reported isoform names, *uc001pak.2* is the reference one. Many exons are shared across isoforms and considering that *DLG2* is an antisense gene, we numbered unique exons from right to left (reported in the x-axis). In Reggiani *et al.*, we refer to these exons using such numbers.

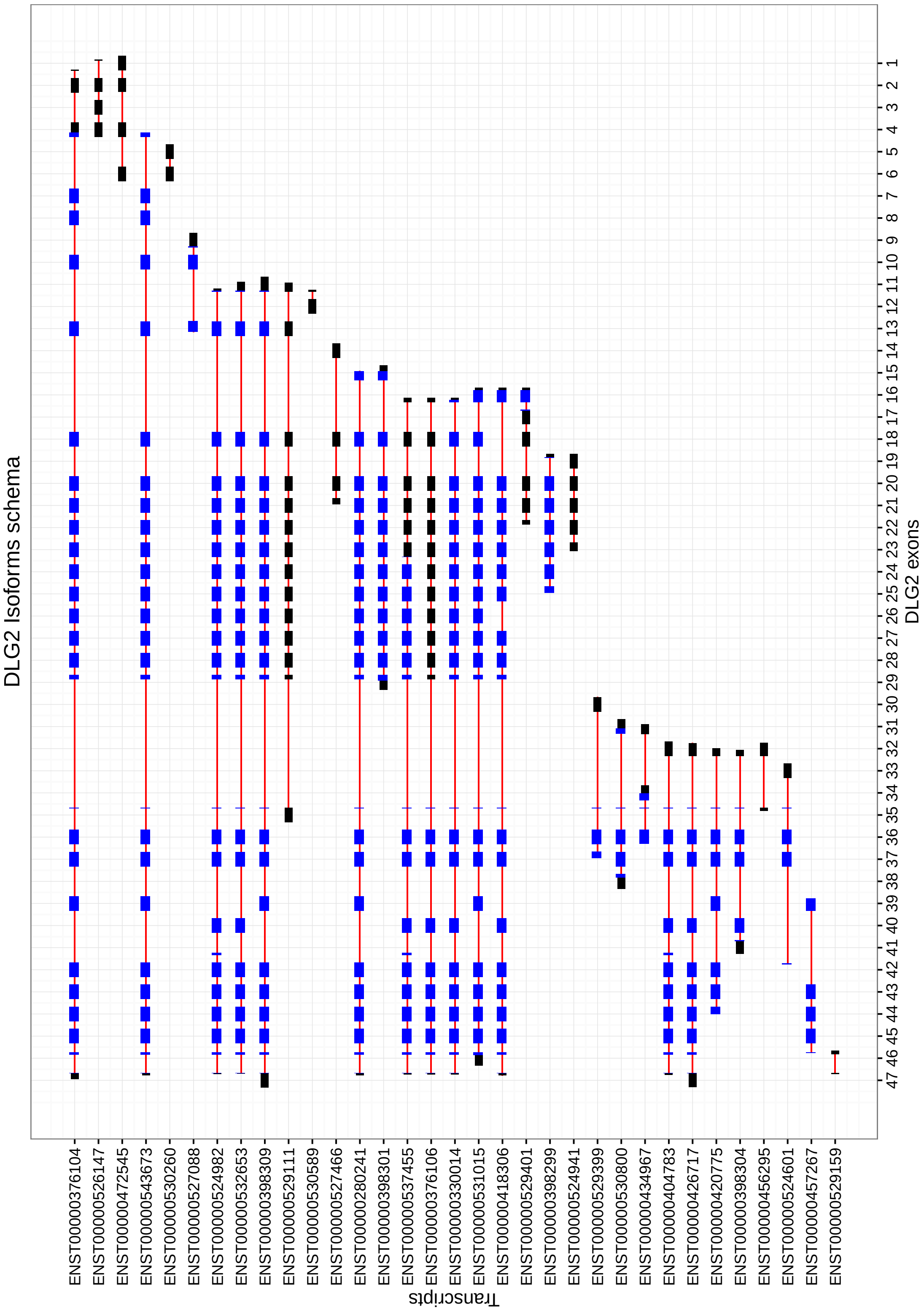

Figure S2: *DLG2* isoforms schematic representation using Ensembl data. This representation visualizes exons across all isoforms and identifies which ones are shared. Each line represents a Ensembl *DLG2* isoform, which is a combination of several components: blue boxes are cds regions, black boxes are non-coding regions such as 3'-UTR and 5'-UTR. Red lines are introns. In the y-axis are reported isoform names. Many exons are shared across isoforms and considering that *DLG2* is an antisense gene, we numbered unique exons from right to left (reported in the x-axis).

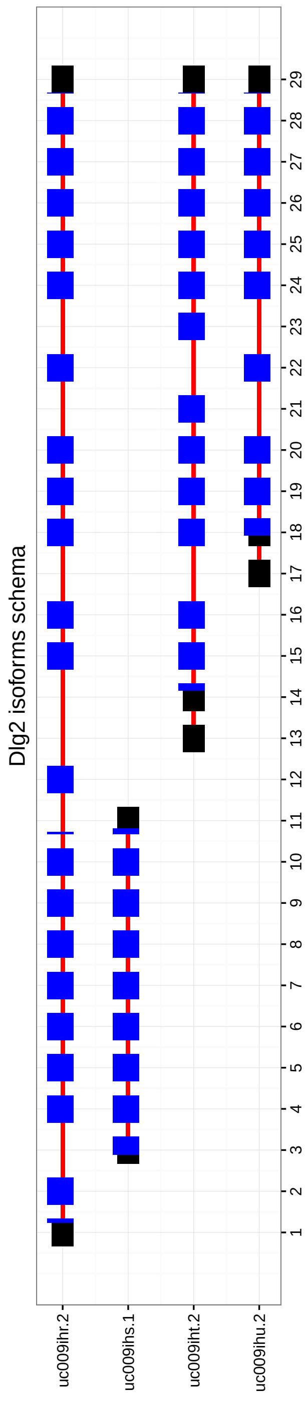

Figure S3: *Dlg2* isoforms schematic representation using UCSC data.

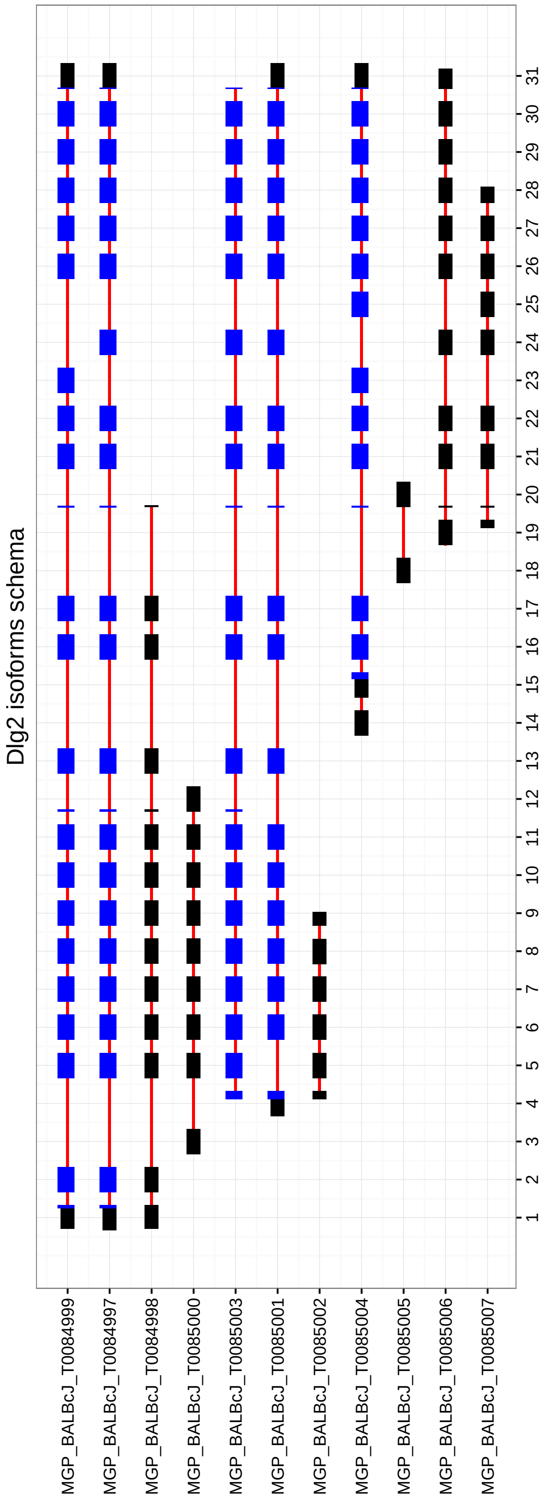

Figure S4: *Dlg2* isoforms schematic representation using Ensembl data (mm10, BALB/cJ strain).

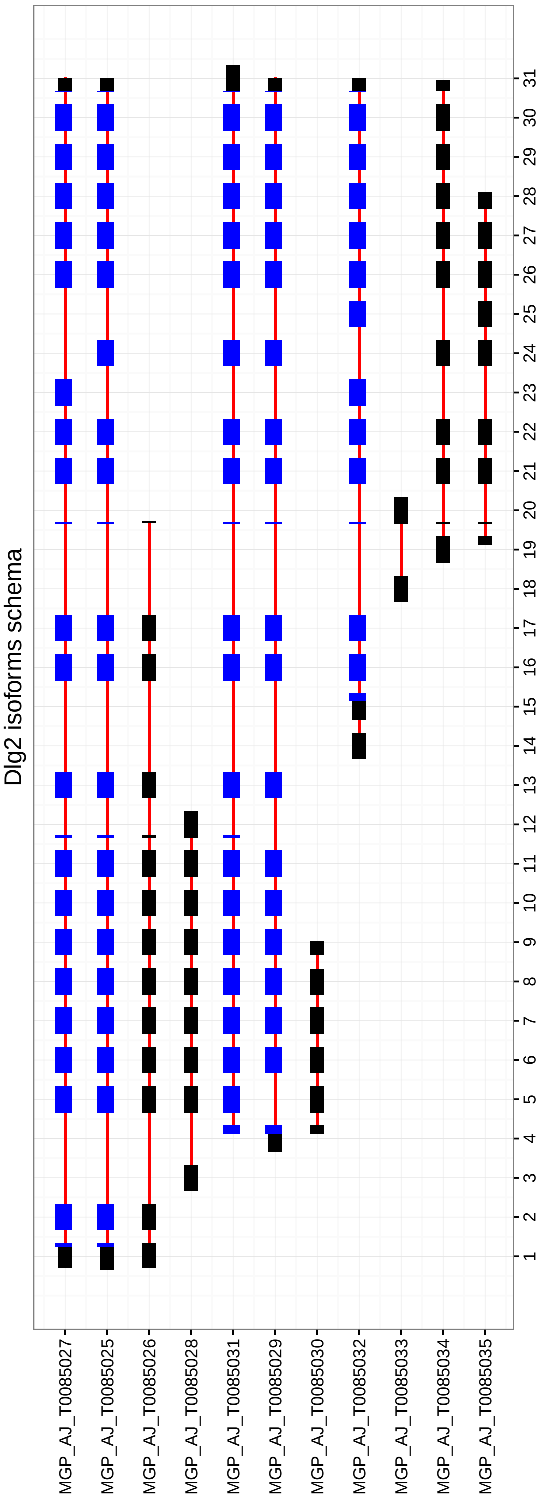

Figure S5: *Dlg2* isoforms schematic representation using Ensembl data (mm10, A/J strain).

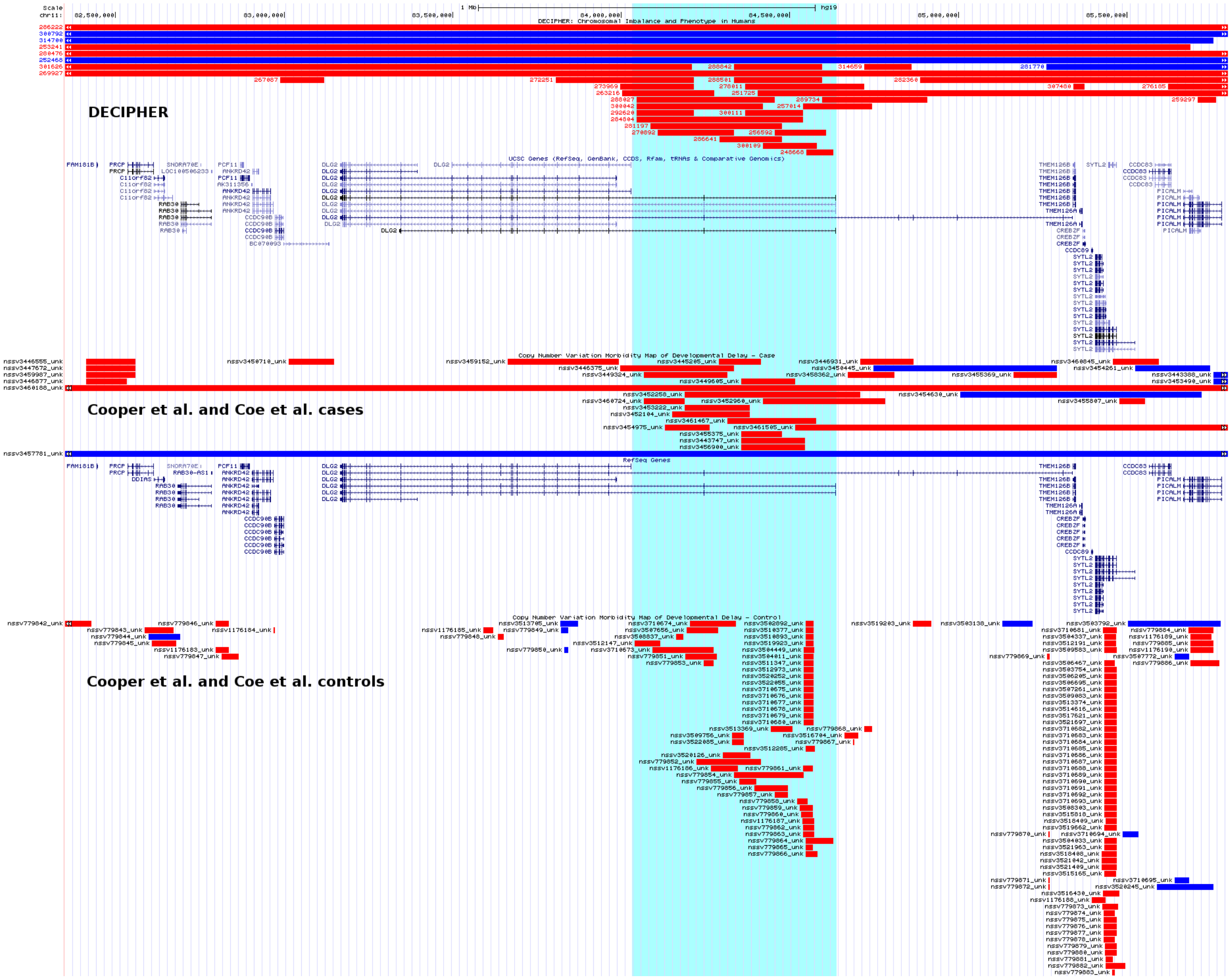

Figure S6: DECIPHER, Cooper *et al.* [1] and Coe *et al.* [2] (GDD/ID) cohorts. The light blue vertical region highlights the *DLG2* 7-9 region. With respect to Figure 1 describing the 29 patients, in this figure the DECIPHER tracks show two more patients which are not considered in analysis, 314659 and 257014, because in a later DECIPHER release. Patients 282360 and 251725 have CNVs larger than 3MB, therefore they have been filtered out in the preprocessing step.

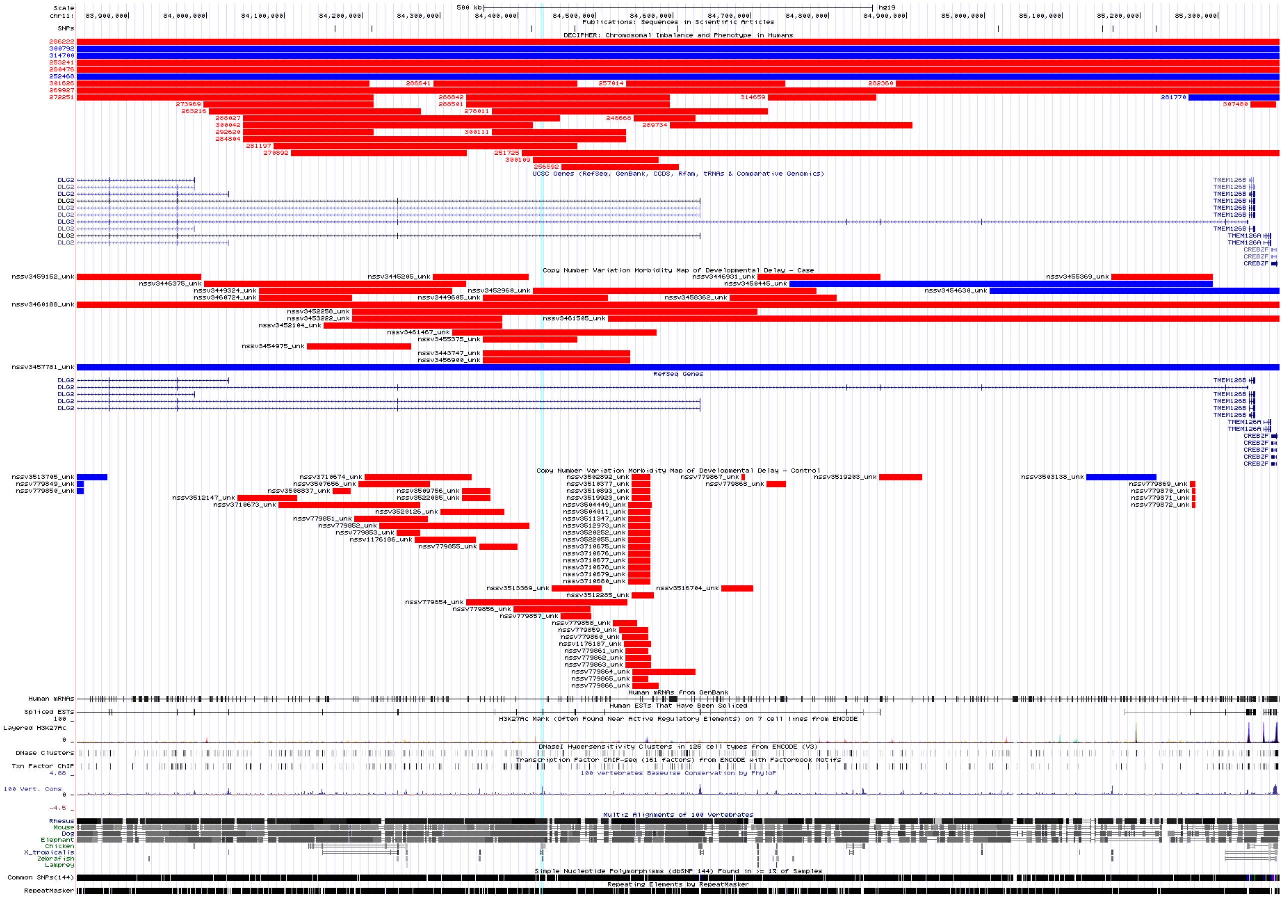

Figure S7: DECIPHER, Cooper *et al.* [1] and Coe *et al.* [2] (GDD/ID) cohorts. The light blue vertical region highlights HPin7. With respect to Figure 1 in the main article describing 29 patients, here the DECIPHER tracks show two more patients which are not considered in analysis, 314659 and 257014, because in a later DECIPHER release. Patients 282360 and 251725 have CNVs larger than 3MB, therefore they have been filtered out in the preprocessing step.

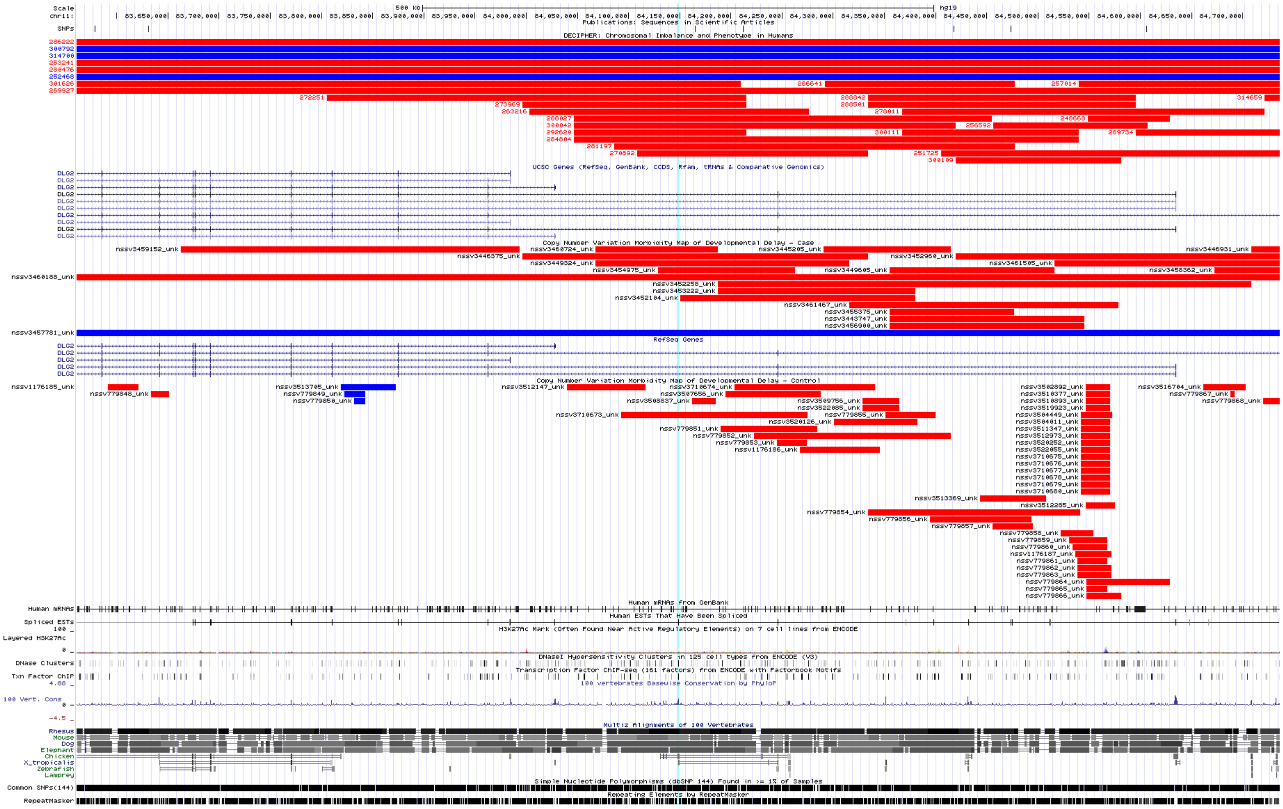

Figure S8: DECIPHER, Cooper *et al.* [1] and Coe *et al.* [2] (GDD/ID) cohorts. The light blue vertical region highlights HPin8. With respect to Figure 1 in the main article describing 29 patients, here the DECIPHER tracks show two more patients which are not considered in analysis, 314659 and 257014, because in a later DECIPHER release. Patients 282360 and 251725 have CNVs larger than 3MB, therefore they have been filtered out in the preprocessing step.

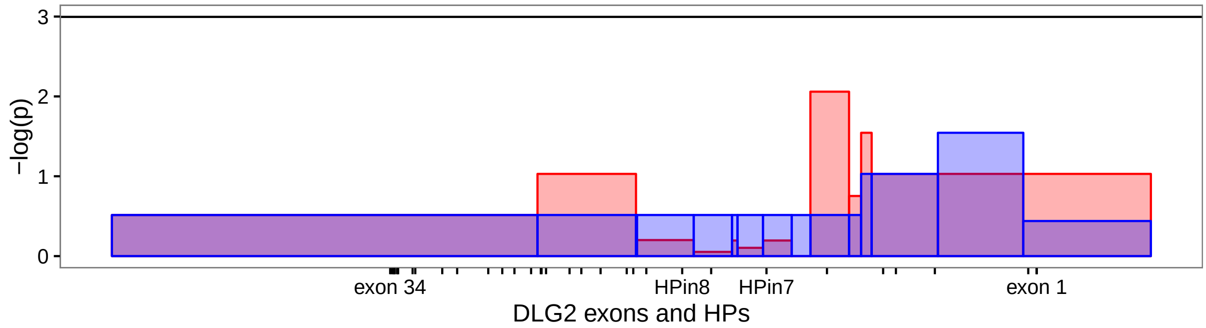

Figure S9: Partial reproduction of the windowed stastistical analysis (one-tailed Fisher's exact test p-values) on *DLG2*. [2] The horizontal bar at the top set the statistical significant threshold *-log(0.05)*. Blue and red colors stand for duplication and deletion analysis, respectively. The original data in hg18 has been converted in hg19 using LiftOver tool. According to Coe *et al.* [2] analysis, no statistical significant enrichment has been detected in *DLG2*, this is probably due to the choice of considering those CNVs intersecting with an exon, therefore filtering out all intronic CNVs.

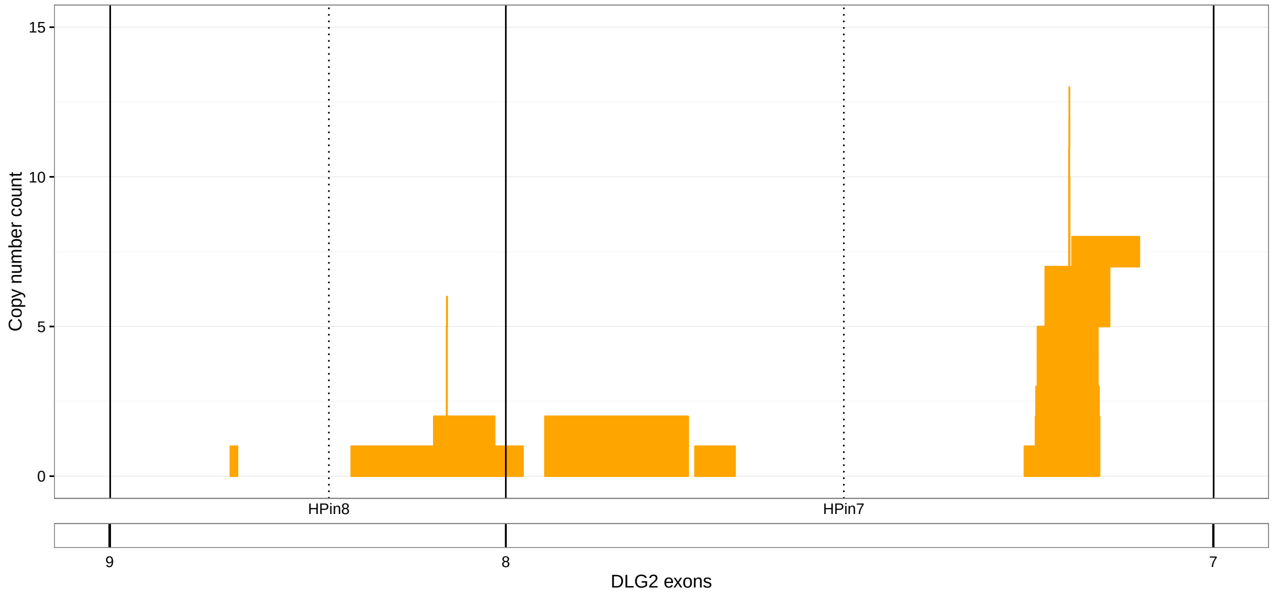

Figure S10: DGV deletions in *DLG2* 7-9 region. Data in Table S12.

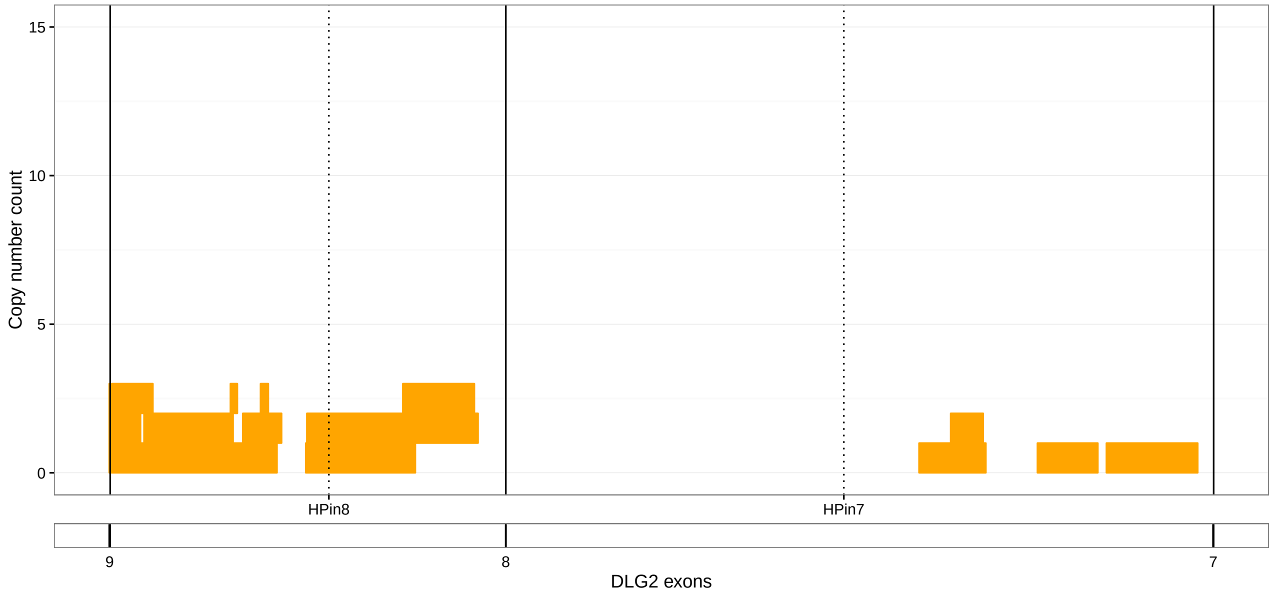

Figure S11: 1KG deletions in *DLG2* 7-9 region. Data in Table S13.

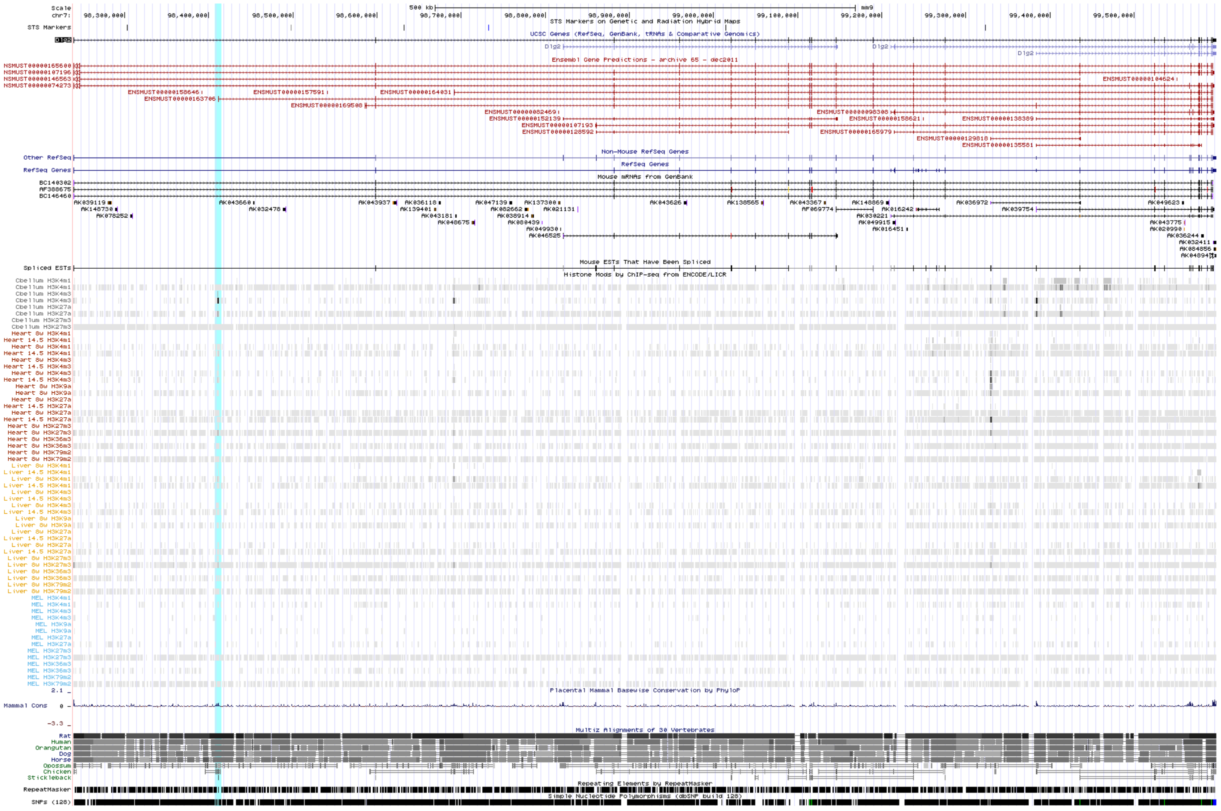

Figure S12: mHPin1 (corresponding to HPin7) in UCSC genome browser (mm9). For readability purposes, the vertical light blue region highlights a genomic range 9 times wider and centered on mHPin1. Such region overlaps ENSMUST00000158646 predicted isoform and H3K4me3 Chip-Seq peak in brain tissue.

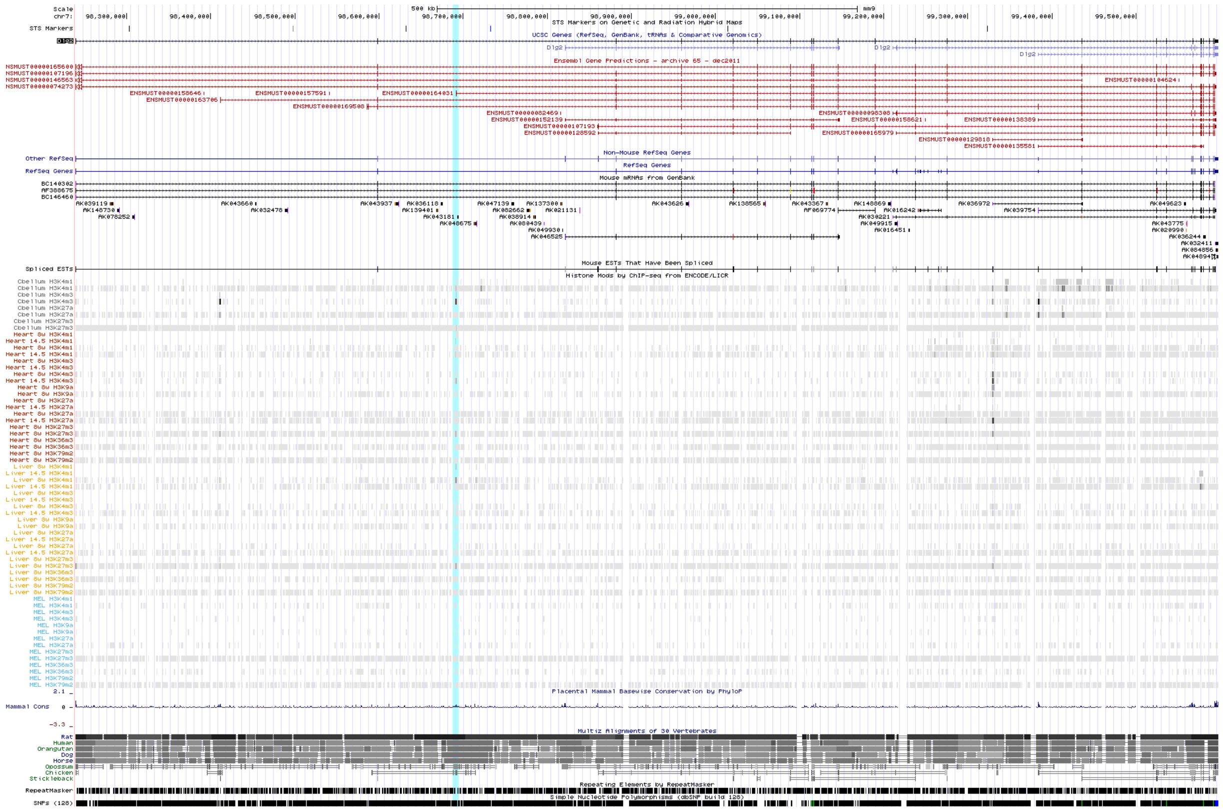

Figure S13: mHPin2 (corresponding to HPin8) in UCSC genome browser (mm9). For readability purposes, the vertical light blue region highlights a genomic range 9 times wider and centered on mHPin2. Such region overlaps ENSMUST00000164031 predicted isoform and H3K4me3 Chip-Seq peak in brain tissue.

Position	Length	Score	Status	Chromati...	DNase	TFBS	Other DB	
chr11 :84147915-84149126	1211	557	intronic			18		

Figure S14: CEGA conserved elements inside HPin8 (*Euarchontoglires* clade and *Homo Sapiens* species). *Score* represents the PhastCons score of the region. *DNase* grayscale value represents the DNA accessibility of the region using ENCODE data, using a percentage score black = 100% and white = 0% or data missing. The database reports one long highly conserved genomic region inside HPin8 and site for 18 transcription factors. The chromatin state column (see CEGA website for legend) suggests HPin8 as weak/inactive/poised promoter (in 9 ENCODE cell lines).

Position	Length	Score	Status	Chromati...	DNase	TFBS	Other DB	
chr11 :84430606-84430809	203	54	intronic			1		
chr11 :84431909-84432233	324	97	intronic			2		
chr11 :84432330-84432392	62	20	intronic			1		

Figure S15: CEGA conserved elements inside HPin7 (*Euarchontoglires* clade and *Homo Sapiens* species). *Score* represents the PhastCons score of the region. *DNase* grayscale value represents the DNA accessibility of the region using ENCODE data, using a percentage score black = 100% and white = 0% or data missing. The database reports three genomic regions inside HPin7 overlapping a total of 4 transcription factors. The chromatin state column (see CEGA website for legend) suggests HPin7 as weak/inactive/poised promoter (in 9 ENCODE cell lines).

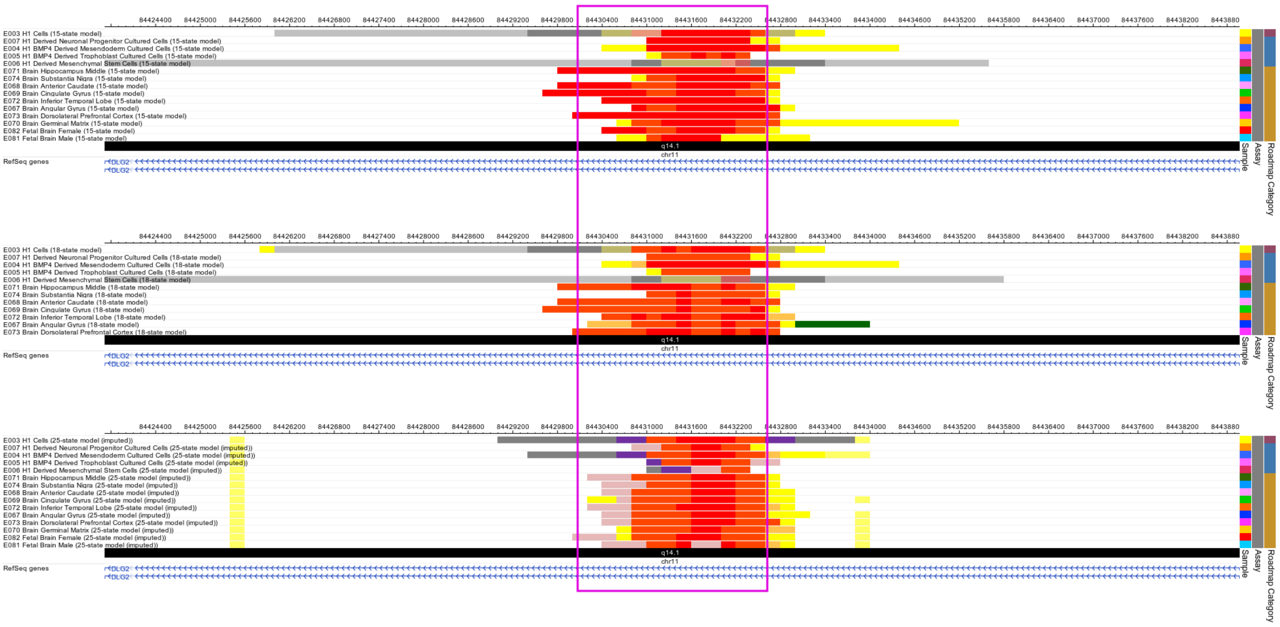

Figure S16: Roadmap Epigenomics 15-state, 18-state and 25-state models in HPin7 (purple box). Figure S18 reports the color legend

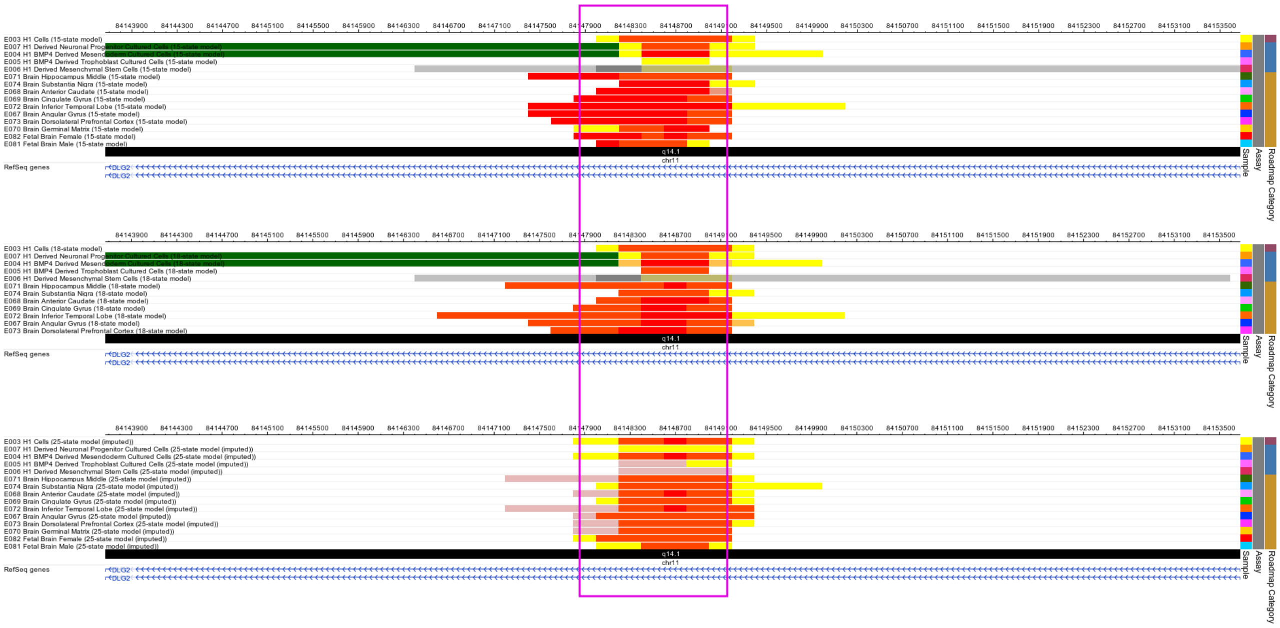

Figure S17: Roadmap Epigenomics 15-state, 18-state and 25-state models in HPin8 (purple box). Figure S18 reports the color legend

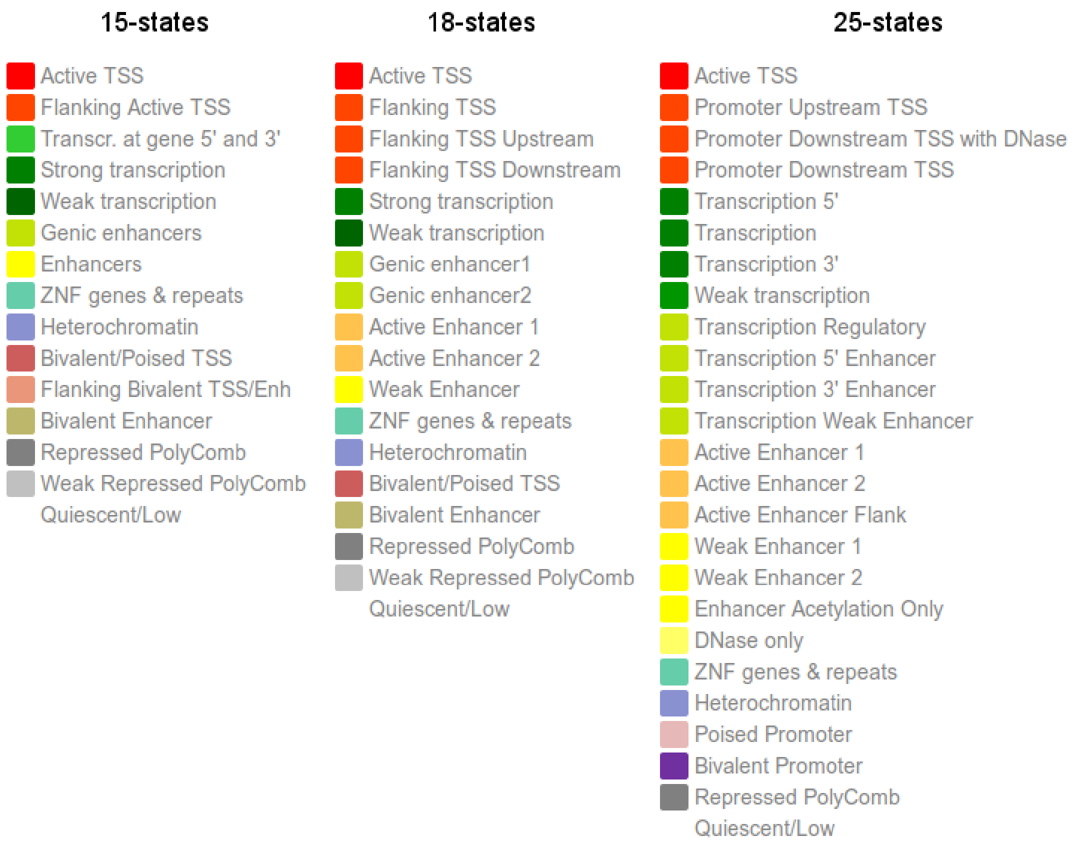

Figure S18: Roadmap Epigenomics color legend.

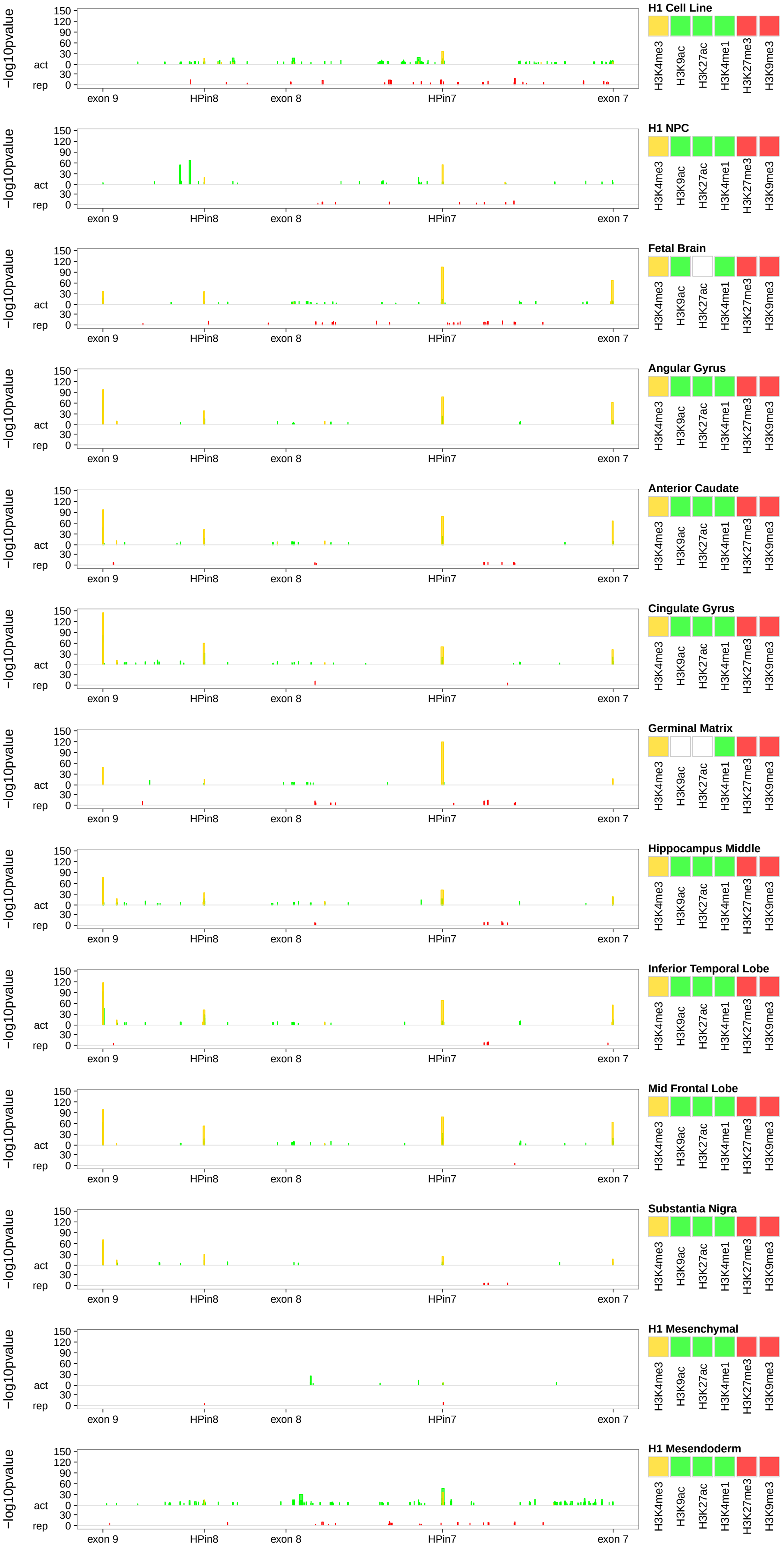

Figure S19: Roadmap epigenomic peaks across *DLG2* 7-9 region.

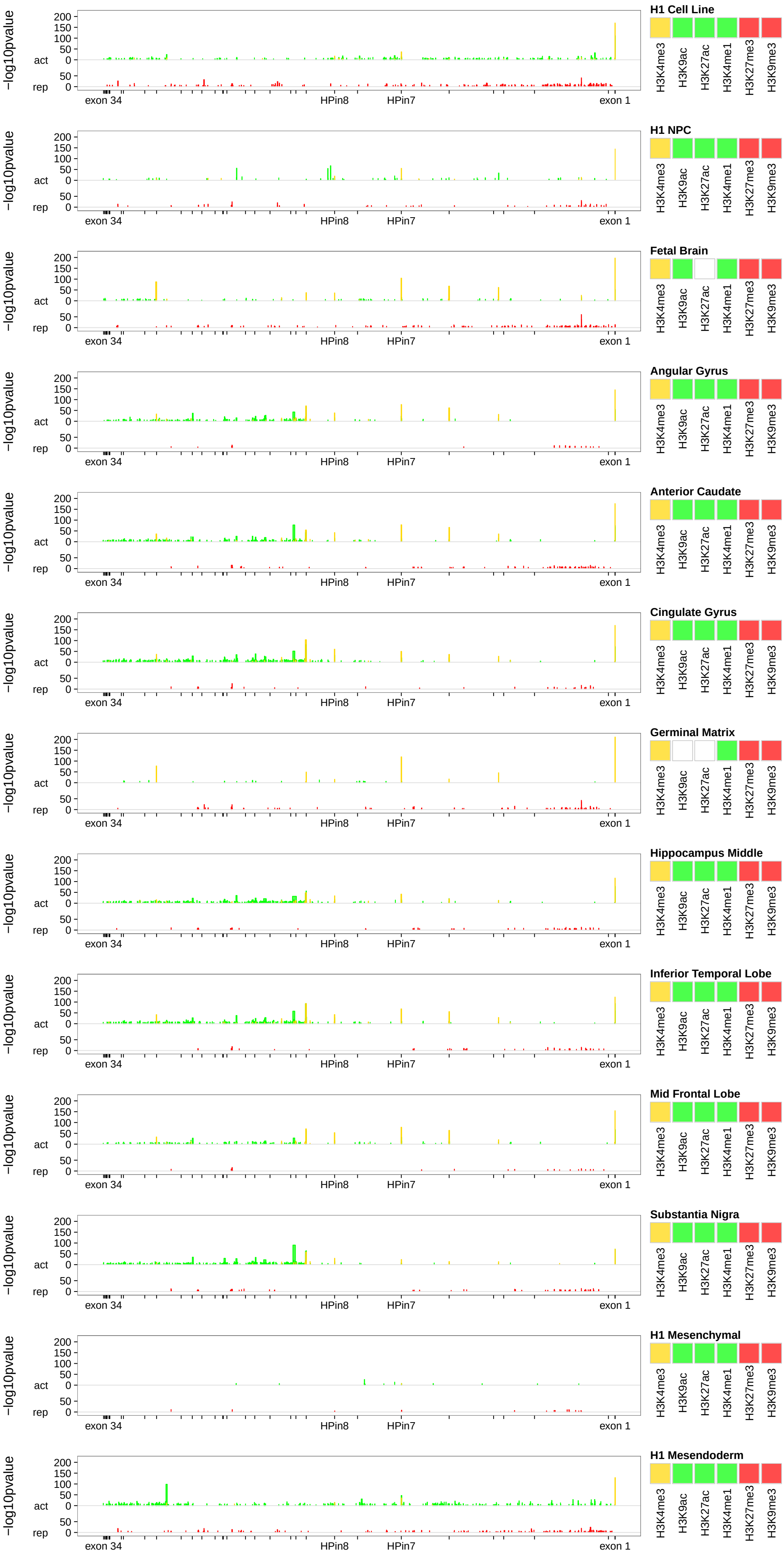

Figure S20: Roadmap epigenomic peaks across *DLG2* gene.

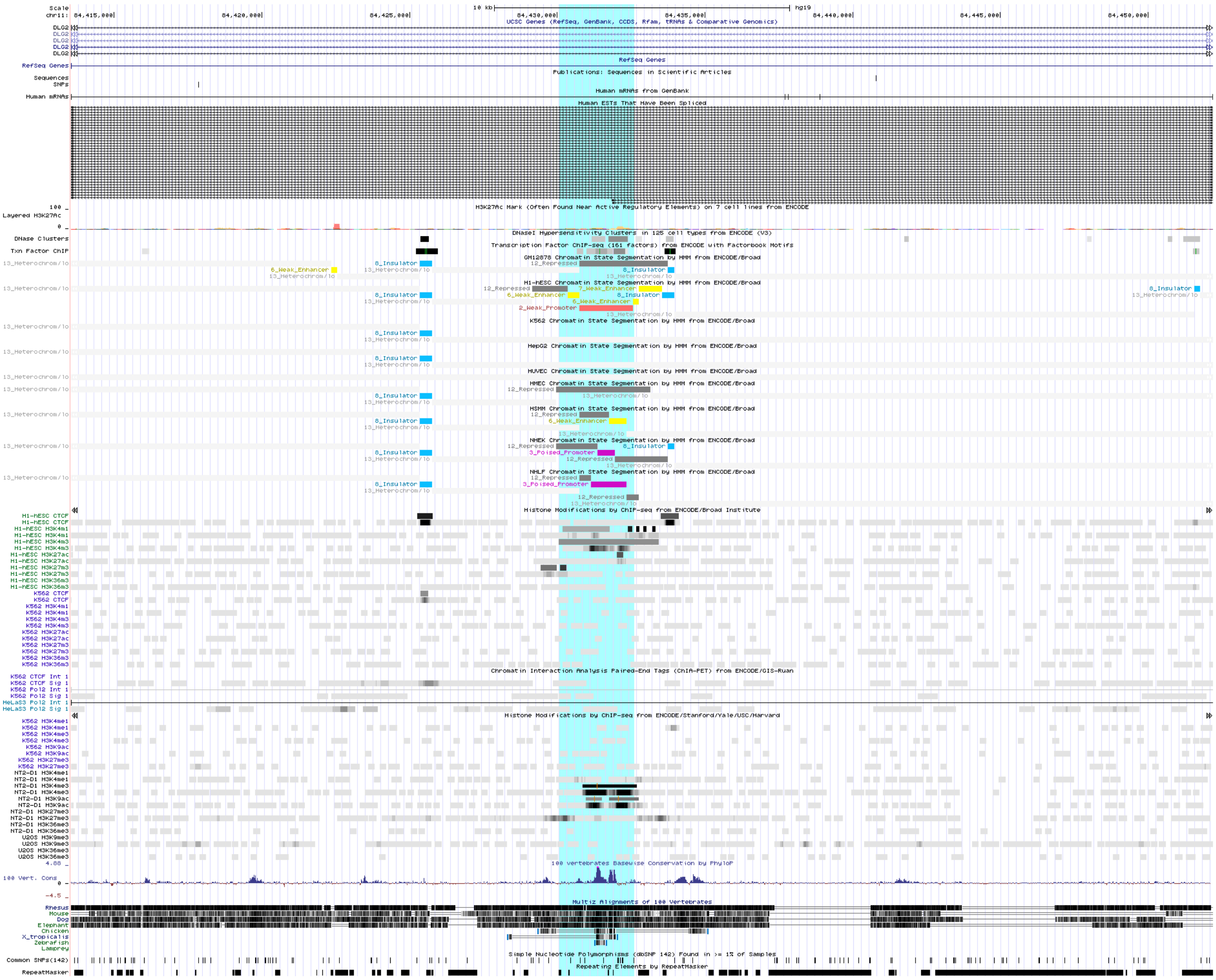

Figure S21: HPin7 region integrated with human EST and epigenomics data. Epigenomics data from ENCODE database repeatedly suggest the presence of a poised promoter, a repressed functional element or a weak enhancer/promoter in non-brain tissue. Human ESTs (sense) sequence name which begins are included in the highlighted region are AA180967 and AA180882.

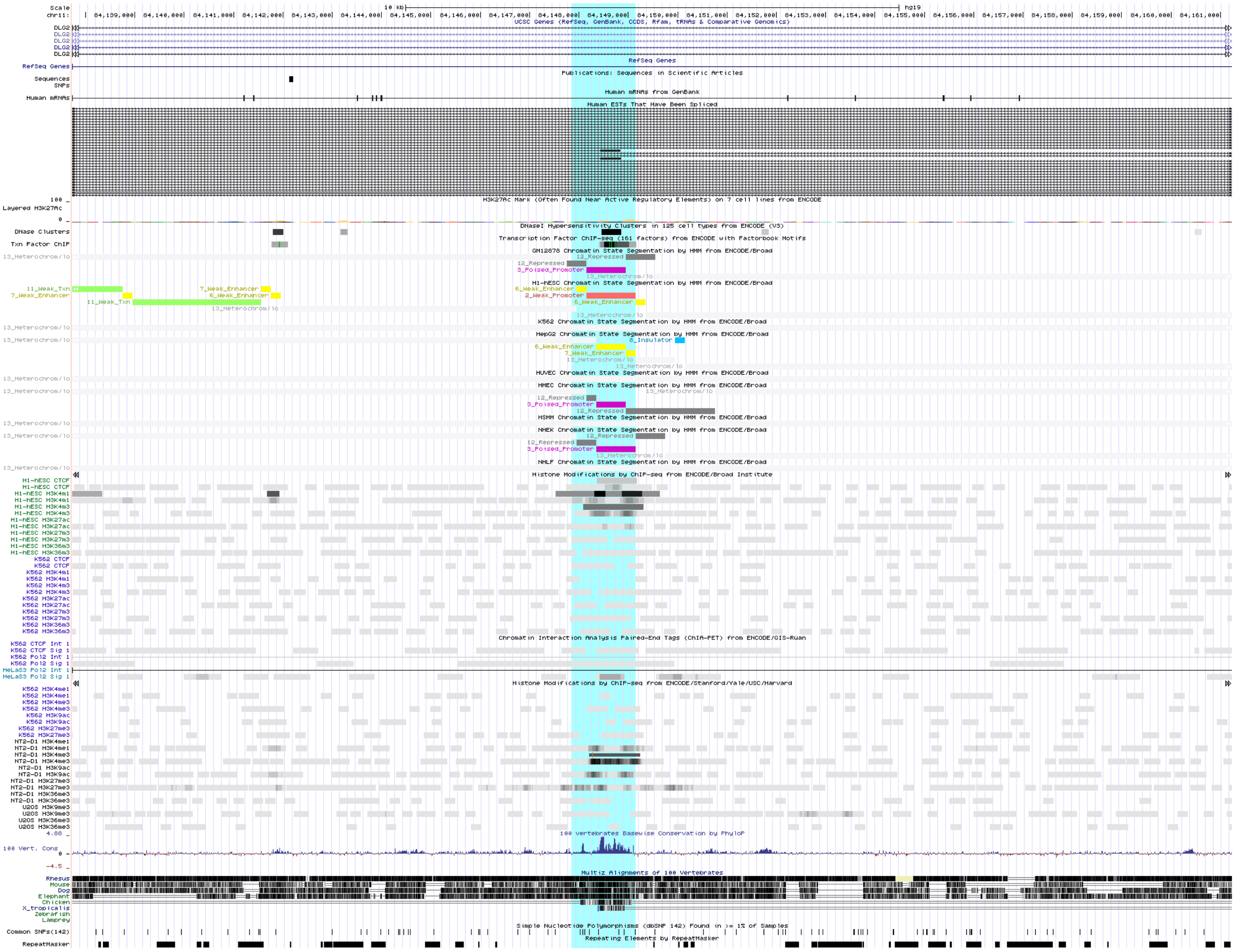

Figure S22: HPin8 region integrated with human EST and epigenomics data. Epigenomics data from ENCODE database repeatedly suggest the presence of a poised promoter, a repressed functional element or a weak enhancer/promoter in non-brain tissue. Human ESTs (antisense) sequence name which begins are included in the highlighted region are DA163026 and DA357282.

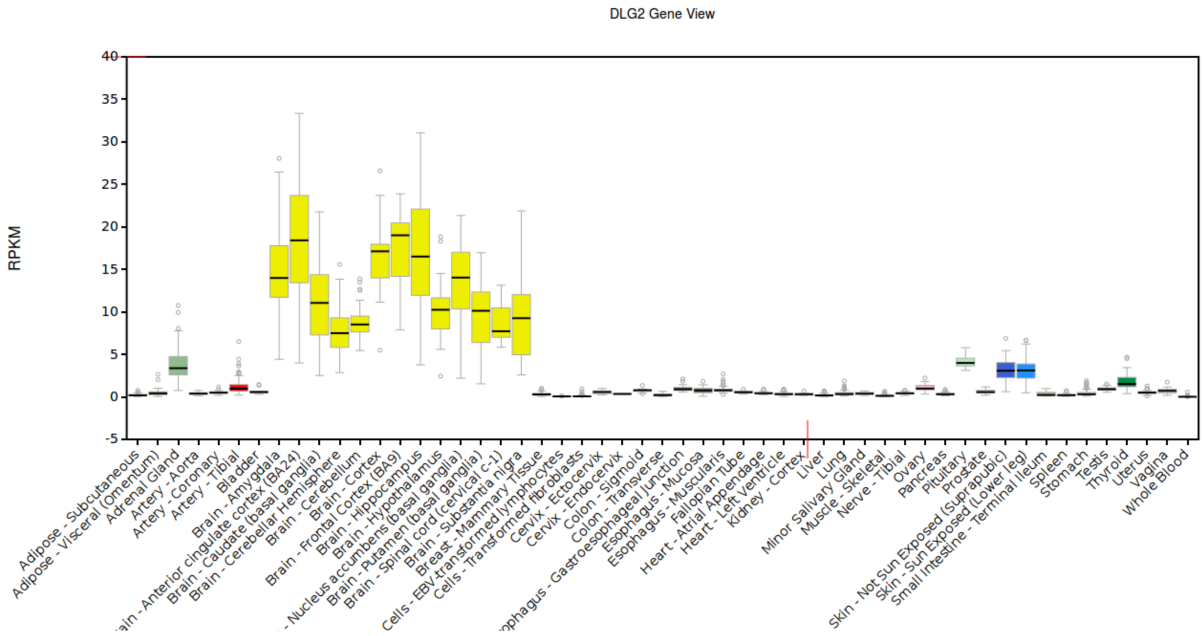

Figure S23: *DLG2* gene expression across tissues (linear scale), from GTEx Project (date 23 Sept 2015). Please note that GTEx studies only known isoforms expression, hence, HPs isoforms were not evaluated by the GTEx project.

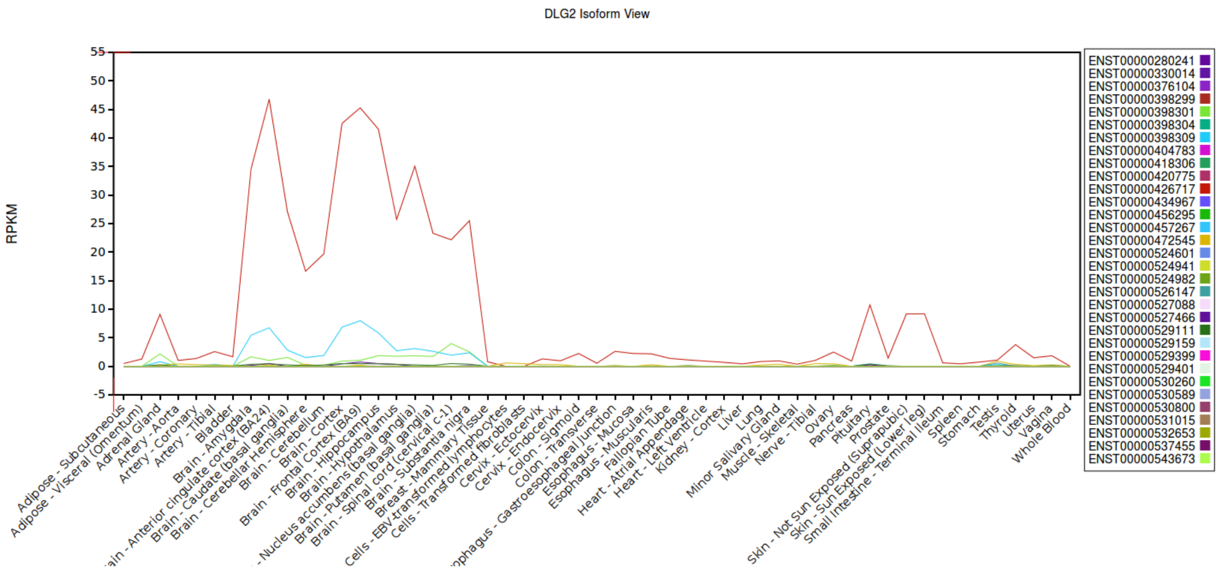

Figure S24: *DLG2* (Ensembl) isoforms expression across tissues (linear scale), from GTEx Project (date 23 Sept 2015). Please note that GTEx studies only known isoforms expression, hence, HPs isoforms were not evaluated by the GTEx project.

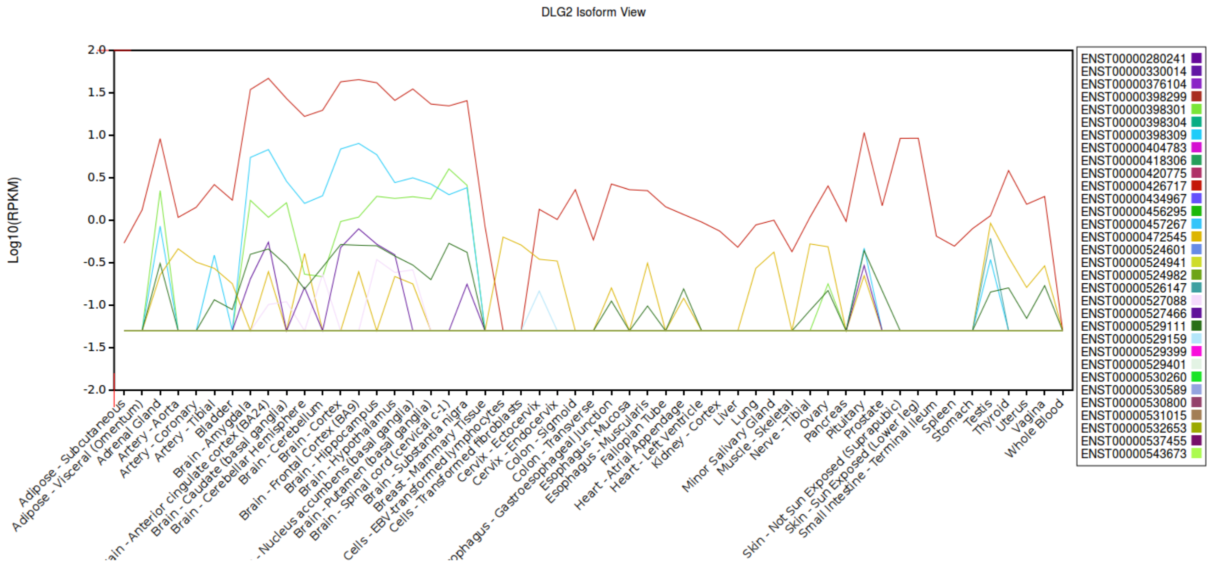

Figure S25: *DLG2* (Ensembl) isoforms expression across tissues (log scale), from GTEx Project (date 23 Sept 2015). Please note that GTEx studies only known isoforms expression, hence, HPs isoforms were not evaluated by the GTEx project.

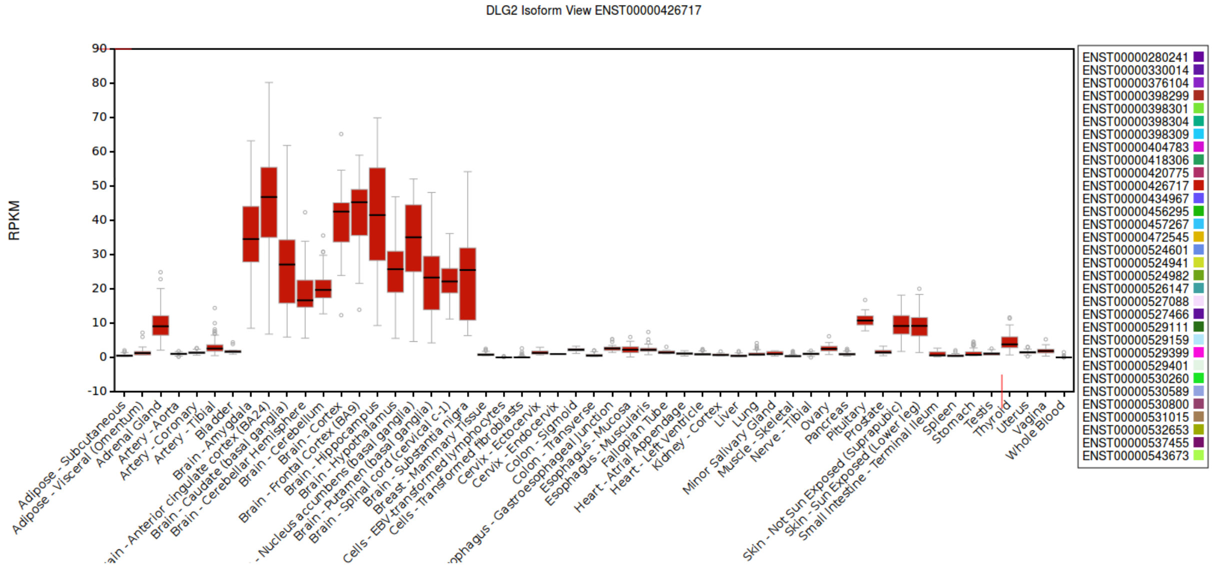

Figure S26: ENST00000426717 expression across tissues (linear scale), from GTEx Project (date 23 Sept 2015). It starts at exon 32, Ensembl numbering (or exon 22 in UCSC numbering; see Figure S29 and Table S3). Please note that GTEx studies only known isoforms expression, hence, HPs isoforms were not evaluated by the GTEx project.

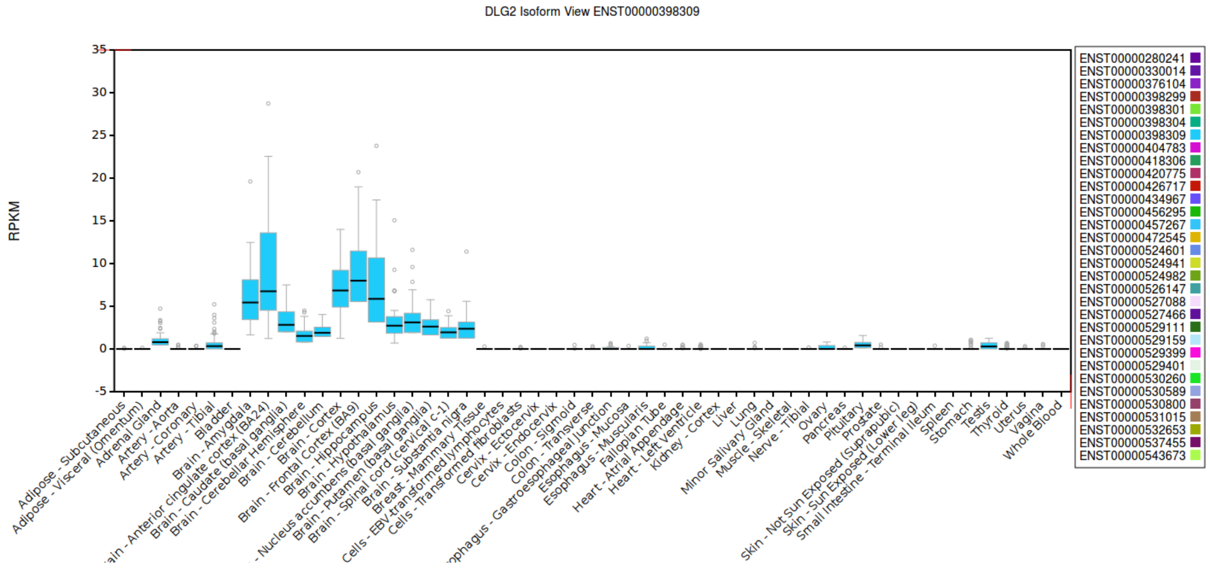

Figure S27: ENST00000398309 expression across tissues (linear scale), from GTEx Project (date 23 Sept 2015). It starts at exon 11, Ensembl numbering (or exon 7 in UCSC numbering; see Figure S29 and Table S3). Please note that GTEx studies only known isoforms expression, hence, HPs isoforms were not evaluated by the GTEx project.

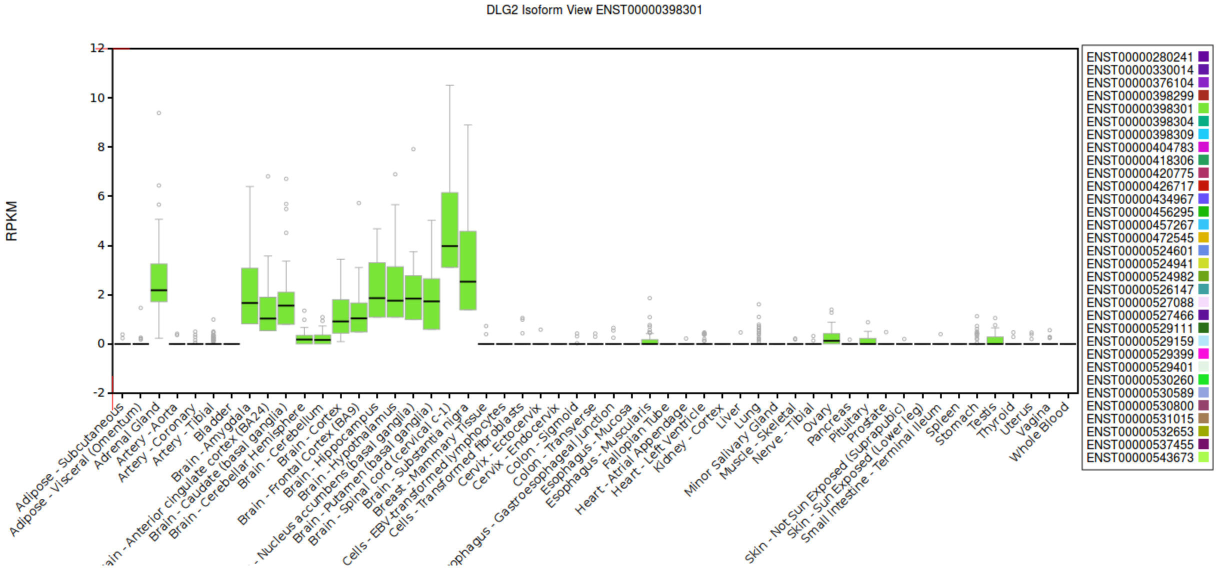

Figure S28: ENST00000398301 expression across tissues (linear scale), from GTEx Project (date 23 Sept 2015). It starts at exon 15, Ensembl numbering (or exon 9 in UCSC numbering; see Figure S29 and Table S3). Please note that GTEx studies only known isoforms expression, hence, HPs isoforms were not evaluated by the GTEx project.

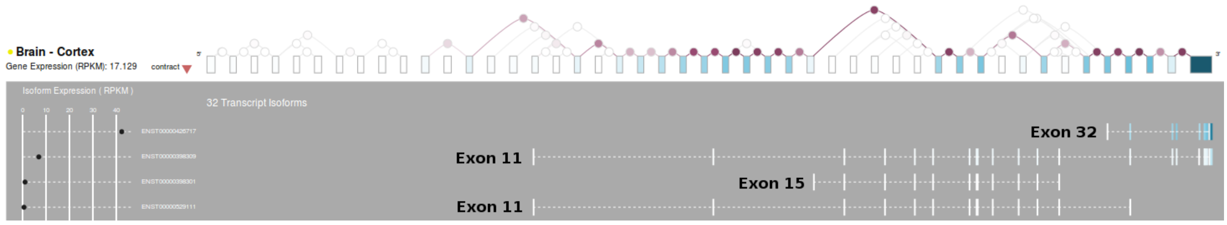

Figure S29: *DLG2* isoforms details (brain cortex), from GTEx Project (date 23 Sept 2015). In Enseml, exon 11 is located in chr11:84634120-84634633, which corresponds to exon 7 in UCSC; exon 15 is located in chr11:84027868-84028382, which corresponds to exon 9 in UCSC. The second and the third most expressed *DLG2* isoforms start at UCSC exon 7 and 9, respectively, suggesting that any PRE deletion might suppress or reduce their expression. The most expressed isoform starts at exon 32: chr11:83393201-83393468 (exon 22 in UCSC numbering). Please note that GTEx studies only known isoforms expression, hence, HPs isoforms were not evaluated by the GTEx project.

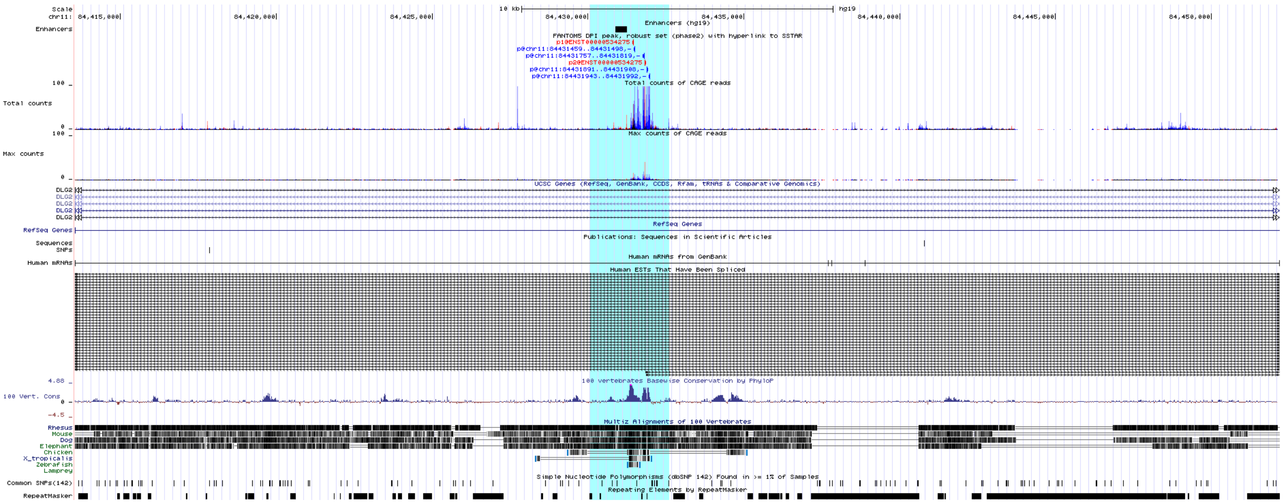

Figure S30: HPin7 region integrated with human EST, FANTOM5 and epigenomics data. The start of Ensembl human (sense) ESTs AA180967 and AA180882 are included in the HPin7 region (light-blue). Multiple detected peaks and human ESTs in HPs (highlighted with the light-blue region) suggest the beginning of a transcription.

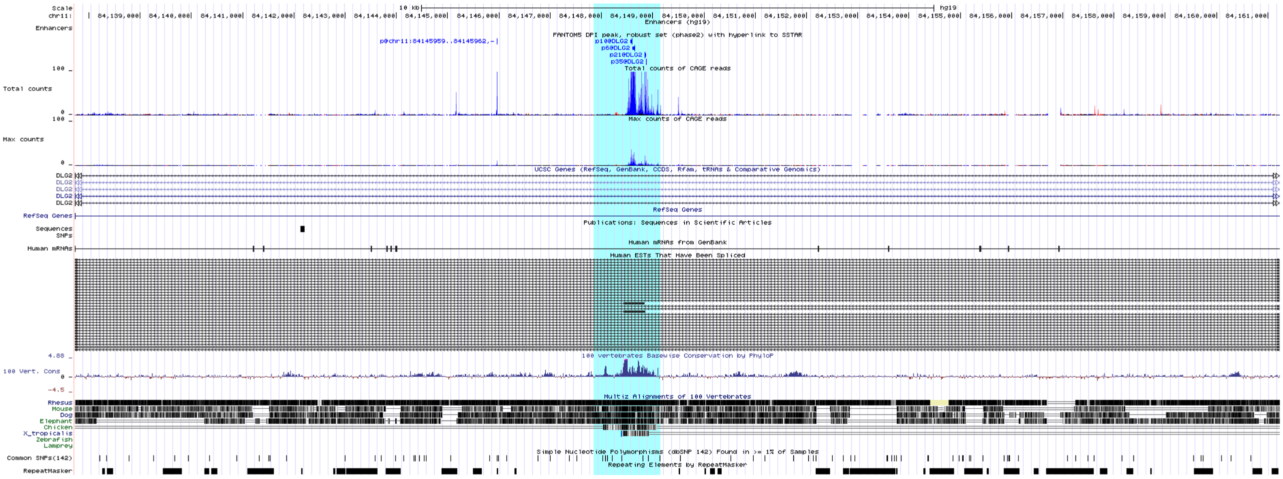

Figure S31: HPin8 region integrated with human EST and FANTOM5 data. The start of Ensembl human (antisense) ESTs DA163026 and DA357282 are included in the HPin8 region (light-blue). Multiple detected peaks and human ESTs in HPs (highlighted with the light-blue region) suggest the beginning of a transcription.

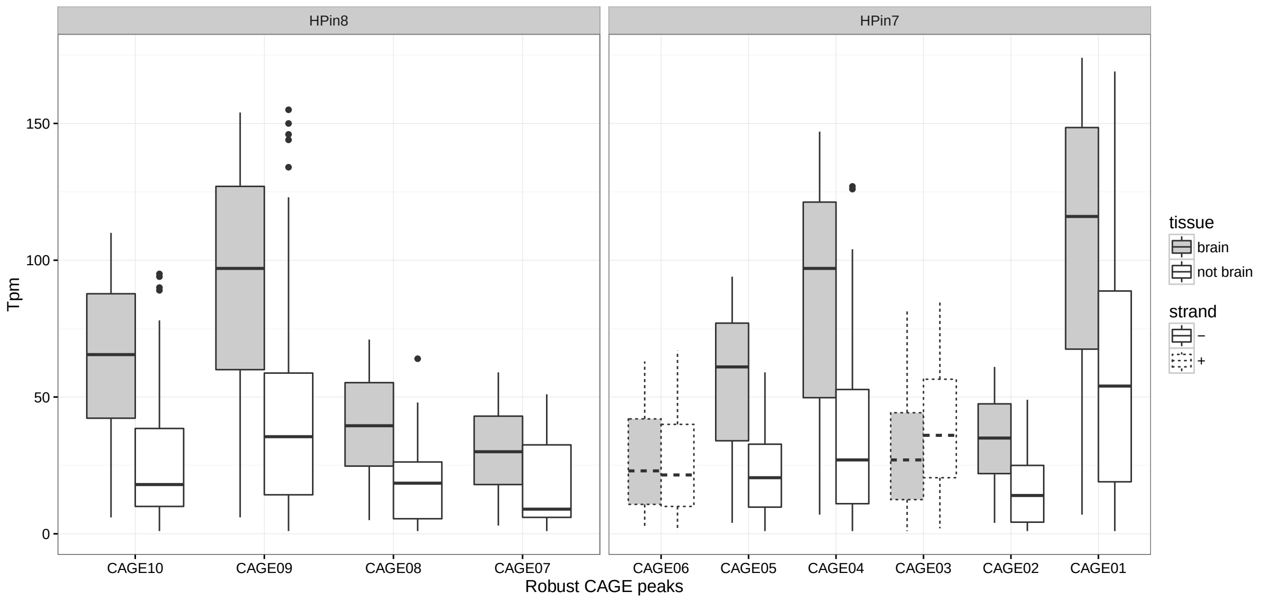

Figure S32: Brain vs other tissue-expression values for every CAGE robust peak found inside HPs. See Table S22 for coordinates.

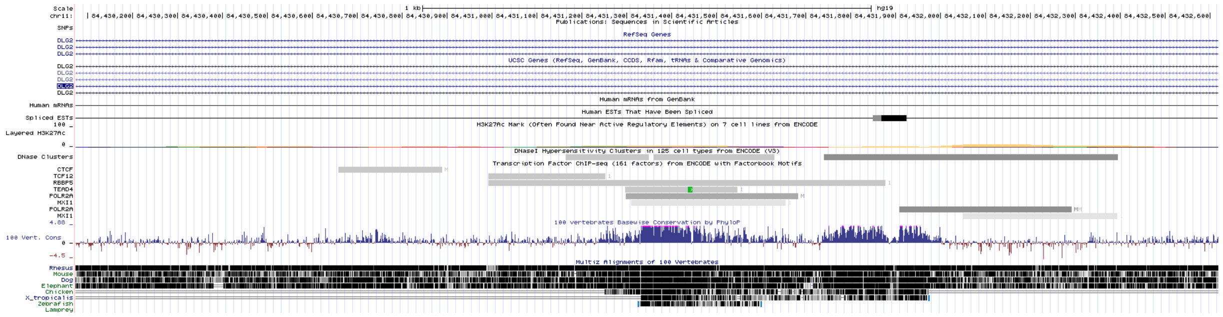

Figure S33: Transcription factors in HPin7 from ENCODE (UCSC Genome browser image).

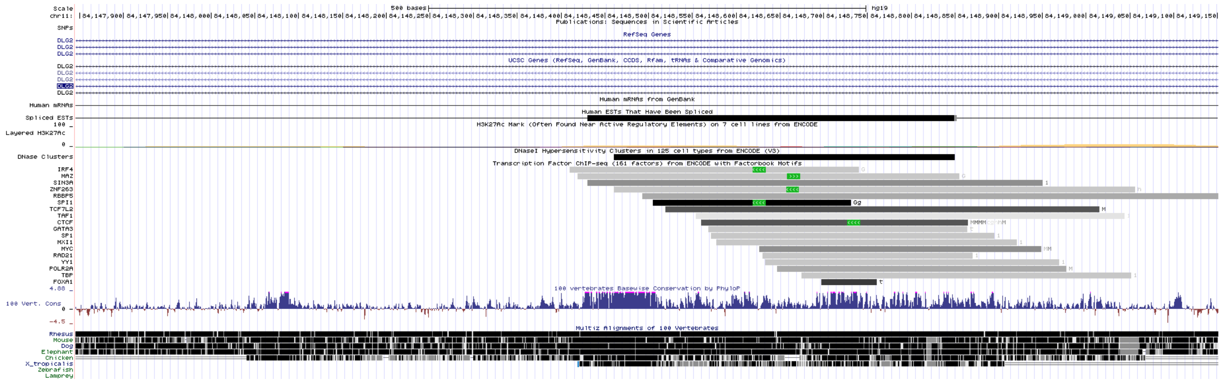

Figure S34: Transcription factors in HPin8 from ENCODE (UCSC Genome browser image).

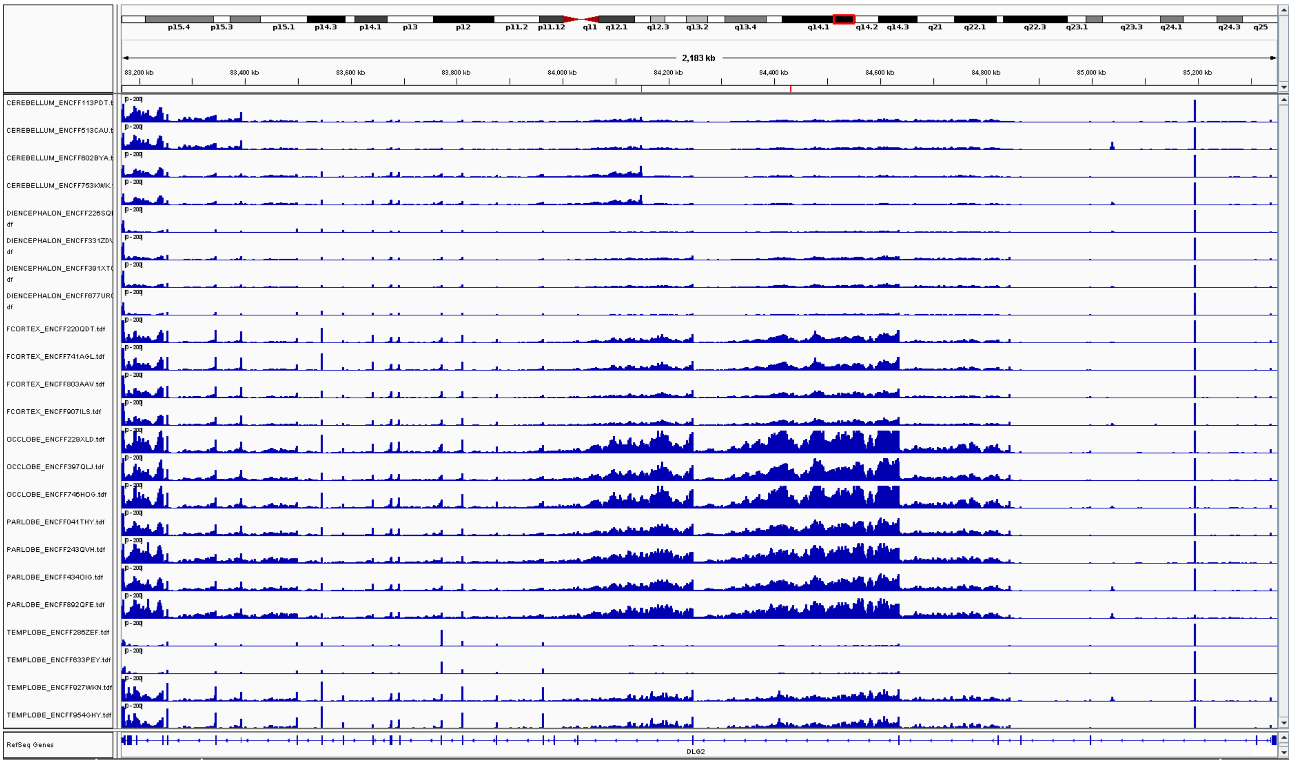

Figure S35: IGV visualization of ENCODE Fetal Brain BAM files coverage in *DLG2* gene. On the left column, *.tdf* files have been named with the tissue name and ENCODE id of the BAM. The top panel shows the region of interest in the chromosome, genomic coordinates and two red vertical lines corresponding to HPin8 and HPin7 (from left to right), respectively. The bottom panel shows the RefSeq *DLG2* gene with exons. In the main panel, the y-scales of all tracks have been manually set and fix to the 0-200 range. This figure shows that HPin7 and HPin8 belong to transcribed regions, and a better overview of those regions is provided in Figures S39 and S40.

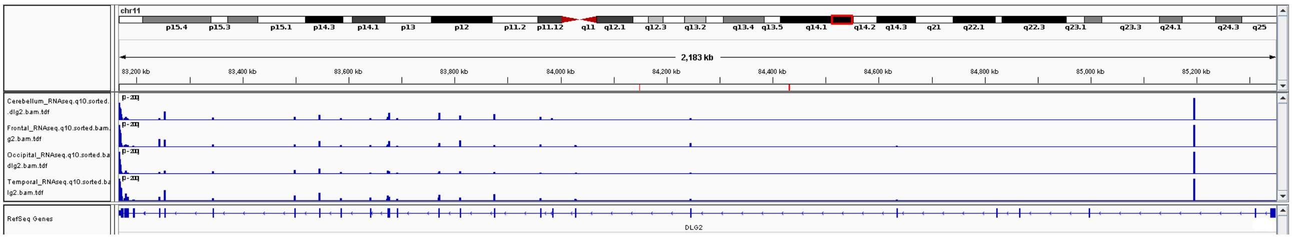

Figure S36: IGV visualization of Adult Brain BAM files coverage in *DLG2* gene from [3]. On the left column, *.tdf* files have been named with the tissue name. The top panel shows the region of interest in the chromosome, genomic coordinates and two red vertical lines corresponding to HPin8 and HPin7 (from left to right), respectively. The bottom panel shows the RefSeq *DLG2* gene with exons. In the main panel, the y-scales of all tracks have been manually set and fix to the 0-200 range.

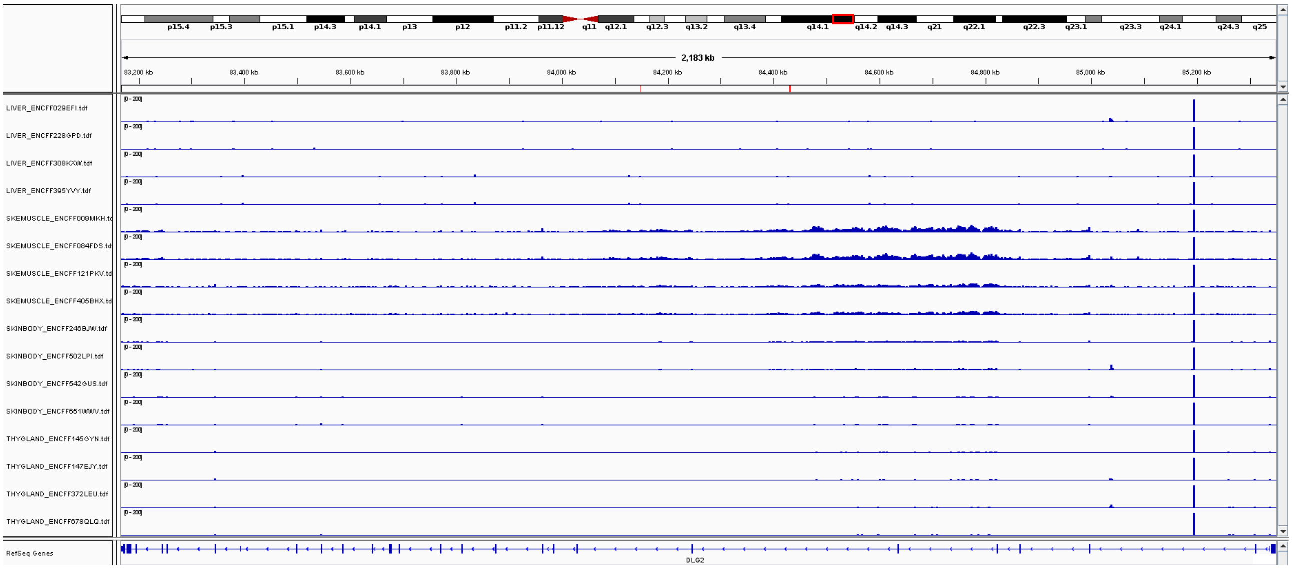

Figure S37: IGV visualization of ENCODE Fetal non-Brain BAM files coverage in *DLG2* gene. On the left column, *.tdf* files have been named with the tissue name and ENCODE id of the BAM. The top panel shows the region of interest in the chromosome, genomic coordinates and two red vertical lines corresponding to HPin8 and HPin7 (from left to right), respectively. The bottom panel shows the RefSeq *DLG2* gene with exons. In the main panel, the y-scales of all tracks have been manually set and fix to the 0-200 range.

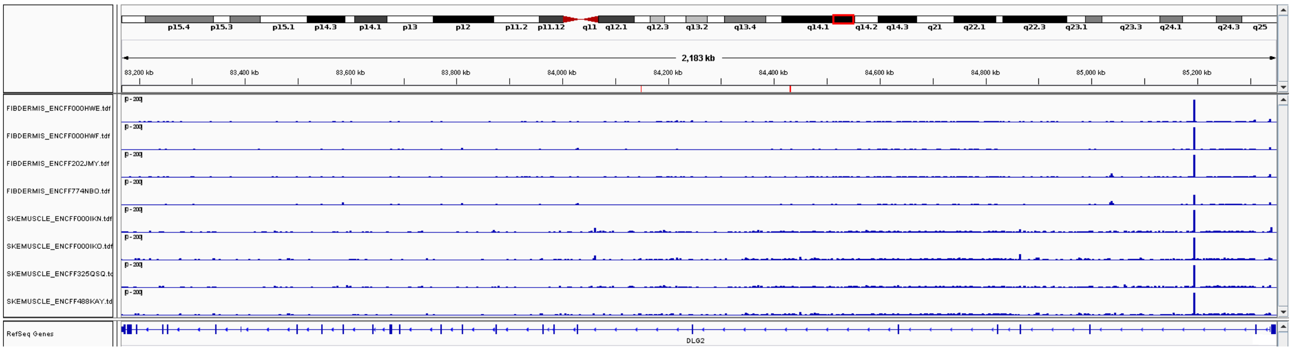

Figure S38: IGV visualization of ENCODE Adult non-Brain BAM files coverage in *DLG2* gene. On the left column, *.tdf* files have been named with the tissue name and ENCODE id of the BAM. The top panel shows the region of interest in the chromosome, genomic coordinates and two red vertical lines corresponding to HPin8 and HPin7 (from left to right), respectively. The bottom panel shows the RefSeq *DLG2* gene with exons. In the main panel, the y-scales of all tracks have been manually set and fix to the 0-200 range.

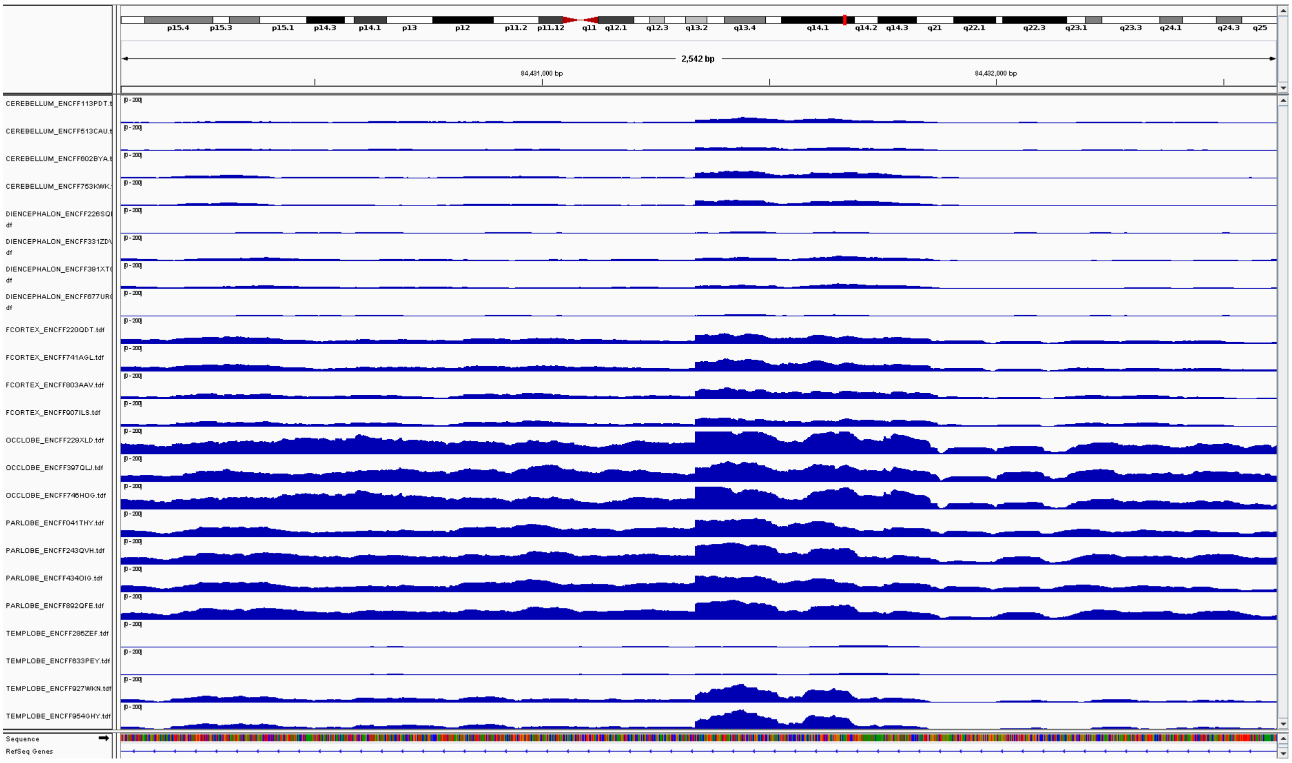

Figure S39: IGV visualization of ENCODE Fetal Brain BAM files coverage in HPin7.

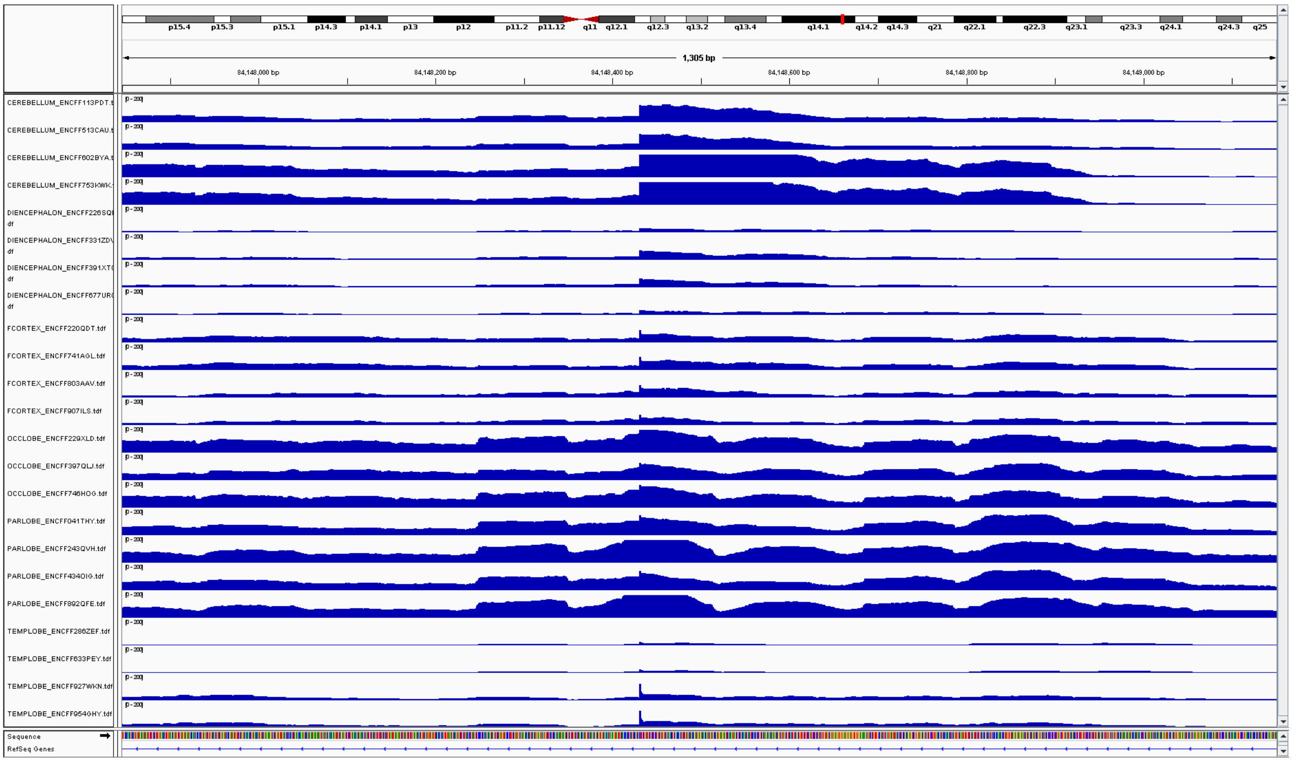

Figure S40: IGV visualization of ENCODE Fetal Brain BAM files coverage in HPin8.

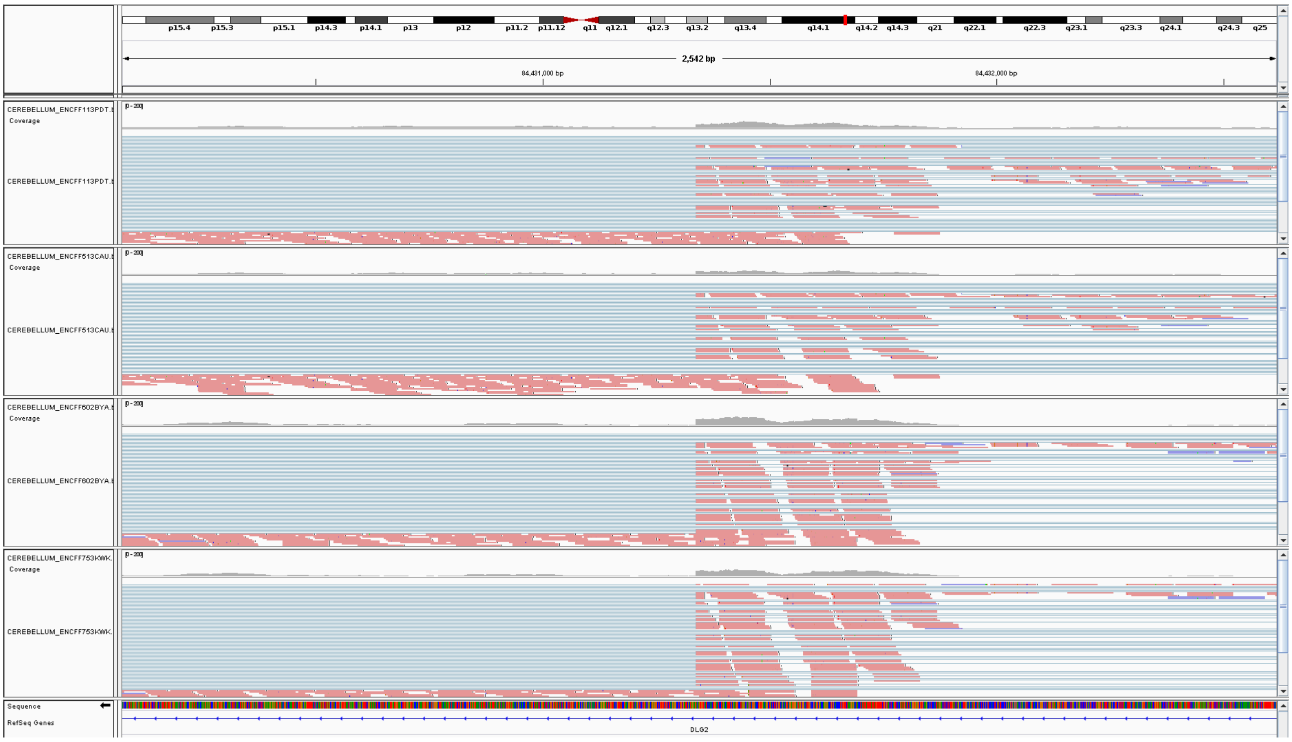

Figure S41: IGV visualization of ENCODE Fetal Brain paired-end reads and coverage in HPin7 (Cerebellum tissue). There is a sharp cut of coverage in the middle of the PRE, with many paired-end reads splitting precisely at chr11:84431338-84431339. See also Figure S43.

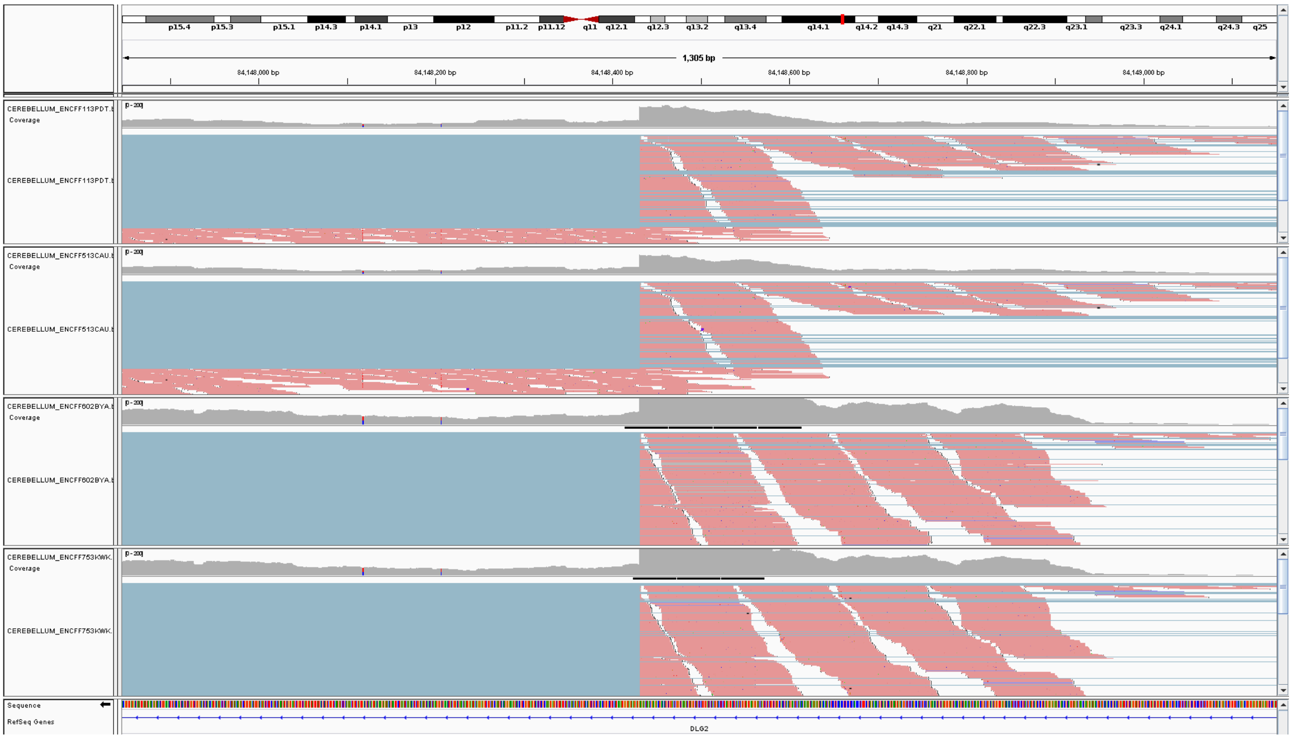

Figure S42: IGV visualization of ENCODE Fetal Brain paired-end reads and coverage in HPin8 (Cerebellum tissue). There is a sharp cut of coverage in the middle of the PRE, with many paired-end reads splitting precisely at chr11:84148430-84148431. See also Figure S44.

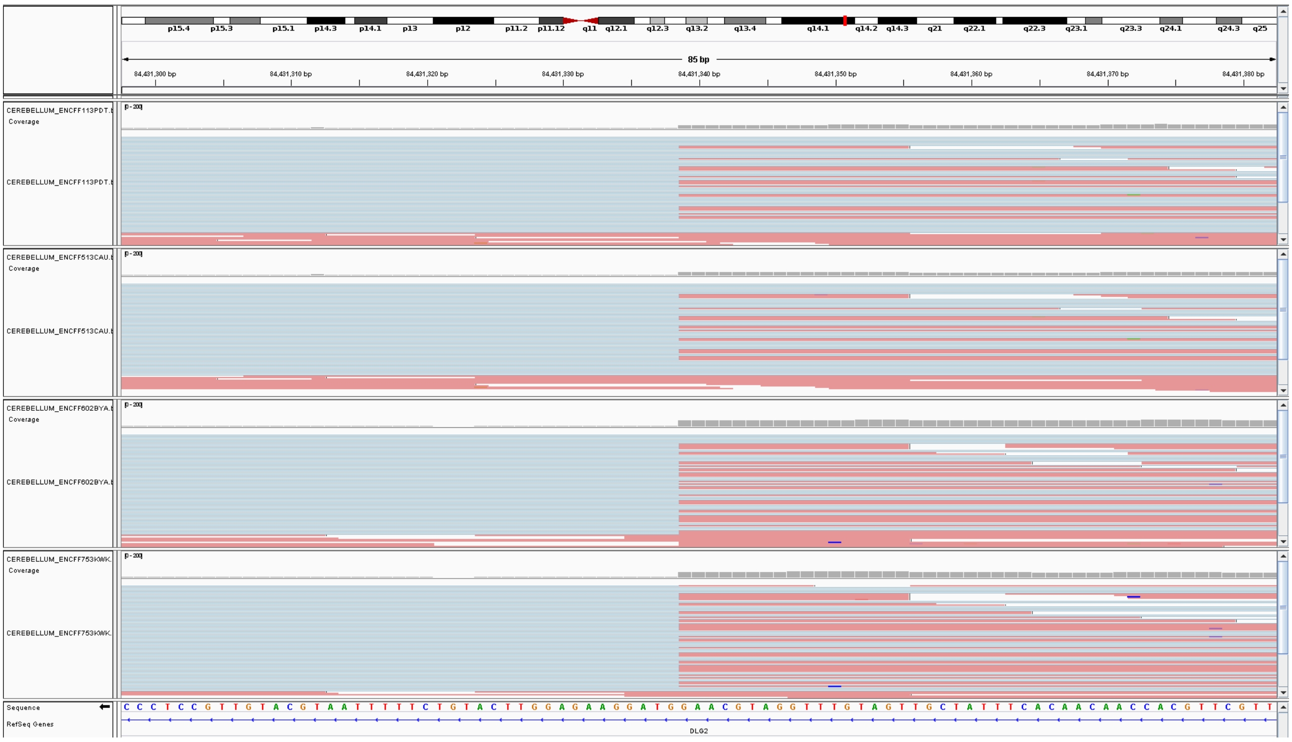

Figure S43: IGV close-up visualization of ENCODE Fetal Brain paired-end reads at the HPin7 splicing site (Cerebellum tissue). In the antisense orientation (corresponding to the *DLG2* gene transcription), the 2bp before and after the splicing site are *AG* and *GT* respectively, agreeing with the splicing consensus sequences studied in [4, 5]. The HPin7 reads splice into *DLG2* exon 8 (see Figure S54).

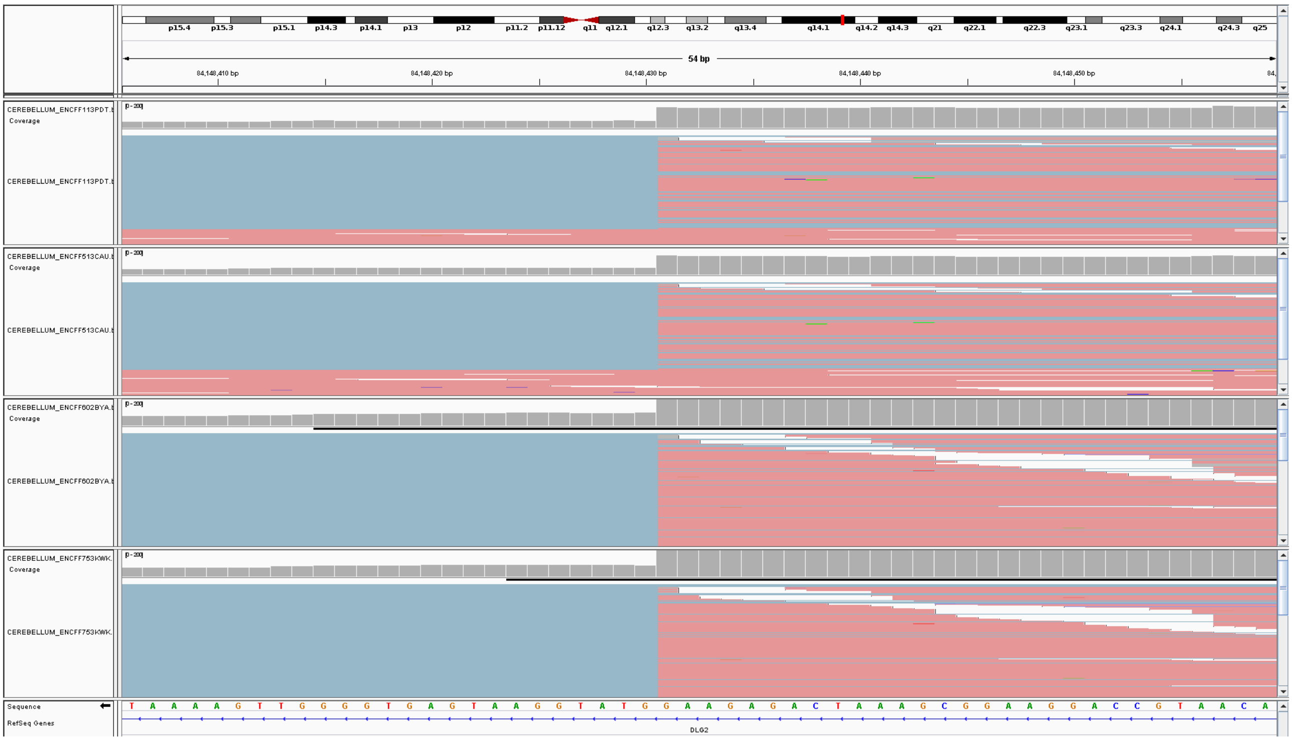

Figure S44: IGV close-up visualization of ENCODE Fetal Brain paired-end reads at the HPin8 splicing site (Cerebellum tissue). In the antisense orientation (corresponding to the *DLG2* gene transcription), the 2bp before and after the splicing site are *AG* and *GT*, respectively, agreeing with the splicing consensus sequences studied in [4, 5]. The HPin8 reads splice into *DLG2* exon 11 (see Figure S54).

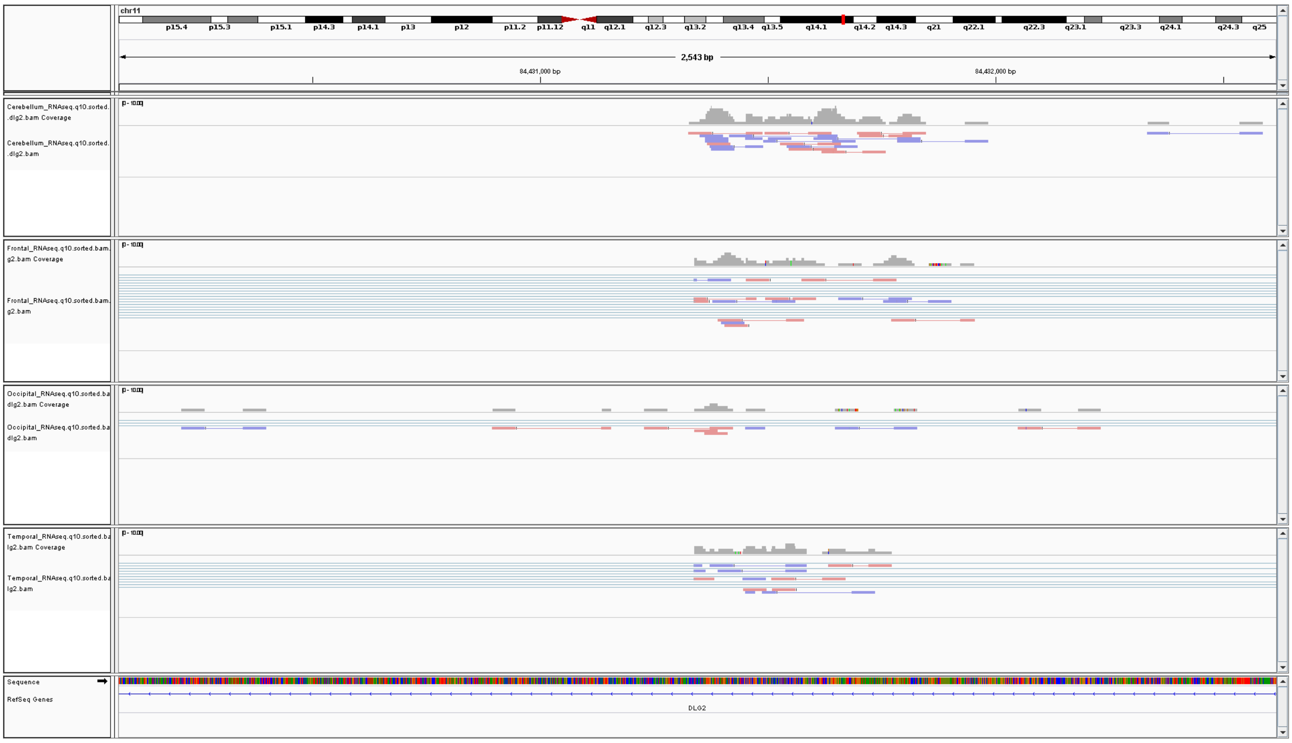

Figure S45: IGV visualization of Adult Brain BAM reads in HPin7. The y-scales of all tracks have been manually set and fix to the 0-10 range.

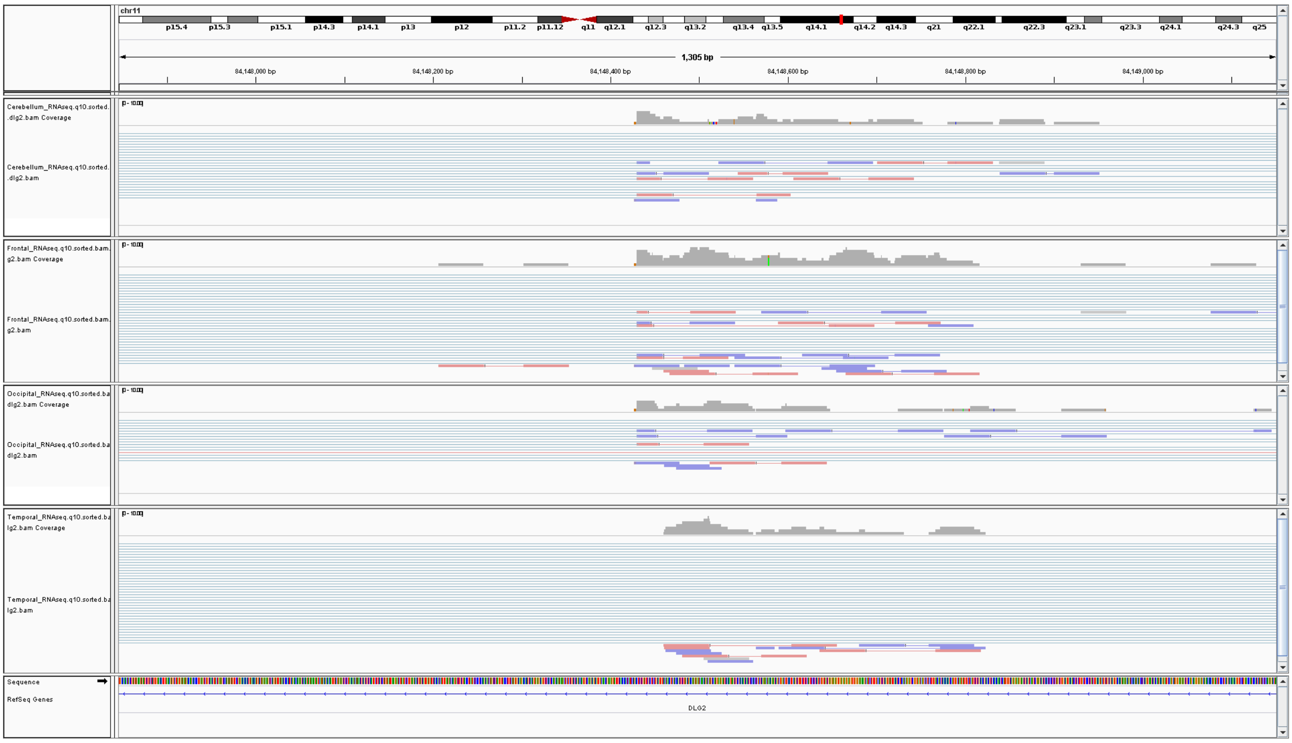

Figure S46: IGV visualization of Adult Brain BAM reads in HPin8. The y-scales of all tracks have been manually set and fix to the 0-10 range.

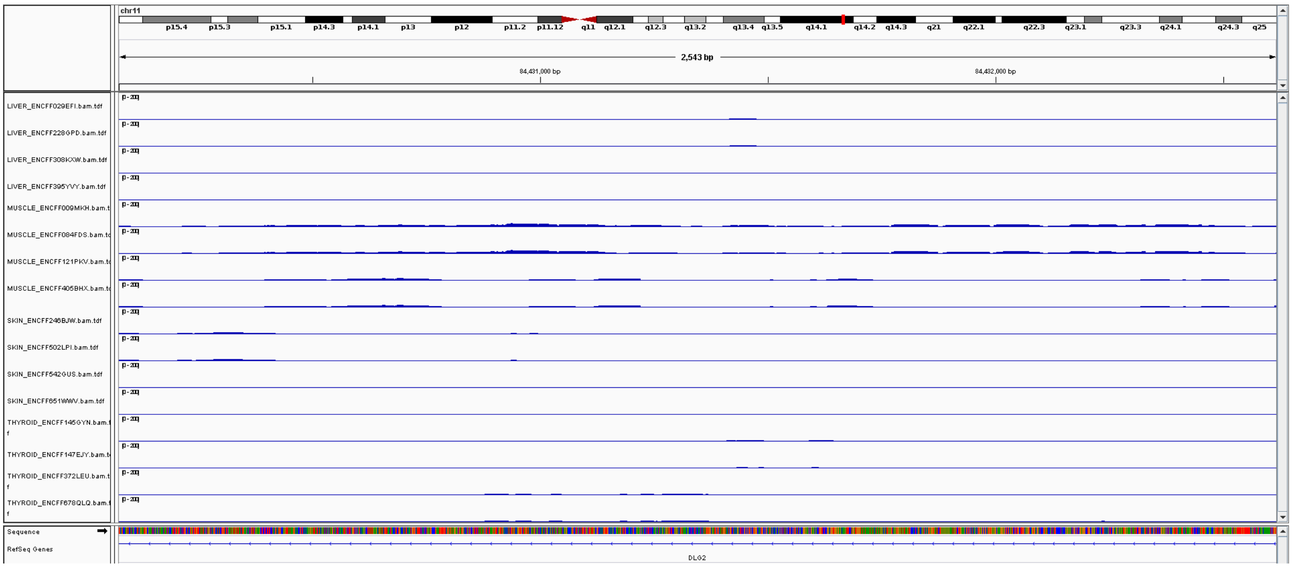

Figure S47: IGV visualization of ENCODE Fetal non-Brain BAM coverage in HPin7. The y-scales of all tracks have been manually set and fix to the 0-200 range.

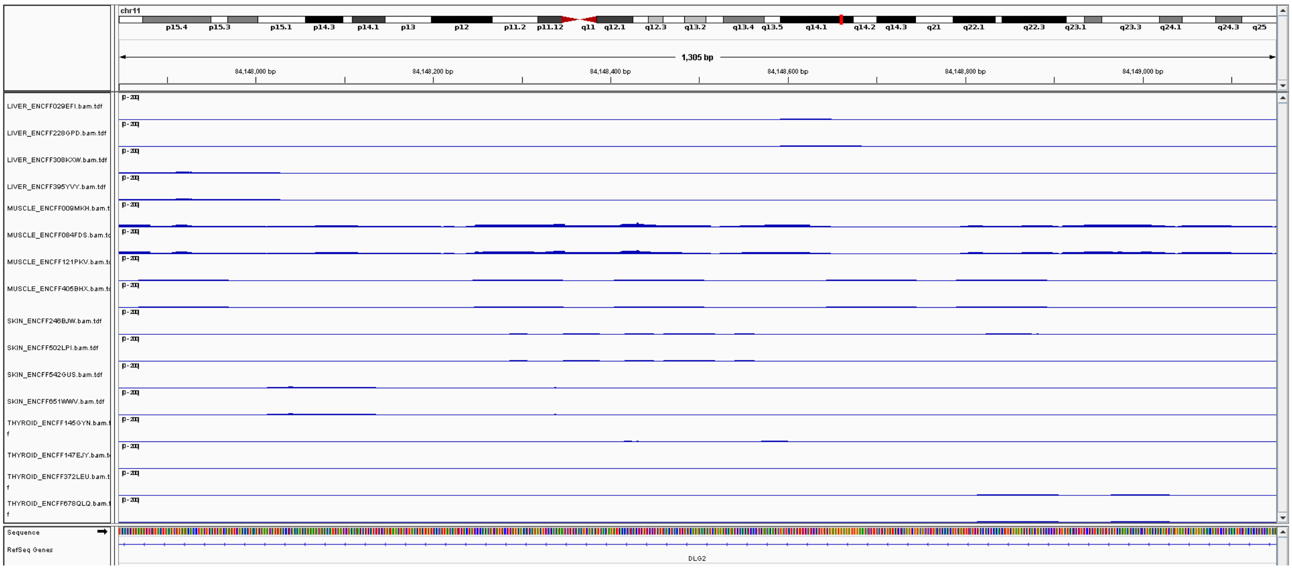

Figure S48: IGV visualization of ENCODE Fetal non-Brain BAM coverage in HPin8. The y-scales of all tracks have been manually set and fix to the 0-200 range.

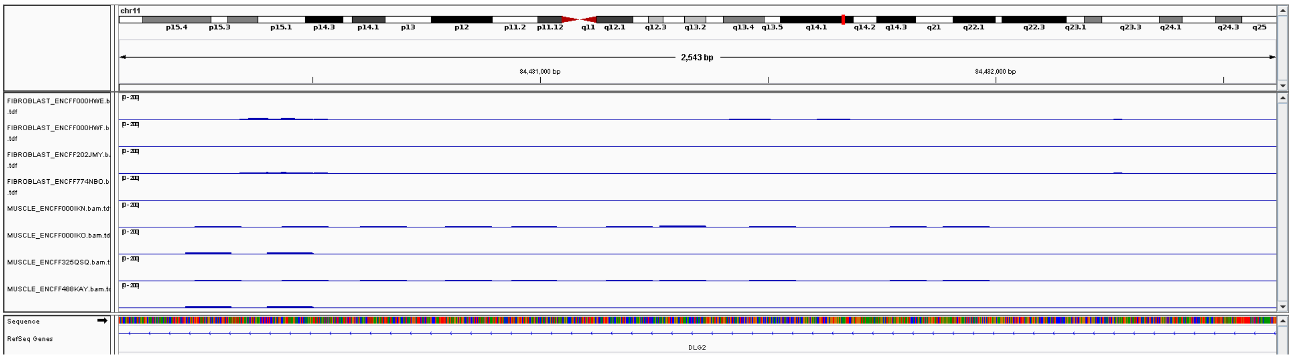

Figure S49: IGV visualization of ENCODE Adult non-Brain BAM coverage in HPin7. The y-scales of all tracks have been manually set and fix to the 0-200 range.

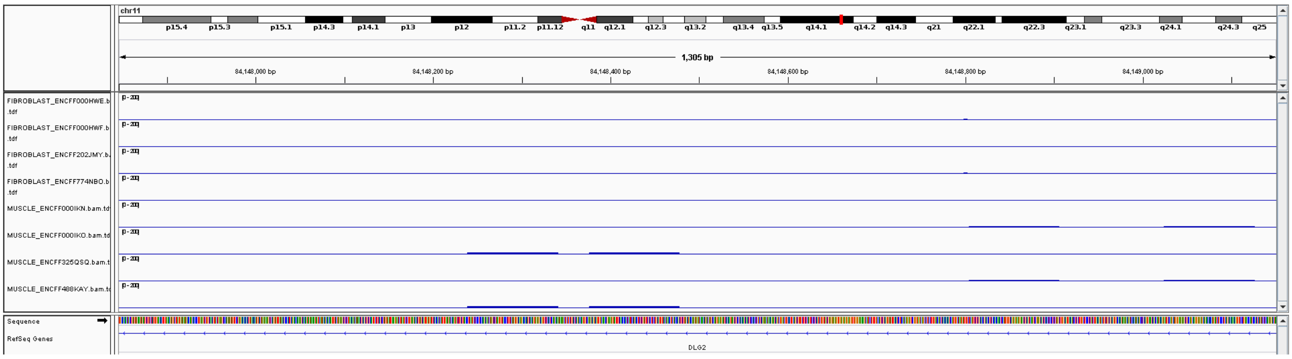

Figure S50: IGV visualization of ENCODE Adult non-Brain BAM coverage in HPin8. The y-scales of all tracks have been manually set and fix to the 0-200 range.

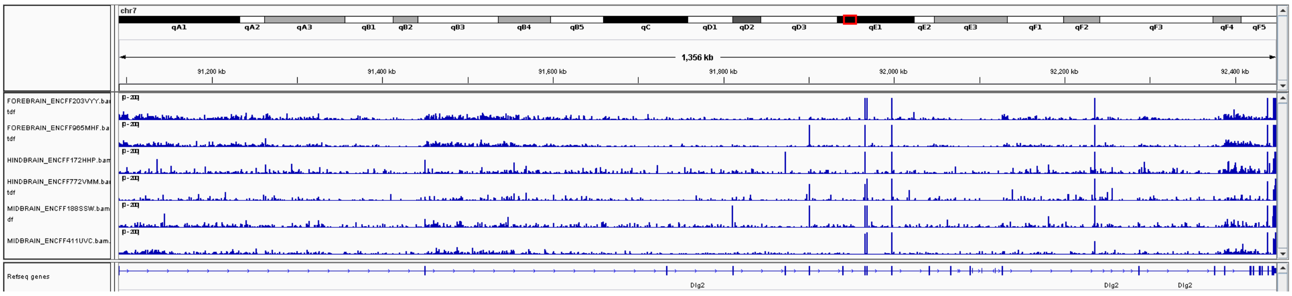

Figure S51: IGV visualization of ENCODE mouse newborn BAM coverage in *Dlg2*. The y-scales of all tracks have been manually set and fix to the 0-200 range.

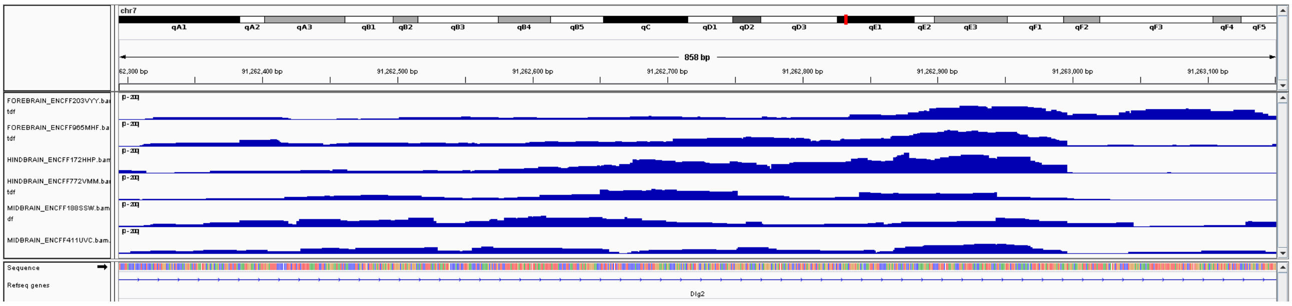

Figure S52: IGV visualization of ENCODE mouse newborn BAM coverage in mHPin1. The y-scales of all tracks have been manually set and fix to the 0-200 range. The splicing site is visible at location chr7:91262995-91262996

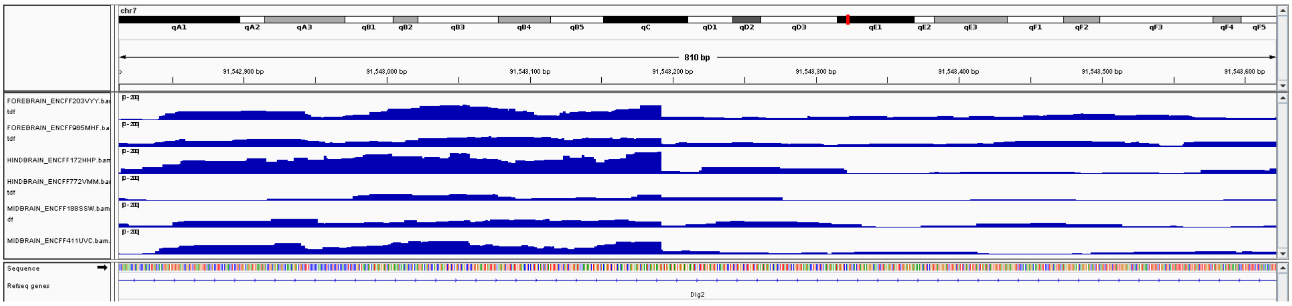

Figure S53: IGV visualization of ENCODE mouse newborn BAM coverage in mHPin1. The y-scales of all tracks have been manually set and fix to the 0-200 range. The splicing site is visible at location chr7:91543191-91543192

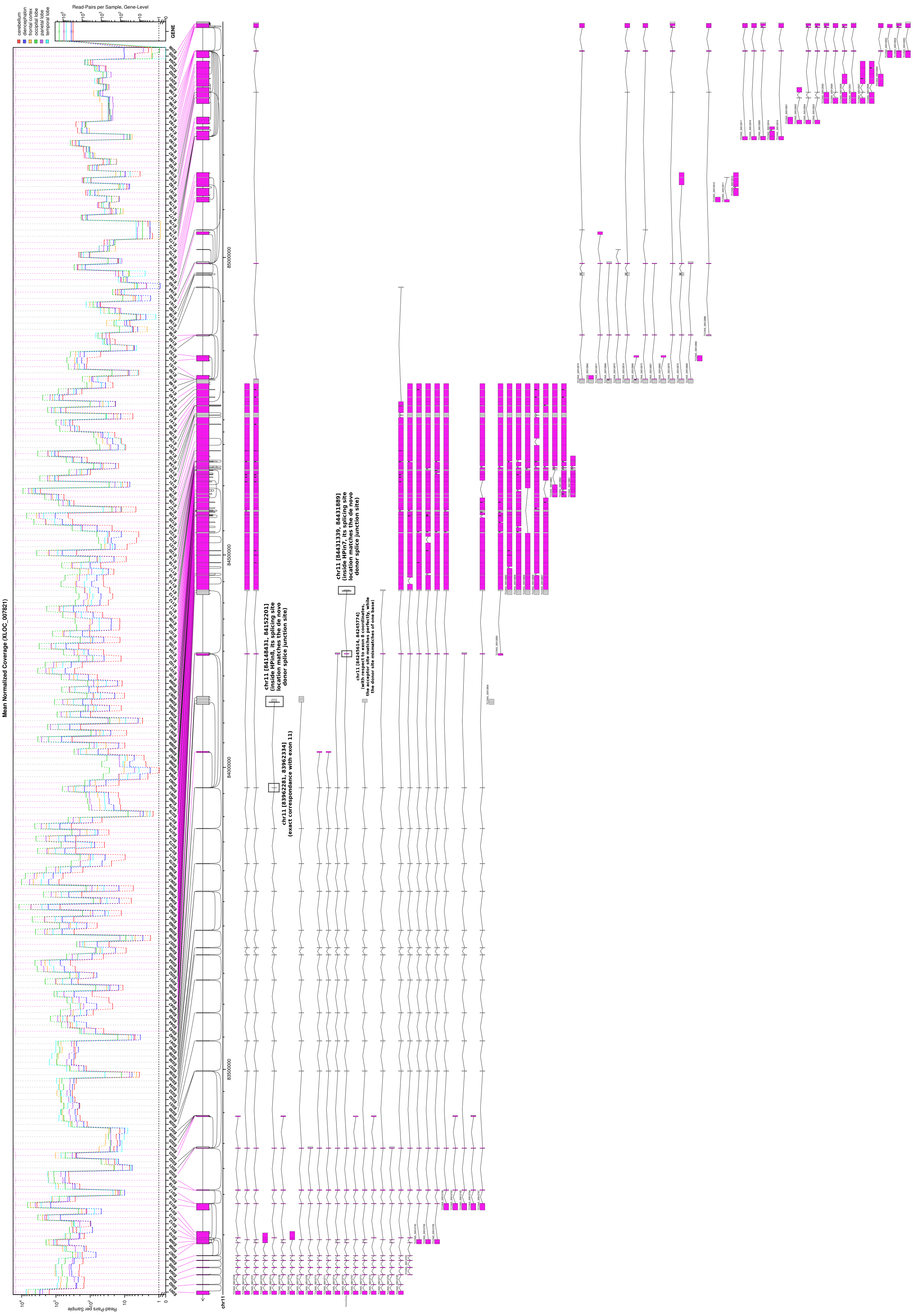

Figure S54: Fetal brain de novo transcriptome assembly of *DLG2*. (Continued on the following page.)

Figure S54: *cuffmerge* min-isoform-fraction parameter set at 0.05. Visualization realized with *JunctionSeq*. Annotation of HPin7 and HPin8 isoforms and coordinates added to the figure (please zoom on *DLG2* 7-9 region for reading). The figure is composed by three panels: the top-panel reports the expression of "exon" (in cufflinks de-novo transcriptome mode, exons are any part of the genome that has been transcribed); the middle panel show genomic coordinates along with unique exons aligned in a row; the bottom panel pictures the predicted isoforms. Some remarks or other useful information to interpret the image are the following (For more details, please see JunctionSeq documentation): a) in middle and bottom panels exons (boxes) are connected through edges, which stands for splicing events; b) in the middle panel, overlapping transcribed regions (detected in different isoforms) are merged and the original boundaries reported as dashed vertical lines, and the boundaries of the merged exons is a solid line; c) in the middle panel, oblique segments at the bottom of exons represent the starting or ending point of isoforms; d) in the middle panel the genomic coordinates are not linear (for visualization purposes). Coordinates of the merged transcribed genomic regions pictured in the middle panel are reported in Table S14; e) in any isoform, exons are plot with solid lines. Vertical dashed lines inside exons represent starting or ending points of exons belonging to other isoforms; f) exons might be colored with purple or gray, in the first case the expression between any two cohorts is significant, while in the second case it is not significant, but for our purposes this exons annotation is not interesting. Some consideration of figure are the following: a) the de novo (so without reference) transcriptome assembly performed by cufflinks starting with paired-end RNA-Seq data of fetal brain tissue finds the *DLG2* gene, from Table S14 the coordinate is chr11:83166048-85339790 (RefSeq *DLG2* is chr11:83166056-85338314); b) Tables S15 and S16 report the de novo transcriptome coordinates that overlap *DLG2* UCSC exons (Table S1) and *DLG2* Ensembl exons (Table S2), respectively. De Novo (DN) *DLG2* overlaps 76.28% of *DLG2* UCSC (exon 10 is completely missing) and 75.04% of *DLG2* Ensembl (exons 3, 16, 17, 19, 30, 31, 33, 34 are completely missing); c) DN *DLG2* exon number 110 (chr11:84146499-84155193) includes HPin8 (chr11:84147846-84149151). The isoforms starting from HPin8 splice into *DLG2* exon 11; d) DN *DLG2* exon number 57 (chr11:84425769-84827382) includes HPin7 (chr11:84430074-84432618). The isoforms starting from HPin7 splice into *DLG2* exon 8; e) DN *DLG2* exon number 57 (DNe57) is 401613 base-pairs (bp) wide, very far away from the 170bp reported in [6]. At the edges of this region are located HPin7 and UCSC exon 6. DNe57 is still present in several isoforms which level of abundance is above 0.50 (not shown). This de novo predicted abnormal long exon (from exon 6 to HPin7) is probably due to the difficulty for cufflinks to deal with nascent transcription known to be present in brain mRNAs [7].

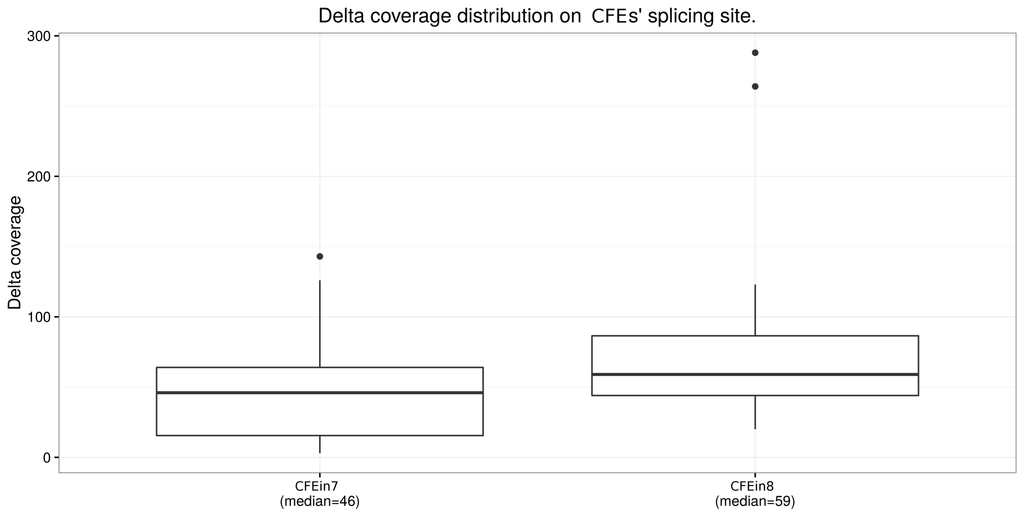

Figure S55: CFEin7 and CFEin8 delta coverage distribution via boxplot representation.

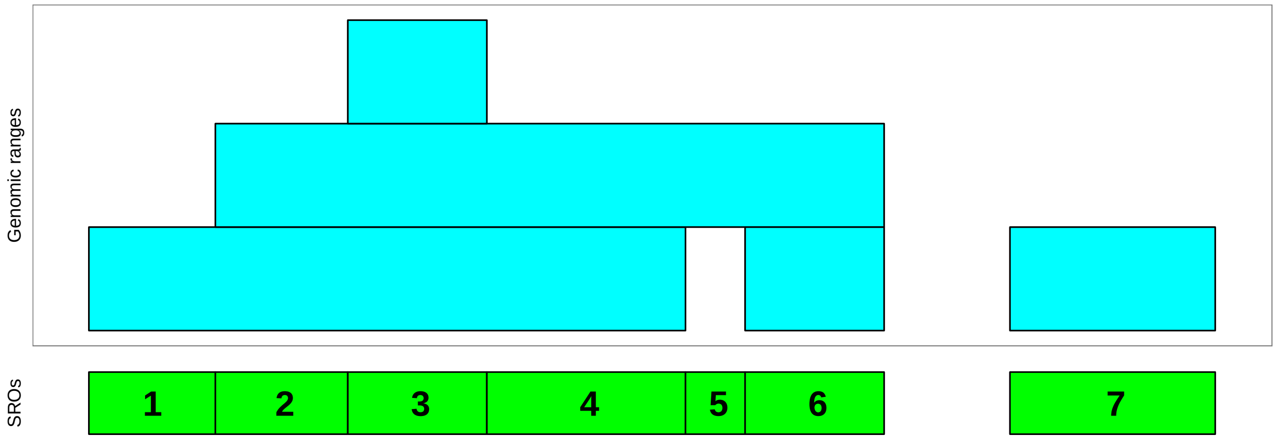

Figure S56: Example of Smallest Regions of Overlap (SROs) from a set of Genomic Ranges (GR). We define the Smallest Regions of Overlap (SROs) of a set of Genomic Ranges (GR) as their disjoint ranges. By definition then, SROs do not overlap each other and SRO's coverage equals the GR's one. In R programming language, the *disjoin* function of *GenomicRanges* package returns the SROs from a collection of GR. In this example, 7 SROs (in green) are created from 5 GR.

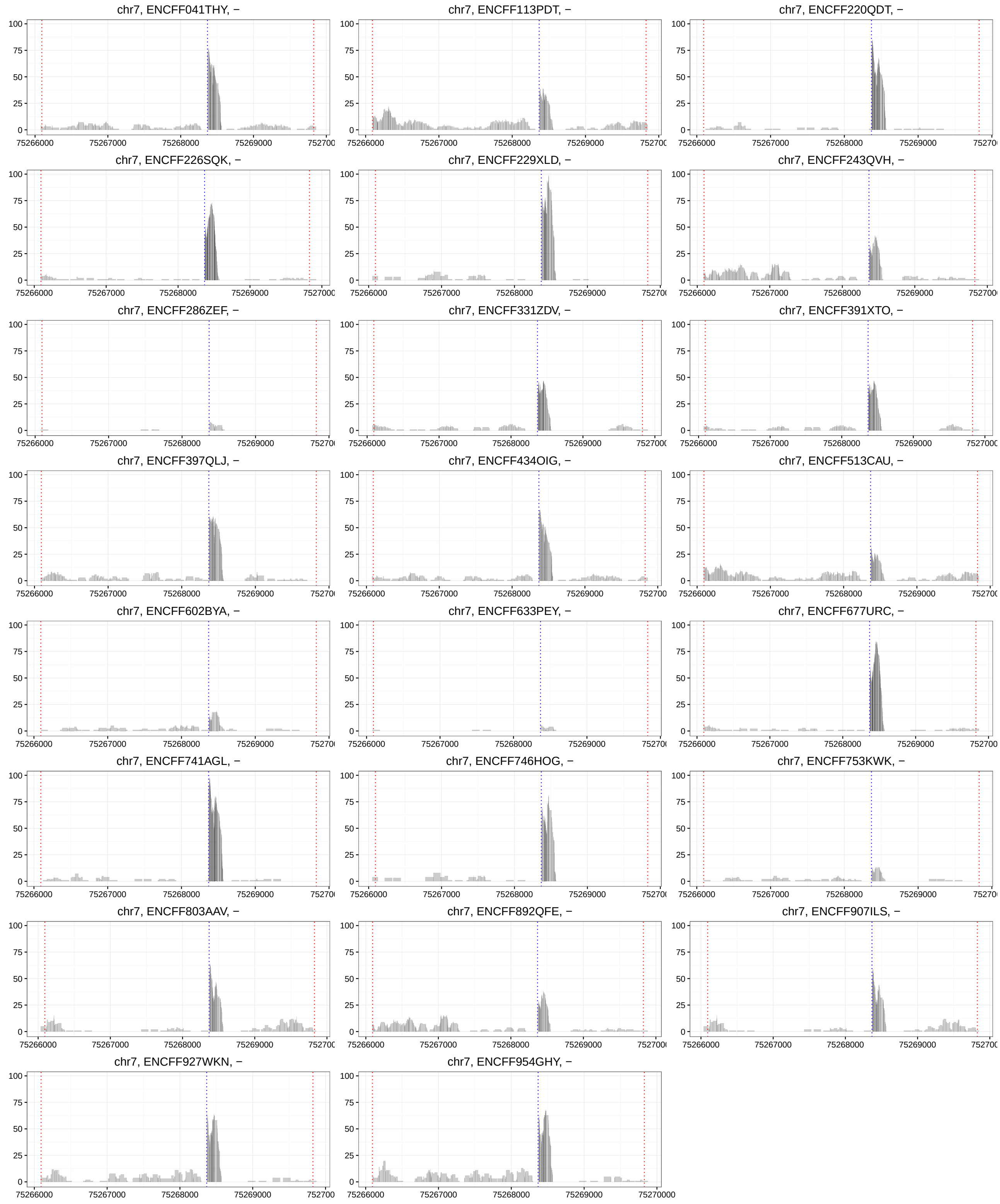

Figure S57: Read coverage in 23 fetal brain BAM files regarding *HIP1* gene at chr7:75266093-75269827. Red lines define the SRO region, while the blue one pinpoint the splicing site. The title of every subplot provides three information: chromosome location, BAM file name, and gene strand.

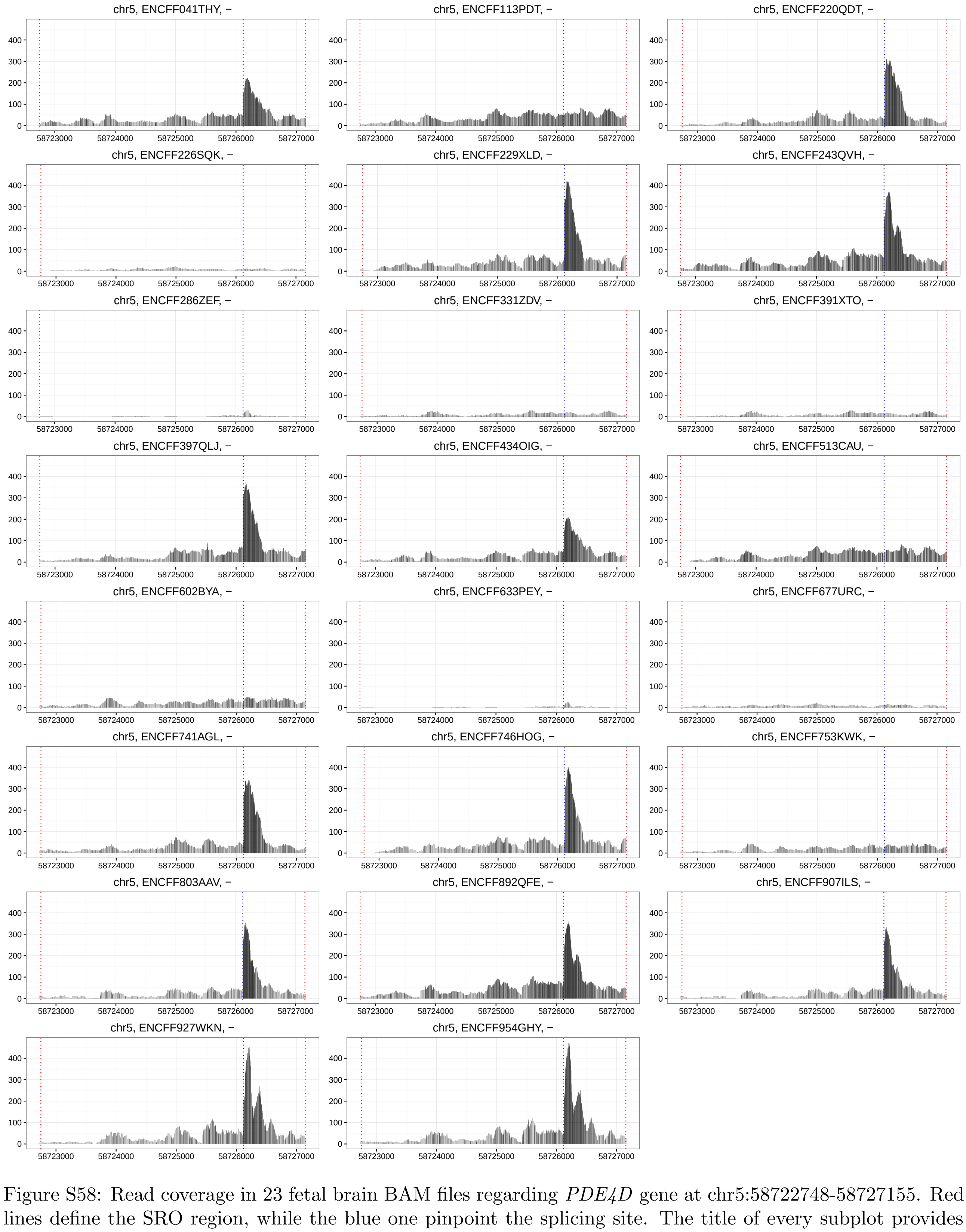

Figure S58: Read coverage in 23 fetal brain BAM files regarding *PDE4D* gene at chr5:58722748-58727155. Red lines define the SRO region, while the blue one pinpoint the splicing site. The title of every subplot provides three information: chromosome location, BAM file name, and gene strand.

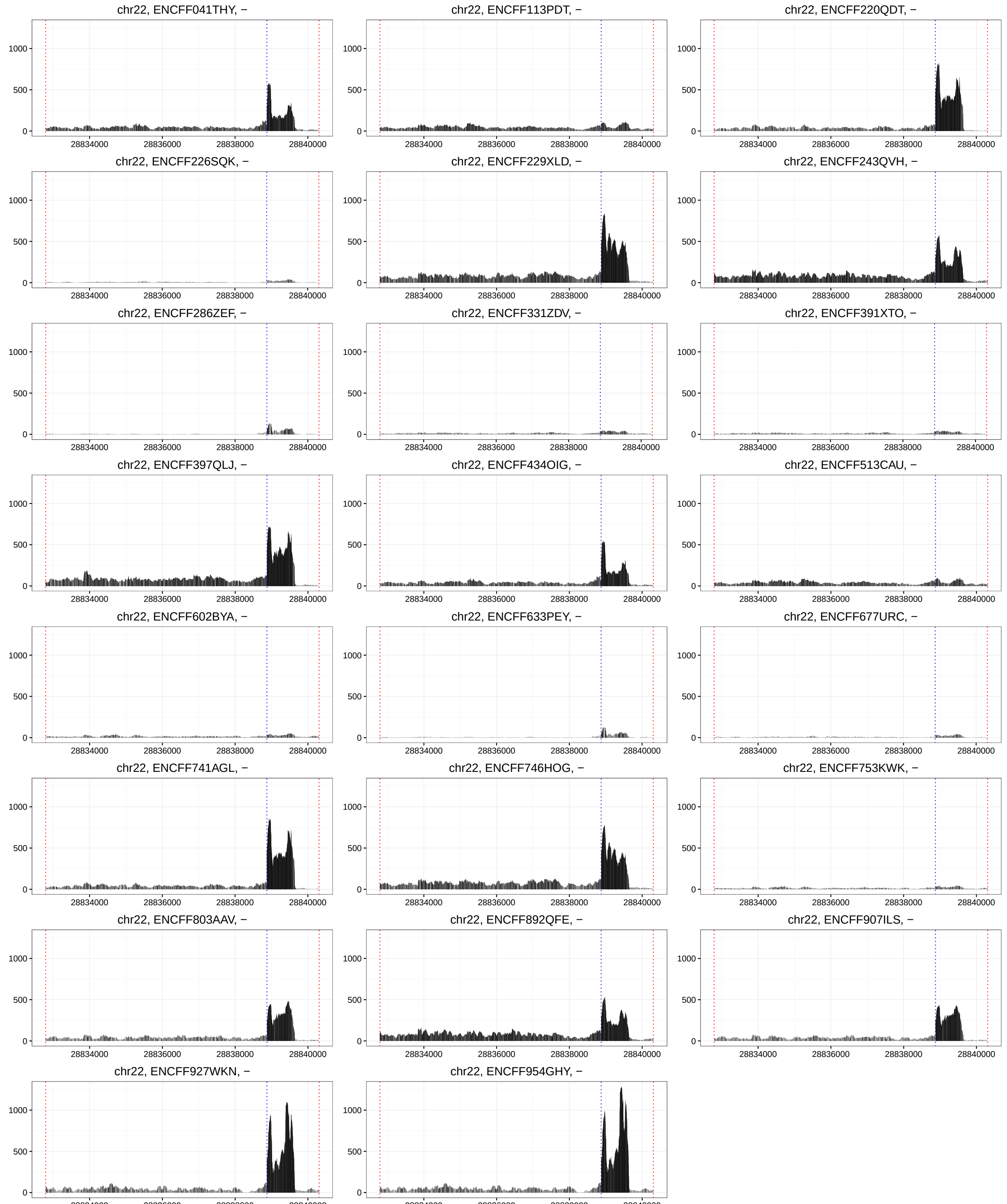

Figure S59: Read coverage in 23 fetal brain BAM files regarding *TTC28* gene at chr22:28832791-28840308. Red lines define the SRO region, while the blue one pinpoint the splicing site. The title of every subplot provides three information: chromosome location, BAM file name, and gene strand.

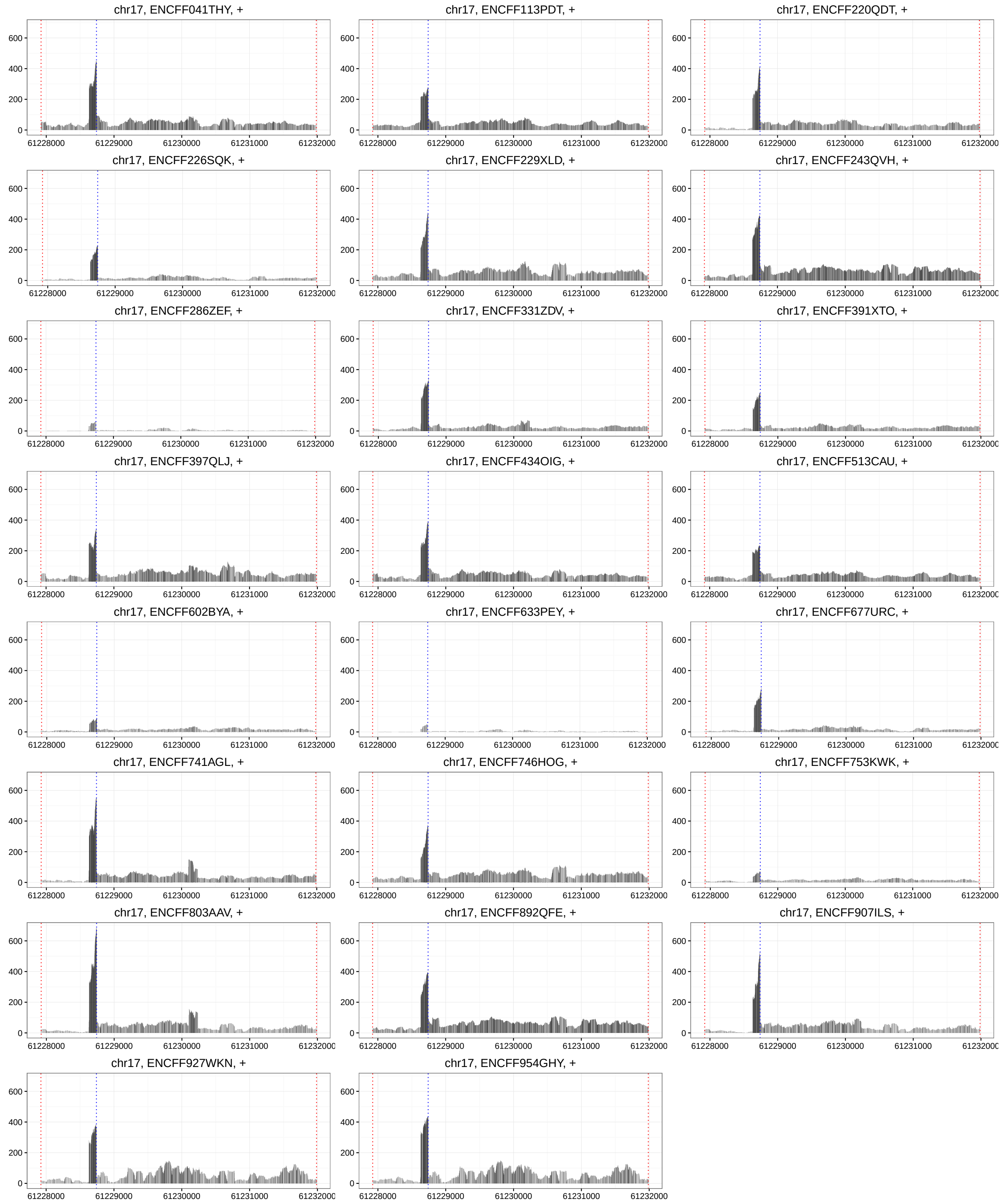

Figure S60: Read coverage in 23 fetal brain BAM files regarding *TANC2* gene at chr17:61227923-61231987. Red lines define the SRO region, while the blue one pinpoint the splicing site. The title of every subplot provides three information: chromosome location, BAM file name, and gene strand.

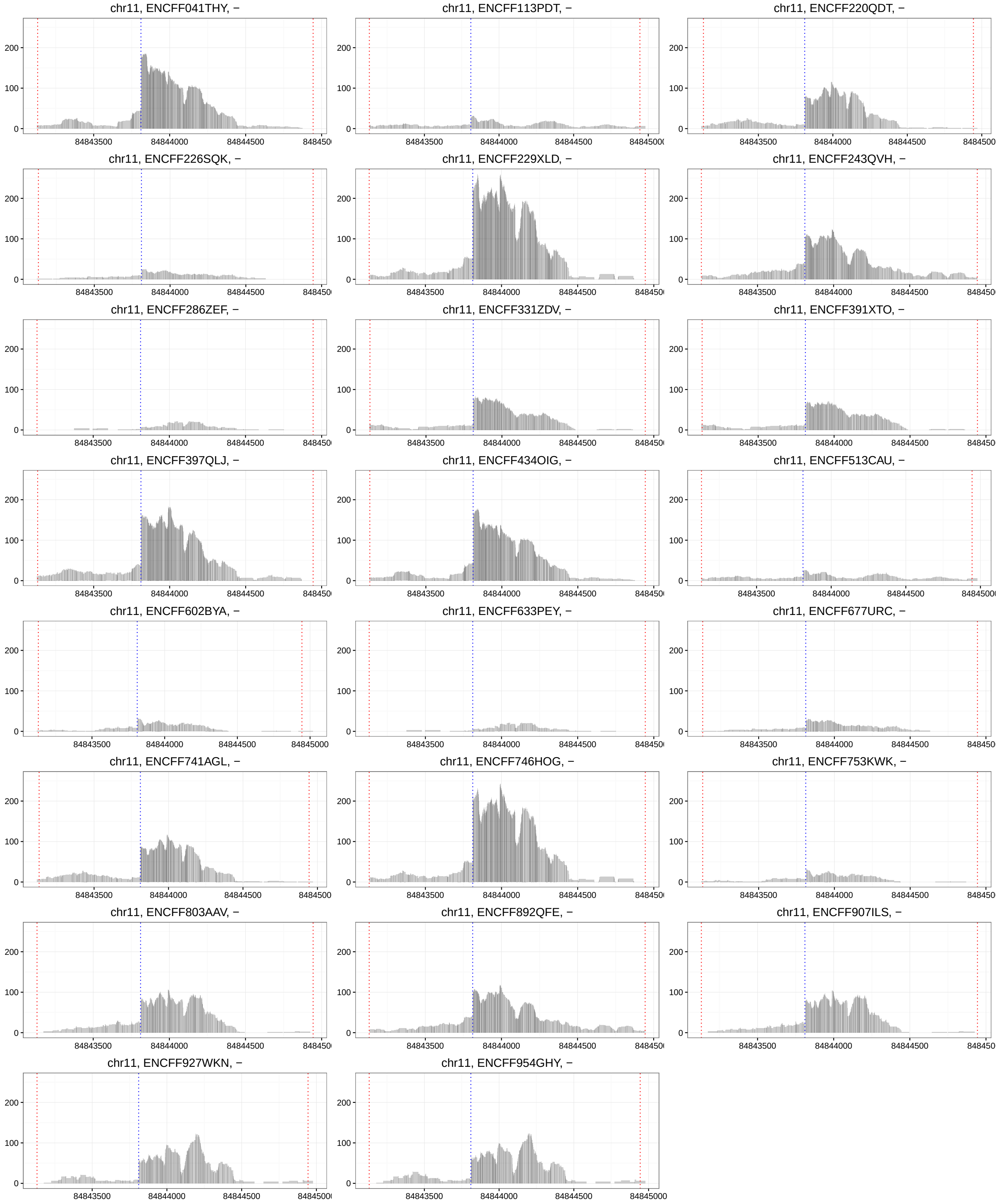

Figure S61: Read coverage in 23 fetal brain BAM files regarding *DLG2* gene at chr11:84843131-84844944. Red lines define the SRO region, while the blue one pinpoint the splicing site. The title of every subplot provides three information: chromosome location, BAM file name, and gene strand.

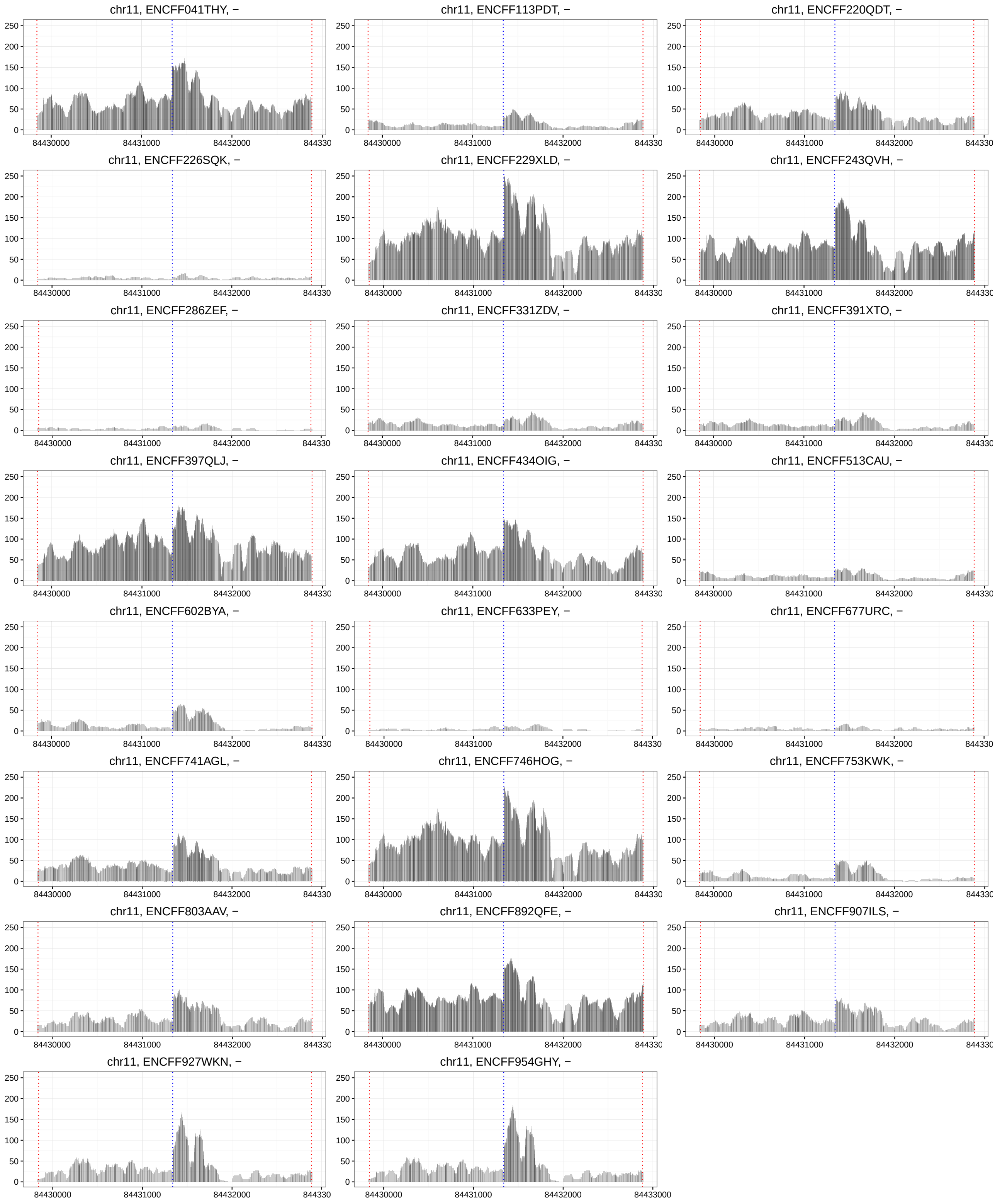

Figure S62: Read coverage in 23 fetal brain BAM files regarding *DLG2* gene at chr11:84429842-84432885. Red lines define the SRO region, while the blue one pinpoint the splicing site. The title of every subplot provides three information: chromosome location, BAM file name, and gene strand.

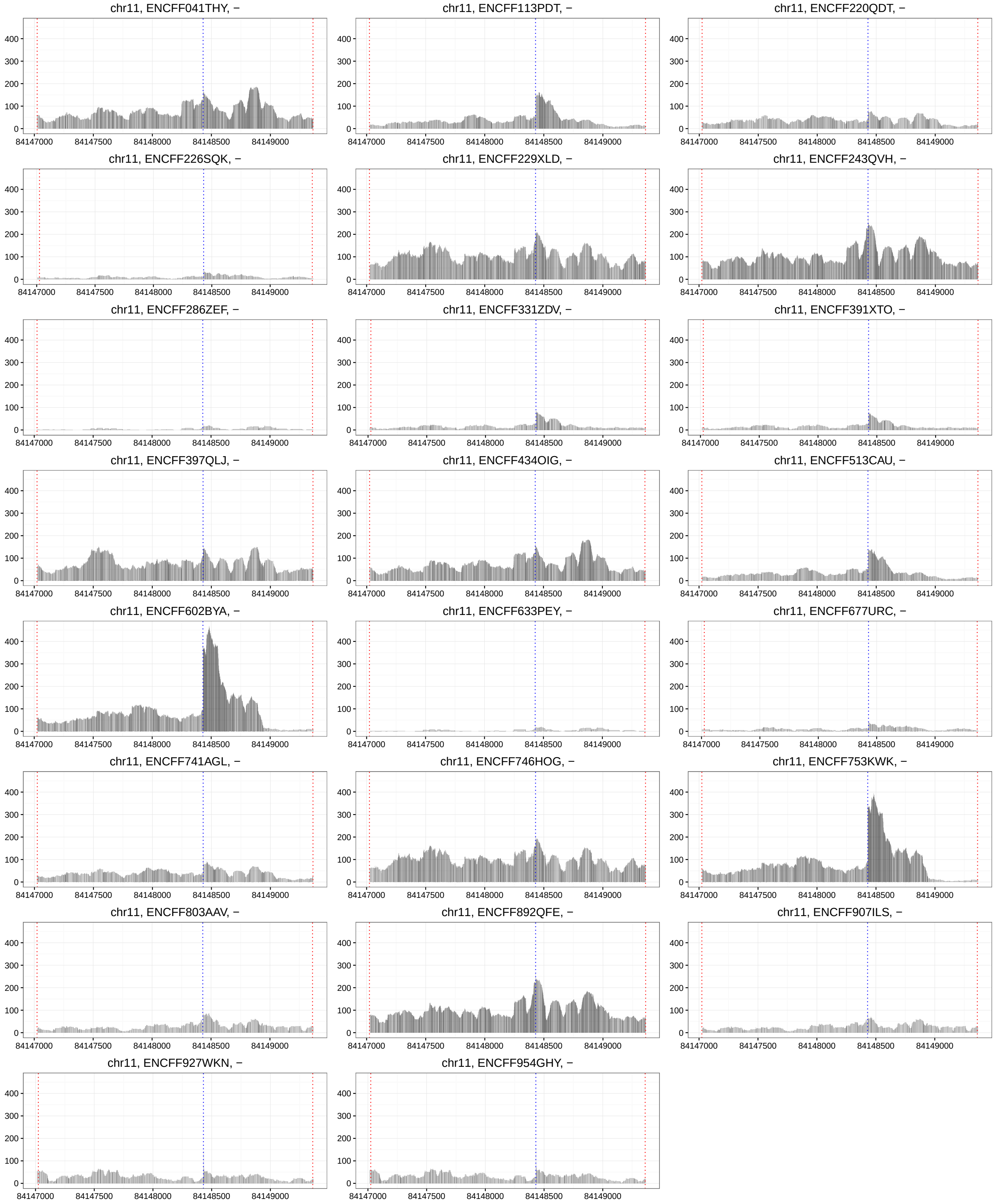

Figure S63: Read coverage in 23 fetal brain BAM files regarding *DLG2* gene at chr11:84147024-84149361. Red lines define the SRO region, while the blue one pinpoint the splicing site. The title of every subplot provides three information: chromosome location, BAM file name, and gene strand.

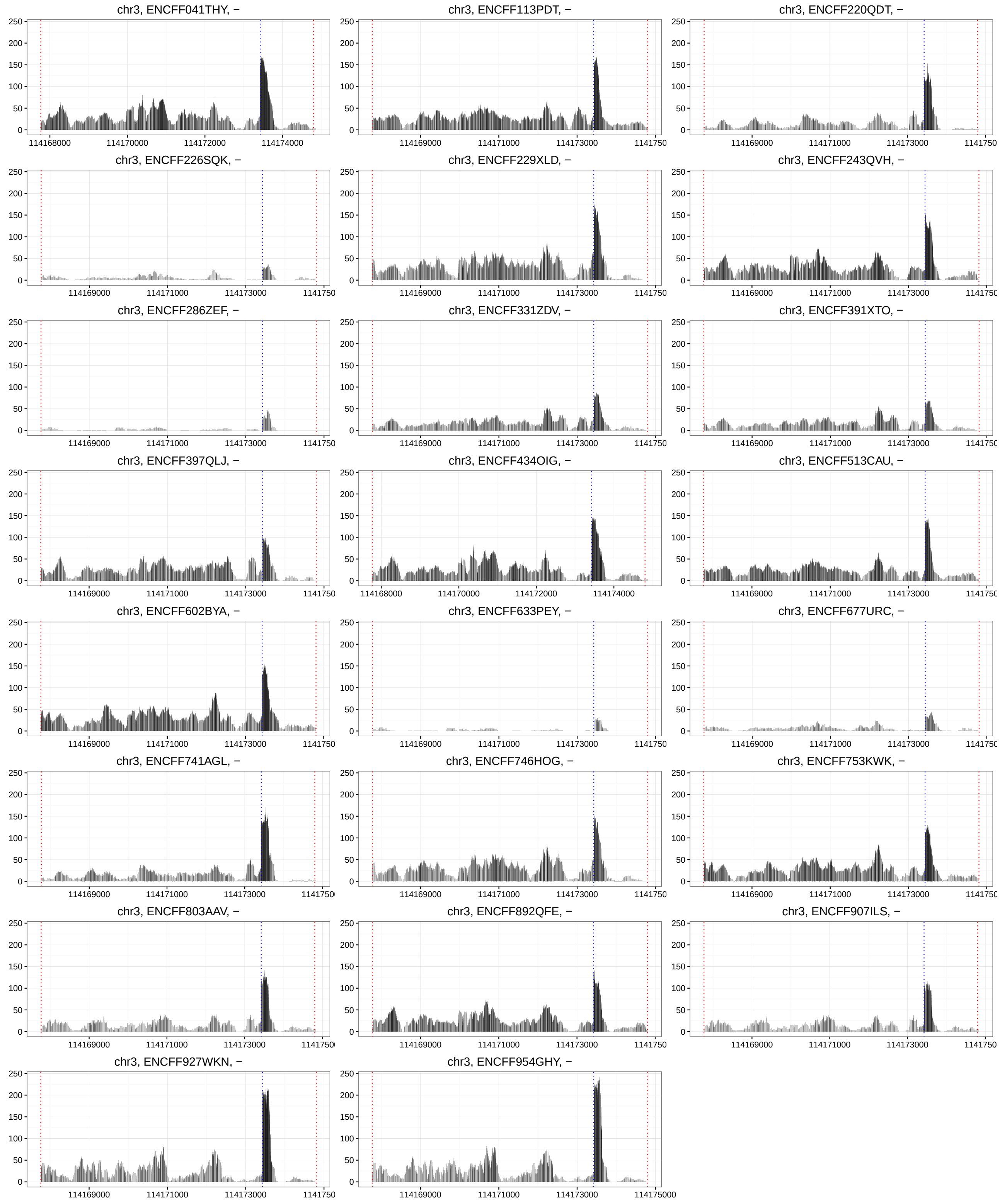

Figure S64: Read coverage in 23 fetal brain BAM files regarding *ZBTB20* gene at chr3:114167766-114174803. Red lines define the SRO region, while the blue one pinpoint the splicing site. The title of every subplot provides three information: chromosome location, BAM file name, and gene strand.

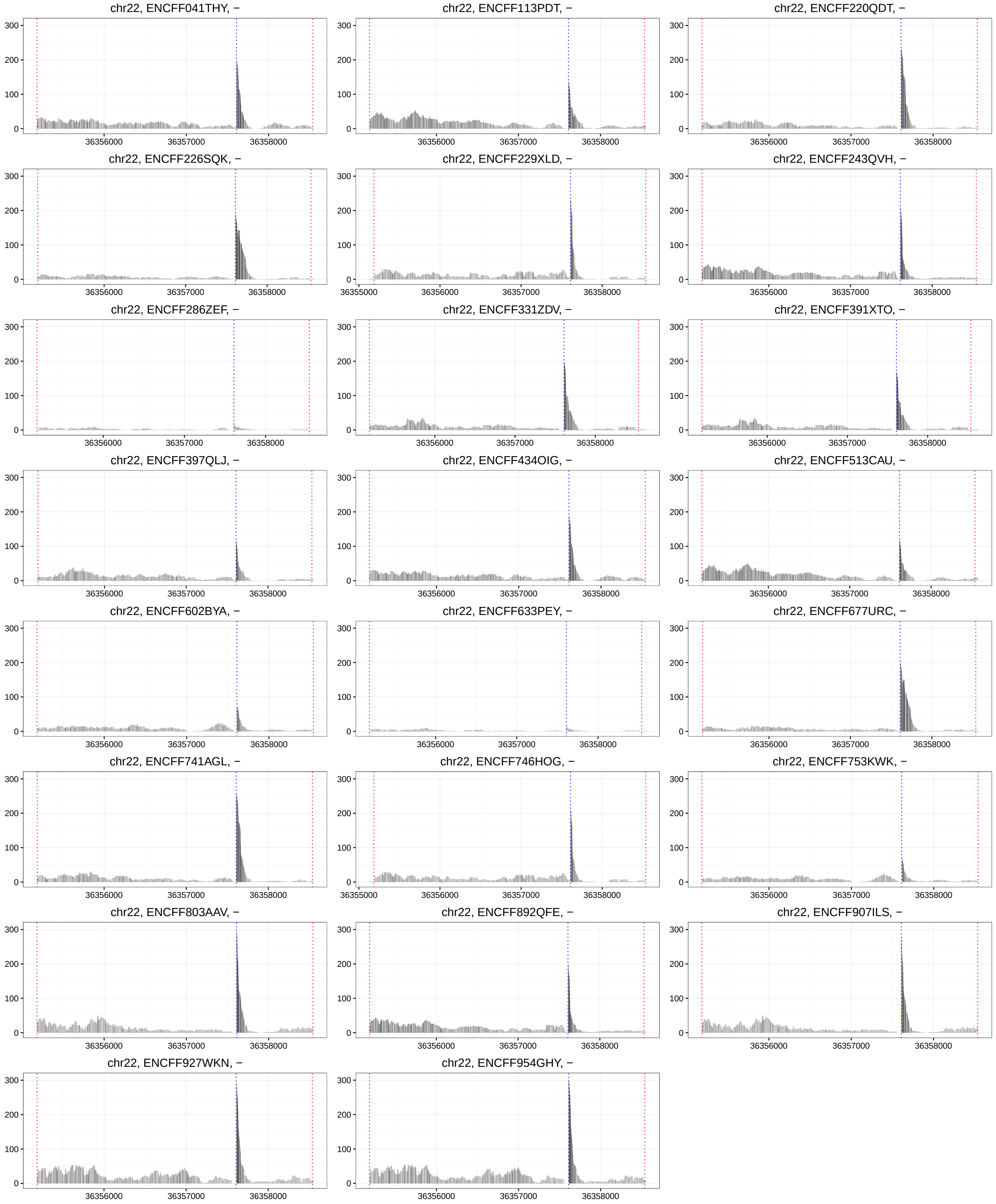

Figure S65: Read coverage in 23 fetal brain BAM files regarding *RBFOX2* gene at chr22:36355185-36358538. Red lines define the SRO region, while the blue one pinpoint the splicing site. The title of every subplot provides three information: chromosome location, BAM file name, and gene strand.

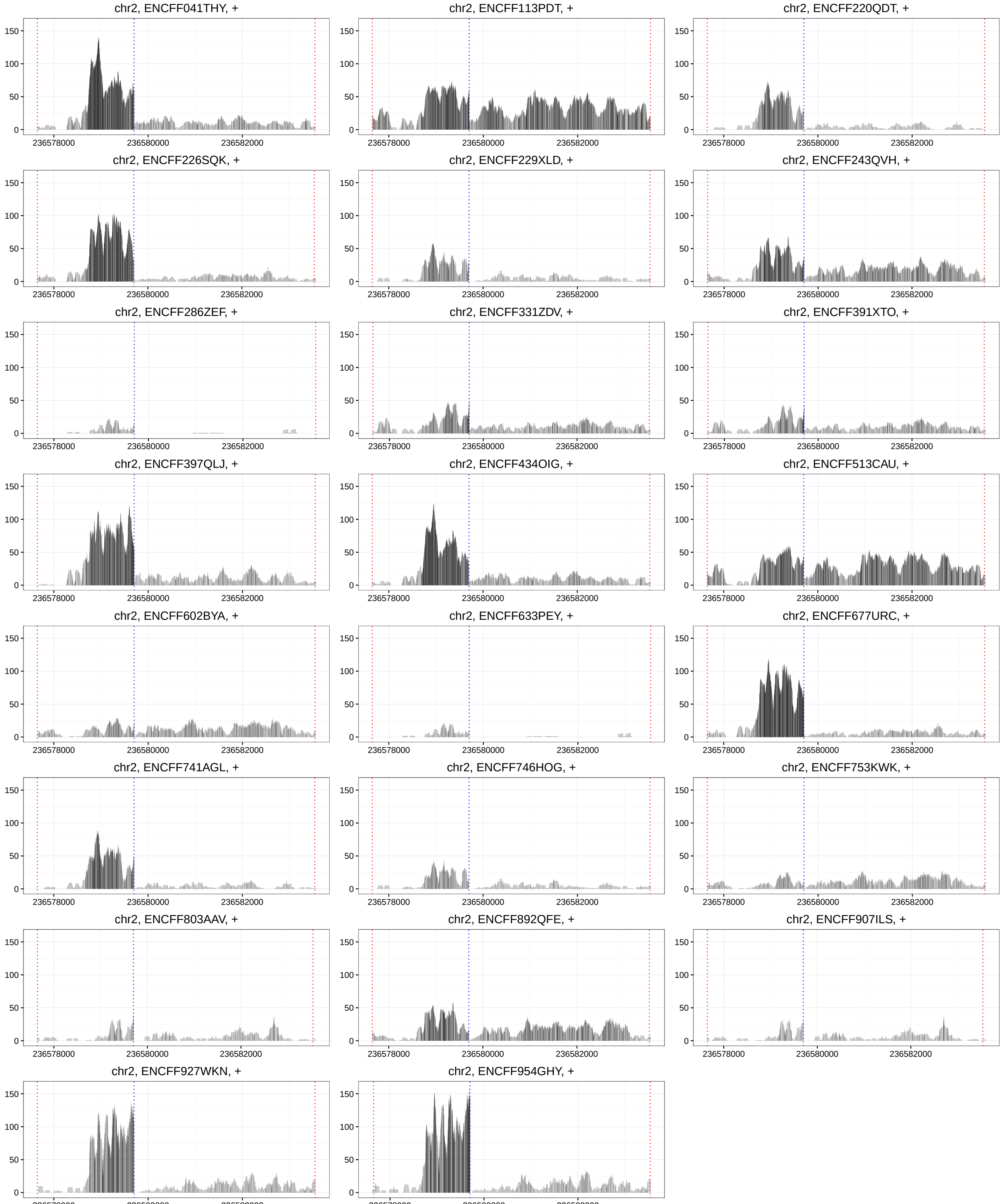

Figure S66: Read coverage in 23 fetal brain BAM files regarding *AGAP1* gene at chr2:236577649-236583540. Red lines define the SRO region, while the blue one pinpoint the splicing site. The title of every subplot provides three information: chromosome location, BAM file name, and gene strand.

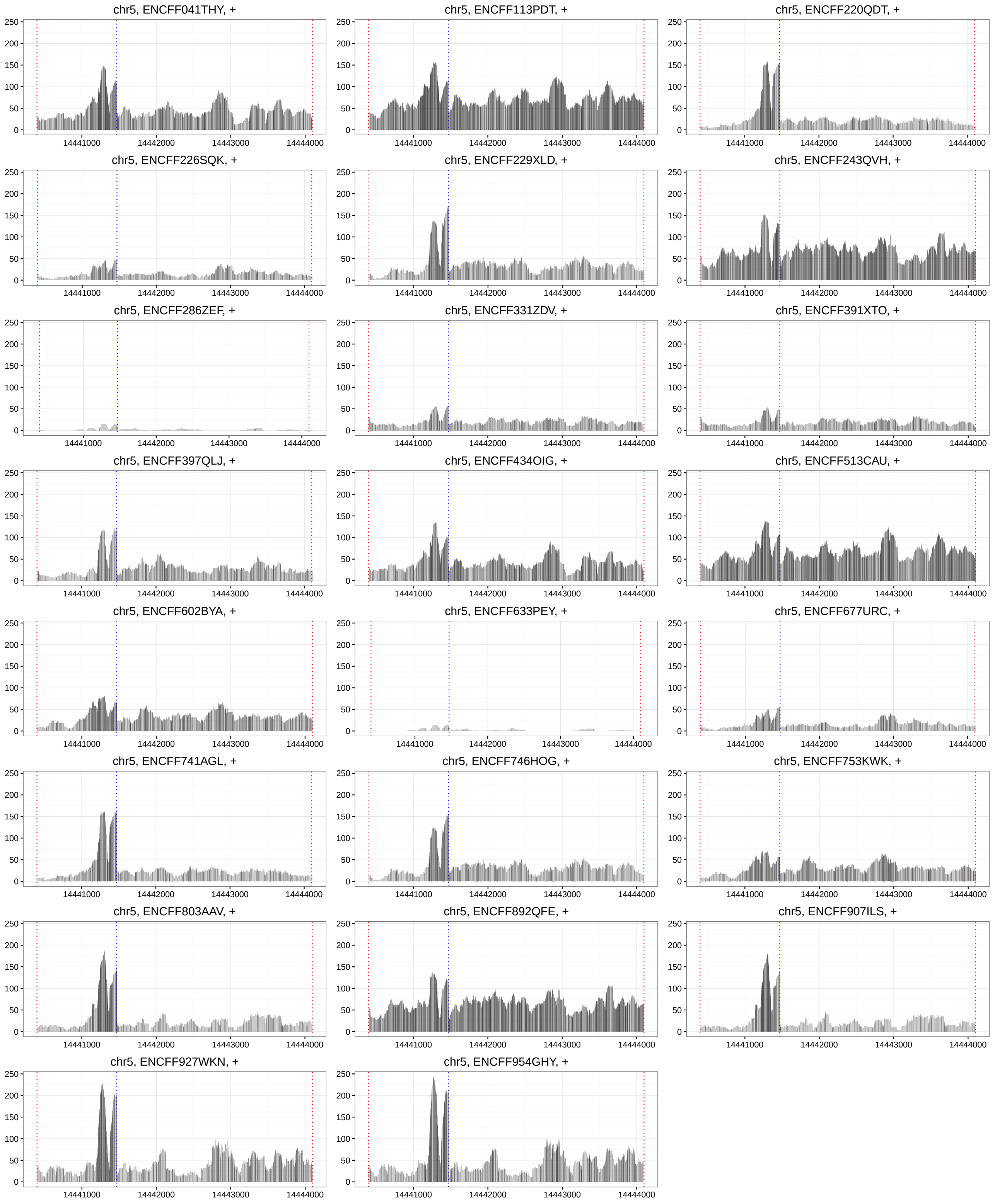

Figure S67: Read coverage in 23 fetal brain BAM files regarding *TRIO* gene at chr5:14440397-14444098. Red lines define the SRO region, while the blue one pinpoint the splicing site. The title of every subplot provides three information: chromosome location, BAM file name, and gene strand.

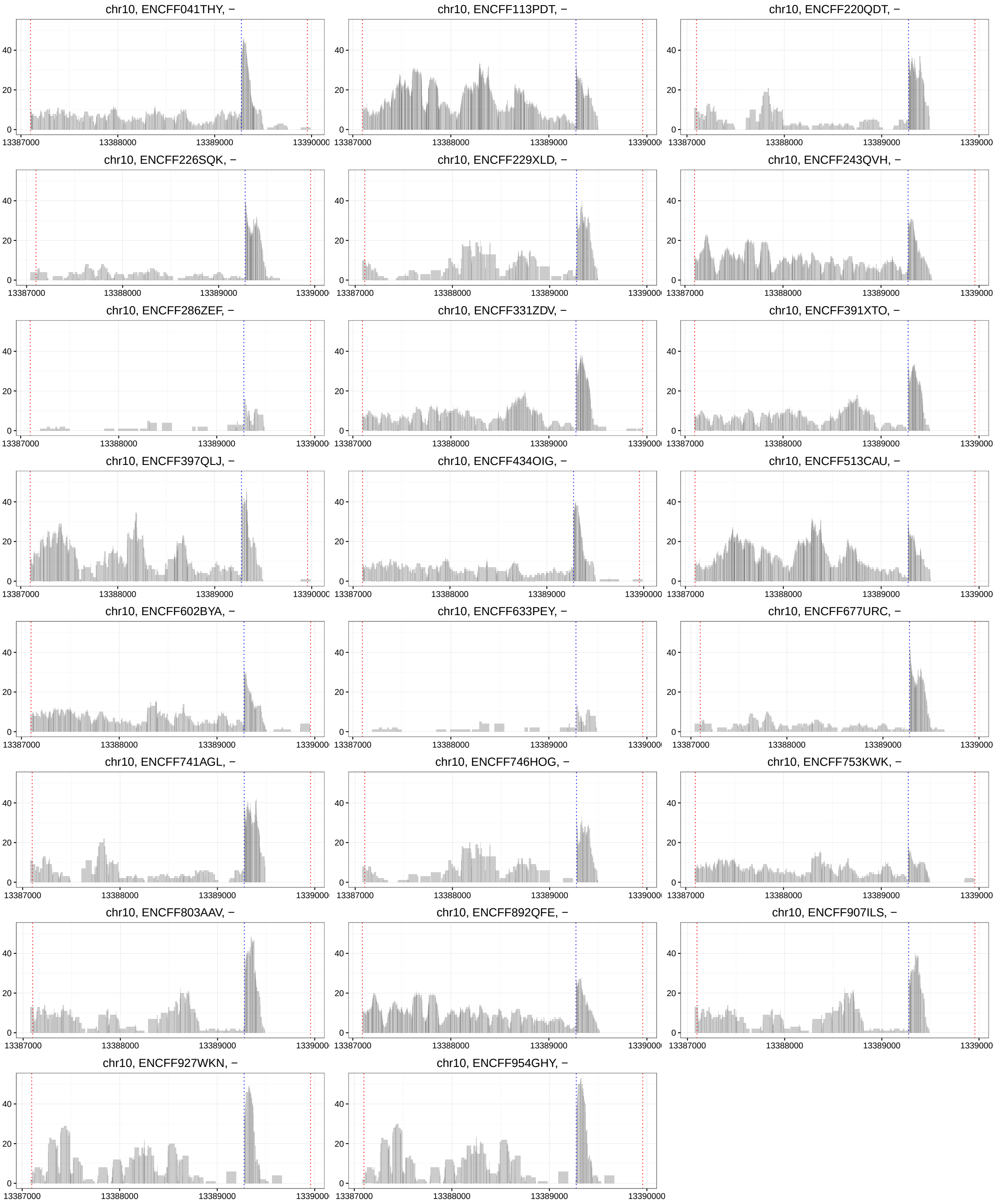

Figure S68: Read coverage in 23 fetal brain BAM files regarding *SEPHS1* gene at chr10:13387098-13389957. Red lines define the SRO region, while the blue one pinpoint the splicing site. The title of every subplot provides three information: chromosome location, BAM file name, and gene strand.

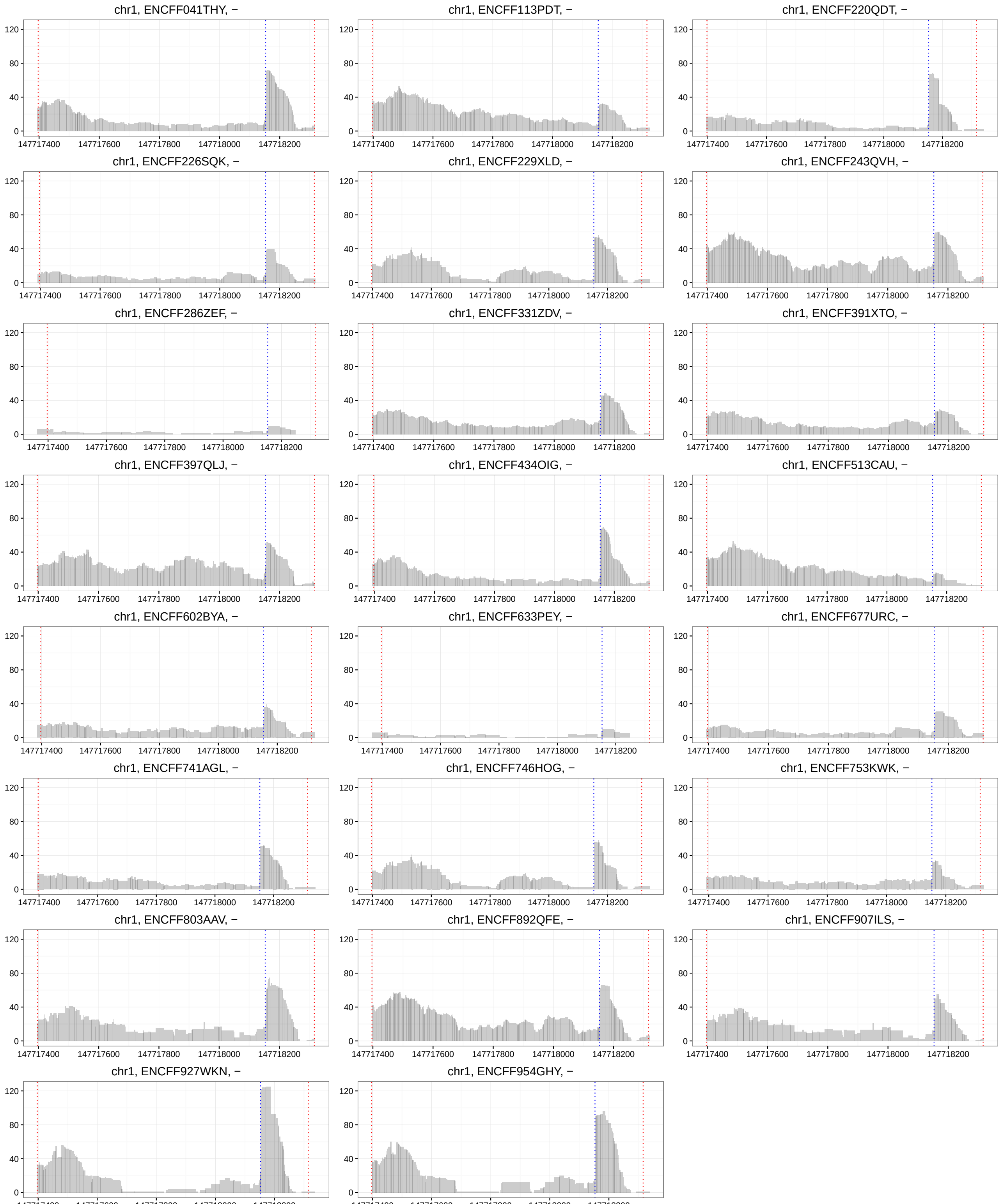

Figure S69: Read coverage in 23 fetal brain BAM files regarding *NBPF8* gene at chr1:147717397-147718316. Red lines define the SRO region, while the blue one pinpoint the splicing site. The title of every subplot provides three information: chromosome location, BAM file name, and gene strand.

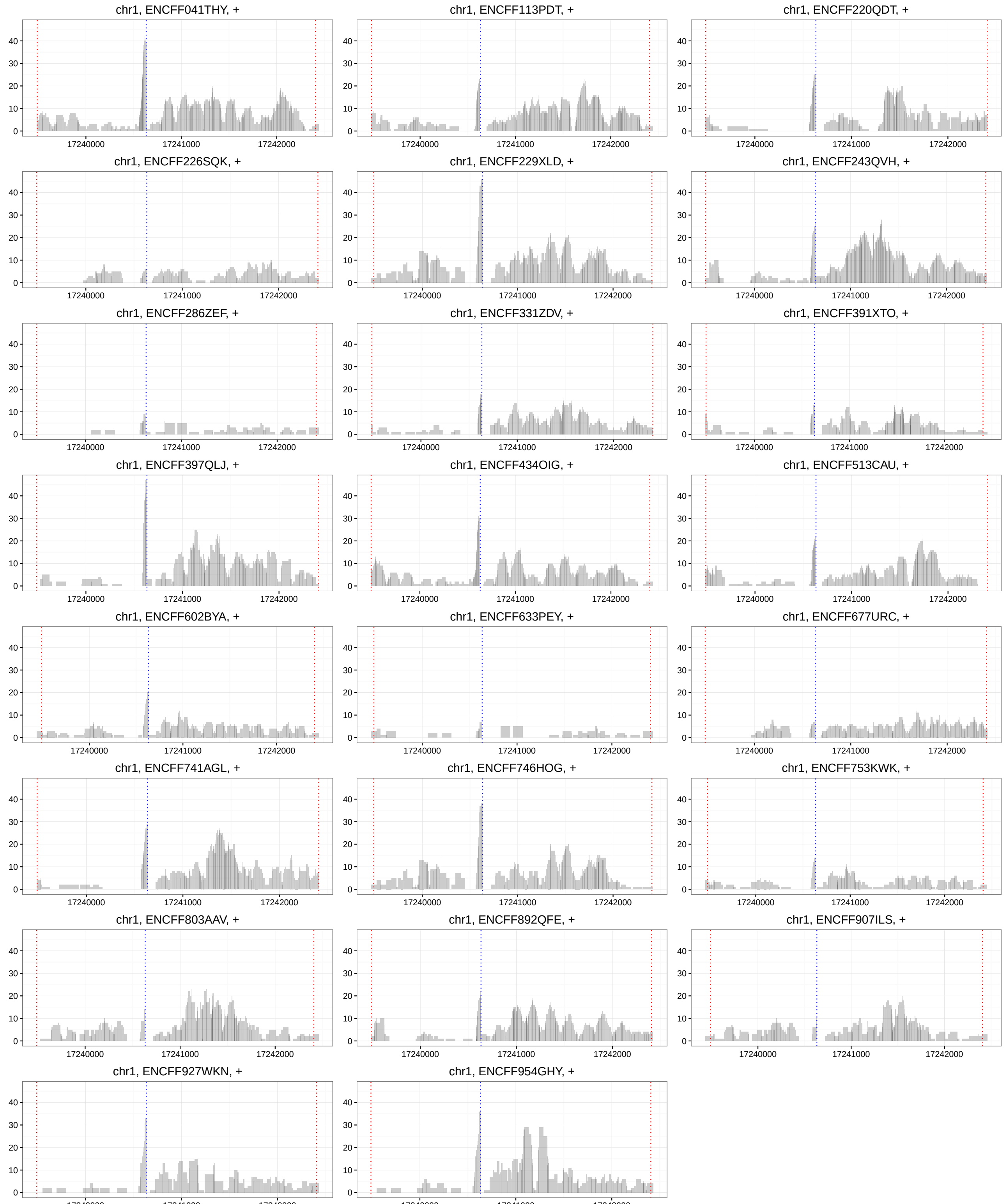

Figure S70: Read coverage in 23 fetal brain BAM files regarding *CROCC* gene at chr1:17239490-17242407. Red lines define the SRO region, while the blue one pinpoint the splicing site. The title of every subplot provides three information: chromosome location, BAM file name, and gene strand.

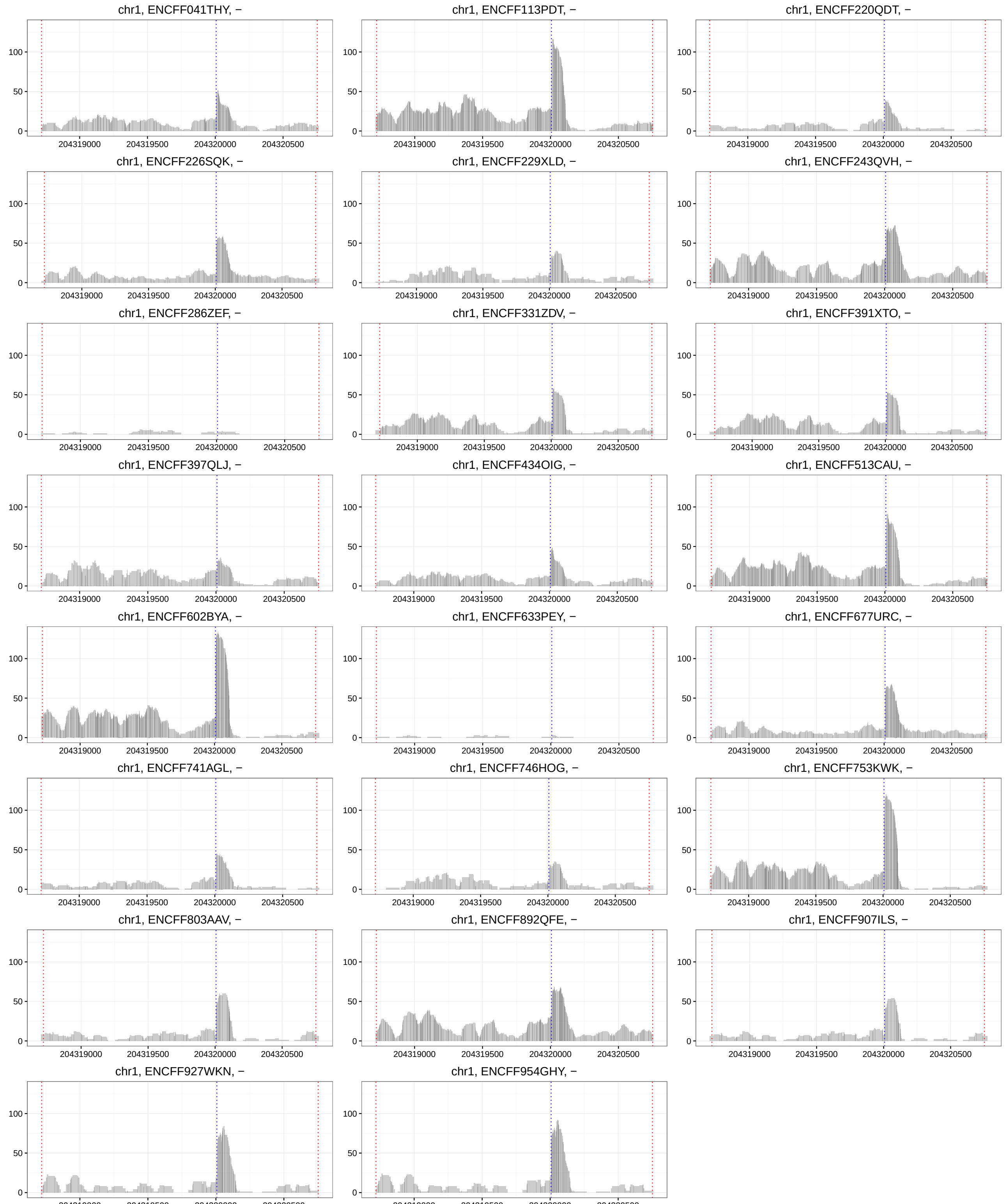

Figure S71: Read coverage in 23 fetal brain BAM files regarding *PLEKHA6* gene at chr1:204318719-204320752. Red lines define the SRO region, while the blue one pinpoint the splicing site. The title of every subplot provides three information: chromosome location, BAM file name, and gene strand.

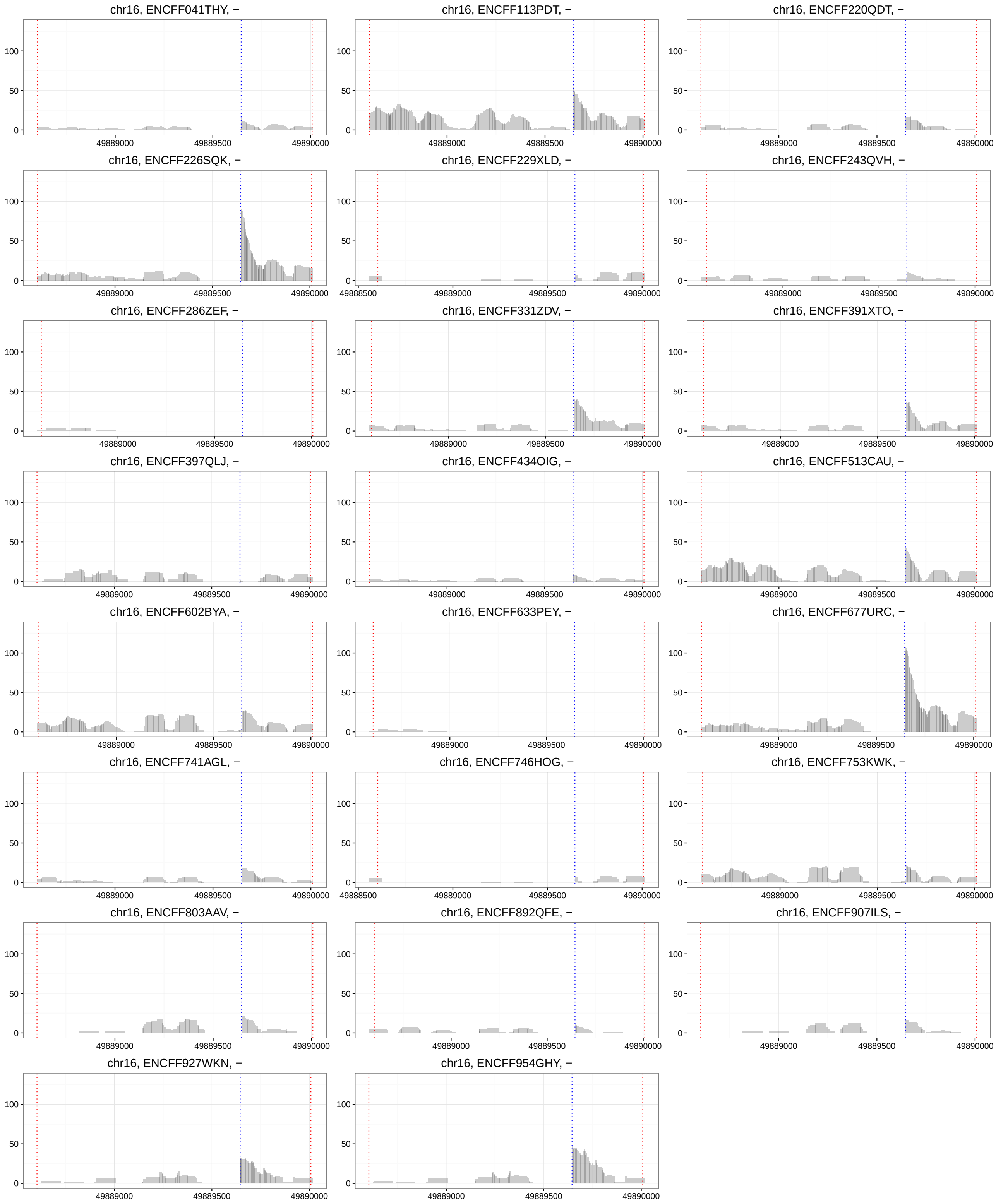

Figure S72: Read coverage in 23 fetal brain BAM files regarding *ZNF423* gene at chr16:49888603-49890008. Red lines define the SRO region, while the blue one pinpoint the splicing site. The title of every subplot provides three information: chromosome location, BAM file name, and gene strand.

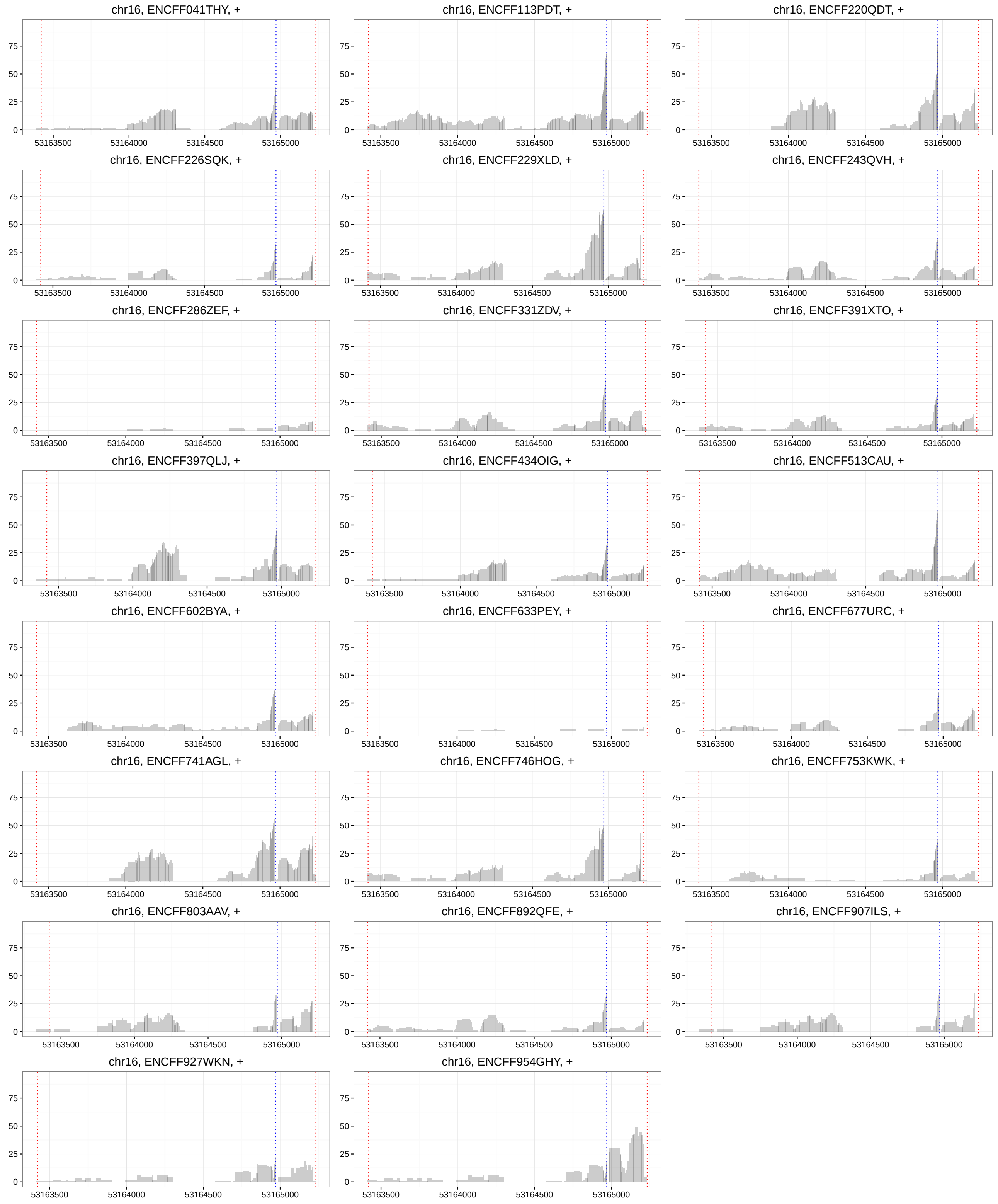

Figure S73: Read coverage in 23 fetal brain BAM files regarding *CHD9* gene at chr16:53163420-53165233. Red lines define the SRO region, while the blue one pinpoint the splicing site. The title of every subplot provides three information: chromosome location, BAM file name, and gene strand.

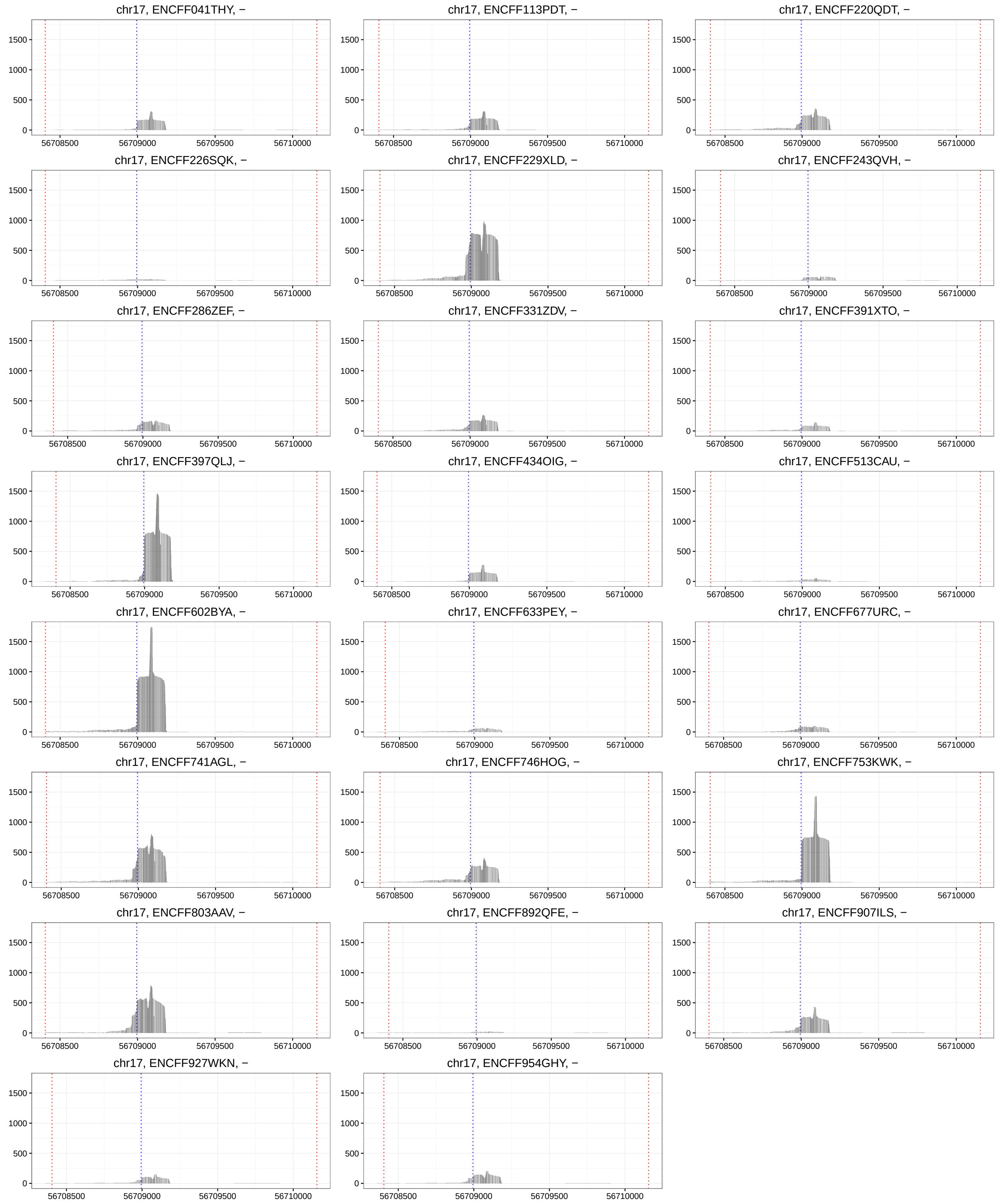

Figure S74: Read coverage in 23 fetal brain BAM files regarding *TEX14* gene at chr17:56708405-56710156. Red lines define the SRO region, while the blue one pinpoint the splicing site. The title of every subplot provides three information: chromosome location, BAM file name, and gene strand.

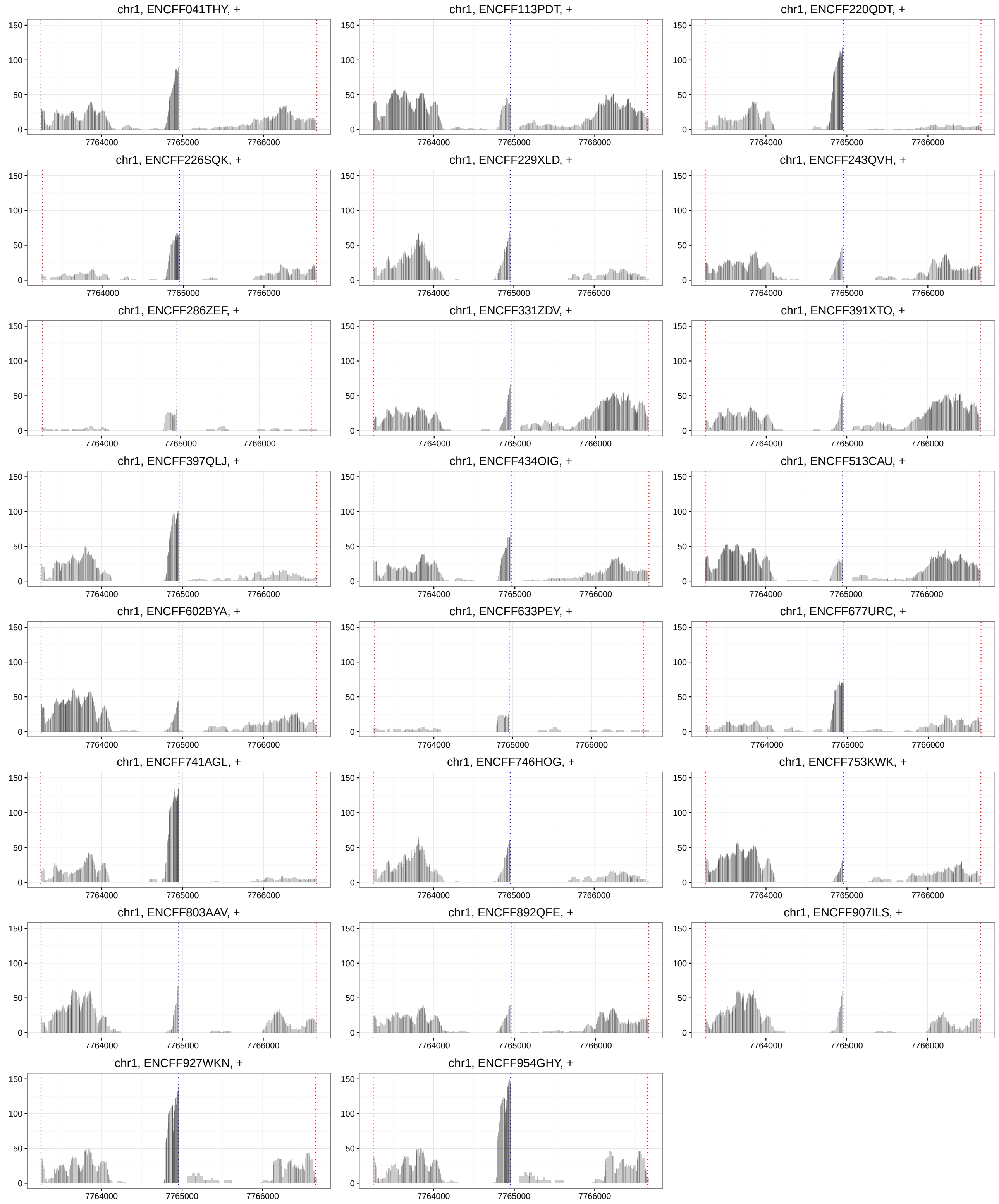

Figure S75: Read coverage in 23 fetal brain BAM files regarding *CAMTA1* gene at chr1:7763249-7766656. Red lines define the SRO region, while the blue one pinpoint the splicing site. The title of every subplot provides three information: chromosome location, BAM file name, and gene strand.

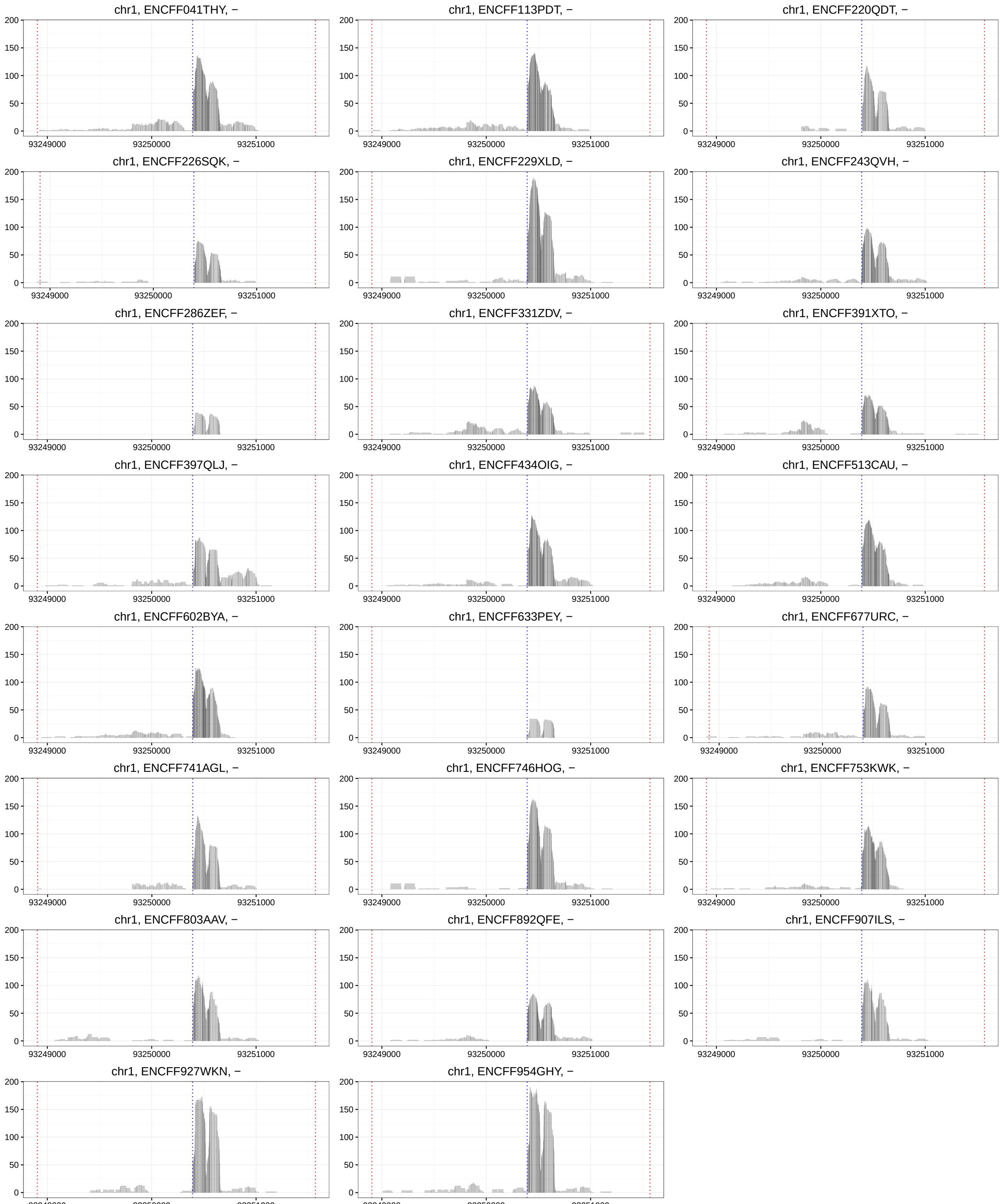

Figure S76: Read coverage in 23 fetal brain BAM files regarding *EVI5* gene at chr1:93248904-93251568. Red lines define the SRO region, while the blue one pinpoint the splicing site. The title of every subplot provides three information: chromosome location, BAM file name, and gene strand.

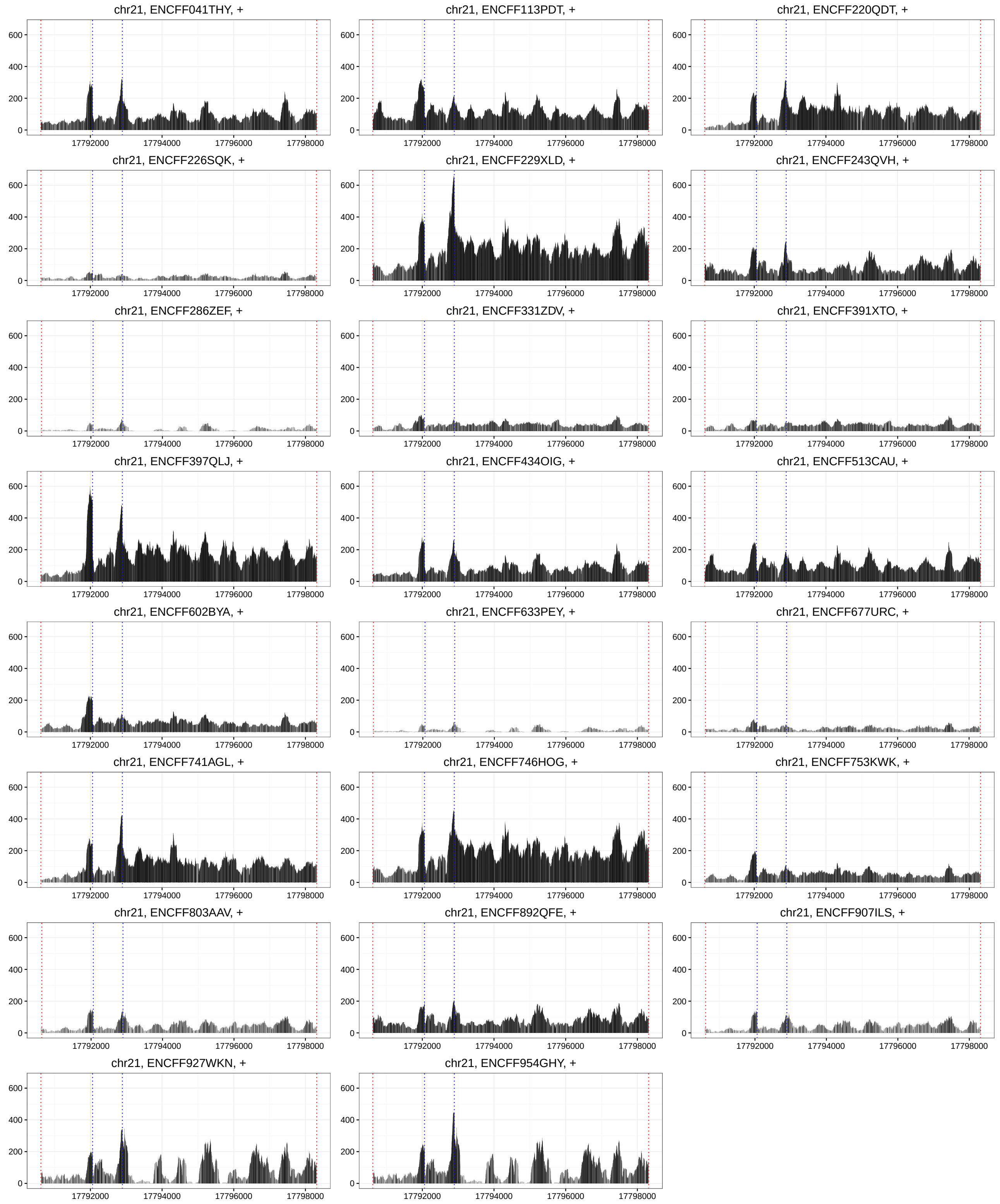

Figure S77: Read coverage in 23 fetal brain BAM files regarding *LINC00478* gene at chr21:17790619-17798315. Red lines define the SRO region, while the blue one pinpoint the splicing site. The title of every subplot provides three information: chromosome location, BAM file name, and gene strand.

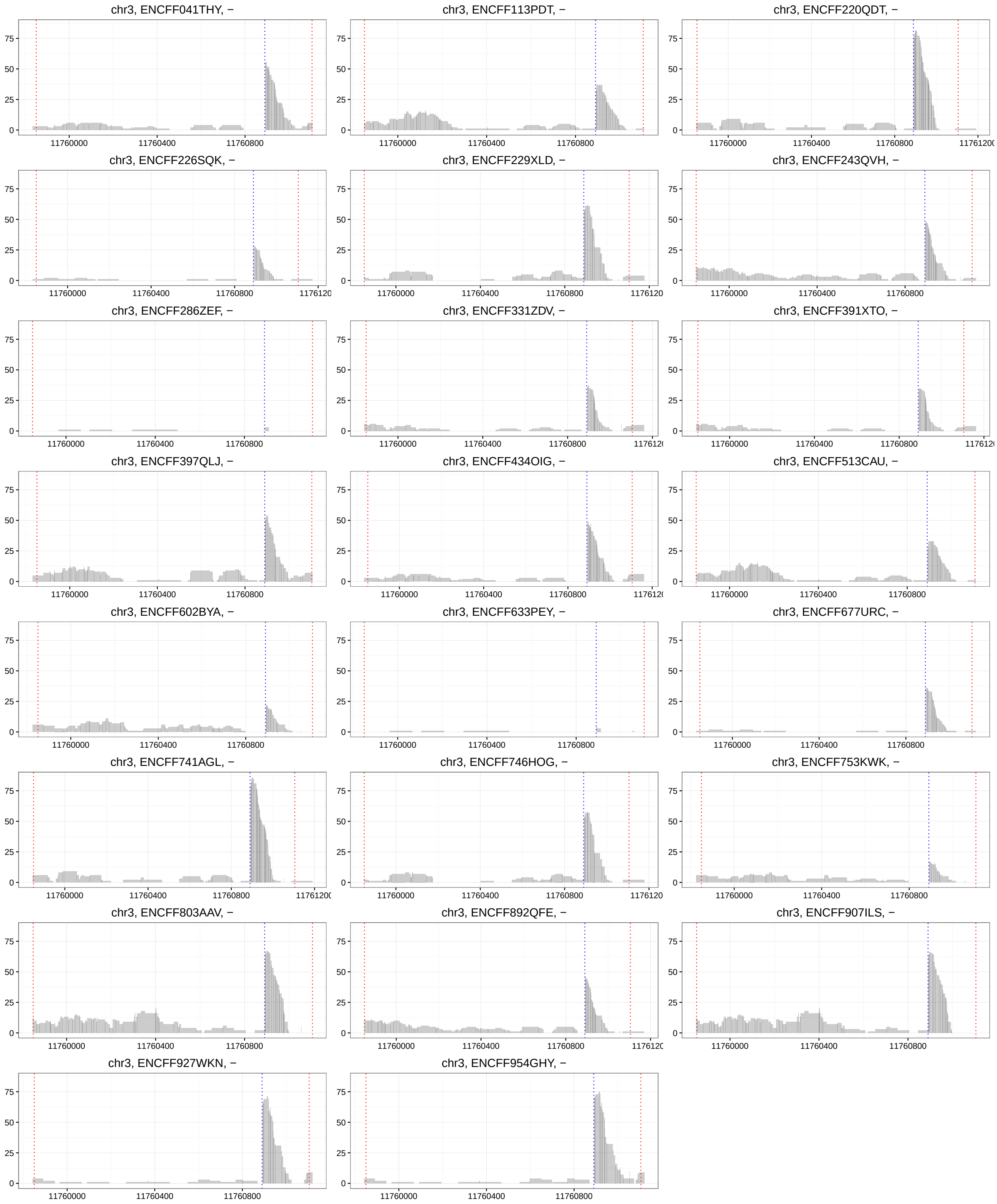

Figure S78: Read coverage in 23 fetal brain BAM files regarding *VGLL4* gene at chr3:11759850-11761105. Red lines define the SRO region, while the blue one pinpoint the splicing site. The title of every subplot provides three information: chromosome location, BAM file name, and gene strand.

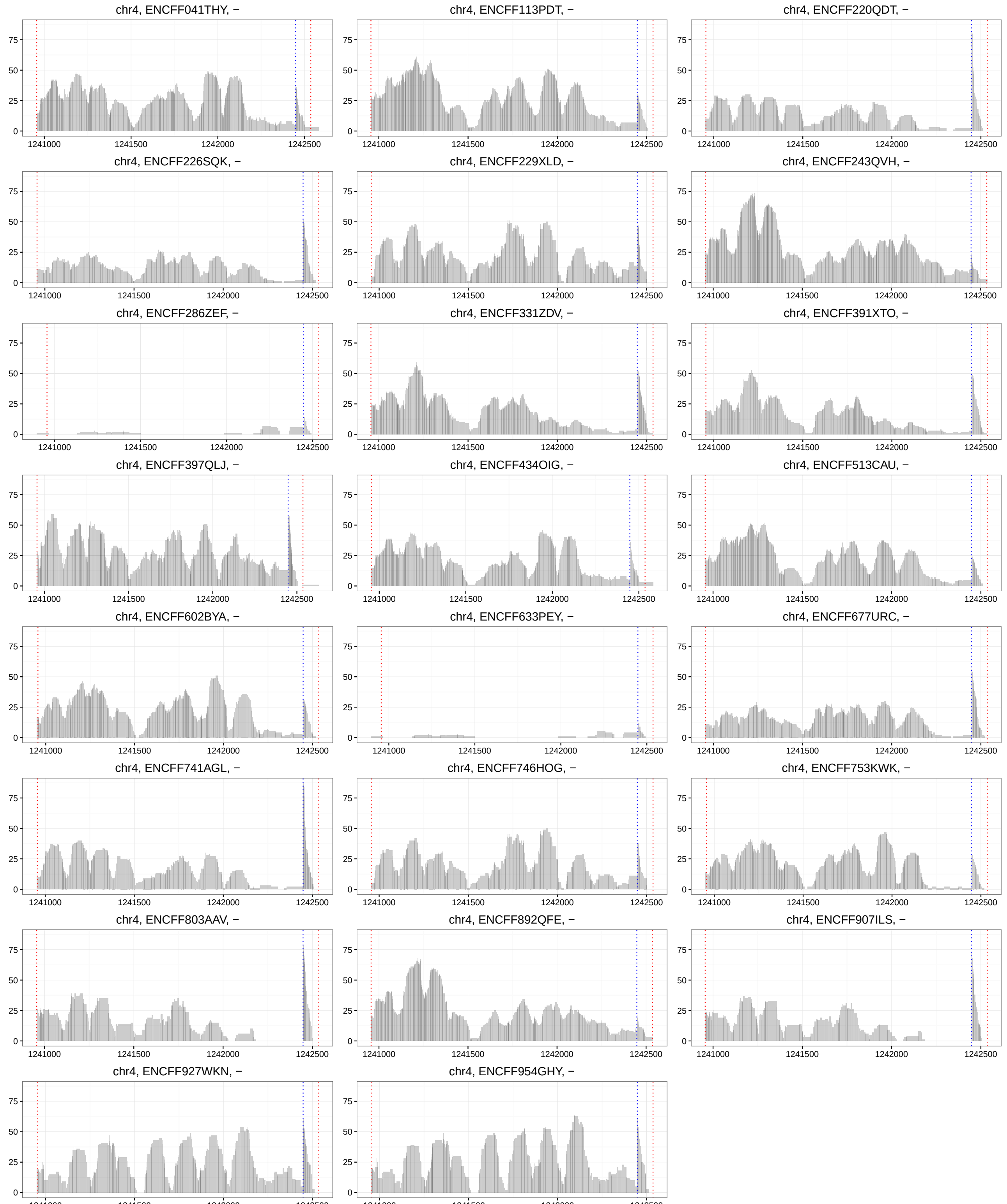

Figure S79: Read coverage in 23 fetal brain BAM files regarding *CTBP1* gene at chr4:1240956-1242536. Red lines define the SRO region, while the blue one pinpoint the splicing site. The title of every subplot provides three information: chromosome location, BAM file name, and gene strand.

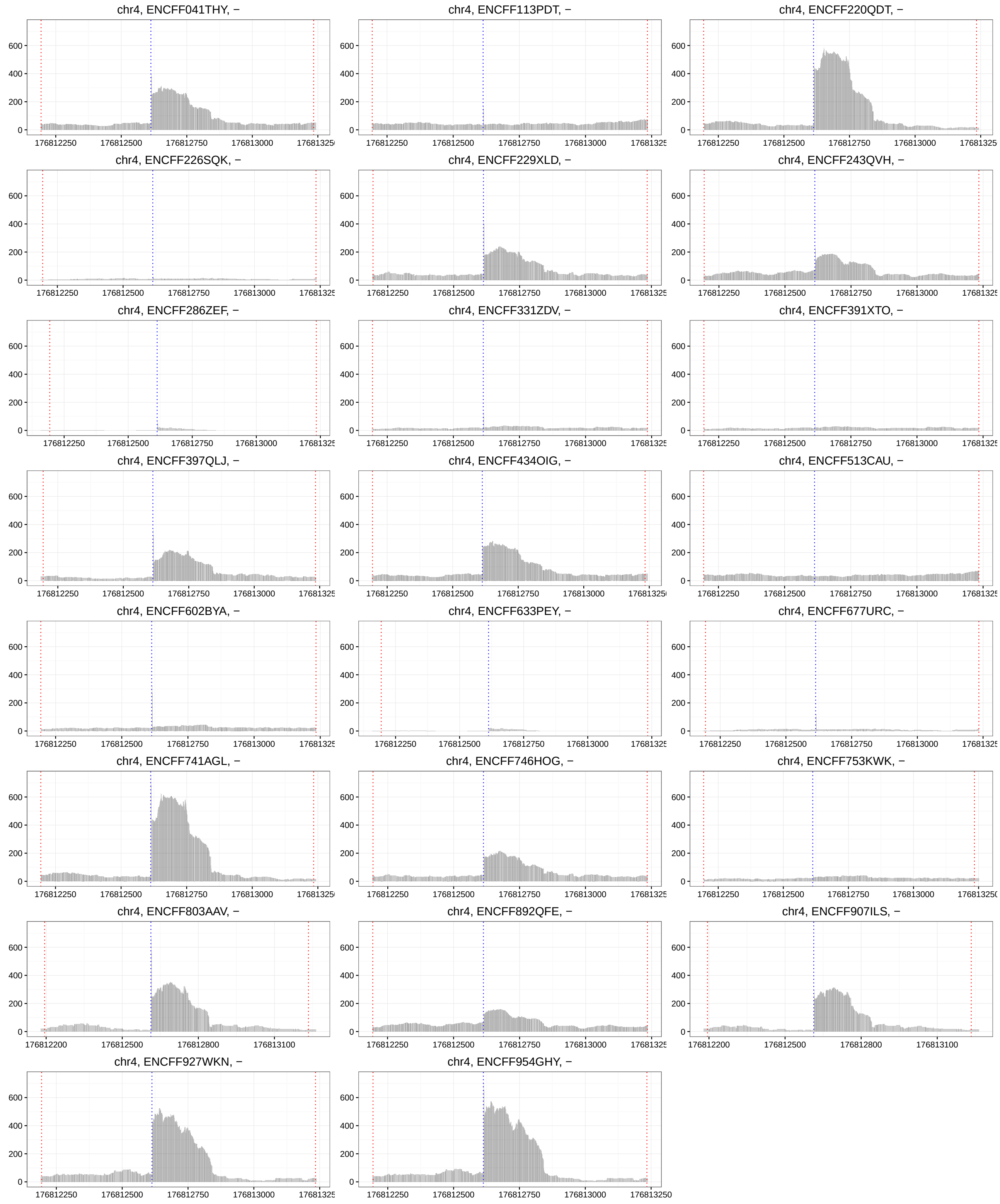

Figure S80: Read coverage in 23 fetal brain BAM files regarding *GPM6A* gene at chr4:176812194-176813234. Red lines define the SRO region, while the blue one pinpoint the splicing site. The title of every subplot provides three information: chromosome location, BAM file name, and gene strand.

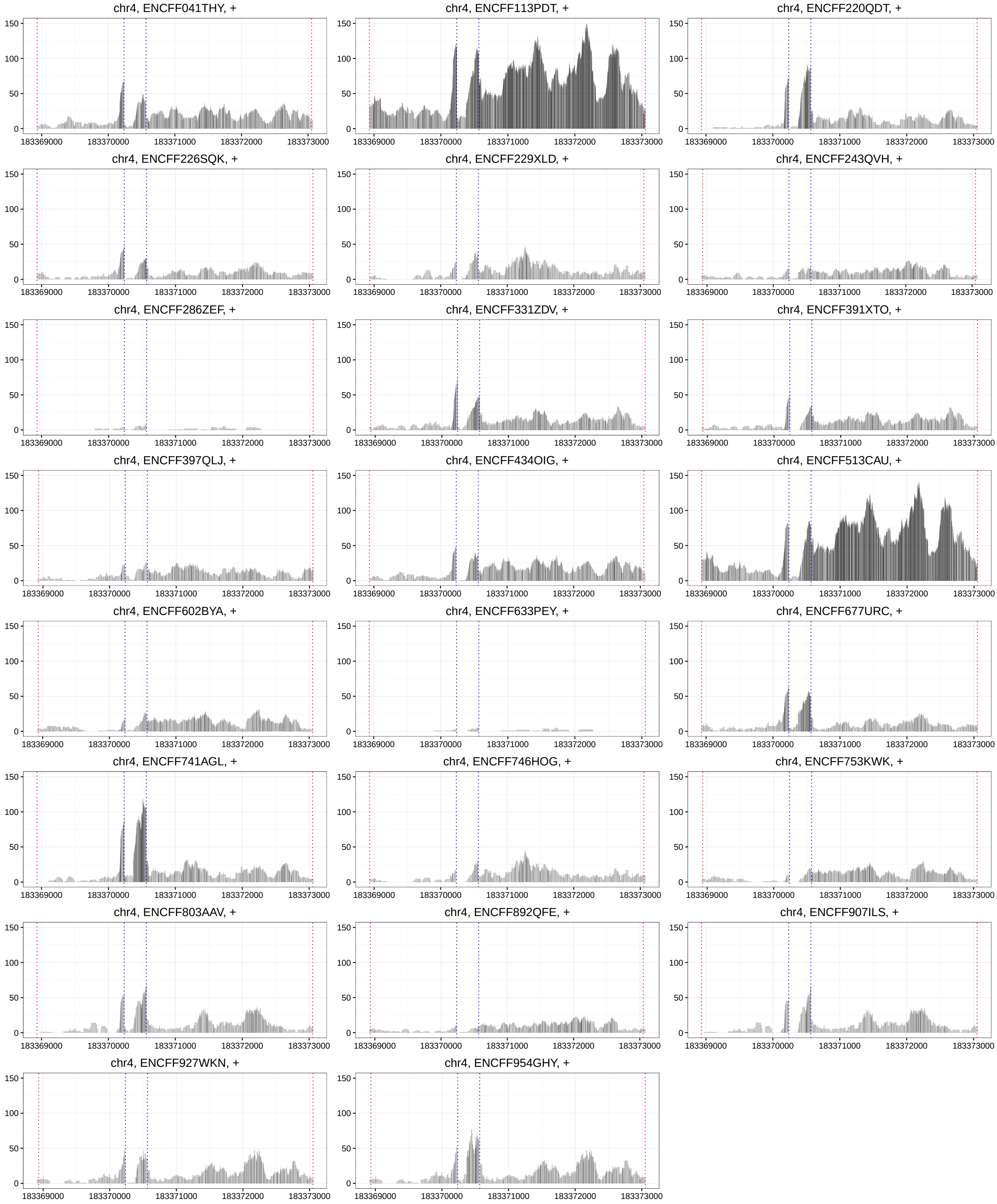

Figure S81: Read coverage in 23 fetal brain BAM files regarding *TENM3* gene at chr4:183368933-183373052. Red lines define the SRO region, while the blue one pinpoint the splicing site. The title of every subplot provides three information: chromosome location, BAM file name, and gene strand.

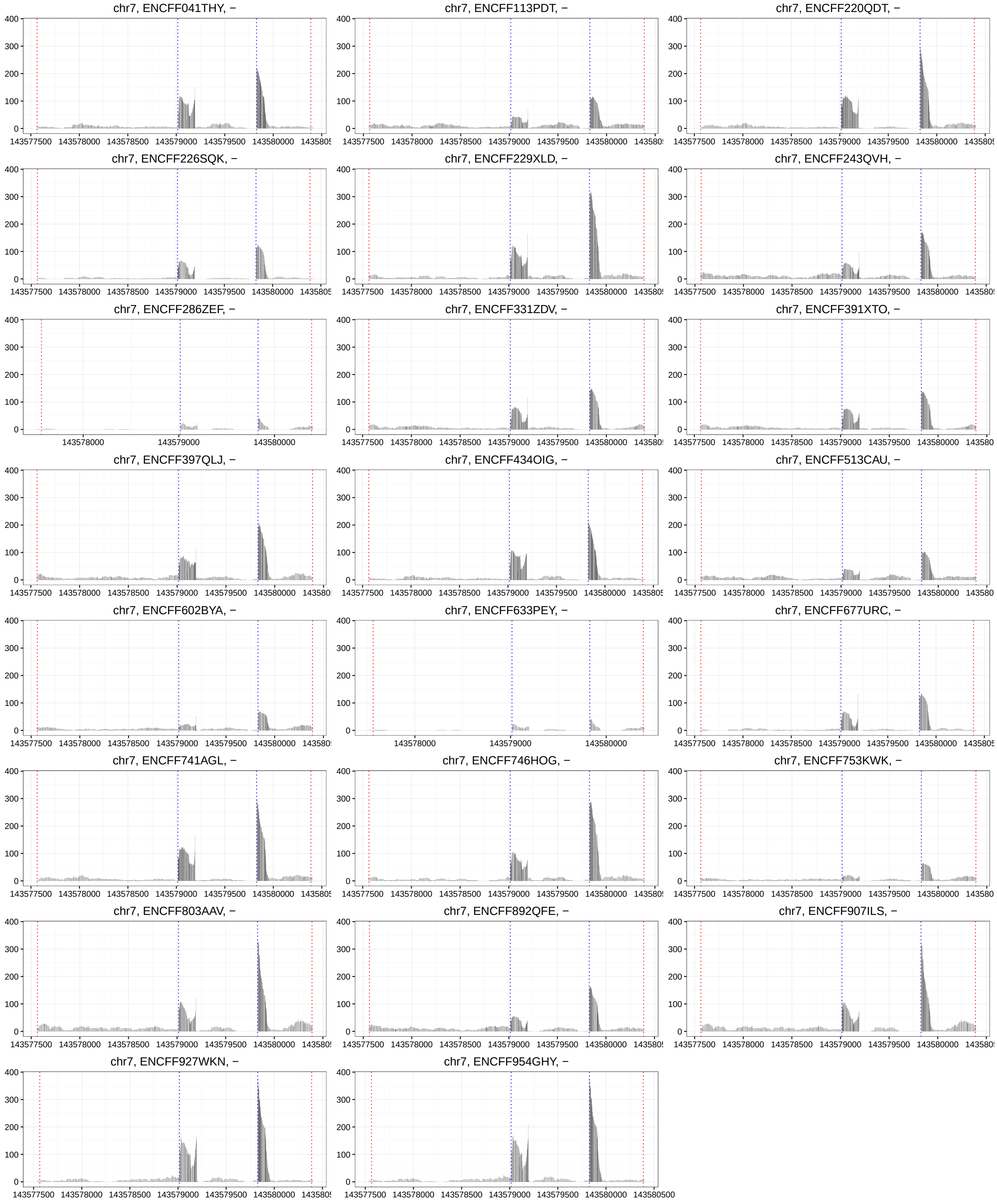

Figure S82: Read coverage in 23 fetal brain BAM files regarding *FAM115A* gene at chr7:143577564-143580388. Red lines define the SRO region, while the blue one pinpoint the splicing site. The title of every subplot provides three information: chromosome location, BAM file name, and gene strand.

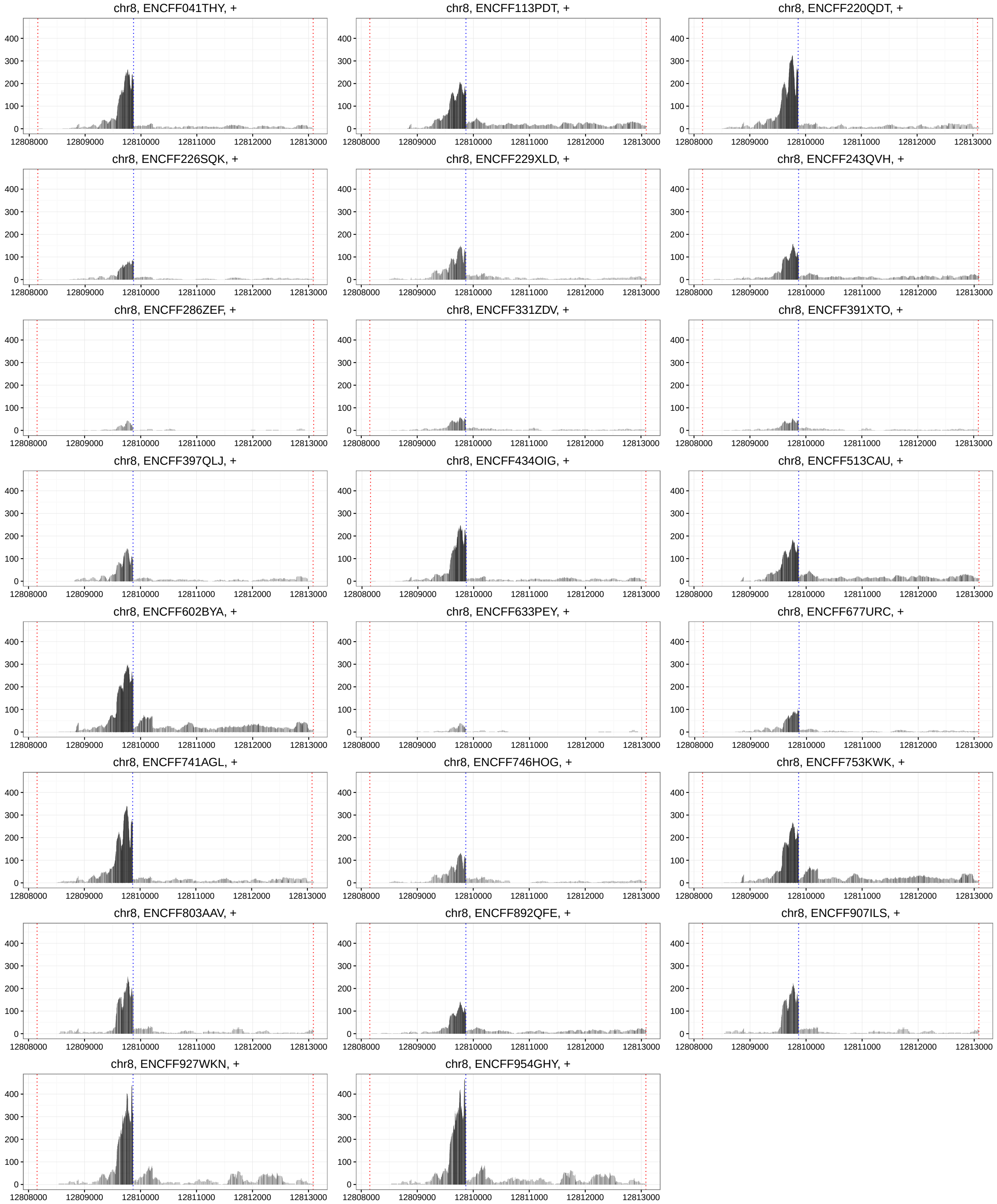

Figure S83: Read coverage in 23 fetal brain BAM files regarding *KIAA1456* gene at chr8:12808153-12813083. Red lines define the SRO region, while the blue one pinpoint the splicing site. The title of every subplot provides three information: chromosome location, BAM file name, and gene strand.

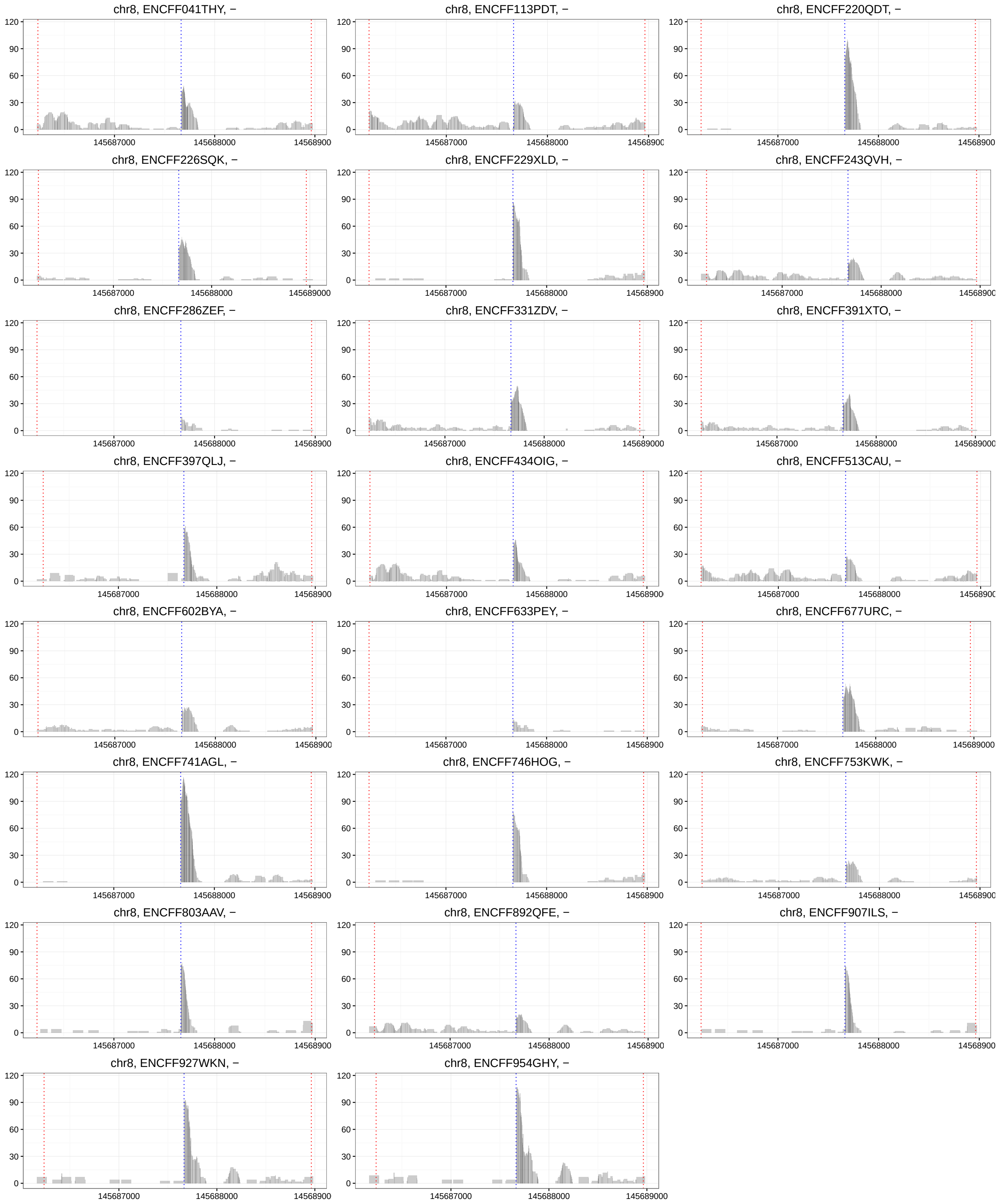

Figure S84: Read coverage in 23 fetal brain BAM files regarding *CYHR1* gene at chr8:145686236-145688964. Red lines define the SRO region, while the blue one pinpoint the splicing site. The title of every subplot provides three information: chromosome location, BAM file name, and gene strand.

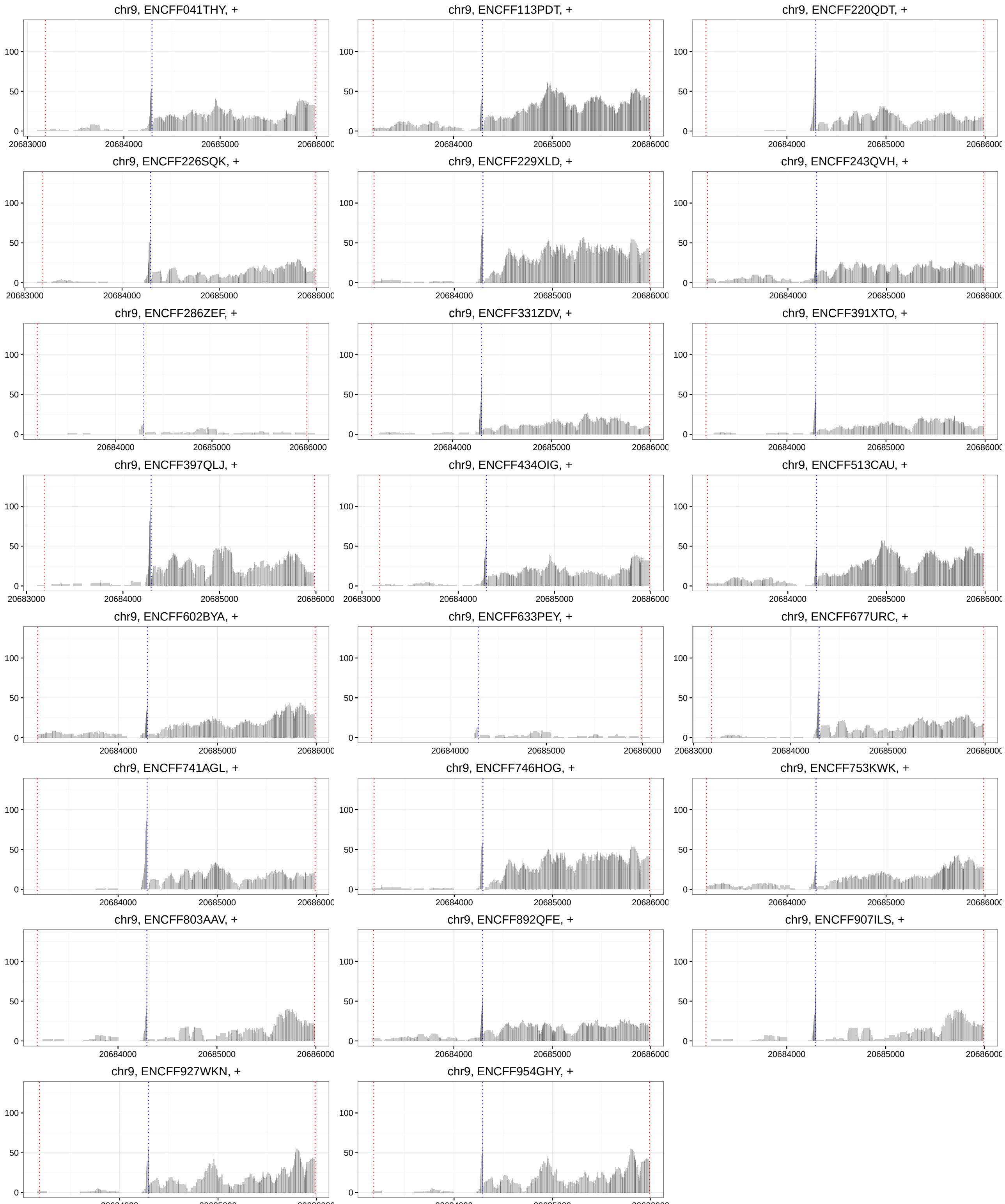

Figure S85: Read coverage in 23 fetal brain BAM files regarding *FOCAD* gene at chr9:20683184-20685987. Red lines define the SRO region, while the blue one pinpoint the splicing site. The title of every subplot provides three information: chromosome location, BAM file name, and gene strand.

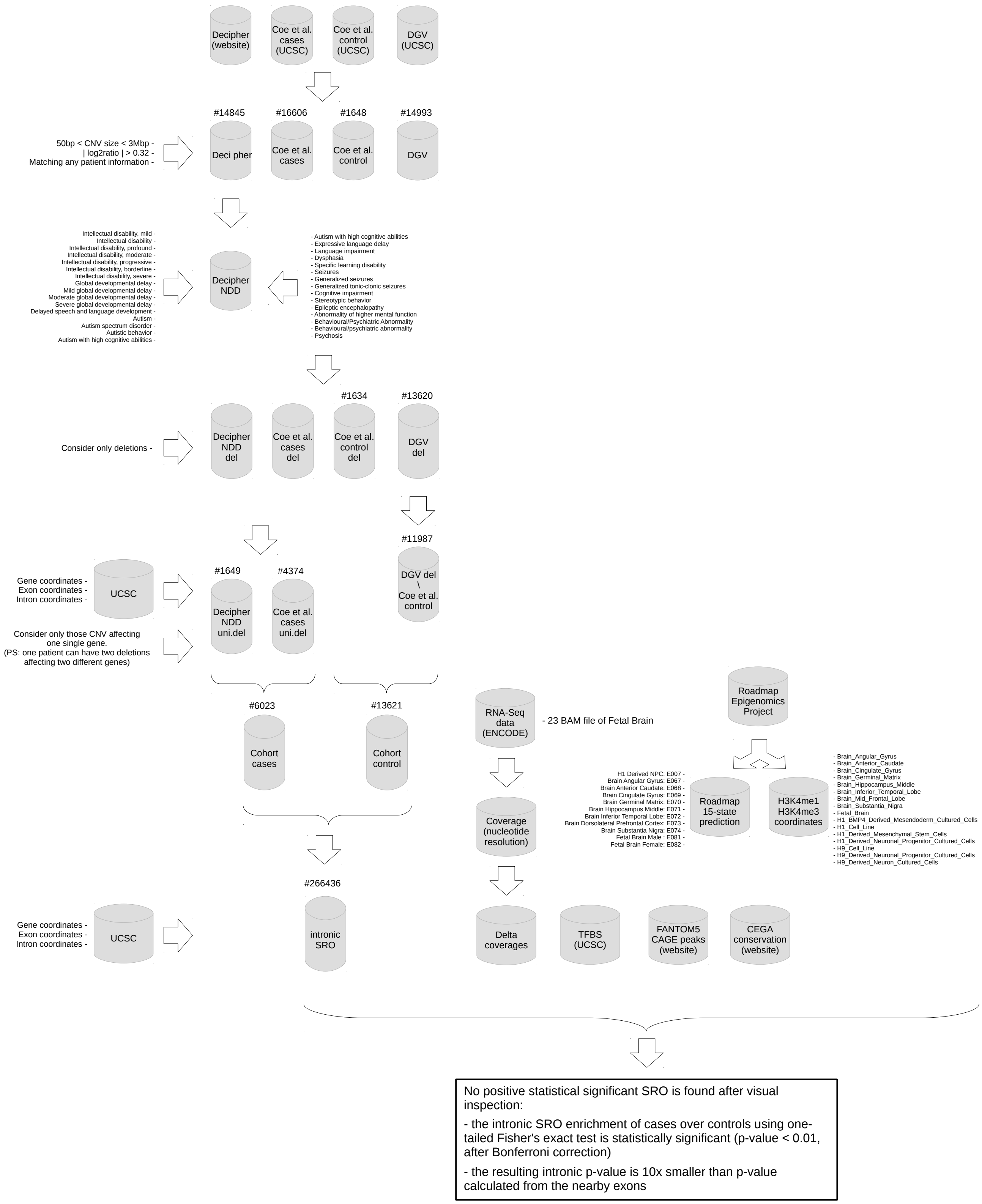

Figure S86: Whole genome analysis overview in traditional settings.

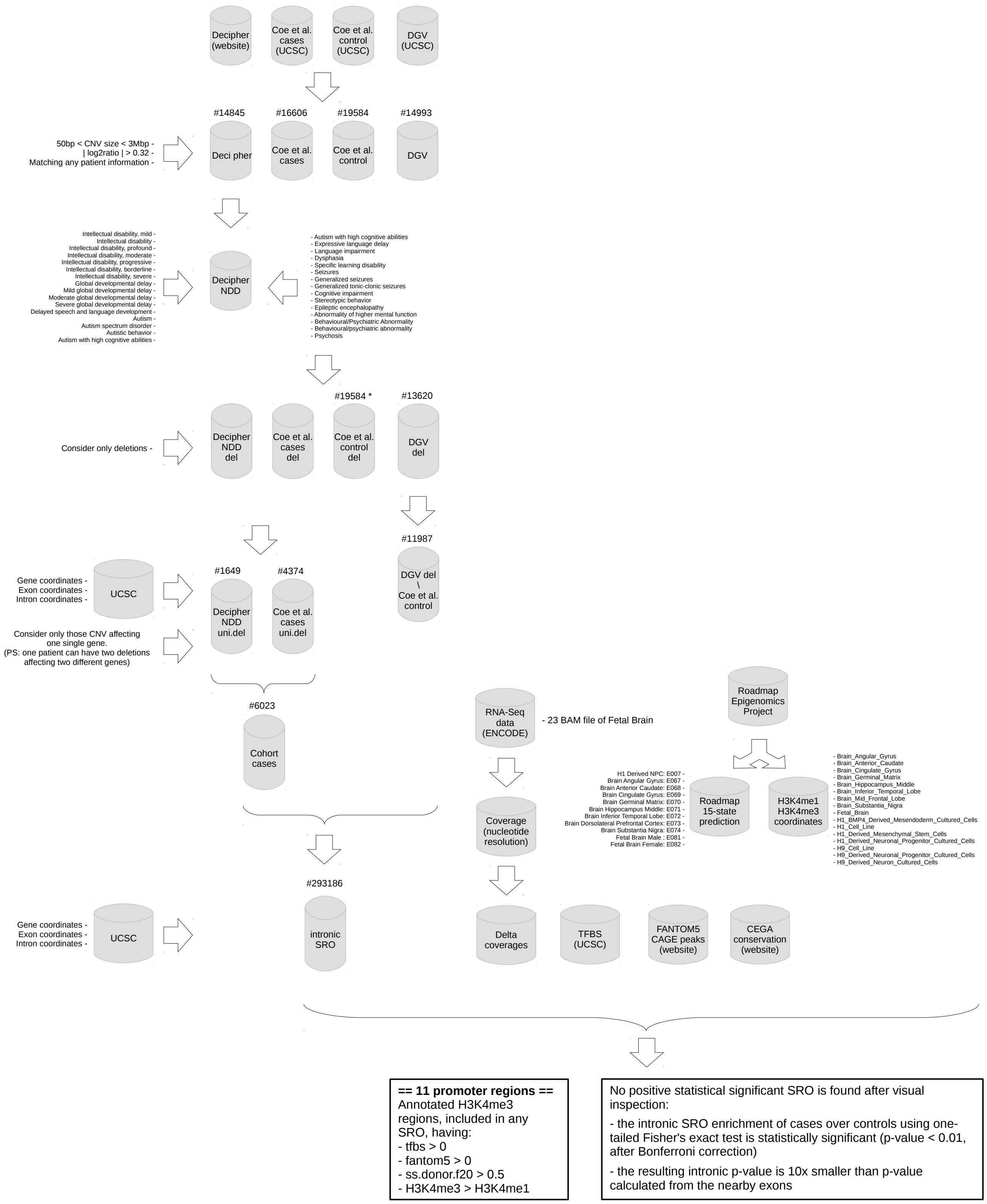

Figure S87: This whole genome analysis overview is for both the knowledge-driven analysis and the data-driven analysis in alternative settings. The two workflows differ for the final results (see Figure 6, article), the former produced a list of 11 putative promoter regions, the latter produced no positive statistical significant region after visual inspection. “*”: although we pull duplications out the dataset, the number of patients stays constant.

TCAGCCCCTTCCTCCCACCCCACTTGCCCGGCCAGCGCTCCCCACAGTGACTCTGGATTGCAGCAAGC

CACTGGCTTTTTACCAAATCGGGAGCTGCAGTATGTTTTCTTCTTCTTCTTCTTCTTCAGAGGGAATA

ATTTCAGCCCCCTTCAGCTCGCATGACAATCTTGTGTGACTGGGGAGAGTAGGCGGGCACATCCTTTT

ACATGCTAATACTGCCCCACCTCCTTTGAACCCTCCTCTAAGTGCCTGGCTTCCTTTTATTTTTATGG

TTTATTTTTATTTTATTTTATTTTTATTTTACCTCCCCCAAATTCACCGTAGGGGGAAAAATTCTTTC

TTGGTTCTGTCCCTCCCCCCACACGCCTCGTTCCCCAGGGAAGGGAGACCCCGAGAGGCTCGGTACTT

GCCCGCGGCGTTGCTTCCACCGGGGCTCCGGGATGGCCACGGCGGGTGCAGGGGGCAGTGATCGAAGC

GGCAGCTCGGTCTAGGCTGTCGCTCCATGTGCCCGGCGAGCACAGCGTTTGGGTCCCCCAGCGAGCTG

CCACTGCCGCAGCCCGGAAGGAATGTCCCCTGTTGTTAAGGACCCTGACTGTTTTACACCAATGATTT
M S P V V K D P D C F T P M I

GCCACTGCAAAGTTGCTTGCACCAACAACACTTTATCGTTGATGTTTGGATGCAAG|GTAGGAAGAGGT
C H C K V A C T N N T L S L M F G C K|

TCATGTCTTTTTAATGCATGTTGCCTCCCTCGGGGGAGAGAACAATTCCTTCTGTTGCTTGTTTATGT

Figure S88: Excerpt of HPin7 sequence at chr11:84431259-84432006 (reverse strand) to highlight the coding regions (red, see Supplementary Note 5) and CAGE peaks (underlined). CFEin7 coding region is at chr11:84431339-84431401. The upstream AA sequence shown in gray (MSPVVKDPDCFTP) corresponds to the mismatch between the predicted human isoforms and the mouse protein isoform Q91XM9-2 (see Supplementary Notes 4 and 5). It is unclear whether this mismatch is due to an evolutionary acquired difference, an error in the mouse protein sequence or an error of the NCBI Gnomon prediction tool [8]. CAGE peaks coordinates are chr11:84431433-84431498, chr11:84431758-84431819, chr11:84431829-84431834, chr11:84431892-84431908 and chr11:84431944-84431992. The vertical bar at the end of the coding region details the splicing site towards *DLG2* exon 8. On a bioinformatics technical level, while the 3' ends of the CFEs (i.e. the splicing sites) were identifiable by single-nucleotide differential coverage, their 5' beginning assessments are challenging due to the possible multiple transcription start sites [9], as suggested by the several upstream robust CAGE peaks. Coordinates are 1-based inclusive and in hg19. CFEin7 coding DNA sequence is registered in GenBank with reference KY368395.

GCTGATTTACCTGCGAGGCTGTATGTGGATCTGTCTTTATGTGGCCACTGCCGCCAGCACGTACTGTC

AAGATAGAGGGAAGCCAAAGCGAGGATTGTAACTGCCCAAGCCTAGAGAGGGAGGTGAGCTGCCTCCG

GACTTGACTGCAGCTTCCCTTCCTCCTGACACACATGGAGTTTGGTGGCGACGGTGCCGGTGCCGAAG

AACTGCAGACAAATACTTAGCCAACTGGATGTGTGTGAGCTGGCTACAAAGGCAGCGCCTGCCTCACC

CGGACCTCCTGCTGGCAGGATGTAAATATACCTGCAGACTGGCCAAACAGGACTGCCTTTTCTCCCCC

AACCCCTCCCTCCTCCCACCCTCTCCCTCAGCTAACCAGCATGAGAGGAACTGAGAAAGCAACAGCCT

GCAAGTGACAGCTCCAGCCTGATTCTGTTCGTCTCTGAGCCGAGGTGGGAAGTTGATTGTGCGGCAGC

TTCATTGTGAATTCCTTCCATTGGCATCGCTATGTTTGCCTCTATCTGGTATGCTAAGAAGCTGGGTC
M F A S I W Y A K K L G

GCAGGTTCGTGCACAATGCCAGGAAGGCGAAATCAGAGAAG|GTATGGAATGAGTGGGGTTGAAAATTA
R R F V H N A R K A K S E K|

CCTTTCCGGTTTGCCAACTGCCTGCTCTGGACCATTGGTCTGAGTACAGACGCAGTCTTTATTTGCTT

Figure S89: Excerpt of HPin8 sequence at chr11:84148336-84149015 (reverse strand) to highlight the coding regions (red, see Supplementary Note 5) and CAGE peaks (underlined). CFEin8 coding region is at chr11:84148431-84148508. CAGE peaks coordinates are chr11:84148566-84148597, chr11:84148599-84148663, chr11:84148844-84148867 and chr11:84148870-84148881. The vertical bar at the end of the coding region details the splicing site towards *DLG2* exon 11. On a bioinformatics technical level, while the 3' ends of the CFEs (i.e. the splicing sites) were identifiable by single-nucleotide differential coverage, their 5' beginning assessments are challenging due to the possible multiple transcription start sites [9], as suggested by the several upstream robust CAGE peaks. Coordinates are 1-based inclusive and in hg19. CFEin8 coding DNA sequence is registered in GenBank with reference KY368394.

chromosome	start	end	width	exon number
chr11	83166056	83170967	4912	34
chr11	83172585	83173136	552	33
chr11	83177751	83177860	110	32
chr11	83180244	83180416	173	31
chr11	83182669	83182770	102	30
chr11	83183770	83183820	51	29
chr11	83191415	83191456	42	28
chr11	83194296	83194341	46	27
chr11	83195172	83195271	100	26
chr11	83243751	83243826	76	25
chr11	83252725	83252901	177	24
chr11	83342231	83344368	2138	23
chr11	83393201	83393468	268	22
chr11	83497484	83497835	352	21
chr11	83544657	83544813	157	20
chr11	83585463	83585531	69	19
chr11	83641371	83641526	156	18
chr11	83673928	83674066	139	17
chr11	83676367	83676511	145	16
chr11	83691549	83691685	137	15
chr11	83770358	83770527	170	14
chr11	83809966	83810090	125	13
chr11	83874504	83874554	51	12
chr11	83962281	83962334	54	11
chr11	83984194	83984323	130	10
chr11	84027868	84028382	515	9
chr11	84245613	84245774	162	8
chr11	84634121	84634465	345	7
chr11	84822705	84822779	75	6
chr11	84865600	84865695	96	5
chr11	84996264	84996409	146	4
chr11	85309701	85309832	132	3
chr11	85337631	85337797	167	2
chr11	85338262	85338314	53	1

Table S1: *DLG2* UCSC exon coordinates, retrieved from *TxDb.Hsapiens.UCSC.hg19.knownGene* R package version 3.2.2. All exons were unified into a single isoform.

chromosome	start	end	width	exon number
chr11	83166055	83170967	4913	47
chr11	83172585	83173192	608	46
chr11	83177751	83177860	110	45
chr11	83180244	83180416	173	44
chr11	83182669	83182770	102	43
chr11	83183770	83183820	51	42
chr11	83191415	83191692	278	41
chr11	83194296	83194341	46	40
chr11	83195172	83195271	100	39
chr11	83197198	83197294	97	38
chr11	83243751	83243826	76	37
chr11	83252725	83252901	177	36
chr11	83342231	83344368	2138	35
chr11	83362882	83362973	92	34
chr11	83392875	83393049	175	33
chr11	83393201	83393468	268	32
chr11	83435899	83435999	101	31
chr11	83436213	83436446	234	30
chr11	83497484	83497835	352	29
chr11	83544657	83544813	157	28
chr11	83585463	83585531	69	27
chr11	83641371	83641526	156	26
chr11	83673928	83674066	139	25
chr11	83676367	83676511	145	24
chr11	83691549	83691685	137	23
chr11	83770358	83770527	170	22
chr11	83809966	83810090	125	21
chr11	83874504	83874554	51	20
chr11	83877871	83878047	177	19
chr11	83962281	83962334	54	18
chr11	83983185	83983343	159	17
chr11	83984194	83984323	130	16
chr11	84027868	84028382	515	15
chr11	84148431	84148852	422	14
chr11	84245613	84245774	162	13
chr11	84397843	84398320	478	12
chr11	84634121	84634633	513	11
chr11	84822705	84822779	75	10
chr11	84843812	84844167	356	9
chr11	84865600	84865695	96	8
chr11	84996264	84996409	146	7
chr11	85180382	85180766	385	6
chr11	85236035	85236066	32	5
chr11	85309701	85309832	132	4
chr11	85336151	85336295	145	3
chr11	85337631	85337797	167	2
chr11	85338262	85338966	705	1

Table S2: *DLG2* Ensembl exon coordinates, retrieved from *GenomicFeatures* R package version 1.22.13, release 84, reference GRCh37.p13. All exons were unified into a single isoform.

Ensembl exon number	UCSC exon number
47	34
46	33
45	32
44	31
43	30
42	29
41	28
40	27
39	26
37	25
36	24
35	23
32	22
29	21
28	20
27	19
26	18
25	17
24	16
23	15
22	14
21	13
20	12
18	11
16	10
15	9
13	8
11	7
10	6
8	5
7	4
4	3
2	2
1	1

Table S3: Mapping between Ensembl and UCSC exon numbers for *DLG2* gene.

chromosome	start	end	width	exon number
chr7	91090786	91091048	263	1
chr7	91449754	91449915	162	2
chr7	91672492	91672954	463	3
chr7	91733091	91733144	54	4
chr7	91810479	91810529	51	5
chr7	91872376	91872500	125	6
chr7	91900735	91900904	170	7
chr7	91939999	91940135	137	8
chr7	91965596	91965740	145	9
chr7	91968118	91968256	139	10
chr7	91997166	91998815	1650	11
chr7	92040798	92040866	69	12
chr7	92062394	92062459	66	13
chr7	92066257	92066406	150	14
chr7	92088562	92088718	157	15
chr7	92126540	92126642	103	16
chr7	92234970	92235224	255	17
chr7	92286491	92286605	115	18
chr7	92375554	92375730	177	19
chr7	92386915	92386990	76	20
chr7	92417224	92417323	100	21
chr7	92418088	92418133	46	22
chr7	92420632	92420673	42	23
chr7	92427691	92427741	51	24
chr7	92428557	92428658	102	25
chr7	92430997	92431169	173	26
chr7	92437965	92438074	110	27
chr7	92442604	92442695	92	28
chr7	92444511	92449246	4736	29

Table S4: *Dlg2* UCSC exon coordinates, retrieved from *TxDb.Mmusculus.UCSC.mm10.knownGene* R package version 3.2.2. All exons were unified into a single isoform.

chromosome	start	end	width	strand	exon number
chr7	90915744	90916086	343	+	1
chr7	91279442	91279603	162	+	2
chr7	91514852	91515314	463	+	3
chr7	91553285	91553660	376	+	4
chr7	91574802	91574855	54	+	5
chr7	91648259	91648309	51	+	6
chr7	91719778	91719902	125	+	7
chr7	91748638	91748807	170	+	8
chr7	91788170	91788306	137	+	9
chr7	91810939	91811083	145	+	10
chr7	91813757	91813895	139	+	11
chr7	91843204	91844853	1650	+	12
chr7	91895796	91895864	69	+	13
chr7	91921111	91921176	66	+	14
chr7	91925497	91925646	150	+	15
chr7	91940008	91940164	157	+	16
chr7	91979857	91979959	103	+	17
chr7	92034522	92034890	369	+	18
chr7	92081648	92081949	302	+	19
chr7	92132855	92134707	1853	+	20
chr7	92220656	92220832	177	+	21
chr7	92231936	92232011	76	+	22
chr7	92262252	92262351	100	+	23
chr7	92263114	92263159	46	+	24
chr7	92265641	92265682	42	+	25
chr7	92272685	92272735	51	+	26
chr7	92273551	92273652	102	+	27
chr7	92275991	92276163	173	+	28
chr7	92282915	92283024	110	+	29
chr7	92287729	92287820	92	+	30
chr7	92289638	92292131	2494	+	31

Table S5: *Dlg2* Ensembl exon coordinates (mm10), BALB/cJ strain. All exons were unified into a single isoform.

chromosome	start	end	width	strand	exon number
chr7	91266035	91266377	343	+	1
chr7	91626714	91626875	162	+	2
chr7	91863528	91863990	463	+	3
chr7	91902921	91903296	376	+	4
chr7	91925797	91925850	54	+	5
chr7	91996610	91996660	51	+	6
chr7	92058858	92058982	125	+	7
chr7	92087930	92088099	170	+	8
chr7	92127690	92127826	137	+	9
chr7	92147820	92147964	145	+	10
chr7	92150145	92150283	139	+	11
chr7	92181780	92183429	1650	+	12
chr7	92226350	92226418	69	+	13
chr7	92249008	92249073	66	+	14
chr7	92254028	92254177	150	+	15
chr7	92273175	92273331	157	+	16
chr7	92314759	92314861	103	+	17
chr7	92369395	92369763	369	+	18
chr7	92417900	92418201	302	+	19
chr7	92468539	92470391	1853	+	20
chr7	92550155	92550331	177	+	21
chr7	92560927	92561002	76	+	22
chr7	92591908	92592007	100	+	23
chr7	92592770	92592815	46	+	24
chr7	92595297	92595338	42	+	25
chr7	92602298	92602348	51	+	26
chr7	92603164	92603265	102	+	27
chr7	92605604	92605776	173	+	28
chr7	92612539	92612648	110	+	29
chr7	92617168	92617259	92	+	30
chr7	92619077	92623800	4724	+	31

Table S6: *Dlg2* Ensembl exon coordinates (mm10), A/J strain. All exons were unified into a single isoform.

Ref	ULB Patient ID	CNV type	*DLG2* CNV hg19 coordinates	CNV size (Mb)	*DLG2* deleted features	Gender	Other rare CNVs	Inheritance of *DLG2* variant	Age of presentation	Clinical description
I	317136	del	chr11:84245639-84772741	0.52	exons 7-8	male	0 cnv	inherited from an unaffected mother	3	Global Developmental Delay, motor developmental milestones delay, moderate language delay, stereotypic behavior, delay in social skills, timid, apprehensive to unknown, learning difficulties
II	317185	del	chr11:84334015-84797219	0.46	exon 7	male	0 cnv	inherited from an unaffected mother	1 1/2	Global Developmental Delay, Severe Language Delay, Social skills delay, special needs education, mild hypotonia, fine motor skills delay, postnatal microcephaly, poor visual contact, absence of facial expressions, low anterior hairline, recurrent otitis media, ear skin tag

Table S7: Summary of genetic and clinical descriptions of ULB patients.

Ref	DECIPHER Patient ID	CNV type	*DLG2* CNV hg19 coordinates	CNV size (Mb)	*DLG2* deleted features	Gender	Other rare CNVs	Inheritance of *DLG2* variant	Age of presentation	Clinical description
III	248668	del	chr11:84548697-84628963	0.08	intron 7	male	32 cnvs	unknown	14	Abnormality of the face, Cognitive impairment, Low anterior hairline, Macrocephaly, Obesity, Short philtrum, Tall stature, Upslanted palpebral fissure

IV	256592	del	chr11:84456097-84607440	0.15	exon 7	male	1 cnv: 30kbp dup on chrY. Genes: AKAP17A, ASMT.	unknown	11	Autism, Constipation, Intellectual disability, Macrocephaly, Obesity
V	263216	del	chr11:84003279-84276072	0.27	exons 8-9	male	0 cnv	inherited from a parent with same phenotype	4	
VI	270892	del	chr11:84108622-84334253	0.23	exon 8	male	0 cnv	inherited from normal parent	NA	Aggressive behavior, Delayed speech and language development, Dysphasia, Intellectual disability, Stereotypic behavior
VII	272251	del	chr11:83805117-84215024	0.41	exons 9-13	female	3 cnvs: 225kbp dup and 278kbp dup on chr9, 148kbp dup on chr11. Genes: TRPM3, TMEM2, EHF.	inherited from an unaffected mother	NA	Aggressive behavior, Anxiety, Attention deficit hyperactivity disorder, Autism, Autoagression, Delayed speech and language development, Hallucinations, Increased body weight, Mild global developmental delay, Motor delay, Precocious puberty in females, Specific learning disability, Strabismus
VIII	273969	del	chr11:83996254-84214903	0.22	exon 9	male	0 cnv	unknown	NA	
IX	278011	del	chr11:84367238-84721340	0.35	exon 7	male	1 cnv: 300kbp del on chr17 variant inherited from normal parent. Genes: SLC39A11.	de novo constitutive	3	Delayed speech and language development, Hyperactivity, Low frustration tolerance
X	281197	del	chr11:84085773-84477088	0.39	exon 8	male	0 cnv	inherited from an unaffected mother	9	Autism spectrum disorder, Intellectual disability, moderate

XI	284804	del	chr11:84046644-84539636	0.49	exon 8	male	0 cnv	de novo constitutive	4	Autism, Delayed speech and language development
XII	286641	del	chr11:84291759-84477088	0.19	intron 7	male	1 cnv: 290kbp dup on chr17 inherited from mother. 22 genes.	inherited from an unaffected mother	NA	
XIII	288027	del	chr11:84046530-84454687	0.41	exon 8	male	1 cnv: 130kbp dup on chr7 inherited from mother. Gene: AKAP9.	inherited from an unaffected mother	NA	Autism, Developmental regression
XIV	288501	del	chr11:84334017-84595634	0.26	intron 7	female	1 cnv: 180kbp del on chr1. Inheritance unknown. Genes: INPP5B, MTF1, SF3A3.	unknown	NA	Abnormality of the palate, Short stature
XV	288842	del	chr11:84334017-84595634	0.26	intron 7	unknown	1 cnv: 541kbp del on chr16. Inheritance unknown. 28 genes.	unknown	NA	Abnormality of the eyelid, Premature birth
XVI	289734	del	chr11:84595575-84907579	0.31	exons 5-7	unknown	2 cnvs: 191kbp del on chr1 and 101kbp del on chr12; both inherited from mother. Genes: SUMF1, PIK3C2G, RERGL.	de novo constitutive	NA	Autism, Intellectual disability
XVII	292620	del	chr11:84046614-84214762	0.17	intron 8	female	0 cnv	unknown	3	Dysphasia
XVIII	300042	del	chr11:84046614-84419502	0.37	exon 8	unknown	0 cnv	unknown	NA	Abnormal facial shape, Cutaneous finger syndactyly, Global developmental delay, Hearing abnormality

XIX	300109	del	chr11:84419443-84581292	0.16	intron 7	unknown	0 cnv	inherited from a mother of unknown phenotype	NA	Cognitive impairment
XX	300111	del	chr11:84367238-84539665	0.17	intron 7	unknown	0 cnv	inherited from a mother of unknown phenotype	NA	Intellectual disability

Table S8: Summary of genetic and clinical descriptions of DECIPHER patients.

**Ref**	**Vulto-van Silfhout *et al.* Patient ID**	**CNV type**	***DLG2* CNV hg19 coordinates**	**CNV size (Mb)**	***DLG2* deleted features**	**Gender**	**Other rare CNVs**	**Inheritance of *DLG2* variant**	**Age of presentation**	**Clinical description**
XXI	1339	del	chr11:83595987-84489649	0.89	exons 8-18	unknown	1 cnv: 5Mbp dup on chr15; de novo. Several genes.	de novo constitutive	NA	Mild ID, autism, epilepsy

Table S9: Summary of genetic and clinical descriptions of Vulto-van Silfhout et al patient.

**Ref**	**Literature Patient ID**	**CNV type**	***DLG2* CNV hg19 coordinates**	**CNV size (Mb)**	***DLG2* deleted features**	**Gender**	**Other rare CNVs**	**Inheritance of *DLG2* variant**	**Age of presentation**	**Clinical description**
Walsh *et al.*	L4	del	chr11:84003321-84266329	0.26	exons 8-9		NA	unknown	25	Schizophrenia
Xu *et al.*	L5	del	chr11:83945764-84214964	0.27	exons 9-11	male	NA	inherited from an unaffected mother		Schizophrenia
Kirov *et al.*	L6	del	chr11:83795102-84165325	0.37	exons 9-13		NA	de novo constitutive	18	Schizophrenia
Kirov *et al.*	L7	del	chr11:84328458-84548416	0.22	intron 7		NA	de novo constitutive	20	Schizophrenia
Noor *et al.*.	L8	del	chr11:84143697-84312722	0.17	exon 8		1 cnv: 41kbp del on chr1. Gene: DNAJC6.	unknown		Borderline personality disorder
Noor *et al.*	L9	del	chr11:84111384-84354568	0.24	exon 8		1 cnv: 23kbp dup on chr20. No gene.	unknown		Borderline personality disorder

Nithian *et al.*	L11	del	chr11:83961633-84633847	0.67	exons 8-11	male	NA	inherited from an unaffected mother		Schizophrenia
Nithian *et al.*	L13	del	chr11:84375859-84521180	0.145	intron 7	female	NA	unknown		Schizophrenia

Table S10: Summary of genetic and clinical descriptions of literature patients.

**CLINICAL SUMMARY**	**PATIENT 1 (317136) MALE**	**PATIENT 2 (317185) MALE**
PRIMARY COMPLAINT	• developmental delay	• developmental delay
AGE AT PRESENTATION	• 3 years	• 1 year 5 months
PRESENT ILLNESS	• motor milestone delay:<br>– sitting: 17m<br>– walking: 24m<br>• language delay:<br>– first words: 2y11m<br>– first phrases: 3y11m<br>• repetitive gestures<br>• social interaction deficit:<br>– excessive timidity but good visual contact<br>– lack of participation in school activities, frequent crying<br>• executive slowness	• motor milestone mild delay:<br>– sitting: 8m<br>– walking: 18m<br>• language delay:<br>– first words: 18m<br>– first phrases: 5y<br>• rhythmic movements of the trunk<br>• social interaction deficit:<br>– lack of visual contact<br>– lack of facial expression<br>– lack of exploratory behavior<br>• general hypotonia
PAST HISTORY<br>• Obstetric	• pregnancy without incident<br>• full term delivery	• pregnancy without incident<br>• full term delivery
• Medical	• birth without incident<br>• normal birth weight<br>• normal sleep and eating habits<br>• recurrent otitis media<br>• around 18 months: episode of loss of contact and deviation of the mouth during 10-15 minutes; spontaneous resolution without recurrence	• apparent rigidity at birth (Apgar scores 7,8,10)<br>• normal birth weight<br>• congenital microcephaly<br>• normal sleep and eating habits<br>• recurrent otitis media with bilateral tympanic tube insertion<br>• bilateral hypermetropia
FAMILY HISTORY	• born to healthy unrelated adults<br>• 3rd child in a sibship of three<br>• two older asymptomatic female siblings	• born to healthy unrelated adults<br>• 2nd child in a sibship of three<br>• one older and one younger asymptomatic male sibling

PHYSICAL FINDINGS	• normal neurological examination<br>• no dysmorphic features<br>• no cutaneous abnormalities	• microcephaly (HC $<$ P3)<br>• generalized hypotonia, no other abnormal neurological findings<br>• low-set right ear with under-folded helix<br>• no cutaneous abnormalities
ADDITIONAL FINDINGS		
• Auditory Testing	• No significant conduction deficit	• No significant conduction deficit
• CNS Evoked Potentials	• BAER: no abnormal findings	• BAER: no abnormal findings<br>• SEP: delayed spinal cord brainstem transmission
• Overnight EEG	• no abnormal findings	• no abnormal findings
• Head MRI	• no abnormal findings	• small cranial size
• Metabolic Workup	• no abnormal findings	• no abnormal findings
GENETIC TESTING		
• Proband Microarray CGH	• 523kbp deletion at band 11q14.1 including *DLG2*	• 463kbp deletion at band 11q14.1 including *DLG2*
• Parental Microarray CGH	• deletion present in asymptomatic female parent	• deletion present in asymptomatic female parent
COGNITIVE TESTING		
• WPPSI-R	• 65 (age of 6)	• 62 (age of 6)
CLINICAL DEVELOPMENT	• persistent developmental delay despite cognitive and motor progress:<br>– diagnosis of mild ID	• persistent developmental delay despite cognitive and motor progress:<br>– diagnosis of mild ID

Table S11: Clinical description of ULB patients.

seqnames	start	end	variantaccession
chr11	84556678	84593655	esv2759844
chr11	84555145	84555203	esv1506583
chr11	84536933	84571495	nsv8849
chr11	84536635	84571784	nsv468767
chr11	84267185	84345829	nsv468766
chr11	84160786	84255107	nsv508642
chr11	84541974	84577299	esv2760388
chr11	84541974	84577299	esv2760388
chr11	84206126	84239486	esv2761687
chr11	84349593	84371598	esv2761689
chr11	84530538	84571784	dgv2039n54
chr11	84267185	84345829	nsv555614
chr11	84537700	84570874	esv2673432
chr11	84537700	84570874	esv2673432
chr11	84094401	84098289	esv2664740
chr11	84213153	84213373	esv2744840
chr11	84213273	84213455	esv2740842
chr11	84554999	84555317	esv2744842
chr11	84213153	84213373	esv2744840
chr11	84554999	84555317	esv2744842
chr11	84555118	84555257	esv2744844
chr11	84554999	84555317	esv2744842
chr11	84555022	84555163	esv2744843
chr11	84213153	84213373	esv2744840

Table S12: DGV deletions overlapping *DLG2* 7-9 region (hg19).

seqnames	start	end
chr11	83965527	84120008
chr11	83965910	84045055
chr11	84018549	84027950
chr11	84029240	84051838
chr11	84046978	84095887
chr11	84094421	84098238
chr11	84101233	84122523
chr11	84110959	84115246
chr11	84135802	84196003
chr11	84136420	84230443
chr11	84189137	84228421
chr11	84472654	84509360
chr11	84490026	84507903
chr11	84537679	84570855
chr11	84575564	84625713

Table S13: Deletions overlapping *DLG2* 7-9 region from the 1000 Genome Project [10].

id	start	end	id	start	end	id	start	end	id	start	end
208	83166048	83170967	156	84058581	84059194	104	84213611	84217237	52	84835578	84835890
207	83173045	83173136	155	84059345	84060015	103	84217475	84219428	51	84836103	84838280
206	83175111	83175718	154	84062879	84063896	102	84219616	84220940	50	84838340	84845118
205	83177751	83177860	153	84065823	84066380	101	84221712	84223461	49	84845822	84854674
204	83179308	83179849	152	84066660	84067047	100	84223737	84224035	48	84856693	84858878
203	83180244	83180416	151	84070032	84070848	99	84224424	84224738	47	84859057	84860998
202	83182669	83182770	150	84071116	84071820	98	84225244	84225497	46	84861180	84863284
201	83183770	83183820	149	84076884	84077092	97	84226681	84226927	45	84863511	84865137
200	83184305	83184592	148	84077414	84077630	96	84228196	84237624	44	84865296	84865695
199	83185017	83185805	147	84078295	84078975	95	84238051	84244873	43	84984025	84984288
198	83187271	83190905	146	84080571	84081177	94	84245003	84246142	42	84987520	84987853
197	83191000	83204351	145	84081609	84081947	93	84246654	84247009	41	84987978	84988346
196	83204621	83209700	144	84088041	84088247	92	84247445	84247890	40	84991578	84991656
195	83209810	83210787	143	84089175	84089801	91	84251408	84251808	39	84991734	84991853
194	83211072	83214376	142	84089896	84090165	90	84252628	84252865	38	84995412	84995764
193	83214521	83218447	141	84090335	84091083	89	84253177	84253615	37	84996264	84997264
192	83218888	83219296	140	84091252	84091470	88	84254099	84254323	36	84997401	84998256
191	83219588	83221251	139	84091803	84092282	87	84255125	84256635	35	84998318	84999222
190	83221426	83221873	138	84092742	84097297	86	84257112	84258026	34	85000366	85001342
189	83222130	83222748	137	84097510	84098321	85	84258881	84259162	33	85001626	85002207
188	83222966	83223560	136	84098518	84100348	84	84262647	84263094	32	85005026	85005065
187	83223753	83224949	135	84100776	84102495	83	84264446	84264958	31	85005860	85007593
186	83225397	83226075	134	84102893	84103156	82	84265477	84266174	30	85007982	85008436
185	83228068	83228369	133	84103209	84104310	81	84267001	84268568	29	85009217	85011638
184	83228726	83243827	132	84106764	84107098	80	84268842	84272965	28	85013280	85016016
183	83252725	83252901	131	84107221	84110282	79	84273114	84273398	27	85016790	85019888
182	83344254	83344908	130	84111158	84111673	78	84273961	84280534	26	85020029	85020067
181	83391321	83391808	129	84113217	84113679	77	84280850	84282212	25	85020741	85023331
180	83393201	83393486	128	84116119	84116369	76	84282492	84286266	24	85023608	85031436
179	83447540	83447829	127	84116797	84117004	75	84286479	84288497	23	85031550	85033747
178	83451567	83452219	126	84119088	84119335	74	84288789	84289789	22	85033811	85037720
177	83468595	83468950	125	84119593	84121395	73	84291490	84292423	21	85039780	85040387
176	83470152	83470423	124	84122366	84128658	72	84293736	84295467	20	85041894	85043701
175	83476547	83476813	123	84129031	84129559	71	84295891	84297686	19	85050723	85051908
174	83488800	83489038	122	84129677	84130047	70	84297877	84302215	18	85052711	85053919
173	83496824	83497126	121	84130154	84131269	69	84303202	84303573	17	85054113	85057436
172	83497733	83497835	120	84132374	84135688	68	84305217	84307263	16	85057632	85058729
171	83544657	83544813	119	84135770	84136486	67	84307470	84311097	15	85061297	85066111
170	83585463	83585531	118	84136632	84137616	66	84311615	84330226	14	85066192	85088840
169	83641371	83641526	117	84138299	84138544	65	84330332	84330884	13	85088961	85119171
168	83673928	83674066	116	84138850	84139120	64	84331032	84336001	12	85119623	85121383
167	83676367	83676511	115	84139281	84140797	63	84336063	84337949	11	85121462	85163989
166	83691549	83691686	114	84141500	84142049	62	84338067	84352990	10	85164071	85173498
165	83770358	83770527	113	84143140	84143589	61	84353142	84373352	9	85173630	85175979
164	83809966	83810090	112	84143852	84144837	60	84373667	84376890	8	85176114	85191724
163	83874504	83874554	111	84145382	84145919	59	84377059	84422387	7	85191837	85194932
162	83962281	83962335	110	84146499	84155193	58	84422484	84425704	6	85195320	85204708
161	84027868	84028380	109	84157412	84160769	57	84425769	84827382	5	85204888	85214258
160	84055925	84056266	108	84161142	84162397	56	84827560	84831598	4	85214442	85244495
159	84056574	84056836	107	84164038	84168890	55	84831661	84832309	3	85244553	85293677
158	84057339	84057700	106	84168981	84209505	54	84832665	84833789	2	85294291	85309833
157	84058130	84058491	105	84209940	84212795	53	84834817	84835404	1	85337643	85339790

Table S14: Fetal brain de novo transcriptome assembly coordinates of *DLG2* (antisense gene). They are coordinate of the exons belonging to the middle panel in Figure S54.

id	start	end	3'-end	5'-end	UCSC
208	83166048	83170967	+	=	34
207	83173045	83173136	−	=	33
205	83177751	83177860	=	=	32
203	83180244	83180416	=	=	31
202	83182669	83182770	=	=	30
201	83183770	83183820	=	=	29
197	83191000	83204351	+	+	28, 27, 26
184	83228726	83243827	+	+	25
183	83252725	83252901	=	=	24
182	83344254	83344908	−	+	23
180	83393201	83393486	=	+	22
172	83497733	83497835	−	=	21
171	83544657	83544813	=	=	20
170	83585463	83585531	=	=	19
169	83641371	83641526	=	=	18
168	83673928	83674066	=	=	17
167	83676367	83676511	=	=	16
166	83691549	83691686	=	+	15
165	83770358	83770527	=	=	14
164	83809966	83810090	=	=	13
163	83874504	83874554	=	=	12
162	83962281	83962335	=	+	11
161	84027868	84028380	=	−	9
94	84245003	84246142	+	+	8
57	84425769	84827382	+	+	7, 6
44	84865296	84865695	+	=	5
37	84996264	84997264	=	+	4
2	85294291	85309833	+	+	3
1	85337643	85339790	−	+	2, 1

Table S15: Fetal brain de novo (DN) transcriptome assembly coordinates overlapping with UCSC exons. *id*, *start*, *end* columns refer to the DN transcriptome; *UCSC* columns report the exon number as defined in Table S1. With respect to the directionality of the gene transcription, *3'-end* describes whether the DN exon 3'-end is before (-), after (+) or coincides (=) the UCSC one, *5'-end* describes whether the DN exon 5'-end is before (+), after (-) or coincides (=) the UCSC one. For example, the DN exon 197 has its 3'-end (83191000) after the UCSC exon 28 3'-end (83191415) (*3'-end*: +) and has its 5'-end (83204351) before the UCSC exon 26 5'-end (83195271) (*5'-end*: +). Remember that the words "before" and "after" are used in the context of transcription directionality and not genomic coordinates, and *DLG2* is an antisense gene.

id	start	end	3'-end	5'-end	Ensembl
208	83166048	83170967	+	=	47
207	83173045	83173136	−	−	46
205	83177751	83177860	=	=	45
203	83180244	83180416	=	=	44
202	83182669	83182770	=	=	43
201	83183770	83183820	=	=	42
197	83191000	83204351	+	+	41, 40, 39, 38
184	83228726	83243827	+	+	37
183	83252725	83252901	=	=	36
182	83344254	83344908	−	+	35
180	83393201	83393486	=	+	32
172	83497733	83497835	−	=	29
171	83544657	83544813	=	=	28
170	83585463	83585531	=	=	27
169	83641371	83641526	=	=	26
168	83673928	83674066	=	=	25
167	83676367	83676511	=	=	24
166	83691549	83691686	=	+	23
165	83770358	83770527	=	=	22
164	83809966	83810090	=	=	21
163	83874504	83874554	=	=	20
162	83962281	83962335	=	+	18
161	84027868	84028380	=	−	15
110	84146499	84155193	+	+	14
94	84245003	84246142	+	+	13
59	84377059	84422387	+	+	12
57	84425769	84827382	+	+	11, 10
50	84838340	84845118	+	+	9
44	84865296	84865695	+	=	8
37	84996264	84997264	=	+	7
8	85176114	85191724	+	+	6
4	85214442	85244495	+	+	5
2	85294291	85309833	+	+	4
1	85337643	85339790	−	+	2, 1

Table S16: Fetal brain de novo (DN) transcriptome assembly coordinates overlapping with Ensembl exons. Same application of Table S15.

HPO phenotype name
Intellectual disability, mild
Intellectual disability
Intellectual disability, profound
Intellectual disability, moderate
Intellectual disability, progressive
Intellectual disability, borderline
Intellectual disability, severe
Global developmental delay
Mild global developmental delay
Moderate global developmental delay
Severe global developmental delay
Delayed speech and language development
Autism
Autism spectrum disorder
Autistic behavior
Autism with high cognitive abilities
Expressive language delay
Language impairment
Dysphasia
Specific learning disability
Seizures
Generalized seizures
Generalized tonic-clonic seizures
Cognitive impairment
Stereotypic behavior
Epileptic encephalopathy
Abnormality of higher mental function
Behavioural/Psychiatric Abnormality
Behavioural/psychiatric abnormality
Psychosis

Table S17: Selected HPO phenotypes for neurodevelopmental disorders.

Brain tissue list from FANTOM5 dataset
substantia nigra
hippocampus
cerebellum
amygdala
medial temporal gyrus
pineal gland
insula
pituitary gland
parietal lobe
medial frontal gyrus
frontal lobe
paracentral gyrus
brain
nucleus accumbens
postcentral gyrus
occipital lobe
pons
locus coeruleus
medulla oblongata
temporal lobe
parietal cortex
occipital pole
occipital cortex
cerebral meninges
spinal cord
caudate nucleus
putamen
corpus callosum
globus pallidus
iPS differentiation to neuron

Table S18: Brain tissue list from FANTOM5 dataset, downloaded from UCSC.

Tissue (ENCODE ID)	BAM file name
Cerebellum (ENCSR000AEW)	ENCFF113PDT<br>ENCFF513CAU<br>ENCFF602BYA<br>ENCFF753KWK
Diencephalon (ENCSR000AEX)	ENCFF226SQK<br>ENCFF331ZDV<br>ENCFF391XTO<br>ENCFF677URC
Frontal cortex (ENCSR000AEY)	ENCFF220QDT<br>ENCFF741AGL<br>ENCFF803AAV<br>ENCFF907ILS
Occipital lobe (ENCSR000AFD)	ENCFF229XLD<br>ENCFF397QLJ<br>ENCFF746HOG
Parietal lobe (ENCSR000AFE)	ENCFF041THY<br>ENCFF243QVH<br>ENCFF434OIG<br>ENCFF892QFE
Temporal lobe (ENCSR000AFJ)	ENCFF286ZEF<br>ENCFF633PEY<br>ENCFF927WKN<br>ENCFF954GHY

Table S19: Fetal brain BAM file names and their corresponding tissues and ENCODE ID.

Tissue (ENCODE ID)	BAM file name
Midbrain (ENCSR255SDF)	ENCFF188SSW<br>ENCFF411UVC
Forebrain (ENCSR723SZV)	ENCFF203VYY<br>ENCFF965MHF
Hindbrain (ENCSR749BAG)	ENCFF172HHP<br>ENCFF772VMM

Table S20: Mouse newborn brain BAM file names and their corresponding tissues and ENCODE ID.

#	chr	start	end	width	gene name	CEGA score	ss start	ss end	type
1	chr1	7763249	7766656	3408	*CAMTA1*	NA	7764954	7764955	N
2	chr1	17239490	17242407	2918	*CROCC*	NA	17240632	17240633	N
3	chr1	93248904	93251568	2665	*EVI5*	NA	93250392	93250393	P
4	chr1	147717397	147718316	920	*NBPF8*	NA	147718153	147718154	N
5	chr1	204318719	204320752	2034	*PLEKHA6*	144	204320007	204320008	N
6	chr2	236577649	236583540	5892	*AGAP1*	31	236579701	236579702	P
7	chr3	11759850	11761105	1256	*VGLL4*	NA	11760890	11760891	P
8	chr3	114167766	114174803	7038	*ZBTB20*	832	114173425	114173426	P
9	chr4	183368933	183373052	4120	*TENM3*	NA	183370236;183370567	183370237;183370568	P;N
10	chr4	1240956	1242536	1581	*CTBP1*	NA	1242448	1242449	P
11	chr4	176812194	176813234	1041	*GPM6A*	NA	176812613	176812614	P and E
12	chr5	14440397	14444098	3702	*TRIO*	192	14441469	14441470	P
13	chr5	58722748	58727155	4408	*PDE4D*	300	58726119	58726120	N
14	chr7	75266093	75269827	3735	*HIP1*	144	75268368	75268369	N
15	chr7	143577564	143580388	2825	*FAM115A*	NA	143579015;143579828	143579016;143579829	E;P
16	chr8	12808153	12813083	4931	*KIAA1456*	NA	12809867	12809868	P and E
17	chr8	145686236	145688964	2729	*CYHR1*	56	145687665	145687666	P
18	chr9	20683184	20685987	2804	*FOCAD*	41	20684292	20684293	P
19	chr10	13387098	13389957	2860	*SEPHS1*	NA	13389276	13389277	P
20	chr11	84147024	84149361	2338	*DLG2*	557	84148430	84148431	P
21	chr11	84429842	84432885	3044	*DLG2*	97	84431338	84431339	N
22	chr11	84843131	84844944	1814	*DLG2*	862	84843811	84843812	P
23	chr16	53163420	53165233	1814	*CHD9*	NA	53164970	53164971	P
24	chr16	49888603	49890008	1406	*ZNF423*	65	49889645	49889646	P
25	chr17	61227923	61231987	4065	*TANC2*	194	61228741	61228742	E
26	chr17	56708405	56710156	1752	*TEX14*	NA	56708994	56708995	N
27	chr21	17790619	17798315	7697	*LINC00478*	NA	17792061;17792892	17792062;17792893	P;P
28	chr22	28832791	28840308	7518	*TTC28*	553	28838873	28838874	P
29	chr22	36355185	36358538	3354	*RBFOX2*	NA	36357610	36357611	P

Table S21: Intronic regions harbouring putative novel promoters, detected by the knowledge-driven genome-wide analysis. *CEGA score* documents the conservation score across vertebrates as reported in Conserved Elements from Genomics Alignments database [11]. *ss start* and *ss end* represent the coordinate of the detected splicing site. Column *type* reports whether such region is predicted as promoter (P) or exon (E) by Ensembl (archive 75, feb 2014), or novel (N); entries *9*, *15* and *27* includes two detected splicing site, while entries *11* and *16* the is predicted as either an exon or a promoter. Coordinates are in hg19.

Name	Position
CAGE10	chr11:84148565-84148597
CAGE09	chr11:84148598-84148663
CAGE08	chr11:84148843-84148867
CAGE07	chr11:84148869-84148881
CAGE06	chr11:84431432-84431459
CAGE05	chr11:84431459-84431498
CAGE04	chr11:84431757-84431819
CAGE03	chr11:84431828-84431834
CAGE02	chr11:84431891-84431908
CAGE01	chr11:84431943-84431992

Table S22: FANTOM5 robust CAGE peaks coordinates in HPs.

Supplementary Note 1

Genome-wide statistical assessment of HPs

We assessed the statistical significance of HPs, as linked to NDDs, by employing DECIPHER and GDD/ID cohorts in a genome-wide analysis using two strategies: a data-driven approach, checking up on other statistically enriched intronic regions, and a knowledge-driven approach, which is based on functional data known to be associated with promoters (Figures S86 and S87 report an overview of the analyses designs). Because DECIPHER enlists a broad spectrum of diseases, we name DECIPHER NDD the subset of DECIPHER patients with NDD phenotypes (Table S17 for the list of selected HPO phenotypes). The collection of DECIPHER NDD and GDD/ID case patients represents hereafter our case cohort; while the collection of DGV and GDD/ID control patients represents hereafter our control cohorts. For both cohorts, we only considered deletions. Because DGV dataset version of July 2015 also includes GDD/ID control patients, we remove the latter from the former, resulting in two disjoint populations and in 11987 individuals in DGV.

It is difficult to assess any gene-diseases association whenever deletions involve multiple genes. For such reason, we filtered out any aberration affecting multiple genes in the cases, while we left unaltered the controls. The result is a selection of 6023 patients with NDD phenotype(s) and harbouring some (1 or more) monogenic deletions; and a total of 31571 control individuals harbouring deletions: 11987 from DGV and 19584 from GDD/ID control cohorts using the *alternative* approach (see Supplementary Note 2 for the introduction of *alternative* and *traditional* approaches).

Using such cohorts, we identified 293186 intronic Smallest Regions of Overlap (SROs), defined as disjoint ranges of the overlapping deletions in both cohorts (Figure S56). Note that, at this stage, some SROs might overlap only control CNVs; these SROs will be filtered out by the enrichment analysis. In the data-driven approach, we inspect for SRO-NDD association by means of a two-step approach: first, a statistical assessment of the enrichment of cases over controls (see Supplementary Note 2), and, second, a visual inspection of the enriched regions using functional and genotypic-phenotypic datasets. This latter step is required in order to provide a biological explanation of the link between SRO and NDDs. Without context, some SROs results could be relevant only because the deletions overlapping the intronic regions also affect the nearby exons. In such scenario, one can conservatively assume exons to account for the disease, therefore making the intronic SRO a false positive. To avoid such false positives, we defined the relevance of intronic SROs for NDDs using two criteria: the intronic SRO enrichment of cases over controls using one-tailed Fisher's exact test is statistically significant (p-value < 0.01, after Bonferroni correction), and the resulting intronic p-value is 10x smaller than p-value calculated from the nearby exons (see Supplementary Note 2). All the resulting p-value significant SROs turned out false positives after manual inspection, due to the impossibility of providing a biological explanation of the region using ENCODE (fetal brain BAM files), Roadmap Epigenomics (NPC/fetal/adult brain) and FANTOM5 functional datasets (data not shown). Using the *traditional* approach, we got equivalent results.

We further screened the genome for promoters using a knowledge-driven strategy focused on introns. Among the overall 293186 SROs, we looked for the intronic regions that share the most similar functional profile to HPin7 and HPin8, i.e., a promoter profile. We began our genome-wide analysis by integrating Roadmap Epigenomics, ENCODE, FANTOM5 and TFs independent datasets. Considering the H3K4me3 regions overlapping any SRO, four criterias shaped our definition of intronic brain promoter: the presence of both TF binding sites and CAGE peaks, a H3K4me3/H3K4me1 peak ratio greater than one and the existence of a splicing site found in at least half of the fetal brain BAM files. We established the presence of splicing sites whenever the difference in reads coverage, of two adjacent nucleotides, was higher than a certain threshold (named hereafter *delta coverage*), in this case 20. It is worth noting that we searched for conserved or non-conserved regions, because some intronic promoters might be specific for the human brain. This genome-wide analysis resulted in the discovery of 29 intronic promoters: 21 predicted by Ensembl and 8 novels (Table S21). High level transcription and sharp splicing sites located in H3K4me3 peak regions are easily visualized in the fetal brain BAM files (see Figures S57 to S85). Table 3 (article) lists the 11 intronic promoters found deleted in NDD patients (Figure 6, article, and see Additional file 2). Both *DLG2* HPin7 and HPin8 are enriched in NDD cases versus controls in a statistically significant manner (p-value < 0.05 after Bonferroni correction, see Table 3, article); the other 9 have a greater number of case than control patients (normalized by the cohort sizes: 6023 cases and the sum of DGV and GDD/ID control individuals, 31571). Comparing the 29 intronic promoters, ten of the eleven deleted promoters are evolutionary conserved, in comparison to the 4 of the 18 remaining (one-tailed Fisher's exact test, p-value is $4.445 \cdot 10^{-04}$).

Regarding the association between HPs and NDDs, while the multiple hypotheses correction in the first strategy causes also HPs to be false positive, because of the high number of hypotheses tested (20767), in the

second approach HPs are still statistical significant due to the low number of hypotheses tested (11).

Supplementary Note 2

Data-driven analysis: cohorts

The data-driven analysis workflow includes the following steps: cohorts conception, identification of regions of interest (SROs in our case), statistical analysis (one-tailed Fisher's exact test), statistical correction due to multiple hypothesis testing (Bonferroni) and, finally, visual inspection of the candidate regions. As described in "Patients and Controls CNV datasets" section (main article), we retrieved DGV and GDD/ID datasets from UCSC. We wanted to have two independent control cohorts for the statistical analysis, hence, as preprocessing step, we pulled GDD/ID CNVs out of July 2015 version of DGV.

The statistical analysis is based on the number of patients, rather than CNVs, in the cohorts. On the other hand, the patient (or sample) information is missing in 99413 (1.7% of the total) CNVs from DGV and 115630 (90.9% of the total) CNVs from GDD/ID control, hence different choices in the cohorts conception would affect the final outcome. There are two possible approaches: consider only CNVs with patient information and merge the two control cohorts, resulting in 13621 healthy patients, or consider only CNVs with patient information from DGV (11987) and all CNVs in GDD/ID control, knowing the number of patients, 19584 [6]. We performed both, and for convenience we name the first traditional approach (Figure S86), the second alternative approach (Figure S87). While in the former settings all genotype-phenotype datasets go through the same preprocessing steps, in the latter we deal with all CNVs at the cost of one approximation: although we pull duplications out of the dataset, the number of patients stays constant.

The statistical analysis is then performed, in the traditional approach, with two populations: 6023 cases, 13621 controls; in the alternative approach, with three populations: 6023 cases, 11987 DGV control patients, 19584 GDD/ID control patients. The SRO is enriched in NDD patients if the number of overlapping cases are statistically significant with respect to controls; regarding the alternative approach, such condition must hold for both DGV and GDD/ID control cohorts, independently.

Data-driven analysis: SRO and exons

Every intronic Smallest Regions of Overlap (SROs, defined as disjoint ranges of the overlapping deletions in both cohorts, Figure S56) is surrounded by two exons and these three elements (individually or combined) could be important in understanding NDD. Hence, we measured four enrichments of cases over controls using one-tailed Fisher's exact test: the intronic SRO, the previous and next exons individually, and both exons combined. Therefore, the intronic SRO is deemed more relevant than its nearby exons if the following rules are matched: if deletions that affect the SRO also overlap both exons, then the SRO's p-value must be smaller than the other three p-values by a factor of 10; if deletions that affect the SRO also, and only, overlap the previous (next) exon, then the SRO's p-value must be smaller than the previous (next) exon's p-value by a factor of 10. Altogether, we opted for the following strict criteria: an intronic SRO is considered relevant when having its p-value significant by itself and smaller than the neighbor exons' p-values (by a factor of 10).

Knowledge-driven analysis

For each putative promoters and first exons found in intronic regions and listed in Table 3 (main article), we evalutate their statistical significance using the alternative approach.

Enrichment analysis

All statistical analysis have been performed using R software, version 3.2. In the knowledge-driven analysis, all p-values reported in Additional file 2, are adjusted via Bonferroni correction, with the total number of tested hypothesis of 11. In the data-driven strategy, before performing the statistical analysis, we selected the SROs where the number of case patients is greather than control individuals (normalized by the cohort size). The amount of selected SRO was 20767 or 16266, numbers used for the Bonferroni correction in both alternative and traditional data-driven analyses, respectively.

Supplementary Note 3

We performed the cross-annotation and mapping of *DLG2*/*Dlg2* exons using DNA and AA sequences. There is an evident lack of cross-annotation between reference genomes and reviewed proteins available, between UCSC and UniProtKB/Swiss-Prot. For this reason we mapped the amino-acid sequence to the underlying exon considering: i) the ordering of information, ii) possible reading frames and iii) the synteny properties of orthologous genes in humans and mice. The results are reported here below for both organisms. The exon regions are from UCSC, while amino-acid sequences are from UniProtKB/Swiss-Prot.

Because some amino-acids might be the result of three nucleotides shared between two exons, we used the following annotation to represent this information: "e3-D]" means the amino-acid "D" is shared between exon 3 and the current exon, similarly, "[S-e19" means amino-acid "S" is shared between the current exon and exon 19. Some exons might splice to two different exons, according to the isoform. We therefore represent this information as "[e11b—S-e12", that says this exon continue to exon 11b without sharing any amino-acid, or continue to exon 12 and they share amino-acid "S".

UniProt *DLG2* protein mapping to exons

```
exon 3       MGIFKSSLFQALL[D-e4
exon 4       e3-D]IQEFYEVTLLNSQKSCEQKIEEANQVLQKWEKTSLLAPCHDRLQKSSE
exon 5       LTDCSGSKENASCIEQNKENQSFENETDETTT
exon 6       QNQGRCPAQNCSVEAPAWMPVHHCT
exon 7       MFFACYCALRTNVK
CFEin7_cds   MSPVVKDPDCFTPMICHCKVACTNNTLSLMFGCK
exon 8       KYRYQDEDAPHDHSLPRLTHEVRGPELVHVSEKNLSQIENVHGYVLQSHISPLK
CFEin8_cds   MFASIWYAKKLGRRFVHNARKAKSEK
exon 9       MNAYLTKQHSCSRGSDGMDAVRSAPTLIRDAHCACGWQRNCQGLGYSSQTMPSSGPGG
             PASNRTGGSSFNRTLWDSVRKSPHKTSTKGKGTCGEHCTCPHGWFSPAQ
exon 10      MQRPSVSRAENYQLLWDTIASLKQCEQAMQHAFIP
exon 11      ASPAPIIVNTDTLDTIPY
exon 12      VNGTEIEYEFEEITLER
exon 13      GNSGLGFSIAGGTDNPHIGDDPGIFITKIIPGGAAAEDGRL[R-e14
exon 14      e13-R]VNDCILRVNEVDVSEVSHSKAVEALKEAGSIVRLYVRRRRPILETVVEIKLFKGPK[G-e15
exon 15      e14-G]LGFSIAGGVGNQHIPGDNSIYVTKIIDGGAAQKDGRLQVGDRLLM
exon 16      VNNYSLEEVTHEEAVAILKNTSEVVYLKVGKPTTIYMTDPYGPPDITH[S-e17
exon 17      e16-S]YSPPMENHLLSGNNGTLEYKTSLPPISPGRYSPIPKHMLVDDDYT[R-e18
exon 18      e17-R]PPEPVYSTVNKLCDKPASPRHYSPVECDKSFLLSAPYSHYHLGLLPDSEMT[S-e19
exon 19      e18-S]HSQHSTATRQPSMTLQRAVSLE[G-e20
exon 20      e19-D]EPRKVVLHKGSTGLGFNIVGGEDGEGIFVSFILAGGPADLSGELQRGDQILS
exon 21      VNGIDLRGASHEQAAAALKGAGQTVTIIAQYQPE[D-e23a
exon 23a     e21-D]YARFEAKIHDLREQ
exon 23b     MMNHSMSSGSGSLRTNQKRSLYV[R-e24
exon 24      e23b-R]AMFDYDKSKDSGLPSQGLSFKYGDILHVINASDDEWWQARRVMLEGDSEEMGVIPSKR[R-e25
exon 25      e24-R]VERKERARLKTVKFNAKPGVIDSKG
exon 26      SFNDKRKKSFIFSRKFPFYKNKEQSEQETSDPE[R-e29
exon 27      DIPGLGDDGYGTKTL[R-e29
exon 28 *
exon 29      e26-R|e27-R]GQEDLILSYEPVTRQE[I-e30
exon 30      e29-I]NYTRPVIILGPMKDRINDDLISEFPDKFGSCVP[H-e31
exon 31      e30-H]TTRPKRDYEVDGRDYHFVISREQMEKDIQEHKFIEAGQYNDNLYGTSVQSVRFVAER
exon 32      GKHCILDVSGNAIKRLQVAQLYPIAIFIKPRSLEPL[M-e33
exon 33      e32-M]EMNKRLTEEQAKKTYDRAIKLEQEFGEYFT[A-e34
exon 34      e33-A]IVQGDTLEDIYNQCKLVIEEQSGPFIWIPSKEKL
```

* UniProt database does not have a *DLG2* isoform sequence including exon 28, therefore we cannot firmly assess its coding sequence. However, considering it is a cassette exon between exons 27 and 29, our best guess is the following

```
exon 28    e27-K]HVSSNASDSESSY[R-e29
```

UniProt *Dlg2* protein mapping to exons

```
exon 1        MFFACYCALRTNVK
mCFEin7_cds   MICHCKVACTNNTLSLMFGCK
exon 2        KYRYQDEDGPHDHSLPRLTHEVRGPELVHVSEKNLSQIENVHGYVLQSHISPLK
mCFEin8_cds   MFASIWYAKKLGRRFVHNARKAKSEK
exon 3        MNAYLTKQHSCSRGSDGMDIGRSAPTLIRDAHCACGWQRNAQGLGYSSQTMPSSGPGGPASNRTK
              LVTLWDSVRKSPHKTSTKGKGNCGERCACPHGWFSPAQ
exon 4        ASPAPIIVNTDTLDTIPY
exon 5        VNGTEIEYEFEEITLER
exon 6        GNSGLGFSIAGGTDNPHIGDDPGIFITKIIPGGAAAEDGRL[R-e7
exon 7        e6-R]VNDCILRVNEVDVSEVSHSKAVEALKEAGSIVRLYVRRRRPILETVVEIKLFKGPK[G-e8
exon 8        e7-G]LGFSIAGGVGNQHIPGDNSIYVTKIIDGGAAQKDGRLQVGDRLLM
exon 9        VNNYSLEEVTHEEAVAILKNTSDVVYLKVGKPTTIYMTDPYGPPDITH[S-e10
exon 10       e9-S]YSPPMENHLLSGNNGTLEYKTSLPPISPGRYSPIPKHMLGEDDYT[R-e11
exon 11       e10-R]PPEPVYSTVNKLCDKPASPRHYSPVECDKSFLLSTPYPHYHLGLLPDSDMT[e11b|S-e12
exon 11b      RYCMRFLTSSSPVACVSTRMDGWNSSPPTSLALSTFLVERCSASMVRWEKLRTWLFCSFCCAH
exon 12       e11-S]HSQHSTATRQPSVTLQRAISLE[G-e15
exon 14 **
exon 15       e12-G]EPRKVVLHKGSTGLGFNIVGGEDGEGIFVSFILAGGPADLSGELQRGDQILS
exon 16       VNGIDLRGASHEQAAAALKGAGQTVTIIAQYQPE[D-s18a
exon 18a      e16-D]YARFEAKIHDLREQ
exon 18b      MMNHSMSSGSGSLRTNQKRSLYV[R-e19
exon 19       e18b-R]AMFDYDKSKDSGLPSQGLSFKYGDILHVINASDDEWWQARRVTLDGDSEEMGVIPSKR[R-e20
exon 20       e19-R]VERKERARLKTVKFNAKPGVIDSKG
exon 21 *     SFNDKRKKSFIFSRKFPFYKNKEQSEQETSDPE[R-e24
exon 22       DIPGLGDDGYGTKTL[R-e24
exon 23 **
exon 24       e21-R|e22-R]GQEDLILSYEPVTRQE[I-e25
exon 25       e24-I]NYTRPVIILGPMKDRINDDLISEFPDKFGSCVP[H-e26
exon 26       e25-H]TTRPKRDYEVDGRDYHFVISREQMEKDIQEHKFIEAGQYNDNLYGTSVQSVRFVAER
exon 27       GKHCILDVSGNAIKRLQVAQLYPIAIFIKPKSLEPL[M-e28
exon 28       e27-M]EMNKRLTEEQAKKTYDRAIKLEQEFGEYFT[A-e29
exon 29       e28-A]IVQGDTLEDIYNQCKLVIEEQSGPFIWIPSKEKL
```

* While UniProt database does not have a *Dlg2* isoform sequence including exon 21, its DNA sequence match to *DLG2* exon 26 according to NCBI BLAST. Once verified that one the three possible translation into amino-acids sequence corresponds to *DLG2* exon 26 coding sequence, we consider *Dlg2* exon 21 coding as *DLG2* exon 26.

** Exons 14 and 23 are coding exons, but UniProt database does not hold any isoform sequence in *Dlg2*. While exon 14 is not mapped to any human orthologous *DLG2* exons, exon 23 aligns to *DLG2* exon 28, for which we do not have the coding sequence either.

Supplementary Note 4

Using the exon-amino-acid mapping in Supplementary Note 3, we map every *DLG2*/*Dlg2* UniProtKB/Swiss-Prot isoform to exons.

UniProt *DLG2* isoforms inspection

Q15700-1

```
>sp|Q15700|DLG2_HUMAN Disks large homolog 2 OS=Homo sapiens GN=DLG2 PE=1 SV=3
MFFACYCALRTNVKKYRYQDEDAPHDHSLPRLTHEVRGPELVHVSEKNLSQIENVHGYVL
QSHISPLKASPAPIIVNTDTLDTIPYVNGTEIEYEFEEITLERGNSGLGFSIAGGTDNPH
IGDDPGIFITKIIPGGAAAEDGRLRVNDCILRVNEVDVSEVSHSKAVEALKEAGSIVRLY
VRRRRPILETVVEIKLFKGPKGLGFSIAGGVGNQHIPGDNSIYVTKIIDGGAAQKDGRLQ
VGDRLLMVNNYSLEEVTHEEAVAILKNTSEVVYLKVGKPTTIYMTDPYGPPDITHSYSPP
MENHLLSGNNGTLEYKTSLPPISPGRYSPIPKHMLVDDDYTRPPEPVYSTVNKLCDKPAS
PRHYSPVECDKSFLLSAPYSHYHLGLLPDSEMTSHSQHSTATRQPSMTLQRAVSLEGEPR
KVVLHKGSTGLGFNIVGGEDGEGIFVSFILAGGPADLSGELQRGDQILSVNGIDLRGASH
EQAAAALKGAGQTVTIIAQYQPEDYARFEAKIHDLREQMMNHSMSSGSGSLRTNQKRSLY
VRAMFDYDKSKDSGLPSQGLSFKYGDILHVINASDDEWWQARRVMLEGDSEEMGVIPSKR
RVERKERARLKTVKFNAKPGVIDSKGSFNDKRKKSFIFSRKFPFYKNKEQSEQETSDPER
GQEDLILSYEPVTRQEINYTRPVIILGPMKDRINDDLISEFPDKFGSCVPHTTRPKRDYE
VDGRDYHFVISREQMEKDIQEHKFIEAGQYNDNLYGTSVQSVRFVAERGKHCILDVSGNA
IKRLQVAQLYPIAIFIKPRSLEPLMEMNKRLTEEQAKKTYDRAIKLEQEFGEYFTAIVQG
DTLEDIYNQCKLVIEEQSGPFIWIPSKEKL
```

	start	end	width	seq	name
1	1	14	14	MFFACYCALRTNVK	exon 7
2	15	68	54	KYRYQDEDAPHDHSLPRLT..SQIENVHGYVLQSHISPLK	exon 8
3	69	86	18	ASPAPIIVNTDTLDTIPY	exon 11
4	87	103	17	VNGTEIEYEFEEITLER	exon 12
5	104	144	41	GNSGLGFSIAGGTDNPHIG..GIFITKIIPGGAAAEDGRL	exon 13
6	145	145	1	R	
7	146	201	56	VNDCILRVNEVDVSEVSHS..RRRPILETVVEIKLFKGPK	exon 14
8	202	202	1	G	
9	203	247	45	LGFSIAGGVGNQHIPGDNS..DGGAAQKDGRLQVGDRLLM	exon 15
10	248	295	48	VNNYSLEEVTHEEAVAILK..GKPTTIYMTDPYGPPDITH	exon 16
11	296	296	1	S	
12	297	341	45	YSPPMENHLLSGNNGTLEY..SPGRYSPIPKHMLVDDDYT	exon 17
13	342	342	1	R	
14	343	393	51	PPEPVYSTVNKLCDKPASP..LSAPYSHYHLGLLPDSEMT	exon 18
15	394	394	1	S	
16	395	416	22	HSQHSTATRQPSMTLQRAVSLE	exon 19
17	417	417	1	G	
18	418	469	52	EPRKVVLHKGSTGLGFNIV..AGGPADLSGELQRGDQILS	exon 20
19	470	503	34	VNGIDLRGASHEQAAAALKGAGQTVTIIAQYQPE	exon 21
20	504	504	1	D	
21	505	518	14	YARFEAKIHDLREQ	exon 23a
22	519	541	23	MMNHSMSSGSGSLRTNQKRSLYV	exon 23b
23	542	542	1	R	
24	543	600	58	AMFDYDKSKDSGLPSQGLS..RRVMLEGDSEEMGVIPSKR	exon 24
25	601	601	1	R	
26	602	626	25	VERKERARLKTVKFNAKPGVIDSKG	exon 25
27	627	659	33	SFNDKRKKSFIFSRKFPFYKNKEQSEQETSDPE	exon 26
28	660	660	1	R	
29	661	676	16	GQEDLILSYEPVTRQE	exon 29
30	677	677	1	I	
31	678	710	33	NYTRPVIILGPMKDRINDDLISEFPDKFGSCVP	exon 30

32	711	711	1	H	
33	712	768	57	TTRPKRDYEVDGRDYHFVI..YNDNLYGTSVQSVRFVAER	exon 31
34	769	804	36	GKHCILDVSGNAIKRLQVAQLYPIAIFIKPRSLEPL	exon 32
35	805	805	1	M	
36	806	835	30	EMNKRLTEEQAKKTYDRAIKLEQEFGEYFT	exon 33
37	836	836	1	A	
38	837	870	34	IVQGDTLEDIYNQCKLVIEEQSGPFIWIPSKEKL	exon 34

Q15700-2

```
>sp|Q15700-2|DLG2_HUMAN Isoform 2 of Disks large homolog 2 OS=Homo sapiens GN=DLG2
MGIFKSSLFQALLDIQEFYEVTLLNSQKSCEQKIEEANQVLQKWEKTSLLAPCHDRLQKS
SELTDCSGSKENASCIEQNKENQSFENETDETTTQNQGRCPAQNCSVEAPAWMPVHHCTK
YRYQDEDAPHDHSLPRLTHEVRGPELVHVSEKNLSQIENVHGYVLQSHISPLKASPAPII
VNTDTLDTIPYVNGTEIEYEFEEITLERGNSGLGFSIAGGTDNPHIGDDPGIFITKIIPG
GAAAEDGRLRVNDCILRVNEVDVSEVSHSKAVEALKEAGSIVRLYVRRRRPILETVVEIK
LFKGPKGLGFSIAGGVGNQHIPGDNSIYVTKIIDGGAAQKDGRLQVGDRLLMVNNYSLEE
VTHEEAVAILKNTSEVVYLKVGKPTTIYMTDPYGPPDITHSYSPPMENHLLSGNNGTLEY
KTSLPPISPGRYSPIPKHMLVDDDYTRPPEPVYSTVNKLCDKPASPRHYSPVECDKSFLL
SAPYSHYHLGLLPDSEMTSHSQHSTATRQPSMTLQRAVSLEGEPRKVVLHKGSTGLGFNI
VGGEDGEGIFVSFILAGGPADLSGELQRGDQILSVNGIDLRGASHEQAAAALKGAGQTVT
IIAQYQPEDYARFEAKIHDLREQMMNHSMSSGSGSLRTNQKRSLYVRAMFDYDKSKDSGL
PSQGLSFKYGDILHVINASDDEWWQARRVMLEGDSEEMGVIPSKRRVERKERARLKTVKF
NAKPGVIDSKGSFNDKRKKSFIFSRKFPFYKNKEQSEQETSDPERGQEDLILSYEPVTRQ
EINYTRPVIILGPMKDRINDDLISEFPDKFGSCVPHTTRPKRDYEVDGRDYHFVISREQM
EKDIQEHKFIEAGQYNDNLYGTSVQSVRFVAERGKHCILDVSGNAIKRLQVAQLYPIAIF
IKPRSLEPLMEMNKRLTEEQAKKTYDRAIKLEQEFGEYFTAIVQGDTLEDIYNQCKLVIE
EQSGPFIWIPSKEKL
```

	start	end	width	seq	name
1	1	13	13	MGIFKSSLFQALL	exon 3
2	14	14	1	D	
3	15	62	48	IQEFYEVTLLNSQKSCEQK..WEKTSLLAPCHDRLQKSSE	exon 4
4	63	94	32	LTDCSGSKENASCIEQNKENQSFENETDETTT	exon 5
5	95	119	25	QNQGRCPAQNCSVEAPAWMPVHHCT	exon 6
6	120	173	54	KYRYQDEDAPHDHSLPRLT..SQIENVHGYVLQSHISPLK	exon 8
7	174	191	18	ASPAPIIVNTDTLDTIPY	exon 11
8	192	208	17	VNGTEIEYEFEEITLER	exon 12
9	209	249	41	GNSGLGFSIAGGTDNPHIG..GIFITKIIPGGAAAEDGRL	exon 13
10	250	250	1	R	
11	251	306	56	VNDCILRVNEVDVSEVSHS..RRRPILETVVEIKLFKGPK	exon 14
12	307	307	1	G	
13	308	352	45	LGFSIAGGVGNQHIPGDNS..DGGAAQKDGRLQVGDRLLM	exon 15
14	353	400	48	VNNYSLEEVTHEEAVAILK..GKPTTIYMTDPYGPPDITH	exon 16
15	401	401	1	S	
16	402	446	45	YSPPMENHLLSGNNGTLEY..SPGRYSPIPKHMLVDDDYT	exon 17
17	447	447	1	R	
18	448	498	51	PPEPVYSTVNKLCDKPASP..LSAPYSHYHLGLLPDSEMT	exon 18
19	499	499	1	S	
20	500	521	22	HSQHSTATRQPSMTLQRAVSLE	exon 19
21	522	522	1	G	
22	523	574	52	EPRKVVLHKGSTGLGFNIV..AGGPADLSGELQRGDQILS	exon 20
23	575	608	34	VNGIDLRGASHEQAAAALKGAGQTVTIIAQYQPE	exon 21
24	609	609	1	D	
25	610	623	14	YARFEAKIHDLREQ	exon 23a
26	624	646	23	MMNHSMSSGSGSLRTNQKRSLYV	exon 23b
27	647	647	1	R	

	start	end	width	seq	name
28	648	705	58	AMFDYDKSKDSGLPSQGLS..RRVMLEGDSEEMGVIPSKR	exon 24
29	706	706	1	R	
30	707	731	25	VERKERARLKTVKFNAKPGVIDSKG	exon 25
31	732	764	33	SFNDKRKKSFIFSRKFPFYKNKEQSEQETSDPE	exon 26
32	765	765	1	R	
33	766	781	16	GQEDLILSYEPVTRQE	exon 29
34	782	782	1	I	
35	783	815	33	NYTRPVIILGPMKDRINDDLISEFPDKFGSCVP	exon 30
36	816	816	1	H	
37	817	873	57	TTRPKRDYEVDGRDYHFVI..YNDNLYGTSVQSVRFVAER	exon 31
38	874	909	36	GKHCILDVSGNAIKRLQVAQLYPIAIFIKPRSLEPL	exon 32
39	910	910	1	M	
40	911	940	30	EMNKRLTEEQAKKTYDRAIKLEQEFGEYFT	exon 33
41	941	941	1	A	
42	942	975	34	IVQGDTLEDIYNQCKLVIEEQSGPFIWIPSKEKL	exon 34

Q15700-3

>sp|Q15700-3|DLG2_HUMAN Isoform 3 of Disks large homolog 2 OS=Homo sapiens GN=DLG2
MQRPSVSRAENYQLLWDTIASLKQCEQAMQHAFIPVNGTEIEYEFEEITLERGNSGLGFS
IAGGTDNPHIGDDPGIFITKIIPGGAAAEDGRLRVNDCILRVNEVDVSEVSHSKAVEALK
EAGSIVRLYVRRRRPILETVVEIKLFKGPKGLGFSIAGGVGNQHIPGDNSIYVTKIIDGG
AAQKDGRLQVGDRLLMVNNYSLEEVTHEEAVAILKNTSEVVYLKVGKPTTIYMTDPYGPP
DITHSYSPPMENHLLSGNNGTLEYKTSLPPISPGRYSPIPKHMLVDDDYTSHSQHSTATR
QPSMTLQRAVSLEGEPRKVVLHKGSTGLGFNIVGGEDGEGIFVSFILAGGPADLSGELQR
GDQILSVNGIDLRGASHEQAAAALKGAGQTVTIIAQYQPEDYARFEAKIHDLREQMMNHS
MSSGSGSLRTNQKRSLYVRAMFDYDKSKDSGLPSQGLSFKYGDILHVINASDDEWWQARR
VMLEGDSEEMGVIPSKRRVERKERARLKTVKFNAKPGVIDSKGDIPGLGDDGYGTKTLRG
QEDLILSYEPVTRQEINYTRPVIILGPMKDRINDDLISEFPDKFGSCVPHTTRPKRDYEV
DGRDYHFVISREQMEKDIQEHKFIEAGQYNDNLYGTSVQSVRFVAERGKHCILDVSGNAI
KRLQVAQLYPIAIFIKPRSLEPLMEMNKRLTEEQAKKTYDRAIKLEQEFGEYFTAIVQGD
TLEDIYNQCKLVIEEQSGPFIWIPSKEKL

	start	end	width	seq	name
1	1	35	35	MQRPSVSRAENYQLLWDTIASLKQCEQAMQHAFIP	exon 10
2	36	52	17	VNGTEIEYEFEEITLER	exon 12
3	53	93	41	GNSGLGFSIAGGTDNPHIG..GIFITKIIPGGAAAEDGRL	exon 13
4	94	94	1	R	
5	95	150	56	VNDCILRVNEVDVSEVSHS..RRRPILETVVEIKLFKGPK	exon 14
6	151	151	1	G	
7	152	196	45	LGFSIAGGVGNQHIPGDNS..DGGAAQKDGRLQVGDRLLM	exon 15
8	197	244	48	VNNYSLEEVTHEEAVAILK..GKPTTIYMTDPYGPPDITH	exon 16
9	245	245	1	S	
10	246	290	45	YSPPMENHLLSGNNGTLEY..SPGRYSPIPKHMLVDDDYT	exon 17
11	291	291	1	S	
12	292	313	22	HSQHSTATRQPSMTLQRAVSLE	exon 19
13	314	314	1	G	
14	315	366	52	EPRKVVLHKGSTGLGFNIV..AGGPADLSGELQRGDQILS	exon 20
15	367	400	34	VNGIDLRGASHEQAAAALKGAGQTVTIIAQYQPE	exon 21
16	401	401	1	D	
17	402	415	14	YARFEAKIHDLREQ	exon 23a
18	416	438	23	MMNHSMSSGSGSLRTNQKRSLYV	exon 23b
19	439	439	1	R	
20	440	497	58	AMFDYDKSKDSGLPSQGLS..RRVMLEGDSEEMGVIPSKR	exon 24
21	498	498	1	R	
22	499	523	25	VERKERARLKTVKFNAKPGVIDSKG	exon 25
23	524	538	15	DIPGLGDDGYGTKTL	exon 27

24	539	539	1	R	
25	540	555	16	GQEDLILSYEPVTRQE	exon 29
26	556	556	1	I	
27	557	589	33	NYTRPVIILGPMKDRINDDLISEFPDKFGSCVP	exon 30
28	590	590	1	H	
29	591	647	57	TTRPKRDYEVDGRDYHFVI..YNDNLYGTSVQSVRFVAER	exon 31
30	648	683	36	GKHCILDVSGNAIKRLQVAQLYPIAIFIKPRSLEPL	exon 32
31	684	684	1	M	
32	685	714	30	EMNKRLTEEQAKKTYDRAIKLEQEFGEYFT	exon 33
33	715	715	1	A	
34	716	749	34	IVQGDTLEDIYNQCKLVIEEQSGPFIWIPSKEKL	exon 34

Q15700-4

>sp|Q15700-4|DLG2_HUMAN Isoform 4 of Disks large homolog 2 OS=Homo sapiens GN=DLG2
MNAYLTKQHSCSRGSDGMDAVRSAPTLIRDAHCACGWQRNCQGLGYSSQTMPSSGPGGPA
SNRTGGSSFNRTLWDSVRKSPHKTSTKGKGTCGEHCTCPHGWFSPAQASPAPIIVNTDTL
DTIPYVNGTEIEYEFEEITLERGNSGLGFSIAGGTDNPHIGDDPGIFITKIIPGGAAAED
GRLRVNDCILRVNEVDVSEVSHSKAVEALKEAGSIVRLYVRRRRPILETVVEIKLFKGPK
GLGFSIAGGVGNQHIPGDNSIYVTKIIDGGAAQKDGRLQVGDRLLMVNNYSLEEVTHEEA
VAILKNTSEVVYLKVGKPTTIYMTDPYGPPDITHSYSPPMENHLLSGNNGTLEYKTSLPP
ISPGRYSPIPKHMLVDDDYTRPPEPVYSTVNKLCDKPASPRHYSPVECDKSFLLSAPYSH
YHLGLLPDSEMTSHSQHSTATRQPSMTLQRAVSLEGEPRKVVLHKGSTGLGFNIVGGEDG
EGIFVSFILAGGPADLSGELQRGDQILSVNGIDLRGASHEQAAAALKGAGQTVTIIAQYQ
PEDYARFEAKIHDLREQMMNHSMSSGSGSLRTNQKRSLYVRAMFDYDKSKDSGLPSQGLS
FKYGDILHVINASDDEWWQARRVMLEGDSEEMGVIPSKRRVERKERARLKTVKFNAKPGV
IDSKGSFNDKRKKSFIFSRKFPFYKNKEQSEQETSDPERGQEDLILSYEPVTRQEINYTR
PVIILGPMKDRINDDLISEFPDKFGSCVPHTTRPKRDYEVDGRDYHFVISREQMEKDIQE
HKFIEAGQYNDNLYGTSVQSVRFVAERGKHCILDVSGNAIKRLQVAQLYPIAIFIKPRSL
EPLMEMNKRLTEEQAKKTYDRAIKLEQEFGEYFTAIVQGDTLEDIYNQCKLVIEEQSGPF
IWIPSKEKL

	start	end	width	seq	name
1	1	107	107	MNAYLTKQHSCSRGSDGMD..KGTCGEHCTCPHGWFSPAQ	exon 9
2	108	125	18	ASPAPIIVNTDTLDTIPY	exon 11
3	126	142	17	VNGTEIEYEFEEITLER	exon 12
4	143	183	41	GNSGLGFSIAGGTDNPHIG..GIFITKIIPGGAAAEDGRL	exon 13
5	184	184	1	R	
6	185	240	56	VNDCILRVNEVDVSEVSHS..RRRPILETVVEIKLFKGPK	exon 14
7	241	241	1	G	
8	242	286	45	LGFSIAGGVGNQHIPGDNS..DGGAAQKDGRLQVGDRLLM	exon 15
9	287	334	48	VNNYSLEEVTHEEAVAILK..GKPTTIYMTDPYGPPDITH	exon 16
10	335	335	1	S	
11	336	380	45	YSPPMENHLLSGNNGTLEY..SPGRYSPIPKHMLVDDDYT	exon 17
12	381	381	1	R	
13	382	432	51	PPEPVYSTVNKLCDKPASP..LSAPYSHYHLGLLPDSEMT	exon 18
14	433	433	1	S	
15	434	455	22	HSQHSTATRQPSMTLQRAVSLE	exon 19
16	456	456	1	G	
17	457	508	52	EPRKVVLHKGSTGLGFNIV..AGGPADLSGELQRGDQILS	exon 20
18	509	542	34	VNGIDLRGASHEQAAAALKGAGQTVTIIAQYQPE	exon 21
19	543	543	1	D	
20	544	557	14	YARFEAKIHDLREQ	exon 23a
21	558	580	23	MMNHSMSSGSGSLRTNQKRSLYV	exon 23b
22	581	581	1	R	
23	582	639	58	AMFDYDKSKDSGLPSQGLS..RRVMLEGDSEEMGVIPSKR	exon 24
24	640	640	1	R	

25	641	665	25	VERKERARLKTVKFNAKPGVIDSKG	exon 25
26	666	698	33	SFNDKRKKSFIFSRKFPFYKNKEQSEQETSDPE	exon 26
27	699	699	1	R	
28	700	715	16	GQEDLILSYEPVTRQE	exon 29
29	716	716	1	I	
30	717	749	33	NYTRPVIILGPMKDRINDDLISEFPDKFGSCVP	exon 30
31	750	750	1	H	
32	751	807	57	TTRPKRDYEVDGRDYHFVI..YNDNLYGTSVQSVRFVAER	exon 31
33	808	843	36	GKHCILDVSGNAIKRLQVAQLYPIAIFIKPRSLEPL	exon 32
34	844	844	1	M	
35	845	874	30	EMNKRLTEEQAKKTYDRAIKLEQEFGEYFT	exon 33
36	875	875	1	A	
37	876	909	34	IVQGDTLEDIYNQCKLVIEEQSGPFIWIPSKEKL	exon 34

Q15700-5

```
>sp|Q15700-5|DLG2_HUMAN Isoform 5 of Disks large homolog 2 OS=Homo sapiens GN=DLG2
MMNHSMSSGSGSLRTNQKRSLYVRAMFDYDKSKDSGLPSQGLSFKYGDILHVINASDDEW
WQARRVMLEGDSEEMGVIPSKRRVERKERARLKTVKFNAKPGVIDSKGDIPGLGDDGYGT
KTLRGQEDLILSYEPVTRQEINYTRPVIILGPMKDRINDDLISEFPDKFGSCVPHTTRPK
RDYEVDGRDYHFVISREQMEKDIQEHKFIEAGQYNDNLYGTSVQSVRFVAERGKHCILDV
SGNAIKRLQVAQLYPIAIFIKPRSLEPLMEMNKRLTEEQAKKTYDRAIKLEQEFGEYFTA
IVQGDTLEDIYNQCKLVIEEQSGPFIWIPSKEKL
```

	start	end	width	seq	name
1	1	23	23	MMNHSMSSGSGSLRTNQKRSLYV	exon 23b
2	24	24	1	R	
3	25	82	58	AMFDYDKSKDSGLPSQGLS..RRVMLEGDSEEMGVIPSKR	exon 24
4	83	83	1	R	
5	84	108	25	VERKERARLKTVKFNAKPGVIDSKG	exon 25
6	109	123	15	DIPGLGDDGYGTKTL	exon 27
7	124	124	1	R	
8	125	140	16	GQEDLILSYEPVTRQE	exon 29
9	141	141	1	I	
10	142	174	33	NYTRPVIILGPMKDRINDDLISEFPDKFGSCVP	exon 30
11	175	175	1	H	
12	176	232	57	TTRPKRDYEVDGRDYHFVI..YNDNLYGTSVQSVRFVAER	exon 31
13	233	268	36	GKHCILDVSGNAIKRLQVAQLYPIAIFIKPRSLEPL	exon 32
14	269	269	1	M	
15	270	299	30	EMNKRLTEEQAKKTYDRAIKLEQEFGEYFT	exon 33
16	300	300	1	A	
17	301	334	34	IVQGDTLEDIYNQCKLVIEEQSGPFIWIPSKEKL	exon 34

UniProt *Dlg2* isoforms inspection

Q91XM9-1

```
>sp|Q91XM9|DLG2_MOUSE Disks large homolog 2 OS=Mus musculus GN=Dlg2 PE=1 SV=2
MFFACYCALRTNVKKYRYQDEDGPHDHSLPRLTHEVRGPELVHVSEKNLSQIENVHGYVL
QSHISPLKASPAPIIVNTDTLDTIPYVNGTEIEYEFEEITLERGNSGLGFSIAGGTDNPH
IGDDPGIFITKIIPGGAAAEDGRLRVNDCILRVNEVDVSEVSHSKAVEALKEAGSIVRLY
VRRRRPILETVVEIKLFKGPKGLGFSIAGGVGNQHIPGDNSIYVTKIIDGGAAQKDGRLQ
VGDRLLMVNNYSLEEVTHEEAVAILKNTSDVVYLKVGKPTTIYMTDPYGPPDITHSYSPP
MENHLLSGNNGTLEYKTSLPPISPGRYSPIPKHMLGEDDYTRPPEPVYSTVNKLCDKPAS
PRHYSPVECDKSFLLSTPYPHYHLGLLPDSDMTSHSQHSTATRQPSVTLQRAISLEGEPR
KVVLHKGSTGLGFNIVGGEDGEGIFVSFILAGGPADLSGELQRGDQILSVNGIDLRGASH
EQAAAALKGAGQTVTIIAQYQPEDYARFEAKIHDLREQMMNHSMSSGSGSLRTNQKRSLY
VRAMFDYDKSKDSGLPSQGLSFKYGDILHVINASDDEWWQARRVTLDGDSEEMGVIPSKR
RVERKERARLKTVKFNAKPGVIDSKGDIPGLGDDGYGTKTLRGQEDLILSYEPVTRQEIN
YTRPVIILGPMKDRINDDLISEFPDKFGSCVPHTTRPKRDYEVDGRDYHFVISREQMEKD
IQEHKFIEAGQYNDNLYGTSVQSVRFVAERGKHCILDVSGNAIKRLQVAQLYPIAIFIKP
KSLEPLMEMNKRLTEEQAKKTYDRAIKLEQEFGEYFTAIVQGDTLEDIYNQCKLVIEEQS
GPFIWIPSKEKL
```

	start	end	width	seq	name
1	1	14	14	MFFACYCALRTNVK	exon 1
2	15	68	54	KYRYQDEDGPHDHSLPRLT..SQIENVHGYVLQSHISPLK	exon 2
3	69	86	18	ASPAPIIVNTDTLDTIPY	exon 4
4	87	103	17	VNGTEIEYEFEEITLER	exon 5
5	104	144	41	GNSGLGFSIAGGTDNPHIG..GIFITKIIPGGAAAEDGRL	exon 6
6	145	145	1	R	
7	146	201	56	VNDCILRVNEVDVSEVSHS..RRRPILETVVEIKLFKGPK	exon 7
8	202	202	1	G	
9	203	247	45	LGFSIAGGVGNQHIPGDNS..DGGAAQKDGRLQVGDRLLM	exon 8
10	248	295	48	VNNYSLEEVTHEEAVAILK..GKPTTIYMTDPYGPPDITH	exon 9
11	296	296	1	S	
12	297	341	45	YSPPMENHLLSGNNGTLEY..SPGRYSPIPKHMLGEDDYT	exon 10
13	342	342	1	R	
14	343	393	51	PPEPVYSTVNKLCDKPASP..LSTPYPHYHLGLLPDSDMT	exon 11
15	394	394	1	S	
16	395	416	22	HSQHSTATRQPSVTLQRAISLE	exon 12
17	417	417	1	G	
18	418	469	52	EPRKVVLHKGSTGLGFNIV..AGGPADLSGELQRGDQILS	exon 15
19	470	503	34	VNGIDLRGASHEQAAAALKGAGQTVTIIAQYQPE	exon 16
20	504	504	1	D	
21	505	518	14	YARFEAKIHDLREQ	exon 18a
22	519	541	23	MMNHSMSSGSGSLRTNQKRSLYV	exon 18b
23	542	542	1	R	
24	543	600	58	AMFDYDKSKDSGLPSQGLS..RRVTLDGDSEEMGVIPSKR	exon 19
25	601	601	1	R	
26	602	626	25	VERKERARLKTVKFNAKPGVIDSKG	exon 20
27	627	641	15	DIPGLGDDGYGTKTL	exon 22
28	642	642	1	R	
29	643	658	16	GQEDLILSYEPVTRQE	exon 24
30	659	659	1	I	
31	660	692	33	NYTRPVIILGPMKDRINDDLISEFPDKFGSCVP	exon 25
32	693	693	1	H	
33	694	750	57	TTRPKRDYEVDGRDYHFVI..YNDNLYGTSVQSVRFVAER	exon 26
34	751	786	36	GKHCILDVSGNAIKRLQVAQLYPIAIFIKPKSLEPL	exon 27
35	787	787	1	M	
36	788	817	30	EMNKRLTEEQAKKTYDRAIKLEQEFGEYFT	exon 28

37	818	818	1	A	
38	819	852	34	IVQGDTLEDIYNQCKLVIEEQSGPFIWIPSKEKL	exon 29

Q91XM9-2

>sp|Q91XM9-2|DLG2_MOUSE Isoform 2 of Disks large homolog 2 OS=Mus musculus GN=Dlg2
MICHCKVACTNNTLSLMFGCKKYRYQDEDGPHDHSLPRLTHEVRGPELVHVSEKNLSQIE
NVHGYVLQSHISPLKASPAPIIVNTDTLDTIPYVNGTEIEYEFEEITLERGNSGLGFSIA
GGTDNPHIGDDPGIFITKIIPGGAAAEDGRLRVNDCILRVNEVDVSEVSHSKAVEALKEA
GSIVRLYVRRRRPILETVVEIKLFKGPKGLGFSIAGGVGNQHIPGDNSIYVTKIIDGGAA
QKDGRLQVGDRLLMVNNYSLEEVTHEEAVAILKNTSDVVYLKVGKPTTIYMTDPYGPPDI
THSYSPPMENHLLSGNNGTLEYKTSLPPISPGRYSPIPKHMLGEDDYTRPPEPVYSTVNK
LCDKPASPRHYSPVECDKSFLLSTPYPHYHLGLLPDSDMTSHSQHSTATRQPSVTLQRAI
SLEGEPRKVVLHKGSTGLGFNIVGGEDGEGIFVSFILAGGPADLSGELQRGDQILSVNGI
DLRGASHEQAAAALKGAGQTVTIIAQYQPEDYARFEAKIHDLREQMMNHSMSSGSGSLRT
NQKRSLYVRAMFDYDKSKDSGLPSQGLSFKYGDILHVINASDDEWWQARRVTLDGDSEEM
GVIPSKRRVERKERARLKTVKFNAKPGVIDSKGDIPGLGDDGYGTKTLRGQEDLILSYEP
VTRQEINYTRPVIILGPMKDRINDDLISEFPDKFGSCVPHTTRPKRDYEVDGRDYHFVIS
REQMEKDIQEHKFIEAGQYNDNLYGTSVQSVRFVAERGKHCILDVSGNAIKRLQVAQLYP
IAIFIKPKSLEPLMEMNKRLTEEQAKKTYDRAIKLEQEFGEYFTAIVQGDTLEDIYNQCK
LVIEEQSGPFIWIPSKEKL

	start	end	width	seq	name
1	1	21	21	MICHCKVACTNNTLSLMFGCK	mCFEin1_cds
2	22	75	54	KYRYQDEDGPHDHSLPRLT..SQIENVHGYVLQSHISPLK	exon 2
3	76	93	18	ASPAPIIVNTDTLDTIPY	exon 4
4	94	110	17	VNGTEIEYEFEEITLER	exon 5
5	111	151	41	GNSGLGFSIAGGTDNPHIG..GIFITKIIPGGAAAEDGRL	exon 6
6	152	152	1	R	
7	153	208	56	VNDCILRVNEVDVSEVSHS..RRRPILETVVEIKLFKGPK	exon 7
8	209	209	1	G	
9	210	254	45	LGFSIAGGVGNQHIPGDNS..DGGAAQKDGRLQVGDRLLM	exon 8
10	255	302	48	VNNYSLEEVTHEEAVAILK..GKPTTIYMTDPYGPPDITH	exon 9
11	303	303	1	S	
12	304	348	45	YSPPMENHLLSGNNGTLEY..SPGRYSPIPKHMLGEDDYT	exon 10
13	349	349	1	R	
14	350	400	51	PPEPVYSTVNKLCDKPASP..LSTPYPHYHLGLLPDSDMT	exon 11
15	401	401	1	S	
16	402	423	22	HSQHSTATRQPSVTLQRAISLE	exon 12
17	424	424	1	G	
18	425	476	52	EPRKVVLHKGSTGLGFNIV..AGGPADLSGELQRGDQILS	exon 15
19	477	510	34	VNGIDLRGASHEQAAAALKGAGQTVTIIAQYQPE	exon 16
20	511	511	1	D	
21	512	525	14	YARFEAKIHDLREQ	exon 18a
22	526	548	23	MMNHSMSSGSGSLRTNQKRSLYV	exon 18b
23	549	549	1	R	
24	550	607	58	AMFDYDKSKDSGLPSQGLS..RRVTLDGDSEEMGVIPSKR	exon 19
25	608	608	1	R	
26	609	633	25	VERKERARLKTVKFNAKPGVIDSKG	exon 20
27	634	648	15	DIPGLGDDGYGTKTL	exon 22
28	649	649	1	R	
29	650	665	16	GQEDLILSYEPVTRQE	exon 24
30	666	666	1	I	
31	667	699	33	NYTRPVIILGPMKDRINDDLISEFPDKFGSCVP	exon 25
32	700	700	1	H	
33	701	757	57	TTRPKRDYEVDGRDYHFVI..YNDNLYGTSVQSVRFVAER	exon 26
34	758	793	36	GKHCILDVSGNAIKRLQVAQLYPIAIFIKPKSLEPL	exon 27

35	794	794	1	M	
36	795	824	30	EMNKRLTEEQAKKTYDRAIKLEQEFGEYFT	exon 28
37	825	825	1	A	
38	826	859	34	IVQGDTLEDIYNQCKLVIEEQSGPFIWIPSKEKL	exon 29

Q91XM9-3

>sp|Q91XM9-3|DLG2_MOUSE Isoform 3 of Disks large homolog 2 OS=Mus musculus GN=Dlg2
MQHAFIPASPAPIIVNTDTLDTIPYVNGTEIEYEFEEITLERGNSGLGFSIAGGTDNPHI
GDDPGIFITKIIPGGAAAEDGRLRVNDCILRVNEVDVSEVSHSKAVEALKEAGSIVRLYV
RRRRPILETVVEIKLFKGPKGLGFSIAGGVGNQHIPGDNSIYVTKIIDGGAAQKDGRLQV
GDRLLMVNNYSLEEVTHEEAVAILKNTSDVVYLKVGKPTTIYMTDPYGPPDITHSYSPPM
ENHLLSGNNGTLEYKTSLPPISPGRYSPIPKHMLGEDDYTRPPEPVYSTVNKLCDKPASP
RHYSPVECDKSFLLSTPYPHYHLGLLPDSDMTSHSQHSTATRQPSVTLQRAISLEGEPRK
VVLHKGSTGLGFNIVGGEDGEGIFVSFILAGGPADLSGELQRGDQILSVNGIDLRGASHE
QAAAALKGAGQTVTIIAQYQPEDYARFEAKIHDLREQMMNHSMSSGSGSLRTNQKRSLYV
RAMFDYDKSKDSGLPSQGLSFKYGDILHVINASDDEWWQARRVTLDGDSEEMGVIPSKRR
VERKERARLKTVKFNAKPGVIDSKGDIPGLGDDGYGTKTLRGQEDLILSYEPVTRQEINY
TRPVIILGPMKDRINDDLISEFPDKFGSCVPHTTRPKRDYEVDGRDYHFVISREQMEKDI
QEHKFIEAGQYNDNLYGTSVQSVRFVAERGKHCILDVSGNAIKRLQVAQLYPIAIFIKPK
SLEPLMEMNKRLTEEQAKKTYDRAIKLEQEFGEYFTAIVQGDTLEDIYNQCKLVIEEQSG
PFIWIPSKEKL

	start	end	width	seq	name
1	1	7	7	MQHAFIP	
2	8	25	18	ASPAPIIVNTDTLDTIPY	exon 4
3	26	42	17	VNGTEIEYEFEEITLER	exon 5
4	43	83	41	GNSGLGFSIAGGTDNPHIG..GIFITKIIPGGAAAEDGRL	exon 6
5	84	84	1	R	
6	85	140	56	VNDCILRVNEVDVSEVSHS..RRRPILETVVEIKLFKGPK	exon 7
7	141	141	1	G	
8	142	186	45	LGFSIAGGVGNQHIPGDNS..DGGAAQKDGRLQVGDRLLM	exon 8
9	187	234	48	VNNYSLEEVTHEEAVAILK..GKPTTIYMTDPYGPPDITH	exon 9
10	235	235	1	S	
11	236	280	45	YSPPMENHLLSGNNGTLEY..SPGRYSPIPKHMLGEDDYT	exon 10
12	281	281	1	R	
13	282	332	51	PPEPVYSTVNKLCDKPASP..LSTPYPHYHLGLLPDSDMT	exon 11
14	333	333	1	S	
15	334	355	22	HSQHSTATRQPSVTLQRAISLE	exon 12
16	356	356	1	G	
17	357	408	52	EPRKVVLHKGSTGLGFNIV..AGGPADLSGELQRGDQILS	exon 15
18	409	442	34	VNGIDLRGASHEQAAAALKGAGQTVTIIAQYQPE	exon 16
19	443	443	1	D	
20	444	457	14	YARFEAKIHDLREQ	exon 18a
21	458	480	23	MMNHSMSSGSGSLRTNQKRSLYV	exon 18b
22	481	481	1	R	
23	482	539	58	AMFDYDKSKDSGLPSQGLS..RRVTLDGDSEEMGVIPSKR	exon 19
24	540	540	1	R	
25	541	565	25	VERKERARLKTVKFNAKPGVIDSKG	exon 20
26	566	580	15	DIPGLGDDGYGTKTL	exon 22
27	581	581	1	R	
28	582	597	16	GQEDLILSYEPVTRQE	exon 24
29	598	598	1	I	
30	599	631	33	NYTRPVIILGPMKDRINDDLISEFPDKFGSCVP	exon 25
31	632	632	1	H	
32	633	689	57	TTRPKRDYEVDGRDYHFVI..YNDNLYGTSVQSVRFVAER	exon 26
33	690	725	36	GKHCILDVSGNAIKRLQVAQLYPIAIFIKPKSLEPL	exon 27

34	726	726	1	M	
35	727	756	30	EMNKRLTEEQAKKTYDRAIKLEQEFGEYFT	exon 28
36	757	757	1	A	
37	758	791	34	IVQGDTLEDIYNQCKLVIEEQSGPFIWIPSKEKL	exon 29

MQHAFIP sequence matches with the last seven amino-acids of *DLG2* exon 10, suggesting the presence of an unknown mouse coding exon between exons 3 and 4. This hypothesis is backed up the the alignment of the RT-PCR primer, used to identify such isoform in mouse, in chr7:91711767-91711790, that locates between exons 3 and 4. See Supplementary Note 6 for details and [12].

Q91XM9-4

```
>sp|Q91XM9-4|DLG2_MOUSE Isoform 4 of Disks large homolog 2 OS=Mus musculus GN=Dlg2
MNAYLTKQHSCSRGSDGMDIGRSAPTLIRDAHCACGWQRNAQGLGYSSQTMPSSGPGGPA
SNRTKLVTLWDSVRKSPHKTSTKGKGNCGERCACPHGWFSPAQASPAPIIVNTDTLDTIP
YVNGTEIEYEFEEITLERGNSGLGFSIAGGTDNPHIGDDPGIFITKIIPGGAAAEDGRLR
VNDCILRVNEVDVSEVSHSKAVEALKEAGSIVRLYVRRRRPILETVVEIKLFKGPKGLGF
SIAGGVGNQHIPGDNSIYVTKIIDGGAAQKDGRLQVGDRLLMVNNYSLEEVTHEEAVAIL
KNTSDVVYLKVGKPTTIYMTDPYGPPDITHSYSPPMENHLLSGNNGTLEYKTSLPPISPG
RYSPIPKHMLGEDDYTRPPEPVYSTVNKLCDKPASPRHYSPVECDKSFLLSTPYPHYHLG
LLPDSDMTSHSQHSTATRQPSVTLQRAISLEGEPRKVVLHKGSTGLGFNIVGGEDGEGIF
VSFILAGGPADLSGELQRGDQILSVNGIDLRGASHEQAAAALKGAGQTVTIIAQYQPEDY
ARFEAKIHDLREQMMNHSMSSGSGSLRTNQKRSLYVRAMFDYDKSKDSGLPSQGLSFKYG
DILHVINASDDEWWQARRVTLDGDSEEMGVIPSKRRVERKERARLKTVKFNAKPGVIDSK
GDIPGLGDDGYGTKTLRGQEDLILSYEPVTRQEINYTRPVIILGPMKDRINDDLISEFPD
KFGSCVPHTTRPKRDYEVDGRDYHFVISREQMEKDIQEHKFIEAGQYNDNLYGTSVQSVR
FVAERGKHCILDVSGNAIKRLQVAQLYPIAIFIKPKSLEPLMEMNKRLTEEQAKKTYDRA
IKLEQEFGEYFTAIVQGDTLEDIYNQCKLVIEEQSGPFIWIPSKEKL
```

	start	end	width	seq	name
1	1	103	103	MNAYLTKQHSCSRGSDGMD..KGNCGERCACPHGWFSPAQ	exon 3
2	104	121	18	ASPAPIIVNTDTLDTIPY	exon 4
3	122	138	17	VNGTEIEYEFEEITLER	exon 5
4	139	179	41	GNSGLGFSIAGGTDNPHIG..GIFITKIIPGGAAAEDGRL	exon 6
5	180	180	1	R	
6	181	236	56	VNDCILRVNEVDVSEVSHS..RRRPILETVVEIKLFKGPK	exon 7
7	237	237	1	G	
8	238	282	45	LGFSIAGGVGNQHIPGDNS..DGGAAQKDGRLQVGDRLLM	exon 8
9	283	330	48	VNNYSLEEVTHEEAVAILK..GKPTTIYMTDPYGPPDITH	exon 9
10	331	331	1	S	
11	332	376	45	YSPPMENHLLSGNNGTLEY..SPGRYSPIPKHMLGEDDYT	exon 10
12	377	377	1	R	
13	378	428	51	PPEPVYSTVNKLCDKPASP..LSTPYPHYHLGLLPDSDMT	exon 11
14	429	429	1	S	
15	430	451	22	HSQHSTATRQPSVTLQRAISLE	exon 12
16	452	452	1	G	
17	453	504	52	EPRKVVLHKGSTGLGFNIV..AGGPADLSGELQRGDQILS	exon 15
18	505	538	34	VNGIDLRGASHEQAAAALKGAGQTVTIIAQYQPE	exon 16
19	539	539	1	D	
20	540	553	14	YARFEAKIHDLREQ	exon 18a
21	554	576	23	MMNHSMSSGSGSLRTNQKRSLYV	exon 18b
22	577	577	1	R	
23	578	635	58	AMFDYDKSKDSGLPSQGLS..RRVTLDGDSEEMGVIPSKR	exon 19
24	636	636	1	R	
25	637	661	25	VERKERARLKTVKFNAKPGVIDSKG	exon 20
26	662	676	15	DIPGLGDDGYGTKTL	exon 22
27	677	677	1	R	
28	678	693	16	GQEDLILSYEPVTRQE	exon 24

	start	end	width	seq	name
29	694	694	1	I	
30	695	727	33	NYTRPVIILGPMKDRINDDLISEFPDKFGSCVP	exon 25
31	728	728	1	H	
32	729	785	57	TTRPKRDYEVDGRDYHFVI..YNDNLYGTSVQSVRFVAER	exon 26
33	786	821	36	GKHCILDVSGNAIKRLQVAQLYPIAIFIKPKSLEPL	exon 27
34	822	822	1	M	
35	823	852	30	EMNKRLTEEQAKKTYDRAIKLEQEFGEYFT	exon 28
36	853	853	1	A	
37	854	887	34	IVQGDTLEDIYNQCKLVIEEQSGPFIWIPSKEKL	exon 29

Q91XM9-5

```
>sp|Q91XM9-5|DLG2_MOUSE Isoform 5 of Disks large homolog 2 OS=Mus musculus GN=Dlg2
MNAYLTKQHSCSRGSDGMDIGRSAPTLIRDAHCACGWQRNAQGLGYSSQTMPSSGPGGPA
SNRTKLVTLWDSVRKSPHKTSTKGKGNCGERCACPHGWFSPAQASPAPIIVNTDTLDTIP
YVNGTEIEYEFEEITLERGNSGLGFSIAGGTDNPHIGDDPGIFITKIIPGGAAAEDGRLR
VNDCILRVNEVDVSEVSHSKAVEALKEAGSIVRLYVRRRRPILETVVEIKLFKGPKGLGF
SIAGGVGNQHIPGDNSIYVTKIIDGGAAQKDGRLQVGDRLLMVNNYSLEEVTHEEAVAIL
KNTSDVVYLKVGKPTTIYMTDPYGPPDITHSYSPPMENHLLSGNNGTLEYKTSLPPISPG
RYSPIPKHMLGEDDYTRPPEPVYSTVNKLCDKPASPRHYSPVECDKSFLLSTPYPHYHLG
LLPDSDMTRYCMRFLTSSSPVACVSTRMDGWNSSPPTSLALSTFLVERCSASMVRWEKLR
TWLFCSFCCAH
```

	start	end	width	seq	name
1	1	103	103	MNAYLTKQHSCSRGSDGMD..KGNCGERCACPHGWFSPAQ	exon 3
2	104	121	18	ASPAPIIVNTDTLDTIPY	exon 4
3	122	138	17	VNGTEIEYEFEEITLER	exon 5
4	139	179	41	GNSGLGFSIAGGTDNPHIG..GIFITKIIPGGAAAEDGRL	exon 6
5	180	180	1	R	
6	181	236	56	VNDCILRVNEVDVSEVSHS..RRRPILETVVEIKLFKGPK	exon 7
7	237	237	1	G	
8	238	282	45	LGFSIAGGVGNQHIPGDNS..DGGAAQKDGRLQVGDRLLM	exon 8
9	283	330	48	VNNYSLEEVTHEEAVAILK..GKPTTIYMTDPYGPPDITH	exon 9
10	331	331	1	S	
11	332	376	45	YSPPMENHLLSGNNGTLEY..SPGRYSPIPKHMLGEDDYT	exon 10
12	377	377	1	R	
13	378	428	51	PPEPVYSTVNKLCDKPASP..LSTPYPHYHLGLLPDSDMT	exon 11
14	429	491	63	RYCMRFLTSSSPVACVSTR..MVRWEKLRTWLFCSFCCAH	exon 11b

Q91XM9-6

```
>sp|Q91XM9-6|DLG2_MOUSE Isoform 6 of Disks large homolog 2 OS=Mus musculus GN=Dlg2
MFASIWYAKKLGRRFVHNARKAKSEKASPAPIIVNTDTLDTIPYVNGTEIEYEFEEITLE
RGNSGLGFSIAGGTDNPHIGDDPGIFITKIIPGGAAAEDGRLRVNDCILRVNEVDVSEVS
HSKAVEALKEAGSIVRLYVRRRRPILETVVEIKLFKGPKGLGFSIAGGVGNQHIPGDNSI
YVTKIIDGGAAQKDGRLQVGDRLLMVNNYSLEEVTHEEAVAILKNTSDVVYLKVGKPTTI
YMTDPYGPPDITHSYSPPMENHLLSGNNGTLEYKTSLPPISPGRYSPIPKHMLGEDDYTR
PPEPVYSTVNKLCDKPASPRHYSPVECDKSFLLSTPYPHYHLGLLPDSDMTSHSQHSTAT
RQPSVTLQRAISLEGEPRKVVLHKGSTGLGFNIVGGEDGEGIFVSFILAGGPADLSGELQ
RGDQILSVNGIDLRGASHEQAAAALKGAGQTVTIIAQYQPEDYARFEAKIHDLREQMMNH
SMSSGSGSLRTNQKRSLYVRAMFDYDKSKDSGLPSQGLSFKYGDILHVINASDDEWWQAR
RVTLDGDSEEMGVIPSKRRVERKERARLKTVKFNAKPGVIDSKGDIPGLGDDGYGTKTLR
GQEDLILSYEPVTRQEINYTRPVIILGPMKDRINDDLISEFPDKFGSCVPHTTRPKRDYE
VDGRDYHFVISREQMEKDIQEHKFIEAGQYNDNLYGTSVQSVRFVAERGKHCILDVSGNA
IKRLQVAQLYPIAIFIKPKSLEPLMEMNKRLTEEQAKKTYDRAIKLEQEFGEYFTAIVQG
DTLEDIYNQCKLVIEEQSGPFIWIPSKEKL
```

start	end	width	seq	name

1	1	26	26	MFASIWYAKKLGRRFVHNARKAKSEK	mCFEin2_cds
2	27	44	18	ASPAPIIVNTDTLDTIPY	exon 4
3	45	61	17	VNGTEIEYEFEEITLER	exon 5
4	62	102	41	GNSGLGFSIAGGTDNPHIG..GIFITKIIPGGAAAEDGRL	exon 6
5	103	103	1	R	
6	104	159	56	VNDCILRVNEVDVSEVSHS..RRRPILETVVEIKLFKGPK	exon 7
7	160	160	1	G	
8	161	205	45	LGFSIAGGVGNQHIPGDNS..DGGAAQKDGRLQVGDRLLM	exon 8
9	206	253	48	VNNYSLEEVTHEEAVAILK..GKPTTIYMTDPYGPPDITH	exon 9
10	254	254	1	S	
11	255	299	45	YSPPMENHLLSGNNGTLEY..SPGRYSPIPKHMLGEDDYT	exon 10
12	300	300	1	R	
13	301	351	51	PPEPVYSTVNKLCDKPASP..LSTPYPHYHLGLLPDSDMT	exon 11
14	352	352	1	S	
15	353	374	22	HSQHSTATRQPSVTLQRAISLE	exon 12
16	375	375	1	G	
17	376	427	52	EPRKVVLHKGSTGLGFNIV..AGGPADLSGELQRGDQILS	exon 15
18	428	461	34	VNGIDLRGASHEQAAAALKGAGQTVTIIAQYQPE	exon 16
19	462	462	1	D	
20	463	476	14	YARFEAKIHDLREQ	exon 18a
21	477	499	23	MMNHSMSSGSGSLRTNQKRSLYV	exon 18b
22	500	500	1	R	
23	501	558	58	AMFDYDKSKDSGLPSQGLS..RRVTLDGDSEEMGVIPSKR	exon 19
24	559	559	1	R	
25	560	584	25	VERKERARLKTVKFNAKPGVIDSKG	exon 20
26	585	599	15	DIPGLGDDGYGTKTL	exon 22
27	600	600	1	R	
28	601	616	16	GQEDLILSYEPVTRQE	exon 24
29	617	617	1	I	
30	618	650	33	NYTRPVIILGPMKDRINDDLISEFPDKFGSCVP	exon 25
31	651	651	1	H	
32	652	708	57	TTRPKRDYEVDGRDYHFVI..YNDNLYGTSVQSVRFVAER	exon 26
33	709	744	36	GKHCILDVSGNAIKRLQVAQLYPIAIFIKPKSLEPL	exon 27
34	745	745	1	M	
35	746	775	30	EMNKRLTEEQAKKTYDRAIKLEQEFGEYFT	exon 28
36	776	776	1	A	
37	777	810	34	IVQGDTLEDIYNQCKLVIEEQSGPFIWIPSKEKL	exon 29

Q91XM9-7, PSD-93 zeta

```
>sp|Q91XM9-7|DLG2_MOUSE Isoform 7 of Disks large homolog 2 OS=Mus musculus GN=Dlg2
MPVKKKDTDRALSLLEEYCKKLRKPEEQLLKNAVKKVMSIFKSSLFQALLDIQEFYEVTL
LNSQKSCEQKIEEANHVAQKWEKTLLLDSCRDSLQKSSEHASCSGPKENALYIEQNKENQ
CSENETEEKTCQNQGKCPAQNCSVEAPTWMPVHHCTKYRYQDEDGPHDHSLPRLTHEVRG
PELVHVSEKNLSQIENVHGYVLQSHISPLKVNGTEIEYEFEEITLERGNSGLGFSIAGGT
DNPHIGDDPGIFITKIIPGGAAAEDGRLRVNDCILRVNEVDVSEVSHSKAVEALKEAGSI
VRLYVRRRRPILETVVEIKLFKGPKGLGFSIAGGVGNQHIPGDNSIYVTKIIDGGAAQKD
GRLQVGDRLLMVNNYSLEEVTHEEAVAILKNTSDVVYLKVGKPTTIYMTDPYGPPDITHS
YSPPMENHLLSGNNGTLEYKTSLPPISPGRYSPIPKHMLGEDDYTRPPEPVYSTVNKLCD
KPASPRHYSPVECDKSFLLSTPYPHYHLGLLPDSDMTSHSQHSTATRQPSVTLQRAISLE
GEPRKVVLHKGSTGLGFNIVGGEDGEGIFVSFILAGGPADLSGELQRGDQILSVNGIDLR
GASHEQAAAALKGAGQTVTIIAQYQPEDYARFEAKIHDLREQMMNHSMSSGSGSLRTNQK
RSLYVRAMFDYDKSKDSGLPSQGLSFKYGDILHVINASDDEWWQARRVTLDGDSEEMGVI
PSKRRVERKERARLKTVKFNAKPGVIDSKGDIPGLGDDGYGTKTLRGQEDLILSYEPVTR
QEINYTRPVIILGPMKDRINDDLISEFPDKFGSCVPHTTRPKRDYEVDGRDYHFVISREQ
MEKDIQEHKFIEAGQYNDNLYGTSVQSVRFVAERGKHCILDVSGNAIKRLQVAQLYPIAI
FIKPKSLEPLMEMNKRLTEEQAKKTYDRAIKLEQEFGEYFTAIVQGDTLEDIYNQCKLVI
```

EEQSGPFIWIPSKEKL

	start	end	width	seq	name
1	1	156	156	MPVKKKDTDRALSLLEEYC..PAQNCSVEAPTWMPVHHCT	
2	157	210	54	KYRYQDEDGPHDHSLPRLT..SQIENVHGYVLQSHISPLK	exon 2
3	211	227	17	VNGTEIEYEFEEITLER	exon 5
4	228	268	41	GNSGLGFSIAGGTDNPHIG..GIFITKIIPGGAAAEDGRL	exon 6
5	269	269	1	R	
6	270	325	56	VNDCILRVNEVDVSEVSHS..RRRPILETVVEIKLFKGPK	exon 7
7	326	326	1	G	
8	327	371	45	LGFSIAGGVGNQHIPGDNS..DGGAAQKDGRLQVGDRLLM	exon 8
9	372	419	48	VNNYSLEEVTHEEAVAILK..GKPTTIYMTDPYGPPDITH	exon 9
10	420	420	1	S	
11	421	465	45	YSPPMENHLLSGNNGTLEY..SPGRYSPIPKHMLGEDDYT	exon 10
12	466	466	1	R	
13	467	517	51	PPEPVYSTVNKLCDKPASP..LSTPYPHYHLGLLPDSDMT	exon 11
14	518	518	1	S	
15	519	540	22	HSQHSTATRQPSVTLQRAISLE	exon 12
16	541	541	1	G	
17	542	593	52	EPRKVVLHKGSTGLGFNIV..AGGPADLSGELQRGDQILS	exon 15
18	594	627	34	VNGIDLRGASHEQAAAALKGAGQTVTIIAQYQPE	exon 16
19	628	628	1	D	
20	629	642	14	YARFEAKIHDLREQ	exon 18a
21	643	665	23	MMNHSMSSGSGSLRTNQKRSLYV	exon 18b
22	666	666	1	R	
23	667	724	58	AMFDYDKSKDSGLPSQGLS..RRVTLDGDSEEMGVIPSKR	exon 19
24	725	725	1	R	
25	726	750	25	VERKERARLKTVKFNAKPGVIDSKG	exon 20
26	751	765	15	DIPGLGDDGYGTKTL	exon 22
27	766	766	1	R	
28	767	782	16	GQEDLILSYEPVTRQE	exon 24
29	783	783	1	I	
30	784	816	33	NYTRPVIILGPMKDRINDDLISEFPDKFGSCVP	exon 25
31	817	817	1	H	
32	818	874	57	TTRPKRDYEVDGRDYHFVI..YNDNLYGTSVQSVRFVAER	exon 26
33	875	910	36	GKHCILDVSGNAIKRLQVAQLYPIAIFIKPKSLEPL	exon 27
34	911	911	1	M	
35	912	941	30	EMNKRLTEEQAKKTYDRAIKLEQEFGEYFT	exon 28
36	942	942	1	A	
37	943	976	34	IVQGDTLEDIYNQCKLVIEEQSGPFIWIPSKEKL	exon 29

Using UniProt BLAST on UniProtKB/Swiss-Prot, MPVKKKDTDRALSLLEEYC..PAQNCSVEAPTWMPVHHCT aligns to the beginning of *DLG2* Q15700-2 isoform with E-value of $1.1 \cdot 10^{-58}$, Score: 510 and Ident.: 82.4%. The beginning of the human isoform corresponds to exons 3-6 of *DLG2*.

```
>
MPVKKKDTDRALSLLEEYCKKLRKPEEQLLKNAVKKVMS
IFKSSLFQALLDIQEFYEVTLLNSQKSCEQKIEEANHVA
QKWEKTLLLDSCRDSLQKSSEHASCSGPKENALYIEQNK
ENQCSENETEEKTCQNQGKCPAQNCSVEAPTWMPVHHCT

Query       38   MSIFKSSLFQALLDIQEFYEVTLLNSQKSCEQKIEEANHVAQKWEKTLLLDSCRDSLQKS         97
                 M IFKSSLFQALLDIQEFYEVTLLNSQKSCEQKIEEAN V QKWEKT LL  C D LQKS
Q15700-2    1    MGIFKSSLFQALLDIQEFYEVTLLNSQKSCEQKIEEANQVLQKWEKTSLLAPCHDRLQKS         60

Query       98   SEHASCSGPKENALYIEQNKENQCSENETEEKTCQNQGKCPAQNCSVEAPTWMPVHHCT         156
                 SE   CSG KENA  IEQNKENQ  ENET+E T QNQG+CPAQNCSVEAP WMPVHHCT
Q15700-2    61   SELTDCSGSKENASCIEQNKENQSFENETDETTTQNQGRCPAQNCSVEAPAWMPVHHCT         119
```

Q91XM9-8

```
>sp|Q91XM9-8|DLG2_MOUSE Isoform 8 of Disks large homolog 2 OS=Mus musculus GN=Dlg2
MFFACYCALRTNVKKYRYQDEDGPHDHSLPRLTHEVRGPELVHVSEKNLSQIENVHGYVL
QSHISPLKASPAPIIVNTDTLDTIPYVNGTEIEYEFEEITLERGNSGLGFSIAGGTDNPH
IGDDPGIFITKIIPGGAAAEDGRLRVNDCILRVNEVDVSEVSHSKAVEALKEAGSIVRLY
VRRRRPILETVVEIKLFKGPKGLGFSIAGGVGNQHIPGDNSIYVTKIIDGGAAQKDGRLQ
VGDRLLMVNNYSLEEVTHEEAVAILKNTSDVVYLKVGKPTTIYMTDPYGPPDITHSYSPP
MENHLLSGNNGTLEYKTSLPPISPGRYSPIPKHMLGEDDYTRPPEPVYSTVNKLCDKPAS
PRHYSPVECDKSFLLSTPYPHYHLGLLPDSDMTSHSQHSTATRQPSVTLQRAISLEGEPR
KVVLHKGSTGLGFNIVGGEDGEGIFVSFILAGGPADLSGELQRGVINASVNRTGDRRIWH
QGNGKAASSVSCLLPALFPNFVLDYARFEAKIHDLREQMMNHSMSSGSGSLRTNQKRSLY
VRAMFDYDKSKDSGLPSQGLSFKYGDILHVINASDDEWWQARRVTLDGDSEEMGVIPSKR
RVERKERARLKTVKFNAKPGVIDSKGDIPGLGDDGYGTKTLRGQEDLILSYEPVTRQEIN
YTRPVIILGPMKDRINDDLISEFPDKFGSCVPHTTRPKRDYEVDGRDYHFVISREQMEKD
IQEHKFIEAGQYNDNLYGTSVQSVRFVAERGKHCILDVSGNAIKRLQVAQLYPIAIFIKP
KSLEPLMEMNKRLTEEQAKKTYDRAIKLEQEFGEYFTAIVQGDTLEDIYNQCKLVIEEQS
GPFIWIPSKEKL
```

	start	end	width	seq	name
1	1	14	14	MFFACYCALRTNVK	exon 1
2	15	68	54	KYRYQDEDGPHDHSLPRLT..SQIENVHGYVLQSHISPLK	exon 2
3	69	86	18	ASPAPIIVNTDTLDTIPY	exon 4
4	87	103	17	VNGTEIEYEFEEITLER	exon 5
5	104	144	41	GNSGLGFSIAGGTDNPHIG..GIFITKIIPGGAAAEDGRL	exon 6
6	145	145	1	R	
7	146	201	56	VNDCILRVNEVDVSEVSHS..RRRPILETVVEIKLFKGPK	exon 7
8	202	202	1	G	
9	203	247	45	LGFSIAGGVGNQHIPGDNS..DGGAAQKDGRLQVGDRLLM	exon 8
10	248	295	48	VNNYSLEEVTHEEAVAILK..GKPTTIYMTDPYGPPDITH	exon 9
11	296	296	1	S	
12	297	341	45	YSPPMENHLLSGNNGTLEY..SPGRYSPIPKHMLGEDDYT	exon 10
13	342	342	1	R	
14	343	393	51	PPEPVYSTVNKLCDKPASP..LSTPYPHYHLGLLPDSDMT	exon 11
15	394	394	1	S	
16	395	416	22	HSQHSTATRQPSVTLQRAISLE	exon 12
17	417	504	88	GEPRKVVLHKGSTGLGFNI..AASSVSCLLPALFPNFVLD	
18	505	518	14	YARFEAKIHDLREQ	exon 18a
19	519	541	23	MMNHSMSSGSGSLRTNQKRSLYV	exon 18b
20	542	542	1	R	
21	543	600	58	AMFDYDKSKDSGLPSQGLS..RRVTLDGDSEEMGVIPSKR	exon 19
22	601	601	1	R	
23	602	626	25	VERKERARLKTVKFNAKPGVIDSKG	exon 20
24	627	641	15	DIPGLGDDGYGTKTL	exon 22
25	642	642	1	R	
26	643	658	16	GQEDLILSYEPVTRQE	exon 24
27	659	659	1	I	
28	660	692	33	NYTRPVIILGPMKDRINDDLISEFPDKFGSCVP	exon 25
29	693	693	1	H	
30	694	750	57	TTRPKRDYEVDGRDYHFVI..YNDNLYGTSVQSVRFVAER	exon 26
31	751	786	36	GKHCILDVSGNAIKRLQVAQLYPIAIFIKPKSLEPL	exon 27
32	787	787	1	M	
33	788	817	30	EMNKRLTEEQAKKTYDRAIKLEQEFGEYFT	exon 28
34	818	818	1	A	
35	819	852	34	IVQGDTLEDIYNQCKLVIEEQSGPFIWIPSKEKL	exon 29

Using UniProt BLAST on UniProtKB/Swiss-Prot, `GEPRKVVLHKGSTGLGFNI..AASSVSCLLPALFPNFVLD` aligns to *DLG2* Q15700-3 isoform with E-value of $7.3 \cdot 10^{-25}$, Score: 253 and Ident.: 81.5%. The beginning of the

sequence corresponds approximately to *DLG2* exon 20.

```
>
GEPRKVVLHKGSTGLGFNIVGGEDGEGIFV
SFILAGGPADLSGELQRGVINASVNRTGDR
RIWHQGNGKAASSVSCLLPALFPNFVLD
```

```
Query       1      GEPRKVVLHKGSTGLGFNIVGGEDGEGIFVSFILAGGPADLSGELQRGVINASVNRTGDR          60
                   GEPRKVVLHKGSTGLGFNIVGGEDGEGIFVSFILAGGPADLSGELQRG    SVN    R
Q15700-3    314    GEPRKVVLHKGSTGLGFNIVGGEDGEGIFVSFILAGGPADLSGELQRGDQILSVNGIDLR          373

Query       61     RIWHQ          65
                      H+
Q15700-3    374    GASHE          378
```

Supplementary Note 5

Using the exon-amino-acid mapping in Supplementary Note 3, we map the predicted *DLG2* isoform from NCBI BLAST database to exons. For this mapping, we assume this additional exon-AA mapping:

```
exon 28     e26-Q]HVSSNASDSESSY[R-e29
```

UniProt *DLG2* predicted isoforms inspection

XP_016872766

```
>XP_016872766
MFASIWYAKKLGRRFVHNARKAKSEKASPAPIIVNTDTLDTIPY
VNGTEIEYEFEEITLERGNSGLGFSIAGGTDNPHIGDDPGIFITKIIPGGAAAEDGRL
RVNDCILRVNEVDVSEVSHSKAVEALKEAGSIVRLYVRRRRPILETVVEIKLFKGPKG
LGFSIAGGVGNQHIPGDNSIYVTKIIDGGAAQKDGRLQVGDRLLMVNNYSLEEVTHEE
AVAILKNTSEVVYLKVGKPTTIYMTDPYGPPDITHSYSPPMENHLLSGNNGTLEYKTS
LPPISPGRYSPIPKHMLVDDDYTRPPEPVYSTVNKLCDKPASPRHYSPVECDKSFLLS
APYSHYHLGLLPDSEMTSHSQHSTATRQPSMTLQRAVSLEGEPRKVVLHKGSTGLGFN
IVGGEDGEGIFVSFILAGGPADLSGELQRGDQILSVNGIDLRGASHEQAAAALKGAGQ
TVTIIAQYQPEDYARFEAKIHDLREQMMNHSMSSGSGSLRTNQKRSLYVRAMFDYDKS
KDSGLPSQGLSFKYGDILHVINASDDEWWQARRVMLEGDSEEMGVIPSKRRVERKERA
RLKTVKFNAKPGVIDSKGSFNDKRKKSFIFSRKFPFYKNKEQSEQETSDPEQHVSSNA
SDSESSYRGQEDLILSYEPVTRQEINYTRPVIILGPMKDRINDDLISEFPDKFGSCVP
HTTRPKRDYEVDGRDYHFVISREQMEKDIQEHKFIEAGQYNDNLYGTSVQSVRFVAER
GKHCILDVSGNAIKRLQVAQLYPIAIFIKPRSLEPLMEMNKRLTEEQAKKTYDRAIKL
EQEFGEYFTAIVQGDTLEDIYNQCKLVIEEQSGPFIWIPSKEKL
```

	start	end	width	seq	name
1	1	26	26	MFASIWYAKKLGRRFVHNARKAKSEK	CFEin8_cds
2	27	44	18	ASPAPIIVNTDTLDTIPY	exon 11
3	45	61	17	VNGTEIEYEFEEITLER	exon 12
4	62	102	41	GNSGLGFSIAGGTDNPHIG..GIFITKIIPGGAAAEDGRL	exon 13
5	103	103	1	R	
6	104	159	56	VNDCILRVNEVDVSEVSHS..RRRPILETVVEIKLFKGPK	exon 14
7	160	160	1	G	
8	161	205	45	LGFSIAGGVGNQHIPGDNS..DGGAAQKDGRLQVGDRLLM	exon 15
9	206	253	48	VNNYSLEEVTHEEAVAILK..GKPTTIYMTDPYGPPDITH	exon 16
10	254	254	1	S	
11	255	299	45	YSPPMENHLLSGNNGTLEY..SPGRYSPIPKHMLVDDDYT	exon 17
12	300	300	1	R	
13	301	351	51	PPEPVYSTVNKLCDKPASP..LSAPYSHYHLGLLPDSEMT	exon 18
14	352	352	1	S	
15	353	374	22	HSQHSTATRQPSMTLQRAVSLE	exon 19
16	375	375	1	G	
17	376	427	52	EPRKVVLHKGSTGLGFNIV..AGGPADLSGELQRGDQILS	exon 20
18	428	461	34	VNGIDLRGASHEQAAAALKGAGQTVTIIAQYQPE	exon 21
19	462	462	1	D	
20	463	476	14	YARFEAKIHDLREQ	exon 23a
21	477	499	23	MMNHSMSSGSGSLRTNQKRSLYV	exon 23b
22	500	500	1	R	
23	501	558	58	AMFDYDKSKDSGLPSQGLS..RRVMLEGDSEEMGVIPSKR	exon 24
24	559	559	1	R	
25	560	584	25	VERKERARLKTVKFNAKPGVIDSKG	exon 25
26	585	617	33	SFNDKRKKSFIFSRKFPFYKNKEQSEQETSDPE	exon 26
27	618	618	1	Q	
28	619	631	13	HVSSNASDSESSY	exon 28
29	632	632	1	R	

30	633	648	16	GQEDLILSYEPVTRQE	exon 29
31	649	649	1	I	
32	650	682	33	NYTRPVIILGPMKDRINDDLISEFPDKFGSCVP	exon 30
33	683	683	1	H	
34	684	740	57	TTRPKRDYEVDGRDYHFVI..YNDNLYGTSVQSVRFVAER	exon 31
35	741	776	36	GKHCILDVSGNAIKRLQVAQLYPIAIFIKPRSLEPL	exon 32
36	777	777	1	M	
37	778	807	30	EMNKRLTEEQAKKTYDRAIKLEQEFGEYFT	exon 33
38	808	808	1	A	
39	809	842	34	IVQGDTLEDIYNQCKLVIEEQSGPFIWIPSKEKL	exon 34

XP_016872772

>XP_016872772
MFASIWYAKKLGRRFVHNARKAKSEKASPAPIIVNTDTLDTIPY
VNGTEIEYEFEEITLERGNSGLGFSIAGGTDNPHIGDDPGIFITKIIPGGAAAEDGRL
RVNDCILRVNEVDVSEVSHSKAVEALKEAGSIVRLYVRRRRPILETVVEIKLFKGPKG
LGFSIAGGVGNQHIPGDNSIYVTKIIDGGAAQKDGRLQVGDRLLMVNNYSLEEVTHEE
AVAILKNTSEVVYLKVGKPTTIYMTDPYGPPDITHSYSPPMENHLLSGNNGTLEYKTS
LPPISPGRYSPIPKHMLVDDDYTRPPEPVYSTVNKLCDKPASPRHYSPVECDKSFLLS
APYSHYHLGLLPDSEMTSHSQHSTATRQPSMTLQRAVSLEGEPRKVVLHKGSTGLGFN
IVGGEDGEGIFVSFILAGGPADLSGELQRGDQILSVNGIDLRGASHEQAAAALKGAGQ
TVTIIAQYQPEDYARFEAKIHDLREQMMNHSMSSGSGSLRTNQKRSLYVRAMFDYDKS
KDSGLPSQGLSFKYGDILHVINASDDEWWQARRVMLEGDSEEMGVIPSKRRVERKERA
RLKTVKFNAKPGVIDSKGDIPGLGDDGYGTKTLRGQEDLILSYEPVTRQEINYTRPVI
ILGPMKDRINDDLISEFPDKFGSCVPHTTRPKRDYEVDGRDYHFVISREQMEKDIQEH
KFIEAGQYNDNLYGTSVQSVRFVAERGKHCILDVSGNAIKRLQVAQLYPIAIFIKPRS
LEPLMEMNKRLTEEQAKKTYDRAIKLEQEFGEYFTAIVQGDTLEDIYNQCKLVIEEQS
GPFIWIPSKEKL

	start	end	width	seq	name
1	1	26	26	MFASIWYAKKLGRRFVHNARKAKSEK	CFEin8_cds
2	27	44	18	ASPAPIIVNTDTLDTIPY	exon 11
3	45	61	17	VNGTEIEYEFEEITLER	exon 12
4	62	102	41	GNSGLGFSIAGGTDNPHIG..GIFITKIIPGGAAAEDGRL	exon 13
5	103	103	1	R	
6	104	159	56	VNDCILRVNEVDVSEVSHS..RRRPILETVVEIKLFKGPK	exon 14
7	160	160	1	G	
8	161	205	45	LGFSIAGGVGNQHIPGDNS..DGGAAQKDGRLQVGDRLLM	exon 15
9	206	253	48	VNNYSLEEVTHEEAVAILK..GKPTTIYMTDPYGPPDITH	exon 16
10	254	254	1	S	
11	255	299	45	YSPPMENHLLSGNNGTLEY..SPGRYSPIPKHMLVDDDYT	exon 17
12	300	300	1	R	
13	301	351	51	PPEPVYSTVNKLCDKPASP..LSAPYSHYHLGLLPDSEMT	exon 18
14	352	352	1	S	
15	353	374	22	HSQHSTATRQPSMTLQRAVSLE	exon 19
16	375	375	1	G	
17	376	427	52	EPRKVVLHKGSTGLGFNIV..AGGPADLSGELQRGDQILS	exon 20
18	428	461	34	VNGIDLRGASHEQAAAALKGAGQTVTIIAQYQPE	exon 21
19	462	462	1	D	
20	463	476	14	YARFEAKIHDLREQ	exon 23a
21	477	499	23	MMNHSMSSGSGSLRTNQKRSLYV	exon 23b
22	500	500	1	R	
23	501	558	58	AMFDYDKSKDSGLPSQGLS..RRVMLEGDSEEMGVIPSKR	exon 24
24	559	559	1	R	
25	560	584	25	VERKERARLKTVKFNAKPGVIDSKG	exon 25
26	585	599	15	DIPGLGDDGYGTKTL	exon 27

27	600	600	1	R	
28	601	616	16	GQEDLILSYEPVTRQE	exon 29
29	617	617	1	I	
30	618	650	33	NYTRPVIILGPMKDRINDDLISEFPDKFGSCVP	exon 30
31	651	651	1	H	
32	652	708	57	TTRPKRDYEVDGRDYHFVI..YNDNLYGTSVQSVRFVAER	exon 31
33	709	744	36	GKHCILDVSGNAIKRLQVAQLYPIAIFIKPRSLEPL	exon 32
34	745	745	1	M	
35	746	775	30	EMNKRLTEEQAKKTYDRAIKLEQEFGEYFT	exon 33
36	776	776	1	A	
37	777	810	34	IVQGDTLEDIYNQCKLVIEEQSGPFIWIPSKEKL	exon 34

XP_016872770

```
>XP_016872770
MSPVVKDPDCFTPMICHCKVACTNNTLSLMFGCKKYRYQDEDAP
HDHSLPRLTHEVRGPELVHVSEKNLSQIENVHGYVLQSHISPLKASPAPIIVNTDTLD
TIPYVNGTEIEYEFEEITLERGNSGLGFSIAGGTDNPHIGDDPGIFITKIIPGGAAAE
DGRLRVNDCILRVNEVDVSEVSHSKAVEALKEAGSIVRLYVRRRRPILETVVEIKLFK
GPKGLGFSIAGGVGNQHIPGDNSIYVTKIIDGGAAQKDGRLQVGDRLLMVNNYSLEEV
THEEAVAILKNTSEVVYLKVGKPTTIYMTDPYGPPDITHSYSPPMENHLLSGNNGTLE
YKTSLPPISPGRYSPIPKHMLVDDDYTSHSQHSTATRQPSMTLQRAVSLEGEPRKVVL
HKGSTGLGFNIVGGEDGEGIFVSFILAGGPADLSGELQRGDQILSVNGIDLRGASHEQ
AAAALKGAGQTVTIIAQYQPEDYARFEAKIHDLREQMMNHSMSSGSGSLRTNQKRSLY
VRAMFDYDKSKDSGLPSQGLSFKYGDILHVINASDDEWWQARRVMLEGDSEEMGVIPS
KRRVERKERARLKTVKFNAKPGVIDSKGDIPGLGDDGYGTKTLRGQEDLILSYEPVTR
QEINYTRPVIILGPMKDRINDDLISEFPDKFGSCVPHTTRPKRDYEVDGRDYHFVISR
EQMEKDIQEHKFIEAGQYNDNLYGTSVQSVRFVAERGKHCILDVSGNAIKRLQVAQLY
PIAIFIKPRSLEPLMEMNKRLTEEQAKKTYDRAIKLEQEFGEYFTAIVQGDTLEDIYN
QCKLVIEEQSGPFIWIPSKEKL
```

	start	end	width	seq	name
1	1	34	34	MSPVVKDPDCFTPMICHCKVACTNNTLSLMFGCK	CFEin7_cds
2	35	88	54	KYRYQDEDAPHDHSLPRLT..SQIENVHGYVLQSHISPLK	exon 8
3	89	106	18	ASPAPIIVNTDTLDTIPY	exon 11
4	107	123	17	VNGTEIEYEFEEITLER	exon 12
5	124	164	41	GNSGLGFSIAGGTDNPHIG..GIFITKIIPGGAAAEDGRL	exon 13
6	165	165	1	R	
7	166	221	56	VNDCILRVNEVDVSEVSHS..RRRPILETVVEIKLFKGPK	exon 14
8	222	222	1	G	
9	223	267	45	LGFSIAGGVGNQHIPGDNS..DGGAAQKDGRLQVGDRLLM	exon 15
10	268	315	48	VNNYSLEEVTHEEAVAILK..GKPTTIYMTDPYGPPDITH	exon 16
11	316	316	1	S	
12	317	361	45	YSPPMENHLLSGNNGTLEY..SPGRYSPIPKHMLVDDDYT	exon 17
13	362	362	1	S	
14	363	384	22	HSQHSTATRQPSMTLQRAVSLE	exon 19
15	385	385	1	G	
16	386	437	52	EPRKVVLHKGSTGLGFNIV..AGGPADLSGELQRGDQILS	exon 20
17	438	471	34	VNGIDLRGASHEQAAAALKGAGQTVTIIAQYQPE	exon 21
18	472	472	1	D	
19	473	486	14	YARFEAKIHDLREQ	exon 23a
20	487	509	23	MMNHSMSSGSGSLRTNQKRSLYV	exon 23b
21	510	510	1	R	
22	511	568	58	AMFDYDKSKDSGLPSQGLS..RRVMLEGDSEEMGVIPSKR	exon 24
23	569	569	1	R	
24	570	594	25	VERKERARLKTVKFNAKPGVIDSKG	exon 25
25	595	609	15	DIPGLGDDGYGTKTL	exon 27

26	610	610	1	R	
27	611	626	16	GQEDLILSYEPVTRQE	exon 29
28	627	627	1	I	
29	628	660	33	NYTRPVIILGPMKDRINDDLISEFPDKFGSCVP	exon 30
30	661	661	1	H	
31	662	718	57	TTRPKRDYEVDGRDYHFVI..YNDNLYGTSVQSVRFVAER	exon 31
32	719	754	36	GKHCILDVSGNAIKRLQVAQLYPIAIFIKPRSLEPL	exon 32
33	755	755	1	M	
34	756	785	30	EMNKRLTEEQAKKTYDRAIKLEQEFGEYFT	exon 33
35	786	786	1	A	
36	787	820	34	IVQGDTLEDIYNQCKLVIEEQSGPFIWIPSKEKL	exon 34

XP_016872753

```
>XP_016872753
MSPVVKDPDCFTPMICHCKVACTNNTLSLMFGCKKYRYQDEDAP
HDHSLPRLTHEVRGPELVHVSEKNLSQIENVHGYVLQSHISPLKASPAPIIVNTDTLD
TIPYVNGTEIEYEFEEITLERGNSGLGFSIAGGTDNPHIGDDPGIFITKIIPGGAAAE
DGRLRVNDCILRVNEVDVSEVSHSKAVEALKEAGSIVRLYVRRRRPILETVVEIKLFK
GPKGLGFSIAGGVGNQHIPGDNSIYVTKIIDGGAAQKDGRLQVGDRLLMVNNYSLEEV
THEEAVAILKNTSEVVYLKVGKPTTIYMTDPYGPPDITHSYSPPMENHLLSGNNGTLE
YKTSLPPISPGRYSPIPKHMLVDDDYTRPPEPVYSTVNKLCDKPASPRHYSPVECDKS
FLLSAPYSHYHLGLLPDSEMTSHSQHSTATRQPSMTLQRAVSLEGEPRKVVLHKGSTG
LGFNIVGGEDGEGIFVSFILAGGPADLSGELQRGDQILSVNGIDLRGASHEQAAAALK
GAGQTVTIIAQYQPEDYARFEAKIHDLREQMMNHSMSSGSGSLRTNQKRSLYVRAMFD
YDKSKDSGLPSQGLSFKYGDILHVINASDDEWWQARRVMLEGDSEEMGVIPSKRRVER
KERARLKTVKFNAKPGVIDSKGSFNDKRKKSFIFSRKFPFYKNKEQSEQETSDPEQHV
SSNASDSESSYRGQEDLILSYEPVTRQEINYTRPVIILGPMKDRINDDLISEFPDKFG
SCVPHTTRPKRDYEVDGRDYHFVISREQMEKDIQEHKFIEAGQYNDNLYGTSVQSVRF
VAERGKHCILDVSGNAIKRLQVAQLYPIAIFIKPRSLEPLMEMNKRLTEEQAKKTYDR
AIKLEQEFGEYFTAIVQGDTLEDIYNQCKLVIEEQSGPFIWIPSKEKL
```

	start	end	width	seq	name
1	1	34	34	MSPVVKDPDCFTPMICHCKVACTNNTLSLMFGCK	CFEin7_cds
2	35	88	54	KYRYQDEDAPHDHSLPRLT..SQIENVHGYVLQSHISPLK	exon 8
3	89	106	18	ASPAPIIVNTDTLDTIPY	exon 11
4	107	123	17	VNGTEIEYEFEEITLER	exon 12
5	124	164	41	GNSGLGFSIAGGTDNPHIG..GIFITKIIPGGAAAEDGRL	exon 13
6	165	165	1	R	
7	166	221	56	VNDCILRVNEVDVSEVSHS..RRRPILETVVEIKLFKGPK	exon 14
8	222	222	1	G	
9	223	267	45	LGFSIAGGVGNQHIPGDNS..DGGAAQKDGRLQVGDRLLM	exon 15
10	268	315	48	VNNYSLEEVTHEEAVAILK..GKPTTIYMTDPYGPPDITH	exon 16
11	316	316	1	S	
12	317	361	45	YSPPMENHLLSGNNGTLEY..SPGRYSPIPKHMLVDDDYT	exon 17
13	362	362	1	R	
14	363	413	51	PPEPVYSTVNKLCDKPASP..LSAPYSHYHLGLLPDSEMT	exon 18
15	414	414	1	S	
16	415	436	22	HSQHSTATRQPSMTLQRAVSLE	exon 19
17	437	437	1	G	
18	438	489	52	EPRKVVLHKGSTGLGFNIV..AGGPADLSGELQRGDQILS	exon 20
19	490	523	34	VNGIDLRGASHEQAAAALKGAGQTVTIIAQYQPE	exon 21
20	524	524	1	D	
21	525	538	14	YARFEAKIHDLREQ	exon 23a
22	539	561	23	MMNHSMSSGSGSLRTNQKRSLYV	exon 23b
23	562	562	1	R	
24	563	620	58	AMFDYDKSKDSGLPSQGLS..RRVMLEGDSEEMGVIPSKR	exon 24

25	621	621	1	R	
26	622	646	25	VERKERARLKTVKFNAKPGVIDSKG	exon 25
27	647	679	33	SFNDKRKKSFIFSRKFPFYKNKEQSEQETSDPE	exon 26
28	680	680	1	Q	
29	681	693	13	HVSSNASDSESSY	exon 28
30	694	694	1	R	
31	695	710	16	GQEDLILSYEPVTRQE	exon 29
32	711	711	1	I	
33	712	744	33	NYTRPVIILGPMKDRINDDLISEFPDKFGSCVP	exon 30
34	745	745	1	H	
35	746	802	57	TTRPKRDYEVDGRDYHFVI..YNDNLYGTSVQSVRFVAER	exon 31
36	803	838	36	GKHCILDVSGNAIKRLQVAQLYPIAIFIKPRSLEPL	exon 32
37	839	839	1	M	
38	840	869	30	EMNKRLTEEQAKKTYDRAIKLEQEFGEYFT	exon 33
39	870	870	1	A	
40	871	904	34	IVQGDTLEDIYNQCKLVIEEQSGPFIWIPSKEKL	exon 34

XP_016872760

```
>XP_016872760
MSPVVKDPDCFTPMICHCKVACTNNTLSLMFGCKKYRYQDEDAP
HDHSLPRLTHEVRGPELVHVSEKNLSQIENVHGYVLQSHISPLKASPAPIIVNTDTLD
TIPYVNGTEIEYEFEEITLERGNSGLGFSIAGGTDNPHIGDDPGIFITKIIPGGAAAE
DGRLRVNDCILRVNEVDVSEVSHSKAVEALKEAGSIVRLYVRRRRPILETVVEIKLFK
GPKGLGFSIAGGVGNQHIPGDNSIYVTKIIDGGAAQKDGRLQVGDRLLMVNNYSLEEV
THEEAVAILKNTSEVVYLKVGKPTTIYMTDPYGPPDITHSYSPPMENHLLSGNNGTLE
YKTSLPPISPGRYSPIPKHMLVDDDYTRPPEPVYSTVNKLCDKPASPRHYSPVECDKS
FLLSAPYSHYHLGLLPDSEMTSHSQHSTATRQPSMTLQRAVSLEGEPRKVVLHKGSTG
LGFNIVGGEDGEGIFVSFILAGGPADLSGELQRGDQILSVNGIDLRGASHEQAAAALK
GAGQTVTIIAQYQPEDYARFEAKIHDLREQMMNHSMSSGSGSLRTNQKRSLYVRAMFD
YDKSKDSGLPSQGLSFKYGDILHVINASDDEWWQARRVMLEGDSEEMGVIPSKRRVER
KERARLKTVKFNAKPGVIDSKGDIPGLGDDGYGTKTLRGQEDLILSYEPVTRQEINYT
RPVIILGPMKDRINDDLISEFPDKFGSCVPHTTRPKRDYEVDGRDYHFVISREQMEKD
IQEHKFIEAGQYNDNLYGTSVQSVRFVAERGKHCILDVSGNAIKRLQVAQLYPIAIFI
KPRSLEPLMEMNKRLTEEQAKKTYDRAIKLEQEFGEYFTAIVQGDTLEDIYNQCKLVI
EEQSGPFIWIPSKEKL
```

	start	end	width	seq	name
1	1	34	34	MSPVVKDPDCFTPMICHCKVACTNNTLSLMFGCK	CFEin7_cds
2	35	88	54	KYRYQDEDAPHDHSLPRLT..SQIENVHGYVLQSHISPLK	exon 8
3	89	106	18	ASPAPIIVNTDTLDTIPY	exon 11
4	107	123	17	VNGTEIEYEFEEITLER	exon 12
5	124	164	41	GNSGLGFSIAGGTDNPHIG..GIFITKIIPGGAAAEDGRL	exon 13
6	165	165	1	R	
7	166	221	56	VNDCILRVNEVDVSEVSHS..RRRPILETVVEIKLFKGPK	exon 14
8	222	222	1	G	
9	223	267	45	LGFSIAGGVGNQHIPGDNS..DGGAAQKDGRLQVGDRLLM	exon 15
10	268	315	48	VNNYSLEEVTHEEAVAILK..GKPTTIYMTDPYGPPDITH	exon 16
11	316	316	1	S	
12	317	361	45	YSPPMENHLLSGNNGTLEY..SPGRYSPIPKHMLVDDDYT	exon 17
13	362	362	1	R	
14	363	413	51	PPEPVYSTVNKLCDKPASP..LSAPYSHYHLGLLPDSEMT	exon 18
15	414	414	1	S	
16	415	436	22	HSQHSTATRQPSMTLQRAVSLE	exon 19
17	437	437	1	G	
18	438	489	52	EPRKVVLHKGSTGLGFNIV..AGGPADLSGELQRGDQILS	exon 20
19	490	523	34	VNGIDLRGASHEQAAAALKGAGQTVTIIAQYQPE	exon 21

20	524	524	1	D	
21	525	538	14	YARFEAKIHDLREQ	exon 23a
22	539	561	23	MMNHSMSSGSGSLRTNQKRSLYV	exon 23b
23	562	562	1	R	
24	563	620	58	AMFDYDKSKDSGLPSQGLS..RRVMLEGDSEEMGVIPSKR	exon 24
25	621	621	1	R	
26	622	646	25	VERKERARLKTVKFNAKPGVIDSKG	exon 25
27	647	661	15	DIPGLGDDGYGTKTL	exon 27
28	662	662	1	R	
29	663	678	16	GQEDLILSYEPVTRQE	exon 29
30	679	679	1	I	
31	680	712	33	NYTRPVIILGPMKDRINDDLISEFPDKFGSCVP	exon 30
32	713	713	1	H	
33	714	770	57	TTRPKRDYEVDGRDYHFVI..YNDNLYGTSVQSVRFVAER	exon 31
34	771	806	36	GKHCILDVSGNAIKRLQVAQLYPIAIFIKPRSLEPL	exon 32
35	807	807	1	M	
36	808	837	30	EMNKRLTEEQAKKTYDRAIKLEQEFGEYFT	exon 33
37	838	838	1	A	
38	839	872	34	IVQGDTLEDIYNQCKLVIEEQSGPFIWIPSKEKL	exon 34

XP_016872754

```
>XP_016872754
MSPVVKDPDCFTPMICHCKVACTNNTLSLMFGCKYRYQDEDAPH
DHSLPRLTHEVRGPELVHVSEKNLSQIENVHGYVLQSHISPLKASPAPIIVNTDTLDT
IPYVNGTEIEYEFEEITLERGNSGLGFSIAGGTDNPHIGDDPGIFITKIIPGGAAAED
GRLRVNDCILRVNEVDVSEVSHSKAVEALKEAGSIVRLYVRRRRPILETVVEIKLFKG
PKGLGFSIAGGVGNQHIPGDNSIYVTKIIDGGAAQKDGRLQVGDRLLMVNNYSLEEVT
HEEAVAILKNTSEVVYLKVGKPTTIYMTDPYGPPDITHSYSPPMENHLLSGNNGTLEY
KTSLPPISPGRYSPIPKHMLVDDDYTRPPEPVYSTVNKLCDKPASPRHYSPVECDKSF
LLSAPYSHYHLGLLPDSEMTSHSQHSTATRQPSMTLQRAVSLEGEPRKVVLHKGSTGL
GFNIVGGEDGEGIFVSFILAGGPADLSGELQRGDQILSVNGIDLRGASHEQAAAALKG
AGQTVTIIAQYQPEDYARFEAKIHDLREQMMNHSMSSGSGSLRTNQKRSLYVRAMFDY
DKSKDSGLPSQGLSFKYGDILHVINASDDEWWQARRVMLEGDSEEMGVIPSKRRVERK
ERARLKTVKFNAKPGVIDSKGSFNDKRKKSFIFSRKFPFYKNKEQSEQETSDPEQHVS
SNASDSESSYRGQEDLILSYEPVTRQEINYTRPVIILGPMKDRINDDLISEFPDKFGS
CVPHTTRPKRDYEVDGRDYHFVISREQMEKDIQEHKFIEAGQYNDNLYGTSVQSVRFV
AERGKHCILDVSGNAIKRLQVAQLYPIAIFIKPRSLEPLMEMNKRLTEEQAKKTYDRA
IKLEQEFGEYFTAIVQGDTLEDIYNQCKLVIEEQSGPFIWIPSKEKL
```

	start	end	width	seq	name
1	1	34	34	MSPVVKDPDCFTPMICHCKVACTNNTLSLMFGCK	CFEin7_cds
2	34	87	54	KYRYQDEDAPHDHSLPRLT..SQIENVHGYVLQSHISPLK	exon 8
3	88	105	18	ASPAPIIVNTDTLDTIPY	exon 11
4	106	122	17	VNGTEIEYEFEEITLER	exon 12
5	123	163	41	GNSGLGFSIAGGTDNPHIG..GIFITKIIPGGAAAEDGRL	exon 13
6	164	164	1	R	
7	165	220	56	VNDCILRVNEVDVSEVSHS..RRRPILETVVEIKLFKGPK	exon 14
8	221	221	1	G	
9	222	266	45	LGFSIAGGVGNQHIPGDNS..DGGAAQKDGRLQVGDRLLM	exon 15
10	267	314	48	VNNYSLEEVTHEEAVAILK..GKPTTIYMTDPYGPPDITH	exon 16
11	315	315	1	S	
12	316	360	45	YSPPMENHLLSGNNGTLEY..SPGRYSPIPKHMLVDDDYT	exon 17
13	361	361	1	R	
14	362	412	51	PPEPVYSTVNKLCDKPASP..LSAPYSHYHLGLLPDSEMT	exon 18
15	413	413	1	S	
16	414	435	22	HSQHSTATRQPSMTLQRAVSLE	exon 19

17	436	436	1	G	
18	437	488	52	EPRKVVLHKGSTGLGFNIV..AGGPADLSGELQRGDQILS	exon 20
19	489	522	34	VNGIDLRGASHEQAAAALKGAGQTVTIIAQYQPE	exon 21
20	523	523	1	D	
21	524	537	14	YARFEAKIHDLREQ	exon 23a
22	538	560	23	MMNHSMSSGSGSLRTNQKRSLYV	exon 23b
23	561	561	1	R	
24	562	619	58	AMFDYDKSKDSGLPSQGLS..RRVMLEGDSEEMGVIPSKR	exon 24
25	620	620	1	R	
26	621	645	25	VERKERARLKTVKFNAKPGVIDSKG	exon 25
27	646	678	33	SFNDKRKKSFIFSRKFPFYKNKEQSEQETSDPE	exon 26
28	679	679	1	Q	
29	680	692	13	HVSSNASDSESSY	exon 28
30	693	693	1	R	
31	694	709	16	GQEDLILSYEPVTRQE	exon 29
32	710	710	1	I	
33	711	743	33	NYTRPVIILGPMKDRINDDLISEFPDKFGSCVP	exon 30
34	744	744	1	H	
35	745	801	57	TTRPKRDYEVDGRDYHFVI..YNDNLYGTSVQSVRFVAER	exon 31
36	802	837	36	GKHCILDVSGNAIKRLQVAQLYPIAIFIKPRSLEPL	exon 32
37	838	838	1	M	
38	839	868	30	EMNKRLTEEQAKKTYDRAIKLEQEFGEYFT	exon 33
39	869	869	1	A	
40	870	903	34	IVQGDTLEDIYNQCKLVIEEQSGPFIWIPSKEKL	exon 34

XP_016872761

```
>XP_016872761
MSPVVKDPDCFTPMICHCKVACTNNTLSLMFGCKYRYQDEDAPH
DHSLPRLTHEVRGPELVHVSEKNLSQIENVHGYVLQSHISPLKASPAPIIVNTDTLDT
IPYVNGTEIEYEFEEITLERGNSGLGFSIAGGTDNPHIGDDPGIFITKIIPGGAAAED
GRLRVNDCILRVNEVDVSEVSHSKAVEALKEAGSIVRLYVRRRRPILETVVEIKLFKG
PKGLGFSIAGGVGNQHIPGDNSIYVTKIIDGGAAQKDGRLQVGDRLLMVNNYSLEEVT
HEEAVAILKNTSEVVYLKVGKPTTIYMTDPYGPPDITHSYSPPMENHLLSGNNGTLEY
KTSLPPISPGRYSPIPKHMLVDDDYTRPPEPVYSTVNKLCDKPASPRHYSPVECDKSF
LLSAPYSHYHLGLLPDSEMTSHSQHSTATRQPSMTLQRAVSLEGEPRKVVLHKGSTGL
GFNIVGGEDGEGIFVSFILAGGPADLSGELQRGDQILSVNGIDLRGASHEQAAAALKG
AGQTVTIIAQYQPEDYARFEAKIHDLREQMMNHSMSSGSGSLRTNQKRSLYVRAMFDY
DKSKDSGLPSQGLSFKYGDILHVINASDDEWWQARRVMLEGDSEEMGVIPSKRRVERK
ERARLKTVKFNAKPGVIDSKGDIPGLGDDGYGTKTLRGQEDLILSYEPVTRQEINYTR
PVIILGPMKDRINDDLISEFPDKFGSCVPHTTRPKRDYEVDGRDYHFVISREQMEKDI
QEHKFIEAGQYNDNLYGTSVQSVRFVAERGKHCILDVSGNAIKRLQVAQLYPIAIFIK
PRSLEPLMEMNKRLTEEQAKKTYDRAIKLEQEFGEYFTAIVQGDTLEDIYNQCKLVIE
EQSGPFIWIPSKEKL
```

	start	end	width	seq	name
1	1	34	34	MSPVVKDPDCFTPMICHCKVACTNNTLSLMFGCK	CFEin7_cds
2	34	87	54	KYRYQDEDAPHDHSLPRLT..SQIENVHGYVLQSHISPLK	exon 8
3	88	105	18	ASPAPIIVNTDTLDTIPY	exon 11
4	106	122	17	VNGTEIEYEFEEITLER	exon 12
5	123	163	41	GNSGLGFSIAGGTDNPHIG..GIFITKIIPGGAAAEDGRL	exon 13
6	164	164	1	R	
7	165	220	56	VNDCILRVNEVDVSEVSHS..RRRPILETVVEIKLFKGPK	exon 14
8	221	221	1	G	
9	222	266	45	LGFSIAGGVGNQHIPGDNS..DGGAAQKDGRLQVGDRLLM	exon 15
10	267	314	48	VNNYSLEEVTHEEAVAILK..GKPTTIYMTDPYGPPDITH	exon 16
11	315	315	1	S	

12	316	360	45	YSPPMENHLLSGNNGTLEY..SPGRYSPIPKHMLVDDDYT	exon 17
13	361	361	1	R	
14	362	412	51	PPEPVYSTVNKLCDKPASP..LSAPYSHYHLGLLPDSEMT	exon 18
15	413	413	1	S	
16	414	435	22	HSQHSTATRQPSMTLQRAVSLE	exon 19
17	436	436	1	G	
18	437	488	52	EPRKVVLHKGSTGLGFNIV..AGGPADLSGELQRGDQILS	exon 20
19	489	522	34	VNGIDLRGASHEQAAAALKGAGQTVTIIAQYQPE	exon 21
20	523	523	1	D	
21	524	537	14	YARFEAKIHDLREQ	exon 23a
22	538	560	23	MMNHSMSSGSGSLRTNQKRSLYV	exon 23b
23	561	561	1	R	
24	562	619	58	AMFDYDKSKDSGLPSQGLS..RRVMLEGDSEEMGVIPSKR	exon 24
25	620	620	1	R	
26	621	645	25	VERKERARLKTVKFNAKPGVIDSKG	exon 25
27	646	660	15	DIPGLGDDGYGTKTL	exon 27
28	661	661	1	R	
29	662	677	16	GQEDLILSYEPVTRQE	exon 29
30	678	678	1	I	
31	679	711	33	NYTRPVIILGPMKDRINDDLISEFPDKFGSCVP	exon 30
32	712	712	1	H	
33	713	769	57	TTRPKRDYEVDGRDYHFVI..YNDNLYGTSVQSVRFVAER	exon 31
34	770	805	36	GKHCILDVSGNAIKRLQVAQLYPIAIFIKPRSLEPL	exon 32
35	806	806	1	M	
36	807	836	30	EMNKRLTEEQAKKTYDRAIKLEQEFGEYFT	exon 33
37	837	837	1	A	
38	838	871	34	IVQGDTLEDIYNQCKLVIEEQSGPFIWIPSKEKL	exon 34

Supplementary Note 6

We report the forward (F) primers used in [12] to target *Dlg2* isoform in brain mouse, along with their BLAST alignments in the mouse genome reference.

RT-PCR primers

PSD-93 zeta F (Q91XM9-7)

```
 Mus musculus strain C57BL/6J chromosome 7, GRCm38.p4 C57BL/6J
Sequence ID: NC_000073.6 Length: 145441459 Number of Matches: 248
Range 1: 90504814 to 90504835
Alignment statistics for match #1
Score Expect Identities Gaps Strand
44.1 bits(22)  8e-04  22/22(100%)  0/22(0%)  Plus/Plus
Features:
disks large homolog 2 isoform X8
disks large homolog 2 isoform X3

Query  1         CGAGCTTTGTCATTACTGGAGG  22
                 ||||||||||||||||||||||
Sbjct  90504814  CGAGCTTTGTCATTACTGGAGG  90504835
```

PSD-93 epsilon F (Q91XM9-6)

```
 Mus musculus strain C57BL/6J chromosome 7, GRCm38.p4 C57BL/6J
Sequence ID: NC_000073.6 Length: 145441459 Number of Matches: 548
Range 1: 91542839 to 91542861
Alignment statistics for match #1
Score Expect Identities Gaps Strand
46.1 bits(23)  2e-04  23/23(100%)  0/23(0%)  Plus/Plus
Features:
disks large homolog 2 isoform X8
disks large homolog 2 isoform X3

Query  1         GCCAACTGGATGTGTGTGAGCCG  23
                 |||||||||||||||||||||||
Sbjct  91542839  GCCAACTGGATGTGTGTGAGCCG  91542861
```

PSD-93 beta F (Q91XM9-2)

```
 Mus musculus strain C57BL/6J chromosome 7, GRCm38.p4 C57BL/6J
Sequence ID: NC_000073.6 Length: 145441459 Number of Matches: 12
Range 1: 91262788 to 91262809
Alignment statistics for match #1
Score Expect Identities Gaps Strand
36.2 bits(18)  0.19  21/22(95%)  0/22(0%)  Plus/Plus
Features:
disks large homolog 2 isoform X8
disks large homolog 2 isoform X3

Query  1         AGCTGCCGCTCGGTCTAGGCTG  22
                 ||||||||||| ||||||||||
Sbjct  91262788  AGCTGCCGCTCTGTCTAGGCTG  91262809
```

PSD-93 gamma F (Q91XM9-3)

```
 Mus musculus strain C57BL/6J chromosome 7, GRCm38.p4 C57BL/6J
Sequence ID: NC_000073.6 Length: 145441459 Number of Matches: 202
```

```
Range 1: 91711767 to 91711790
Alignment statistics for match #1
Score Expect Identities Gaps Strand
40.1 bits(20)  0.017  23/24(96%)  0/24(0%)  Plus/Plus
Features:
disks large homolog 2 isoform X8
disks large homolog 2 isoform X3

Query  1         GTGAAGAAGCTATGCAACATGCGT  24
                 ||||||||||||||||||| ||||
Sbjct  91711767  GTGAAGAAGCTATGCAACACGCGT  91711790
```

Supplementary Note 7

Regarding CNVs belonging to GDD/ID datasets and affecting *DLG2* gene, we performed an enrichment analysis comparing cases and controls populations on (not) affecting any HPs. We discarded from cases *nssv3460188_unk* (15mbp longer), *nssv3461505_unk* (affecting many other genes on the right-size of *DLG2*); from control dataset a large serie of patients bearing a small common CNV (Figure S6): *nssv3502892_unk*, *nssv3510377_unk*, *nssv3510893_unk*, *nssv3519923_unk*, *nssv3504449_unk*, *nssv3504011_unk*, *nssv3511347_unk*, *nssv3512973_unk*, *nssv3520252_unk*, *nssv3522055_unk*, *nssv3710675_unk*, *nssv3710676_unk*, *nssv3710677_unk*, *nssv3710678_unk*, *nssv3710679_unk*, *nssv3710680_unk*, *nssv3512285_unk*, *nssv779859_unk*, *nssv779860_unk*, *nssv1176187_unk*, *nssv779861_unk*, *nssv779862_unk*, *nssv779863_unk*, *nssv779865_unk*, *nssv779866_unk*.

Supplementary Note 8

We used the following Array-CGH method to diagnose the ULB patients:

- TYPE: whole genome analysis on Agilent oligonucleotide probe array
- SLIDE: Cytochip oligo 4x180K ISCA (Bluegnome)
- ANALYSIS PROGRAM: BlueFuse Multi (Bluegnome)
- RESOLUTION: average resolution on genome: 200kbp. Resolution increased in pathogenic regions defined by "International Standard Cytogenomic Array" consortium (ISCA)
- GENOME ASSEMBLY: hg19/GRCh37

Supplementary Note 9

Here below are reported the patients mentioned in the discussion section. In the recent DECIPHER version (December 2016) there are five additional patients with deletions in *DLG2*. Patients 257014 and 314659 have NDD phenotypes, while for patients 325807 and 331366 clinical data is missing. We do not consider patient 301626 because its deletion affecting *DLG2* also alters nearby genes. From the independent Signature Genomic Laboratories dataset [13], six patients were reported with deletions affecting *DLG2*. Three patients (GC8406, GC33254, GC43330) are reported with cognitive phenotype and one with anxiety disorder at 5 years old (GC53207).

Supplementary Note 10

Q91XM9-3 (PSD-93 gamma)

Unmapped amino acid sequence (first 34 amino acids).

```
>
MQRPSVSRAENYQLLWDTIASLKQCEQAMQHAFIP
```

TBLASTN to Mus musculus (taxid:10090) reported one relevant mapping, chr7:91711694-91711798.

```
 Mus musculus chromosome 7, clone RP24-69L13, complete sequence
Sequence ID: AC121261.9 Length: 163319 Number of Matches: 1
Range 1: 68556 to 68660
Alignment statistics for match #1
Score Expect Method Identities Positives Gaps Frame
73.2 bits(178)  1e-15  Compositional matrix adjust.  33/35(94%)  34/35(97%)  0/35(0%)  +3

Query  1      MQRPSVSRAENYQLLWDTIASLKQCEQAMQHAFIP  35
              MQRPS SRAENYQLLWDTIASLKQCE+AMQHAFIP
Sbjct  68556  MQRPSASRAENYQLLWDTIASLKQCEEAMQHAFIP  68660
```

Subject sequence

```
>AC121261.9 Mus musculus chromosome 7, clone RP24-69L13, complete sequence
ATGCAACGGCCAAGTGCTTCCCGAGCTGAGAATTATCAGCTTCTGTGGGATACAATTGCTTCTTTAAAACAATGTGAAGA
AGCTATGCAACACGCGTTCATTCCG
```

BLASTN result

```
 Mus musculus strain C57BL/6J chromosome 7, GRCm38.p4 C57BL/6J
Sequence ID: NC_000073.6 Length: 145441459 Number of Matches: 2
Range 1: 91711694 to 91711798
Alignment statistics for match #1
Score Expect Identities Gaps Strand
190 bits(210)  9e-47  105/105(100%)  0/105(0%)  Plus/Plus
Features:
disks large homolog 2 isoform X8
disks large homolog 2 isoform X3

Query  1         ATGCAACGGCCAAGTGCTTCCCGAGCTGAGAATTATCAGCTTCTGTGGGATACAATTGCT  60
                 ||||||||||||||||||||||||||||||||||||||||||||||||||||||||||||
Sbjct  91711694  ATGCAACGGCCAAGTGCTTCCCGAGCTGAGAATTATCAGCTTCTGTGGGATACAATTGCT  91711753

Query  61        TCTTTAAAACAATGTGAAGAAGCTATGCAACACGCGTTCATTCCG  105
                 |||||||||||||||||||||||||||||||||||||||||||||
Sbjct  91711754  TCTTTAAAACAATGTGAAGAAGCTATGCAACACGCGTTCATTCCG  91711798
```

Q91XM9-7 (PSD-93 zeta)

Unmapped amino acid sequence (first 156 amino acids).

```
>
MPVKKKDTDRALSLLEEYCKKLRKPEEQLLKNAVKKVMSIFKSSLFQALLDIQEFYEVTLLNSQKSCEQKIEEANHVAQK
WEKTLLLDSCRDSLQKSSEHASCSGPKENALYIEQNKENQCSENETEEKTCQNQGKCPAQNCSVEAPTWMPVHHCT
```

TBLASTN to Mus musculus (taxid:10090) reported four relevant mappings. For each one we completed the analysis with the its genomic coordinates in mm10.

First region, mapping into chr7:90504805-90504936 (mm10)

TBLASTN result

```
 Mus musculus BAC clone RP24-335H15 from 7, complete sequence
Sequence ID: AC122002.2 Length: 114070 Number of Matches: 1
Range 1: 93663 to 93800
Next Match
Previous Match
Alignment statistics for match #1
Score Expect Method Identities Positives Gaps Frame
89.4 bits(220)  4e-19  Compositional matrix adjust.  44/46(96%)  45/46(97%)  0/46(0%)  -3

Query  7      DTDRALSLLEEYCKKLRKPEEQLLKNAVKKVMSIFKSSLFQALLDI  52
              DTDRALSLLEEYCKKLRKPEEQLLKNAVKKVMSIFKSSLFQALL +
Sbjct  93800  DTDRALSLLEEYCKKLRKPEEQLLKNAVKKVMSIFKSSLFQALLGM  93663
```

Subject sequence

```
>AC122002.2 Mus musculus BAC clone RP24-335H15 from 7, complete sequence
GATACTGACCGAGCTTTGTCATTACTGGAGGAATACTGCAAAAAATTAAGAAAGCCTGAGGAACAGCTGTTGAAAAATGC
TGTGAAAAAGGTGATGAGTATTTTCAAGAGCAGCTTATTTCAAGCCTTACTGGGTATG
```

Subject sequence having perfect match (last 44 amino acids to nucleotides)

```
>
GATACTGACCGAGCTTTGTCATTACTGGAGGAATACTGCAAAAAATTAAGAAAGCCTGAGGAACAGCTGTTGAAAAATGC
TGTGAAAAAGGTGATGAGTATTTTCAAGAGCAGCTTATTTCAAGCCTTACTG
```

BLASTN result

```
 Mus musculus strain C57BL/6J chromosome 7, GRCm38.p4 C57BL/6J
Sequence ID: NC_000073.6 Length: 145441459 Number of Matches: 2
Range 1: 90504805 to 90504936
Alignment statistics for match #1
Score Expect Identities Gaps Strand
239 bits(264)  3e-61  132/132(100%)  0/132(0%)  Plus/Plus
Features:
disks large homolog 2 isoform X8
disks large homolog 2 isoform X3

Query  1         GATACTGACCGAGCTTTGTCATTACTGGAGGAATACTGCAAAAAATTAAGAAAGCCTGAG  60
                 ||||||||||||||||||||||||||||||||||||||||||||||||||||||||||||
Sbjct  90504805  GATACTGACCGAGCTTTGTCATTACTGGAGGAATACTGCAAAAAATTAAGAAAGCCTGAG  90504864

Query  61        GAACAGCTGTTGAAAAATGCTGTGAAAAAGGTGATGAGTATTTTCAAGAGCAGCTTATTT  120
                 ||||||||||||||||||||||||||||||||||||||||||||||||||||||||||||
Sbjct  90504865  GAACAGCTGTTGAAAAATGCTGTGAAAAAGGTGATGAGTATTTTCAAGAGCAGCTTATTT  90504924

Query  121       CAAGCCTTACTG  132
                 ||||||||||||
Sbjct  90504925  CAAGCCTTACTG  90504936
```

Second region, mapping into chr7:90731894-90732040 (mm10)

TBLASTN result

```
 Mus musculus chromosome 7, clone RP24-136D20, complete sequence
Sequence ID: AC101784.7 Length: 169423 Number of Matches: 1
Range 1: 133447 to 133593
```

```
Next Match
Previous Match
Alignment statistics for match #1
Score Expect Method Identities Positives Gaps Frame
99.8 bits(247)  8e-23  Compositional matrix adjust.  49/49(100%)  49/49(100%)  0/49(0%)  +1

Query  51      DIQEFYEVTLLNSQKSCEQKIEEANHVAQKWEKTLLLDSCRDSLQKSSE  99
               DIQEFYEVTLLNSQKSCEQKIEEANHVAQKWEKTLLLDSCRDSLQKSSE
Sbjct  133447  DIQEFYEVTLLNSQKSCEQKIEEANHVAQKWEKTLLLDSCRDSLQKSSE  133593
```

Subject sequence

```
>AC101784.7 Mus musculus chromosome 7, clone RP24-136D20, complete sequence
GATATTCAAGAATTTTATGAGGTAACGCTATTAAATTCTCAAAAAAGTTGCGAGCAGAAGATAGAAGAAGCCAATCACGT
GGCACAGAAATGGGAGAAGACTCTCCTCCTTGATTCATGTCGTGACAGTCTTCAAAAATCCTCAGAG
```

BLASTN result

```
>AC101784.7 Mus musculus chromosome 7, clone RP24-136D20, complete sequence
GATATTCAAGAATTTTATGAGGTAACGCTATTAAATTCTCAAAAAAGTTGCGAGCAGAAGATAGAAGAAGCCAATCACGT
GGCACAGAAATGGGAGAAGACTCTCCTCCTTGATTCATGTCGTGACAGTCTTCAAAAATCCTCAGAG

 Mus musculus strain C57BL/6J chromosome 7, GRCm38.p4 C57BL/6J
Sequence ID: NC_000073.6 Length: 145441459 Number of Matches: 2
Range 1: 90731894 to 90732040
Next Match
Previous Match
Alignment statistics for match #1
Score Expect Identities Gaps Strand
266 bits(294)  2e-69  147/147(100%)  0/147(0%)  Plus/Plus
Features:
disks large homolog 2 isoform X8
disks large homolog 2 isoform X3

Query  1         GATATTCAAGAATTTTATGAGGTAACGCTATTAAATTCTCAAAAAAGTTGCGAGCAGAAG  60
                 ||||||||||||||||||||||||||||||||||||||||||||||||||||||||||||
Sbjct  90731894  GATATTCAAGAATTTTATGAGGTAACGCTATTAAATTCTCAAAAAAGTTGCGAGCAGAAG  90731953

Query  61        ATAGAAGAAGCCAATCACGTGGCACAGAAATGGGAGAAGACTCTCCTCCTTGATTCATGT  120
                 ||||||||||||||||||||||||||||||||||||||||||||||||||||||||||||
Sbjct  90731954  ATAGAAGAAGCCAATCACGTGGCACAGAAATGGGAGAAGACTCTCCTCCTTGATTCATGT  90732013

Query  121       CGTGACAGTCTTCAAAAATCCTCAGAG  147
                 |||||||||||||||||||||||||||
Sbjct  90732014  CGTGACAGTCTTCAAAAATCCTCAGAG  90732040
```

Third region, mapping into chr7:90852668-90852763 (mm10)

TBLASTN result

```
 Mus musculus BAC clone RP24-267C3 from chromosome 7, complete sequence
Sequence ID: AC140196.3 Length: 183537 Number of Matches: 2
Range 1: 139802 to 140005
Alignment statistics for match #1 Score Expect Method Identities Positives Gaps Frame
72.0 bits(175)  4e-13  Compositional matrix adjust.  43/69(62%)  48/69(69%)  7/69(10%)  -3

Query  69      QKIEEANHVAQKWEKTLLLDSCRDSLQ--KSSE----HASCSGPKENALYIEQNKENQCS  122
               Q ++   HV + W KTL L+    S+    SS     HASCSGPKENALYIEQNKENQCS
Sbjct  140005  QALDVLRHVLKSW-KTLNLNYRNVSIDF*TSSSLPL*HASCSGPKENALYIEQNKENQCS  139829
```

```
Query  123     ENETEEKTC  131
               ENETEEKTC
Sbjct  139828  ENETEEKTC  139802
```

Subject sequence

```
>AC140196.3 Mus musculus BAC clone RP24-267C3 from chromosome 7, complete sequence
CAGGCTTTAGATGTTTTAAGGCATGTGTTAAAATCTTGGAAAACACTTAATTTGAATTACAGAAACGTAAGTATTGATTT
TTAAACTTCTTCCTCCCTCCCTCTTTAGCATGCAAGTTGCAGTGGGCCAAAGGAAAATGCTTTATACATTGAGCAAAATA
AAGAAAACCAGTGTTCTGAGAATGAAACTGAAGAAAAGACGTGT
```

Subject sequence having perfect match (44 amino acids to nucleotides)

```
>
CATGCAAGTTGCAGTGGGCCAAAGGAAAATGCTTTATACATTGAGCAAAATAAAGAAAACCAGTGTTCTGAGAATGAAAC
TGAAGAAAAGACGTGT
```

BLASTN result

```
 Mus musculus strain C57BL/6J chromosome 7, GRCm38.p4 C57BL/6J
Sequence ID: NC_000073.6 Length: 145441459 Number of Matches: 2
Range 1: 90852668 to 90852763
Alignment statistics for match #1
Score Expect Identities Gaps Strand
174 bits(192)  7e-42  96/96(100%)  0/96(0%)  Plus/Plus
Features:
disks large homolog 2 isoform X8
disks large homolog 2 isoform X3

Query  1         CATGCAAGTTGCAGTGGGCCAAAGGAAAATGCTTTATACATTGAGCAAAATAAAGAAAAC  60
                 ||||||||||||||||||||||||||||||||||||||||||||||||||||||||||||
Sbjct  90852668  CATGCAAGTTGCAGTGGGCCAAAGGAAAATGCTTTATACATTGAGCAAAATAAAGAAAAC  90852727

Query  61        CAGTGTTCTGAGAATGAAACTGAAGAAAAGACGTGT  96
                 ||||||||||||||||||||||||||||||||||||
Sbjct  90852728  CAGTGTTCTGAGAATGAAACTGAAGAAAAGACGTGT  90852763
```

Fourth region, mapping into chr7:90915460-90915534 (mm10)

TBLASTN result

```
 Mus musculus BAC clone RP24-267C3 from chromosome 7, complete sequence
Sequence ID: AC140196.3 Length: 183537 Number of Matches: 2
Range 2: 77031 to 77105
Alignment statistics for match #2
Score Expect Method Identities Positives Gaps Frame
61.2 bits(147)  2e-09  Compositional matrix adjust.  25/25(100%)  25/25(100%)  0/25(0%)  -2

Query  132    QNQGKCPAQNCSVEAPTWMPVHHCT  156
              QNQGKCPAQNCSVEAPTWMPVHHCT
Sbjct  77105  QNQGKCPAQNCSVEAPTWMPVHHCT  77031
```

Subject sequence

```
>AC140196.3 Mus musculus BAC clone RP24-267C3 from chromosome 7, complete sequence
CAAAACCAAGGCAAATGCCCAGCCCAGAACTGTTCAGTGGAAGCCCCTACCTGGATGCCTGTCCACCACTGTACT
```

BLASTN result

Mus musculus strain C57BL/6J chromosome 7, GRCm38.p4 C57BL/6J
Sequence ID: NC_000073.6 Length: 145441459 Number of Matches: 2
Range 1: 90915460 to 90915534
Alignment statistics for match #1
Score Expect Identities Gaps Strand
136 bits(150) 1e-30 75/75(100%) 0/75(0%) Plus/Plus
Features:
disks large homolog 2 isoform X8
disks large homolog 2 isoform X3

```
Query  1         CAAAACCAAGGCAAATGCCCAGCCCAGAACTGTTCAGTGGAAGCCCCTACCTGGATGCCT  60
                 ||||||||||||||||||||||||||||||||||||||||||||||||||||||||||||
Sbjct  90915460  CAAAACCAAGGCAAATGCCCAGCCCAGAACTGTTCAGTGGAAGCCCCTACCTGGATGCCT  90915519

Query  61        GTCCACCACTGTACT  75
                 |||||||||||||||
Sbjct  90915520  GTCCACCACTGTACT  90915534
```

References

[1] Cooper, G. M., Coe, B. P., Girirajan, S., Rosenfeld, J. A., Vu, T. H., Baker, C., Williams, C., Stalker, H., Hamid, R., Hannig, V., et al. (2011). A copy number variation morbidity map of developmental delay. Nat. Genet. *43*, 838–846.

[2] Coe, B. P., Witherspoon, K., Rosenfeld, J. A., van Bon, B. W. M., Vulto-van Silfhout, A. T., Bosco, P., Friend, K. L., Baker, C., Buono, S., Vissers, L. E. L. M., et al. (2014). Refining analyses of copy number variation identifies specific genes associated with developmental delay. Nat Genet *46*, 1063–1071.

[3] Yao, P., Lin, P., Gokoolparsadh, A., Assareh, A., Thang, M. W. C., and Voineagu, I. (2015). Coexpression networks identify brain region-specific enhancer RNAs in the human brain. Nature neuroscience *18*, 1168–1174.

[4] Scotti, M. M. and Swanson, M. S. (2016). RNA mis-splicing in disease. Nat. Rev. Genet. *17*, 19–32.

[5] Padgett, R. A. (2012). New connections between splicing and human disease. Trends Genet. *28*, 147–154.

[6] Sakharkar, M. K., Chow, V. T., and Kangueane, P. (2004). Distributions of exons and introns in the human genome. In Silico Biol. (Gedrukt) *4*, 387–393.

[7] Ameur, A., Zaghlool, A., Halvardson, J., Wetterbom, A., Gyllensten, U., Cavelier, L., and Feuk, L. (2011). Total RNA sequencing reveals nascent transcription and widespread co-transcriptional splicing in the human brain. Nat Struct Mol Biol *18*, 1435–1440.

[8] Souvorov, A., Kapustin, Y., Kiryutin, B., Chetvernin, V., Tatusova, T., and Lipman, D. (2010). Gnomon the NCBI eukaryotic gene prediction tool. Accessed Dec 2016.

[9] Stamatoyannopoulos, J. A. (2010). Illuminating eukaryotic transcription start sites. Nature methods *7*, 501–503.

[10] Sudmant, P. H., Rausch, T., Gardner, E. J., Handsaker, R. E., Abyzov, A., Huddleston, J., Zhang, Y., Ye, K., Jun, G., Hsi-Yang Fritz, M., et al. (2015). An integrated map of structural variation in 2,504 human genomes. Nature *526*, 75–81.

[11] Dousse, A., Junier, T., and Zdobnov, E. M. (2015). CEGA-a catalog of conserved elements from genomic alignments. Nucleic acids research.

[12] Parker, M. J. (2004). PSD93 Regulates Synaptic Stability at Neuronal Cholinergic Synapses. Journal of Neuroscience *24*, 378–388.

[13] Sahoo, T., Theisen, A., Rosenfeld, J. A., Lamb, A. N., Ravnan, J. B., Schultz, R. A., Torchia, B. S., Neill, N., Casci, I., Bejjani, B. A., et al. (2011). Copy number variants of schizophrenia susceptibility loci are associated with a spectrum of speech and developmental delays and behavior problems. Genetics in Medicine *13*, 868–80.
